# Single-cell RNA sequencing unraveled immune-related expression heterogeneity and lymphoid cell development dysregulation in childhood asthma

**DOI:** 10.3389/fimmu.2025.1606650

**Published:** 2026-01-02

**Authors:** Danying Zhu, Guang Li, Lang Yuan, Zeyu Zeng, Na Dong, Chao Wang, Ming Chen, Lijian Xie, Guohui Ding, Libing Shen, Xiaoyan Dong

**Affiliations:** 1 Department of Respiratory, Shanghai Children’s Hospital, School of Medicine, Shanghai Jiao Tong University, Shanghai, China; 2 Institute of Pediatric Infection, Immunity, and Critical Care Medicine, Shanghai Children’s Hospital, Shanghai Jiao Tong University School of Medicine, Shanghai, China; 3 Daozhi Precision Medicine Technology (Shanghai) Co., Ltd, Shanghai, China; 4 Department of Pediatrics, Jinshan Hospital, Fudan University, Shanghai, China; 5 Intelligent Medicine Institute, Fudan University, Shanghai, China; 6 Longhua Hospital, Shanghai University of Traditional Chinese Medicine, Shanghai, China

**Keywords:** single-cell RNA sequencing, hematopoietic stem and progenitor cell, lymphoid cell, childhood asthma, resistin (RETN)

## Abstract

**Backgrounds:**

Asthma is a chronic inflammatory disease affecting airways, usually starting in childhood. Its cause remains unclear.

**Objective:**

We aim to elucidate the role of immune dysregulation in the pathogenesis of pediatric asthma.

**Methods:**

In this study, we used single-cell RNA sequencing to analyze peripheral blood mononuclear cells (PBMCs) from three pediatric asthma patients and four age-matched healthy controls to investigate the cellular etiology of childhood asthma.

**Results:**

The overall expression patterns of PBMCs from the three asthma patients indicate that both innate and adaptive immunity are imbalanced and abnormally activated in childhood asthma. Analysis of hematopoietic stem and progenitor cells (HSPCs) expression profiles further reveals that HSPCs from asthma patients tend to express immunity-related genes earlier. The cell developmental trajectories observed in asthma patients show an abnormal immune cell development pattern. Dysregulated lymphoid lineage development is observed in all three patients but there is no identical abnormal pattern for each patient. Pseudo-time analysis of gene expression demonstrates that JUN, a gene controlling cell cycle progression, is repressed in asthma patients while SPI1, an essential gene for lymphoid lineage development along with six inflammatory response related genes (S100A8, S100A9, S100A12, IL7R, IL32, and CCL5), exhibit various aberrant expression trajectories in asthmatic individuals. S100A8, S100A9, S100A12, and RETN are universally upregulated in various cell types of asthma patients. The analyses of cell-cell communication further elucidate the contributory roles of dendritic cells and CD14^+^ monocytes in the development and heterogeneity of asthma, as they exhibit increased reception and transmission of annexin and resistin signals in the asthma group. The resistin protein-protein interaction network analysis further suggests that SQSTM1, HSPA5 and A2M might serve as the potential therapeutic targets in childhood asthma.

**Conclusions:**

Our scRNA-Seq analyses unveil childhood asthma as a complex disease with immune-related heterogenicities, characterized by dysregulated lymphoid cell development, a common feature that may offer a novel research direction for comprehensively understanding the key molecular mechanisms underlying childhood asthma.

## 1 Introduction

Asthma is a chronic airway disease driven by eosinophils, lymphocytes, macrophages, neutrophils, and epithelial cells exposed to allergens, viruses, or other stimuli. These interactions cause persistent inflammation, reversible airflow limitation, and airway remodeling (1, 2).

While asthma affects individuals of all ages, there are significant variations in presentation, underlying pathophysiology, and treatment response between children and adults (3). Children often exhibit allergic phenotypes with higher rates of eosinophilic inflammation, while adults may present with a broader range of asthma subtypes, including non-eosinophilic variants. The immature yet adaptable immune system in childhood leads to unique cellular responses and potential immune dysregulation, which may explain differences in treatment approaches between age groups (4).

Previous research has mainly focused on the single-cell sequencing of adult asthma (5, 6), but this study applies single-cell technology to investigate childhood asthma to better understand the role of immune abnormalities in asthma pathogenesis. Through single-cell sequencing, specific cell subpopulations can be identified and their contributions to children’s asthma inflammation or responses to treatment may be understood. These findings may help explain why children with asthma exhibit distinct clinical presentations and treatment effectiveness compared to adults, offering new insights into future research and clinical practice.

## 2 Materials and methods

### 2.1 Patients

All participants were recruited between December 2019 and February 2023. We collected three fresh peripheral blood samples from patients with acute asthma. The blood sample was taken on the first days before therapy. The diagnosis of asthma was confirmed using the criteria outlined in the 2023 Global Initiative for Asthma (GINA) report (https://ginasthma.org/2023-gina-main-report/). Atopic status was determined by a total IgE level of 200 IU/ml or higher. For single-cell RNA sequencing (scRNA-seq), we enrolled four healthy individuals who were participating in routine physical examinations and had no recent history of fever, infection, or immunization. Data from three of these individuals have previously been published by our groups (7). We subsequently collected peripheral blood samples from 14 children with asthma who were receiving inhaled corticosteroid therapy during their outpatient follow-up visits, as well as from 14 age-matched healthy children, and measured their plasma RETN levels. The study was approved by the Ethics Committee (Protocol Numbers: 2019R081, 2022R029-F-01). Informed consent was obtained from all participants and their guardians.

### 2.2 Single-cell preparation and sequencing

We collected 2 mL venous blood from each participant using EDTA tubes. Peripheral blood mononuclear cells (PBMCs) were isolated using Ficoll-Paque and density gradient centrifugation within 4 hours. Only samples with over 90% viable cells, as checked by trypan blue, were used. We prepared a cell suspension with about 12,000 cells for analysis. The Chromium Next GEM Single Cell V(D)J Reagent Kits v1.1 from 10x Genomics was used for capturing single cells and constructing libraries. Single-cell RNA sequencing (scRNA-seq) libraries were constructed using the 5’ Library Kits. The prepared libraries were sequenced on an Illumina NovaSeq platform, generating paired-end reads with a length of 2 × 150 base pairs.

### 2.3 scRNA-seq data analysis

We used Cell Ranger to match the sequences from the samples to the human genome (GRCh38). The data was cleaned up with Seurat version 4.0.2. Quality control criteria were applied to ensure appropriate UMI count (200–6000) and limit mitochondrial (< 10%), hemoglobin (< 0.1%), and ribosome genes (< 3%). Samples from the initial phase of the study (C1-C4) were integrated to remove batch effects using an anchor. SCTransform normalization was employed to integrate all seven single-cell samples in order to better handle over-dispersion in single-cell data. Each sample in this study was first SCTransformed individually. All seven samples were then combined together by selecting 2000 integration features. Finally, the combined data were integrated with FindIntegrationAnchors function and then SCTransformed again to create a SCTransformed matrix. The integrated matrix was scaled and principal components derived from Principal Component Analysis (PCA) were used for UMAP projection in a two-dimensional space. Clustering based on shared nearest neighbor graph analysis was performed on PCA-reduced data to identify primary cell types within PBMCs. The optimal number of PCA dimensions for downstream clustering and visualization was determined using the ElbowPlot in Seurat. Cluster identification was performed using the FindClusters function in Seurat, applying a shared nearest neighbor (SNN) graph-based clustering algorithm on the PCA-reduced data, with dimensions set to 1:20 and a resolution of 1.2. UMAP was employed to visualize the cells in a two-dimensional space. Canonical marker genes were examined to refine cell cluster annotations. Cell identities were determined through multimodal reference mapping with SeuratDisk package.

### 2.4 Differential expression, functional enrichment analysis, pseudo-time analysis and pseudo-bulk RNA-seq analysis

The FindAllMarkers function in the Seurat package was used to calculate marker genes for each cluster. The FindMarkers function was used to find differentially expressed genes (DEGs) among two cluster. The log2 fold change threshold was set to be 0.25 (about 1.2 times of difference) and the minimum percentage of expressed cells was 0.1 (10% of total cells in one sample). The DEGs with the adjusted P value smaller than 0.01 were used for Venn diagram display. The DEGs were identified by comparing each asthmatic sample to all pooled controls in order to identify the DEGs in each asthmatic sample. The clusterProfiler package (version 3.16.0) was utilized for over-representation analysis of DEGs, with a false discovery rate (FDR) threshold of < 0.05. Gene Ontology (GO), KEGG pathways, and hallmark gene sets from the MSigDB database (version 7.1) were employed as gene function databases to assess enrichment of specific biological functions or pathways among DEGs. Pseudo-time analysis of cell differentiation trajectories for each sample dataset was performed using the Monocle R package (8). The unsupervised procedure termed “dpFeature” (differential pattern feature) in Monocle 2 was used to identify the genes that define a biological process. The cell differentiation trajectory analysis was also performed for each cellular lineage in healthy controls and three asthma patients. CD4^+^ T, CD8^+^ T and other T cells were classified as the T cell lineage. B cells and plasma blast cells were classified as the B cell lineage. CD14^+^ and CD16^+^ monocytes were classified as the monocyte lineage. Dimension reduction was conducted using 2 max components and the “DDRTree” method. Cell-cell communication analysis was carried out using CellChat version 1.6.1 (9). The netAnalysis_signalingChanges_scatter function in CellChat was used to visualize the differential outgoing and incoming signaling associated with one cell group. The pseudo-bulk RNA-Seq analysis was performed as follows. The average gene expression value for all cells within each sample was calculated with “Average Expression” function in Seurat. Seven single-cell average expression results were integrated together and then used as pseudo-bulk RNA-seq data for PCA analysis.

### 2.5 Human serum resistin enzyme-linked immunosorbent assay analysis and PPI network analysis

EDTA-anticoagulated whole blood was transferred to the laboratory and processed immediately after collection. Centrifuge samples for 15 minutes at 1000 x g at 4 °C within 30 minutes of collection and stored at -80 °C. We followed a human resistin ELISA kit (Signalway Antibody, Maryland, USA, Catalog No: EK2351) protocol to analyze peripheral blood serums.

We downloaded the interactome data from MENTHA database and used them to perform the protein-protein interaction network analysis for RETN (10). Cytoscape 3.9.1 was used to generate the undirected PPI network for RETN and their interaction proteins (11). The sub network hub genes were examined with their function in GO analysis.

## 3 Results

### 3.1 Characteristics of the study subjects for asthma patients and healthy controls

In our single-cell RNA analysis study, we included a cohort of 7 childhood subjects, comprising 3 children experiencing asthma exacerbations and 4 healthy individuals without asthma. The mean age of the participant children was 6.3 ± 2.5 years, while the healthy controls had a mean age of 4.7 ± 3.2 years (**Supplementary Table 1**). An additional group of 14 asthma patients, consisting of 9 males and 5 females with a mean age of 10.0 ± 2.3 years, and 14 healthy donors, also with 9 males and 5 females, had a mean age of 7.3 ± 2.2 years, were included in the study. Allergic testing in asthmatic children revealed sensitization to house dust mites (**Supplementary Table 2**).

### 3.2 Single-cell RNA profiling of PBMCs in healthy individuals and asthma patients

**Figure 1:**
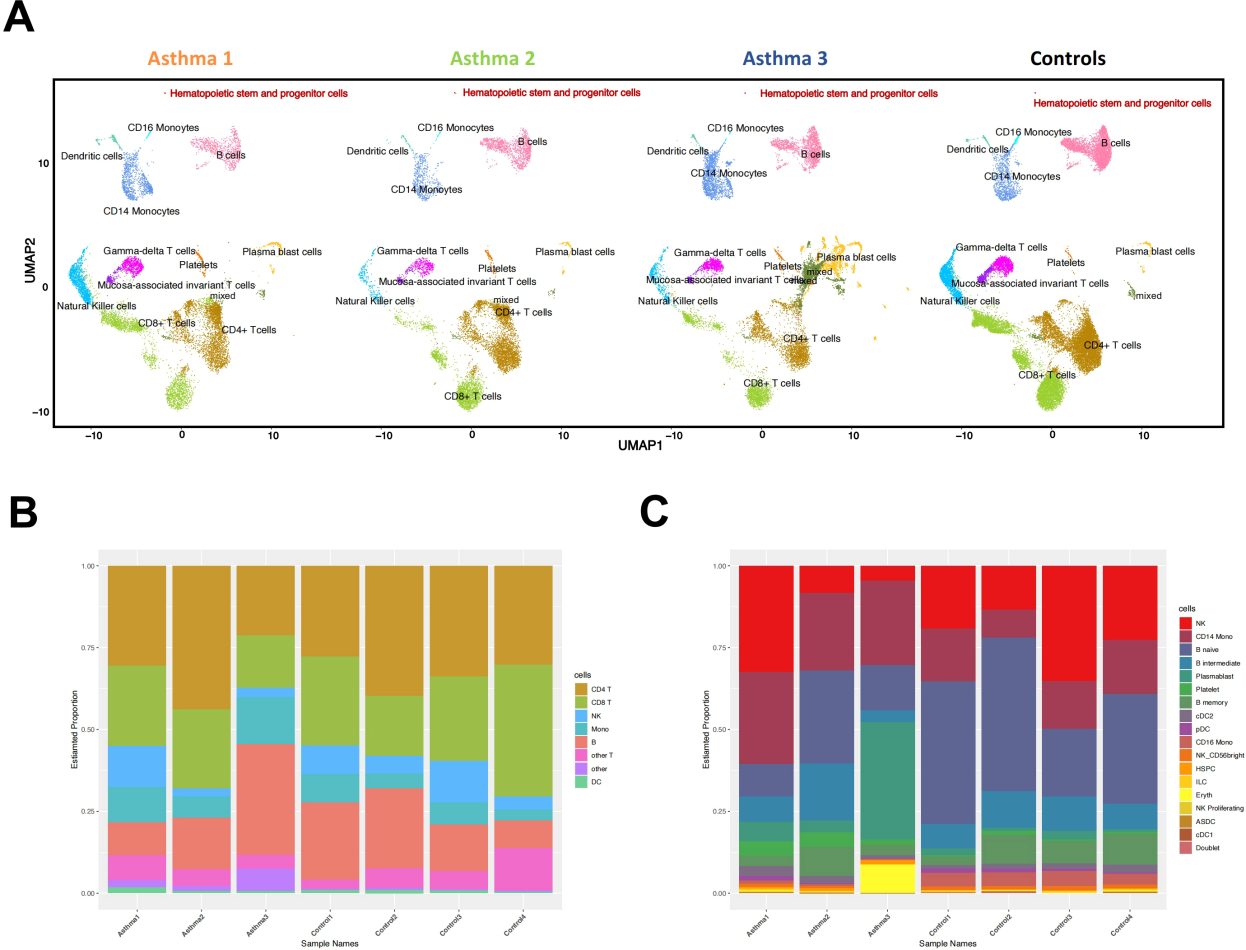
Single-cell profiling of PBMCs in seven samples. **(A)** The integration single-cell profiling analysis of 7 samples including 4 healthy controls and 3 asthma patients (Asthma 1, Asthma 2 and Asthma 3). **(B)** The percentage bar chart shows the continent of different cell types in each sample. **(C)** The percentage bar chart shows the different cell types except T cells in each sample.

We collected peripheral blood samples from 3 patients with asthma onset (Asthma 1-3) and age-matched healthy controls (Control 1-4, **Supplementary Table 1**). The sequencing data yielded approximately 5000–14000 cells per sample. After quality control, a total of 34,354 cells were detected in the asthma patient group and 22,398 cells in the healthy group. We used the average expression value from each gene in scRNA-Seq to simulate the RNA-Seq expression profile (pseudo-bulk RNA-Seq) for seven samples and performed PCA analysis using 5000 most differentially expressed genes. The pseudo-bulk RNA-Seq result shows that four control samples were grouped together and three asthma samples were scattered and far away from control samples, especially Asthma 3 (**Supplementary Figure 1A**). It indicates that four control samples have a similar expression profile and can be unified as a single control dataset, while three asthma samples have a heterogenous expression background. Using Seurat 4.0.2, we clustered the cells across samples and visualized them in two-dimensional space (**Figure 1A**). Our study identified 12 major cell types including B cells, CD4^+^ T cells, CD8^+^ T cells, CD14^+^ monocytes (CD14 mono), CD16^+^ monocytes (CD16 mono), dendritic cells (DC), mucosa-associated invariant T cells (MAIT), gamma-delta cells (gdT), hematopoietic stem and progenitor cells (HSPC), natural killer cells (NK), plasma blast cells (Plasmablast), and platelets. Each cell type was validated using PBMC multimodal reference object and canonical gene markers (**Supplementary Figures 1C–F**). The distribution of major cell types for each sample is shown in **Figure 1B**. Further classification excluding CD4^+^ T and CD8^+^ T cell types revealed a significant increase in CD14^+^ monocytes and a significant decrease in CD16^+^ monocytes among asthma patients compared with healthy controls (**Figure 1C**). **Supplementary Figure 1B** provides detailed classification of T cell types for each sample. Our study revealed that, compared with healthy controls, the upregulated genes in CD4^+^ and CD8^+^ T cells from children with asthma are predominantly enriched in immunity, adaptive immune response, immunoglobulin complex, antigen binding, and inflammatory response (**Supplementary Tables 3–8**).

### 3.3 Heterogenous expression features of all cells and HSPCs in asthma patients

**Figure 2:**
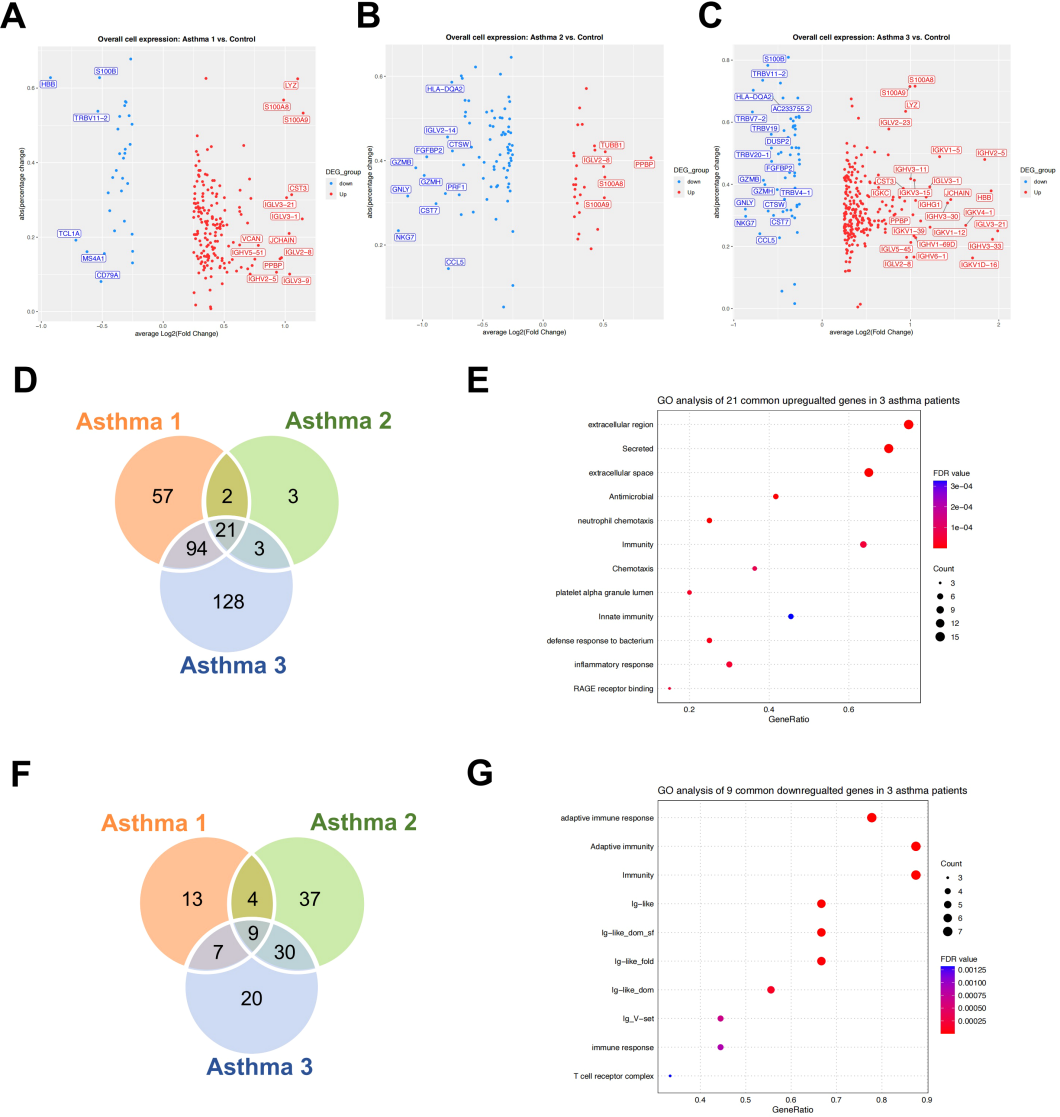
The analysis of overall upregulated and downregulated genes in asthma patients. **(A)** Volcano plot of upregulated and downregulated genes in Asthma 1. **(B)** Volcano plot of upregulated and downregulated genes in Asthma 2. **(C)** Volcano plot of upregulated and downregulated genes in Asthma 3. (Dots for statistically significant genes and gene names for highest fold changes). **(D)** Venn gram of the upregulated genes in 3 asthma patients. **(E)** GO term enrichment analysis of the specific upregulated genes in 3 asthma patients. **(F)** Venn gram of the downregulated genes in 3 asthma patients. **(G)** GO term enrichment analysis of the specific downregulated genes in 3 asthma patients.

We compared gene expression levels between asthma patients and controls, identifying DEGs for each patient (**Figures 2A-C**). There were 21 common upregulated genes among all three patients (**Figure 2D**). GO analysis revealed that these shared upregulated genes are involved in extracellular region, secretion, and antimicrobial function (**Figure 2E**). GO analyses of the specific upregulated genes in Asthma 1 and Asthma 3 indicated different molecular phenotypes between the two patients (**Table 1**). Asthma 1 exhibits the specific expression of the genes related to immune response and positive regulation of T cell migration while Asthma 3 has an excessive expression of immunoglobulin. The upregulated genes in Asthma 2, namely IGHV3-73, CDK6, and NELL2, are not sufficient for GO analysis (**Table 1**). There are 9 common downregulated genes among the three asthma patients (**Figure 2F**). GO analysis reveals their involvement in adaptive immunity and T cell receptor signaling pathway (**Figure 2G**). Asthma 1 exhibits the specific repression of external side of plasma membrane and B cell receptor signaling pathway expression; Asthma 2 exhibits the specific repression of immunoglobulin complex; Asthma 3 exhibits the specific repression of T cell receptor complex (**Table 2**).

**Table 1:** GO results for overall specific upregulated genes in each asthma patient.

Case	GO terms	Genes underlying GO terms	Num of genes	P-value
Asthma 1	GO:0006955~immune response	IGLV2-11, IL32, SPN, TGFBR3, IGLV3-9, FCGR3A, IGLV6-57, CCL5, GZMA, CCL4, IGLV2-18, CST7	12	1.04E-07
	GO:2000406~positive regulation of T cell migration	SPN, APP, CCL5	3	0.0014
Asthma 2	N.A.	N.A.	N.A.	N.A.
Asthma 3	GO:0019814~immunoglobulin complex	IGHV2-70, IGHV4-31, IGHV6-1, IGKV2D-30, IGHV3-43, IGHV4-34, IGHV1-2, IGHV4-59, IGHV4-39, IGHV1-46, IGLV5-45, IGKV1-39, IGKV1-17, IGLV2-14, IGLV7-46, IGLV1-44, IGKV1-12, IGHV1-69D, IGHV4-61, IGKV1-9, IGHV3-74, IGKV2D-40, IGHV3-33, IGHV3-11, IGHV3-15, IGKV1D-16, IGHV2-26, IGLV7-43, IGKV1D-13, IGHV1-18, IGKV2D-29, IGKV2D-28, IGKV1-27, IGLV2-23	34	6.26E-43

**Table 2:** GO results for overall specific downregulated genes in each asthma patient.

Case	GO terms	Genes underlying GO terms	Num of genes	P-value
Asthma 1	GO:0009897~external side of plasma membrane	FCRLA, CD79B, FCER2, CD74, CD79A, CD19, MS4A1	7	2.49E-08
	GO:0050853~B cell receptor signaling pathway	CD79B, CD79A, BANK1, CD19, MS4A1	5	2.58E-08
Asthma 2	GO:0019814~immunoglobulin complex	IGHV3-74, IGHV3-53, IGHV1-2, IGHV4-39, IGHV1-18, IGLV1-51, IGLV3-27, IGLV1-40, IGKV1-27, IGLV6-57, IGLV1-36, IGLV2-14, IGLV3-21, IGLV1-44, IGKV2-30	15	1.79E-21
Asthma 3	GO:0042101~T cell receptor complex	TRBV7-9, CD8B, TRBV5-1, TRBV4-2, TRAV12-2, TRBV3-1, TRBV12-4, TRBV27, TRBV6-5, TRBV6-6, TRAV8-3, TRAV29DV5	12	3.87E-20

**Figure 3:**
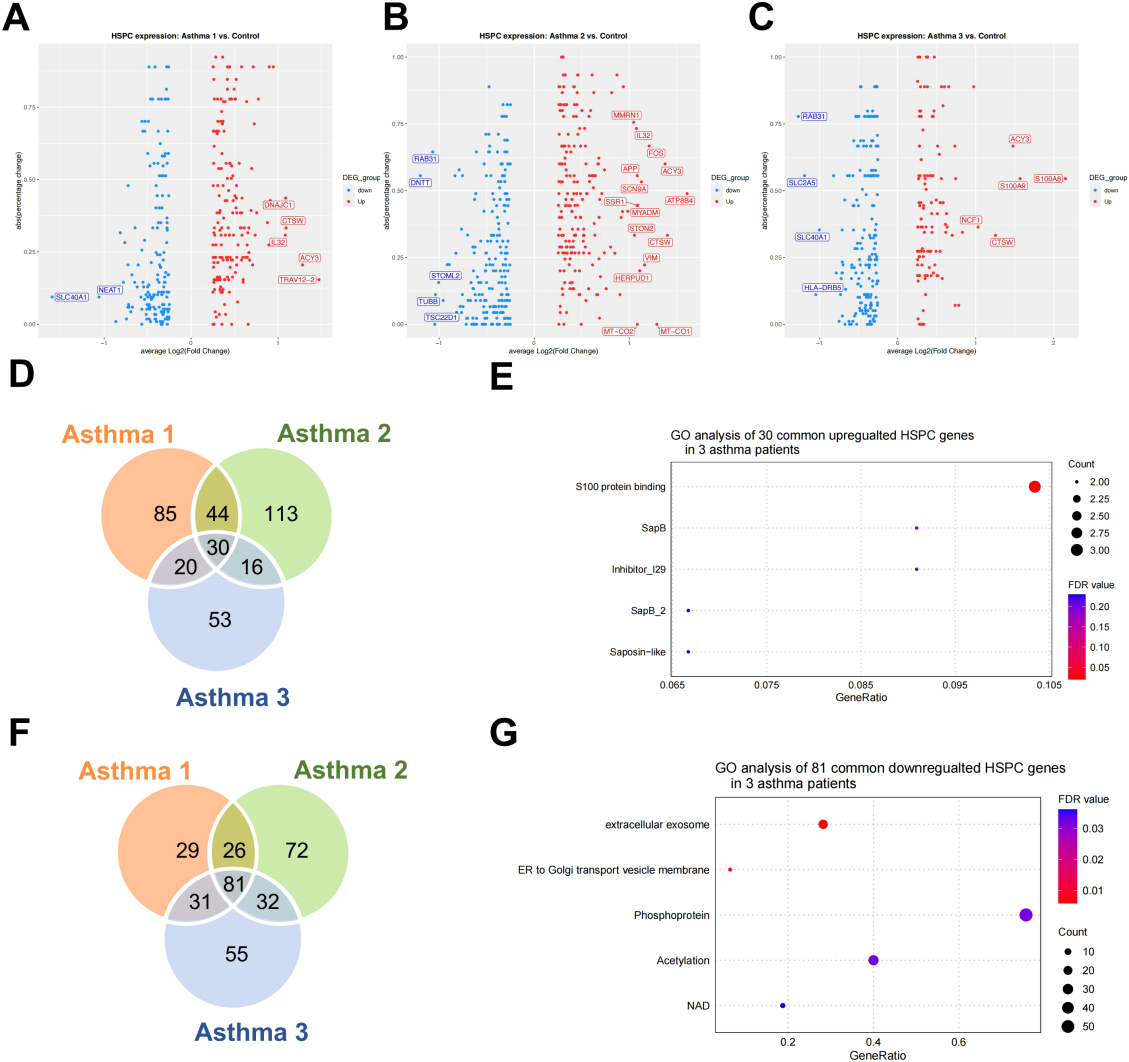
The analysis of upregulated and downregulated genes in asthma patients’ HSPCs. **(A)** Volcano plot of upregulated and downregulated HSPC genes in Asthma 1. **(B)** Volcano plot of upregulated and downregulated HSPC genes in Asthma 2. **(C)** Volcano plot of upregulated and downregulated HSPC genes in Asthma 3. (Dots for statistically significant genes and gene names for highest fold changes). **(D)** Venn gram of the upregulated genes in 3 asthma patients’ HSPCs. **(E)** GO term enrichment analysis of the specific upregulated genes in 3 asthma patients’ HSPCs. **(F)** Venn gram of the downregulated genes in 3 asthma patients’ HSPCs. **(G)** GO term enrichment analysis of the specific downregulated genes in 3 asthma patients’ HSPCs.

We further examined the expression features of HSPCs in asthma patients, as all other cell types are derived from them. DEGs in HSPCs were identified for each asthma patient (**Figures 3A–C**). GO analysis revealed that the shared set of 30 upregulated HSPC genes among all three patients mainly participate in S100 protein binding (**Figures 3D-E**). Notably, specific gene expressions observed include adaptive immunity and T cell receptor complex expression for Asthma 1; immune response-related gene expression for Asthma 2; extracellular exosome and immunity-related gene expression for Asthma 3 (**Table 3**). Interestingly, T cell receptor genes were found to be consistently upregulated across all three asthma patients despite not being statistically significant in the HSPCs of both Asthma 2 and Asthma 3. The three asthma patients share a total of 81 downregulated HSPC genes (**Figure 3F**). GO analysis reveals that the shared downregulated HSPC genes primarily contribute to extracellular exosomes and ER-to-Golgi transport vesicle membranes in terms of cellular components (**Figure 3G**). No significant GO results were found for the specifically downregulated HSPC genes in Asthma 1. Patient-specific repression occurs for extracellular exosome-related and endoplasmic reticulum-related gene sets respectively (**Table 4**).

**Table 3:** GO results for specific upregulated HSPC genes in each asthma patient.

Case	GO terms	Genes underlying GO terms	Num of genes	P-value
Asthma 1	GO:0002250~adaptive immune response	IL4I1, TRGV2, SYK, TRBV4-1, CD8B, IGLV3-21, LILRB2, TRBV7-6, HLA-DOA, HLA-DQA2, TRBV7-4, TRAV25	12	1.87E-06
	GO:0042101~T cell receptor complex	TRGV2, SYK, TRBV4-1, CD8B, TRBV7-6, TRBV7-4, TRAV25	7	2.03E-05
Asthma 2	GO:0006955~immune response	SPN, CIITA, TRBV7-9, CD40LG, IL1B, IGKV3-15, TNFSF10, IGHV4-39, CST7, HLA-DOB, IFI44L, TNFSF13B	12	5.32E-05
Asthma 3	GO:0070062~extracellular exosome	ITGB1, HBB, MYL6B, RHOC, LYZ, MPO, ACTG1, JCHAIN, PSMB6, SCPEP1, LDHA, BASP1, PRKAR2B, GNG7, BLVRB, MAN1A1, CKB, CD14, S100A8	19	9.18E-07
	GO:0045087~innate immune response	HCK, NCF2, LY86, S100A12, TRGV9, CD14, S100A8, JCHAIN	8	2.06E-04

**Table 4:** GO results for specific downregulated HSPC genes in each asthma patient.

Case	GO terms	Genes underlying GO terms	Num of genes	P-value
Asthma 1	N.A.	N.A.	N.A.	N.A.
Asthma 2	GO:0070062~extracellular exosome	CHID1, IGHM, PCNA, SLC44A1, SHMT2, GSTP1, TUBB, TNFSF13, AKR1C3, PEBP1, TXN, NUDT5, CORO1B, JCHAIN, EEF1A1, ADGRG1, RPS18, S100A6, TSPO, GLUL, GAPDH, PPIA, RAN (23)	23	1.28E-06
Asthma 3	GO:0005783~endoplasmic reticulum	TMED9, MPIG6B, RPN2, HSPA5, RPN1, DERL2, HERPUD1, HSP90B1, DNAJC3, MLEC, CALR, CRELD2, WDFY4, DNASE1L3 (14)	14	3.16E-06

In expression feature analyses, we observe universal upregulation of S100A8, S100A9, S100A12, and RETN in various cell types of asthma patients, particularly S100A8 and S100A9 (**Supplementary Figure 9A**). While S100A8, S100A9, and S100A12 are involved in inflammatory response, RETN exhibits antibacterial activity (12–14).

### 3.4 Single-cell trajectory reconstructions of PBMCs in healthy controls and 3 asthma patients

**Figure 4:**
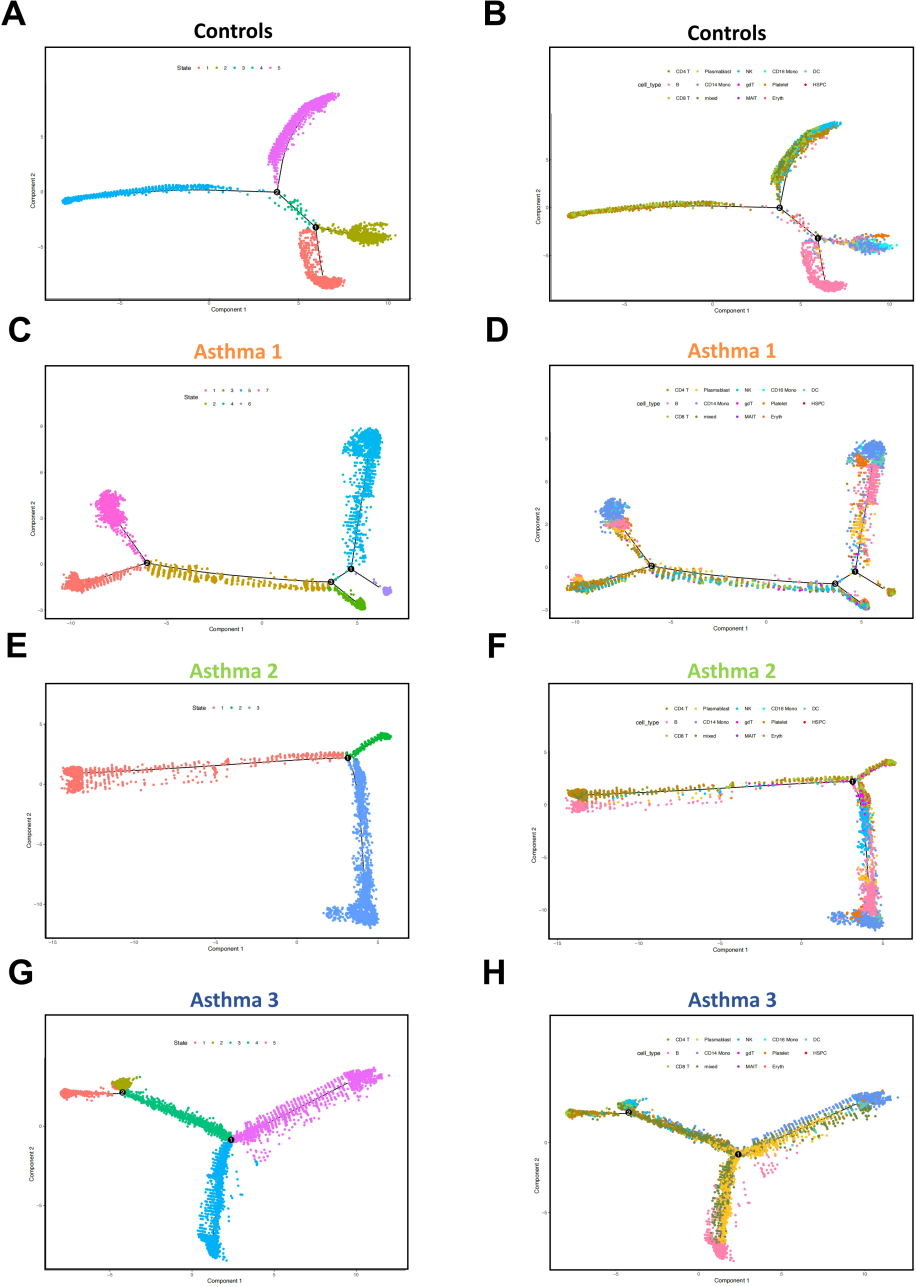
Pseudo-time analysis of all cells in four sample groups. **(A)** The differentiation trajectory of all cells in healthy controls by state. **(B)** The differentiation trajectory of all cells in healthy controls by cell types. **(C)** The differentiation trajectory of all cells in Asthma 1 by state. **(D)** The differentiation trajectory of all cells in Asthma 1 by cell types. **(E)** The differentiation trajectory of all cells in Asthma 2 by state. **(F)** The differentiation trajectory of all cells in Asthma 2 by cell types. **(G)** The differentiation trajectory of all cells in Asthma 3 by state. **(H)** The differentiation trajectory of all cells in Asthma 3 by cell types.

The expression features of asthma patients’ HSPCs indicate abnormal repression of immunity and T cell receptor genes. Given that HSPCs give rise to other cells, it is worth investigating the developmental states of cells in asthma patients. Single-cell trajectory analysis can help reconstruct cell developmental paths based on specific variable genes. The analyses reveal that healthy controls have 5 distinct developmental states for PBMCs, while asthma patients exhibit different numbers: 7, 3, and 5 (**Figures 4A, C, E, G**). All cell types are plotted on each trajectory in every sample (**Figures 4B, D, F, H**).

Based on the major cell types and numbers, state 1 in healthy controls can be classified as B lineage, state 2 as monocyte lineage, state 3 as a mixed lineage (B and T), state 4 as T lineage, and state 5 as T-NK lineage (**Figure 4B**, **Supplementary Figure 2A**). Myeloid lineage (monocytes) and two lymphoid lineages (B cells and T cells) are clearly differentiated in the PBMCs of healthy controls. However, clear cell development patterns disappear in three asthma patients. Each patient exhibits unique cell developmental patterns different from healthy controls. Asthma 1 has seven states with four of them being T lineages based on major cell numbers (state 1, 2, 3, and 6; **Figure 4D**, **Supplementary Figure 2B**). Asthma 2 has only three states with a portion of B cells mixed with T cells (state 1; **Figure 4F**, **Supplementary Figure 2C**). Asthma 3 has five states but B cells are mixed with T cells in state 3(state 1; **Figure 4H**, **Supplementary Figure 2D**). Although each childhood asthma patient shows different cell developmental states, abnormal lymphoid lineage development is observed in all three patients (multiple T lineage states or no clear B lineage state). This result aligns with expression feature analyses indicating that adaptive immunity is repressed in asthma patients. Insufficient development of T cells and B cells naturally leads to inadequate adaptive immunity. We also separately analyzed the developmental states of the T, B and monocyte lineages in each sample group. Compared to the developmental states of the T cell, B cell and monocyte lineages in control group, all three asthma patients showed the underdeveloped trend in each lineage, although the trend pattern was heterogeneous among three patients (**Supplementary Figures 3–5**).

### 3.5 Pseudo-time expression dynamic analyses of proto-oncogenes and inflammatory response related genes

The abnormal cell development in asthma patients should be accompanied by abnormal gene expression patterns. JUN gene is a transcription factor regulated by various extracellular stimuli including peptide growth factors, pro-inflammatory cytokines, and even UV irradiation (15). SPI1 gene is also a transcription factor regulating gene expression during myeloid and B-lymphoid cell development (16). Both of them are proto-oncogenes essential for cellular differentiation (17, 18). We further include six inflammatory response related genes in pseudo-time expression dynamic analyses. Their express dynamics were plotted along cell developmental trajectories in both healthy controls and three asthma patients.

**Figure 5:**
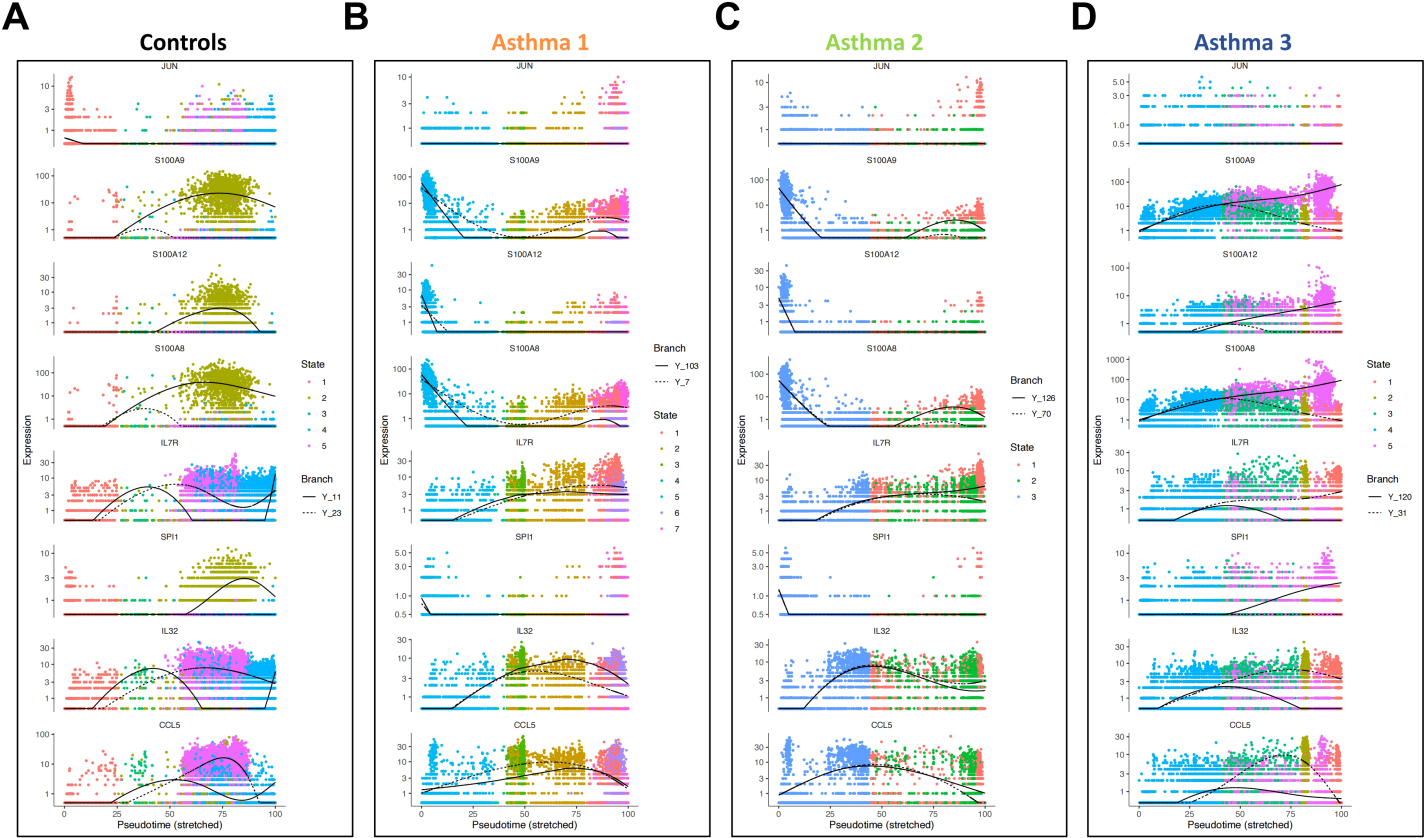
Pseudo-time analyses of expression dynamics of two cell-fate and six inflammation related genes in four sample groups. **(A)** Expression dynamics two cell-fate and six inflammation related genes in healthy controls. **(B)** Expression dynamics two cell-fate and six inflammation related genes in Asthma 1. **(C)** Expression dynamics two cell-fate and six inflammation related genes in Asthma 2. **(D)** Expression dynamics two cell-fate and six inflammation related genes in Asthma 3.

In healthy controls, we observed the onset expression of JUN in the early pseudo-time state and a peak expression of six inflammatory response related genes in the early-middle pseudo-time state. SPI1 is expressed in the late pseudo-time state (**Figure 5A**). In Asthma 1 and Asthma 2, the expression dynamics of JUN, SPI1 and six inflammatory response related genes are almost the same (**Figures 5B, C**). JUN’s onset expression is repressed while SPI1 exhibits an onset expression in the early pseudo-time state. S100A8, S100A9, and S100A12 show an early onset expression rather than a peak expression in early-middle pseudo-time state. IL7R, IL32, and CCL5 start to express in early-middle pseudo-time state as healthy controls, but they show a persistent expression level instead of an obvious expression peak. Notably, IL7R, IL32, and CCL5 all participate in the biological process of negative regulation of T cell apoptotic process (GO:0070233). It implicates that the T cells have a prolonged cell survival in Asthma 1 and Asthma 2. Asthma 3 shows a different expression dynamic pattern from healthy controls, Asthma 1, and Asthma 2 (**Figure 5D**). In Asthma 3, JUN is also repressed while the expression patterns of IL7R, IL32, and CCL5 are more similar to healthy controls. S100A8, S100A9, S100A12 and SPI1 show a persistent expression level without decline. Since SPI1 activates gene expression during myeloid and B-lymphoid cell development and S100 protein family plays a role in cell growth and differentiation, their persistent expression pattern indicates an abnormal lymphoid cell development fate in Asthma 3.

### 3.6 DCs function as a signaling hub in cell communication and play a role in asthma heterogeneity

**Figure 6:**
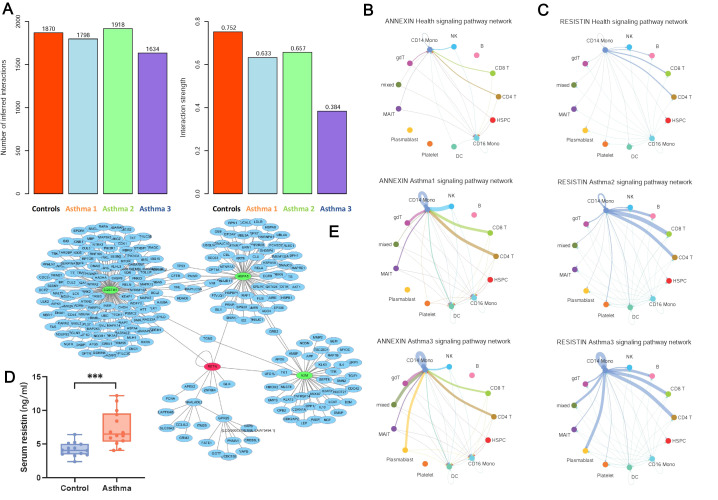
Cell communication pattern for asthma patients and healthy controls, serum resistin expression level and its PPI network. **(A)** The number of cell-cell interactions and interaction strength for four sample groups. **(B)** The annexin signaling pathway network of three sample groups. The annexin signaling pathway network in all cell types is shown for healthy controls, Asthma 1 and Asthma 3. The line thickness represents the signal strength, e.g. CD14^+^ monocytes have an annexin strength of 0.00006 in control, 0.0009 in asthma 1, and 0.0007 in asthma 3, respectively. The signal strength was estimated with the gene expression data of ligand-receptor pair. **(C)** The resistin signaling pathway network of three sample groups. The resistin signaling pathway network in all cell types is shown for healthy controls, Asthma 2 and Asthma 3. The line thickness represents the signal strength, e.g. CD14^+^ monocytes have a resistin strength of 0.0005 in control, 0.0025 in asthma 2, and 0.006 in asthma 3, respectively. The signal strength was estimated with the gene expression data of ligand-receptor pair. **(D)** The expression of RETN gene in two sample groups. The level of serum resistin between asthma patients and healthy controls (***p < 0.001). **(E)** Protein–protein interaction network of RETN, its directly interacting genes and their neighbors. RETN is marked with red color and SQSTM1, HSPA5, and A2M are marked with light green color. RETN has a network degree of 8. SQSTM1, HSPA5, and A2M have a network degree of 109, 44, and 37, respectively. Network degree represents the number of neighbors (genes) connected to a hub, which is a core metric for measuring node importance and connection strength in complex network analysis.

We performed cell communication analyses for both healthy controls and asthma patients to investigate their cell-cell interactions. The number of interactions and interaction strength for healthy controls and three asthma patients are shown in **Figure 6A**. Generally, asthma patients have a weaker interaction strength than healthy controls and Asthma 3 have the smallest number of interactions and the weakest interaction strength among four samples. Among the major immune cell types, we find that dendritic cells have the largest number of both incoming and outgoing interaction numbers (**Supplementary Figures 6A–D**). DCs are known to be the messenger cells communicating between innate and adaptive immune systems (19). They are also known as the antigen presenting cells which interact with T cells and B cells to activate and regulate the adaptive immune response (20). Although DCs don’t have the strongest interaction strength among the major immune cell types (**Supplementary Figures 7A-D**), they surely function as a signaling hub in cell communication based on cell communication analyses.

We further examined the DCs’ signaling differences between healthy controls and asthma patients. Using healthy controls as a signaling background, the signaling changes were detected in three asthma patients. The DCs of Asthma 1 have a specific incoming signal of annexin (**Supplementary Figure 8A**). The DCs of Asthma 2 has a specific outgoing signal of resistin (**Supplementary Figure 8B**). The DCs of Asthma 3 have a specific incoming signal of annexin and a specific outgoing signal of resistin (**Supplementary Figure 8C**). Annexin A1, A2 and A5 have a role in the regulation of inflammation activation and may serve as the potential biomarkers for asthma (21–23). They all show an elevated expression tendency in asthma cases (**Supplementary Figure 9B**). Resistin also serves a potential biomarker for asthma and is related to inflammation as well (24, 25). Our results show that annexin and resistin are actually two opposite directional signals for the DCs of asthma patients. One of them is a sufficient predictor of asthma and both of them indicate a severe symptom of asthma, since Asthma 3 is an acute severe asthma patient. Both annexin and resistin show a self-reinforce trend in the CD14^+^ monocytes of asthma patients (**Figures 6B-C**). The upregulated signaling interactions in asthma are mainly centered on the molecular component of external side of plasma membrane and cell adhesion molecules (GO:0009897 and hsa04514, **Supplementary Figures 10A–C**). Furthermore, we found that CLEC2B-KLRB1 and LGALS9-CD44 signaling interactions are downregulated in all three asthma patients (**Supplementary Figures 11A–C**). CLEC2B-KLRB1 interaction mediates the activation of NK cells and monocytes (26). LGALS9-CD44 interaction enhances the stability and function of adaptive regulatory T cells (27). The down-regulated CLEC2B-KLRB1 and LGALS9-CD44 interactions proposes a weakened functions of NK cells, monocytes and T cells in asthma patients.

The expression of annexin and resistin signaling pathways are examined in all cell types among four sample groups (**Supplementary Figures 12A, B**). The results show that both signaling pathways are more prominent in asthma groups than control group, especially for CD14^+^ monocytes.

### 3.7 RETN and its directly interact genes with therapeutic potential

We present the ELISA results showing that RETN is highly expressed in all 14 asthma cases examined (**Figure 6D**). The figure illustrates these findings, confirming RETN as a potential biomarker for childhood asthma. We then used the protein-protein interaction information to examine the role of RETN in a network context. Although RETN is just a cysteine-rich secreted protein, its closest interaction protein companions may function as important network hubs for possible therapeutic values. Using PPI information, we detected 9 proteins directly interacting with RETN and their 224 network neighbor genes. Three RETN direct PPI neighbors, SQSTM1, HSPA5, and A2M, are network hub genes of their own PPI networks (**Figure 6E**). Sequestosome 1 (SQSTM1, also known as p62) serves as a binding partner for the lymphocyte-specific protein tyrosine kinase. SQSTM1 regulates activation of the nuclear factor kappa-B (NF-kB) signaling pathway. Formoterol reduces the activation of the NLRP3 inflammasome and GSDMD-mediated pyroptosis in microglia by enhancing the inhibition of IκBα/NF-κB, promoting selective autophagy dependent on SQSTM1/p62 (28). HSPA5, is also known as immunoglobulin heavy chain-binding protein, commonly referred to as Bip or GRP78, is a resident chaperone in the endoplasmic reticulum (ER) that assists in the folding and assembly of proteins (29). Alpha-2-macroglobulin (A2M) regulates inflammatory cytokines and disrupts inflammatory cascades (30). SQSTM1 is not a clearly identified target for any drug so far, but HSPA5/A2M are clear targets for multiple drugs such as Acetylsalicylic acid and Becaplermin (31, 32). Thus, HSPA5/A2M could be exploited as novel drug targets for childhood asthma while SQSTM1 might serve as a potential target or therapeutic responder.

## 4 Discussions

In individuals with asthma, the immune system has developed an exaggerated response to allergens, leading to chronic inflammation and airway hyperresponsiveness. During an asthma exacerbation, this preexisting allergic airway disease is further aggravated by additional inflammatory stimuli or triggers. Single-cell sequencing studies in adult asthma have focused on adaptive immunity and reveal that CD4^+^ T cells constitute the dominant population among PBMCs, exhibiting pro-inflammatory characteristics (33). The overall expression features among three asthma patients’ PBMCs demonstrate that both innate and adaptive immunity are imbalanced and abnormally activated in childhood asthma. However, individual differences still exist. Asthma 1 shows increased T-cell immunity, suggesting a Th2-high response linked to eosinophilic inflammation. Targeting T-cell pathways with specific biologics (like anti-IL-5, IL-4Rα, or IL-13) (34) could help this patient. Asthma 3 may indicate a stronger IgE-mediated allergic response. IL-4 activated B cells can switch to IgE, causing mast-cell and basophil-mediated bronchial hyperreactivity (34). Therapies targeting IgE, such as omalizumab, may be effective for these patients. Asthma 2 shows no significant T-cell or B-cell activation. This suggests immune tolerance or a less active disease state (35). This patient might benefit from monitoring and environmental control rather than aggressive immunotherapy. Understanding these different immune profiles is crucial for creating personalized treatment strategies.

Maintenance of blood cell balance relies on hematopoietic stem cells, which possess the dual abilities to perpetually self-renew and to differentiate into all blood lineages. Circulating hematopoietic stem and progenitor cells (cHSPCs) contribute to immune surveillance by generating tissue-resident innate immune cells, both under normal conditions and during localized infections (36). Innate immune cells and their progenitors retain long-term epigenetic memory of past infections or inflammation, reshaping future immune balance and responses (37, 38). Circulating HSPCs may also serve as a peripheral biomarker that reflects the status of bone-marrow-resident HSPCs (39, 40). In our study, the active S100 protein and hyo-immune tendency in the HSPCs of asthma patients seem to lead to over innate immunity and low adaptive immunity for all cells of asthma patients. In HSPCs of COVID-19, late-phase CD14^+^ monocytes retain elevated levels of inflammatory mediators such as S100A8 and S100A9 relative to both healthy controls and early-phase samples, underscoring their progression toward a more differentiated, pro-inflammatory phenotype (40). We found that S100A8, S100A9, and S100A12-proteins involved in innate immunity-were elevated in asthmatic children, although it remains unclear whether similar changes occur in bone-marrow-resident HSPCs, the future study includes bone marrow samples might give us much clearer views for this question.

The single-cell trajectory analyses further demonstrate the cell developmental heterogeneities among asthma patients. The abnormal lymphoid lineage development is observed in all 3 patients. The expression of T cell receptor genes is repressed in the HSPCs of all three asthma patients. The delayed expression of T cell receptor genes could lead to HSPC’s descendant cells difficult to receive the lymphoid development signals. In Asthma 1, such deficiency is responded with the development of multiple T cell states by Asthma 1’s immune system, which seems to compensate for the lack of proper T cell developmental state. In Asthma 2 and Asthma 3, the delayed expression of T cell receptor genes is less severe than that in Asthma 1, which lead to the underdevelopment of lymphoid lineage. The abnormal single-cell developmental trajectories in asthma patients are also accompanied with the key genes’ aberrant expression dynamics. JUN and SPI1 are two proto-oncogenes, both of which have multiple functions in cell fate decisions (18, 41). The expression of JUN is repressed in the early stage of cell trajectories for all three asthma patients. It indicates that the cell developmental fate of asthma patients has the trouble at the very beginning, which is consistent with the expression abnormality in the HSPCs of asthma patients. In Asthma 1 and Asthma 2, the repression of JUN seems to be replaced by the expression of SPI1. In Asthma 3, the beginning of the expression of SPI1 is consistent with that in healthy controls, but the expression of SPI1 shows no decline like healthy controls. It implies that the developmental fates of distal cells in Asthma 3 could be influenced by SPI1.

The cell-cell communication analyses further revealed the possible mechanisms underlying the etiology of asthma. Although the number of cell-cell interactions are similar in healthy controls and asthma cases, the strength of cell-cell interactions is much weaker in asthma patients, especially in Asthma 3. The overall weak signaling strength is a clear sign of cell-cell communication disorder in asthma. It is in accordance with the abnormal cell developmental trajectories of asthma. DCs induce primary immune responses by processing and presenting antigen material on the cell surface of T cells (42). They function as the signaling hub communicating between innate and adaptive immune systems (43). Two aberrant signals, annexin and resistin, are found for the DCs of asthma, both of which play a part in inflammatory response. In our cases, each asthma patient has his or her aberrant signaling pattern, annexin for Asthma 1, resistin for Asthma 2, and both annexin and resistin for Asthma 3. Resistin as an outgoing signal from DCs has been reported as a predictor of asthma (24) and related to disease severity (44). The common receptors include Toll-like receptor (TLR) 4 and adenylyl cyclase-associated protein 1 (CAP1), CAP1 has been identified as the key functional receptor through which resistin drives inflammatory responses in human monocytes (45). Our ELISA results show that its expression can be detected in 14 enlisted childhood asthma patients. Plasma resistin levels were higher in adults with asthma than in controls (24), consistent with our findings.

Asthma 3 exhibits severe symptoms compared to the mild symptoms of Asthma 1 and Asthma 2. He had stayed in pediatric intensive care unit for a short period of time. Asthma 3 has two extra cell clusters, they are annotated as mixed cells and plasma blast cells(**Figure 1A**). The UMAP plot shows that the mixed cells and plasma blast cells in Asthma 3 are stemmed from CD4^+^ T cells. Thus, the plasma blast cells in Asthma 3 likely have dual cell identities of both plasma blast cells and T cells. That Asthma 3’s plasma blast cells show a duality of T cell identity could be linked to the SPI1’s aberrant expression pattern. SPI1 might influence the developmental fate of plasma blast cells in Asthma 3 and further exacerbate the patient’s symptoms. Whether this phenomenon of mixed cell identities is common in severe childhood asthma is worth investigation in the future.

Our study has some limitations. The relatively small sample size and the lack of pulmonary-function and allergen testing in the validation cohort controls may limit the generalizability of our findings. Future studies with larger, comprehensive phenotype cohorts are therefore required to confirm these results. Given the clinical design of the present work, mechanistic insights are restricted; complementary cellular and animal experiments will be necessary to elucidate the underlying biological pathways.

## 5 Conclusions

Our research substantiates that childhood asthma is a multifaceted disease characterized by imbalances and abnormal activation in both innate and adaptive immunity. Single-cell RNA profiling of PBMCs from asthma patients has uncovered a variety of expression patterns among HSPCs, along with aberrant cell developmental trajectories and diminished cell-cell interaction signaling. Although the heterogeneity among asthma cases is prominent, its pathological characteristics converge on the abnormal lymphoid lineage development, the excessive expression of RETN, S100 and ANXA related genes. SQSTM1, HSPA5, and A2M which directly interact with RETN could serve as the potential therapeutic targets in childhood asthma.

## Data Availability

The datasets presented in this study can be found in online repositories. The names of the repository/repositories and accession number(s) can be found below: https://ngdc.cncb.ac.cn/, omix: accession no. OMIX007142. The data presented in the study are deposited in China National Center for Bioinformation repository (https://ngdc.cncb.ac.cn/), accession number OMIX007142.
