# Supplementary material for: Single-cell RNA sequencing unraveled immune-related expression heterogeneity and lymphoid cell development dysregulation in childhood asthma
Source: Front Immunol. 2026 Jan 2;16:1606650. doi: 10.3389/fimmu.2025.1606650 (PMC12807962; doi:10.3389/fimmu.2025.1606650)
Supplement: Supplementary file 1 [file DataSheet1.docx]

**Supplementary figure legends:**

**Supplementary Figure 1.** Integrated single-cell profiling of PBMCs for four sample groups. **(A)** Principal component analysis of seven samples based on average cell expression values (pseudo-bulk RNA-Seq clustering). **(B)** The detailed classification and proportion of T cells for all seven samples in our study. **(C)** The predicted cell types for the integrated single-cell profiling of PBMCs for four sample groups. **(D)** More detailed predicted cell types for the integrated single-cell profiling of PBMCs for four sample groups. **(E)** Expression of canonical gene markers for each cell type in four sample groups based on integration analysis for (C) is presented. **(F)** Expression of canonical gene markers for each cell type in four sample groups based on integration analysis for (D) is presented. Note: Each asthma patient is a single group while 4 healthy controls are pooled together as the control group.

**Supplementary Figure 2.** Pseudo-time analysis of all cells in four sample groups. **(A)** The proportion of major cell types in each state for healthy controls. **(B)** The proportion of major cell type in each state for Asthma 1. **(C)** The proportion of major cell type in each state for Asthma 2. **(D)** The proportion of major cell type in each state for Asthma 3.

**Supplementary Figure 3.** Pseudo-time analysis of the T cell lineage in four sample groups. **(A)** The differentiation trajectory of T cell lineage in healthy controls by state. **(B)** The differentiation trajectory of T cell lineage in Asthma 1 by state. **(C)** The differentiation trajectory of T cell lineage in Asthma 2 by state. **(D)** The differentiation trajectory of T cell lineage in Asthma 3 by state.

**Supplementary Figure 4.** Pseudo-time analysis of the B cell lineage in four sample groups. **(A)** The differentiation trajectory of the B cell lineage in healthy controls by state. **(B)** The differentiation trajectory of the B cell lineage in Asthma 1 by state. **(C)** The differentiation trajectory of the B cell lineage in Asthma 2 by state. **(D)** The differentiation trajectory of the B cell lineage in Asthma 3 by state.

**Supplementary Figure 5.** Pseudo-time analysis of the monocyte lineage in four sample groups. **(A)** The differentiation trajectory of the monocyte lineage in healthy controls by state. **(B)** The differentiation trajectory of the monocyte lineage in Asthma 1 by state. **(C)** The differentiation trajectory of the monocyte lineage in Asthma 2 by state. **(D)** The differentiation trajectory of the monocyte lineage in Asthma 3 by state.

**Supplementary Figure 6.** The number of incoming and outgoing interactions for each cell type in four sample groups. **(A)** The number of incoming and outgoing interactions in health controls. **(B)** The number of incoming and outgoing interactions in Asthma 1. **(C)** The number of incoming and outgoing interactions in Asthma 2. **(D)** The number of incoming and outgoing interactions in Asthma 3.

**Supplementary Figure 7.** The number of incoming and outgoing interaction strength for each cell type in four sample groups. **(A)** The number of incoming and outgoing interaction strength in health controls. **(B)** The number of incoming and outgoing interaction strength in Asthma 1. **(C)** The number of incoming and outgoing interaction strength in Asthma 2. **(D)** The number of incoming and outgoing interaction strength in Asthma 3.

**Supplementary Figure 8.** The signaling changes of DCs in three asthma patients (health controls as the background). **(A)** The signaling changes of DCs in Asthma 1. **(B)** The signaling changes of DCs in Asthma 2. **(C)** The signaling changes of DCs in Asthma 3.

**Supplementary Figure 9.** The violin plot of universally upregulated genes in asthma patients. **(A)** The violin plot of S100A9, S100A8, S100A12 and RETN gene expression levels across different cell types for four sample groups. **(B)** The violin plot of ANXA1, ANXA2 and ANXA2 gene expression levels across different cell types for four sample groups.

**Supplementary Figure 10.** The upregulated signaling from DCs to the other cell types in asthma patients. **(A)** The upregulated signaling from DCs to the other cell types in Asthma 1. **(B)** The upregulated signaling from DCs to the other cell types in Asthma 2. **(C)** The upregulated signaling from DCs to the other cell types in Asthma 3.

**Supplementary Figure 11.** The downregulated signaling from DCs to the other cell types in asthma patients. **(A)** The downregulated signaling from DCs to the other cell types in Asthma 1. **(B)** The downregulated signaling from DCs to the other cell types in Asthma 2. **(C)** The downregulated signaling from DCs to the other cell types in Asthma 3.

**Supplementary Figure 12.** The expression of RESISTIN and ANNEXIN signaling pathway in asthma patients and healthy controls. **(A)** The dot plot of RESISTIN signaling pathway in three asthma patients and healthy controls. **(B)** The dot plot of ANNEXIN signaling pathway in three asthma patients and healthy controls.
