# Supplementary material for: Single-cell RNA sequencing unraveled immune-related expression heterogeneity and lymphoid cell development dysregulation in childhood asthma
Source: Front Immunol. 2026 Jan 2;16:1606650. doi: 10.3389/fimmu.2025.1606650 (PMC12807962; doi:10.3389/fimmu.2025.1606650)
Supplement: Supplementary file 2 [file Table1.docx]

**Supplementary Table 1.** Clinical features of recruited donors.

|  | Asthma 1 | Asthma 2 | Asthma 3 | Control 1 | Control 2 | Control 3 | Control 4 |
| --- | --- | --- | --- | --- | --- | --- | --- |
| Age (Years) | 8.9 | 4 | 6 | 1.7 | 3 | 5 | 9 |
| Sex(Male/Female) | M | F | M | M | F | F | M |
| Serum total IgE (IU/ml) | 202 | 614 | 1330 | / | / | / | / |
| House dust mite sIgE (IU/ml) | 100 | 43.74 | 100 | / | / | / | / |
| FeNO (ppb) | / | 9 | 5 | / | / | / | / |
| VC max % predicted | 73.3 | 84 | 90.0 | / | / | / | / |
| FVC % predicted | 75.2 | 90.4 | 93.7 | / | / | / | / |
| FEV1 % predicted | 50.9 | 91.6 | 70.2 | / | / | / | / |
| FEV1/FVC % predicted | 66.8 | 102.9 | 74.0 | / | / | / | / |
| WBC (*10^9/L) | 7.19 | 11.57 | 9.60 | 6.13 | 7.07 | 5.88 | 8.72 |
| Neutrophil count (*10^9/L) | 3.93 | 4.93 | 5.00 | 1.48 | 2.29 | 1.75 | 4.07 |
| Eosinophil count (*10^9/L) | 0.35 | 0.38 | 0.03 | 0.26 | 0.25 | 0.22 | 0.17 |
| Lymphocyte count (*10^9/L) | 2.47 | 5.59 | 3.51 | 3.87 | 2.29 | 3.54 | 3.81 |
| Monocyte count (*10^9/L) | 0.40 | 0.59 | 1.05 | 0.5 | 0.59 | 0.34 | 0.63 |
| Basophil count (*10^9/L) | 0.03 | 0.08 | 0.01 | 0.02 | 0.06 | 0.03 | 0.04 |
