# Supplementary material for: Single-cell RNA sequencing unraveled immune-related expression heterogeneity and lymphoid cell development dysregulation in childhood asthma
Source: Front Immunol. 2026 Jan 2;16:1606650. doi: 10.3389/fimmu.2025.1606650 (PMC12807962; doi:10.3389/fimmu.2025.1606650)
Supplement: Supplementary file 3 [file Table2.docx]

**Supplementary Table 2.** Lung Function and Allergen Testing in Asthma Patients

| Donors | Sex(Male/Female) | Age (Years) | Serum total IgE (IU/ml) | House dust mite sIgE (IU/ml) | Therapy | FeNO(ppb) | Vc max % predicted | FVC % predicted | FEV1 % predicted | FEV1/FVC % predicted | PEF % predicted |
| --- | --- | --- | --- | --- | --- | --- | --- | --- | --- | --- | --- |
| 1 | F | 13.3 | 493 | 8.42 | ICS+LABA | 42 | 116.6 | 117.2 | 103.9 | 89.6 | 88.2 |
| 2 | M | 13.3 | 748 | 8.42 | ICS+LABA | 36 | 111.7 | 111.2 | 119.3 | 106.2 | 99 |
| 3 | M | 7.2 | 339 | 5.22 | ICS+LABA | 9 | 78.4 | 80.3 | 87.2 | 107.2 | 95 |
| 4 | M | 13.9 | 113 | 4.34 | ICS+LABA | 20 | 100 | 95.4 | 101.2 | 105 | 98.6 |
| 5 | M | 8.3 | 899 | 32.77 | ICS+LABA | 33 | 92.8 | 96.6 | 95.4 | 97.4 | 71.9 |
| 6 | F | 9.9 | 372 | 14.22 | ICS+LABA | 33 | 83.6 | 85.6 | 91.9 | 108.5 | 81.9 |
| 7 | F | 10.9 | 37.7 | 0.52 | ICS+LABA | 24 | 84.8 | 87.5 | 79.1 | 91.5 | 68.7 |
| 8 | M | 7.9 | 828 | 43.76 | ICS | 26 | 92.9 | 93.2 | 89 | 94.3 | 84.4 |
| 9 | M | 7.7 | 2240 | 35.91 | / | 6 | 94.2 | 98.4 | 100 | 103.7 | 64 |
| 10 | F | 8.7 | 431 | 6.46 | ICS+LABA | 17 | 95.7 | 100 | 81.8 | 82.9 | 79.7 |
| 11 | M | 10.1 | / | / | ICS+LABA | 24 | 93.2 | 100.1 | 95.4 | 95.7 | 95.7 |
| 12 | M | 8.7 | 683 | 5.00 | / | 17 | 91.7 | 92.8 | 85.6 | 91.2 | 68.4 |
| 13 | F | 8.1 | 261 | 0.71 | ICS+LABA | 12 | 73.4 | 76.7 | 64.4 | 85 | 61.8 |
| 14 | M | 12.4 | 427 | 22.73 | ICS+LABA | 21 | 79 | 79.3 | 86.2 | 107.5 | 72.7 |
