# Supplementary material for: Single-cell RNA sequencing unraveled immune-related expression heterogeneity and lymphoid cell development dysregulation in childhood asthma
Source: Front Immunol. 2026 Jan 2;16:1606650. doi: 10.3389/fimmu.2025.1606650 (PMC12807962; doi:10.3389/fimmu.2025.1606650)
Supplement: Supplementary file 4 [file Table3.docx]

**Supplementary Table 3.** GO results of 145 upregulated genes in CD4 T cells of Asthma 1 paitent

| Category | Term | Count | % | *P-*Value | Genes | List Total | Pop Hits | Pop Total | Fold Enrichment | Bonferroni | Benjamini | FDR |
| --- | --- | --- | --- | --- | --- | --- | --- | --- | --- | --- | --- | --- |
| GOTERM_BP_DIRECT | GO:0006955~immune response | 45 | 31.69 | 1.41E-35 | LST1, IGHV4-34, IGLV3-1, IGHV4-39, CTSW, CST7, CTSS, JCHAIN, SPN, CST3, IGLV3-9, IGLV1-40, IGLV6-57, IGKC, IGLV2-14, IGLV3-21, CCL5, FTH1, IGKV3-15, IGLV2-18, CTSH, IGHA1, IGKV3-11, CCR4, SLAMF1, HLA-DPA1, IL32, CD74, HLA-DRB5, IL4R, GZMA, IGKV1-5, TNFRSF1B, IGLV1-51, CD8B, CD8A, IGLV2-23, HLA-DPB1, IGKV4-1, HLA-DRA, IGKV3-20, HLA-DRB1, IGKV2-30, IGLV3-19, HLA-DQB1 | 134 | 537 | 19478 | 1.22E+01 | 1.53E-32 | 1.53E-32 | 1.50E-32 |
| GOTERM_CC_DIRECT | GO:0005576~extracellular region | 67 | 47.18 | 2.58E-27 | FCN1, APP, IGHV4-34, PRF1, IGHV4-39, CTSW, MTRNR2L8, HMGB1, CTSS, JCHAIN, LGALS3, LGALS1, GNLY, FTH1, CTSH, SRGN, IGHV5-51, IL4R, ANXA2, TNFRSF1B, DNAJC3, VCAN, CD8B, CD8A, IGKV4-1, S100A4, TLN1, S100A9, PPIA, S100A8, VCL, IGKV2-30, IGLV3-19, FTL, GRN, GSTP1, STXBP2, TSHZ2, FGL2, IGLV3-1, AOAH, HSP90B1, TTN, CST3, IGLV3-9, IGLV1-40, IGKC, IGLV6-57, IGLV2-14, IGLV3-21, CCL5, PSAP, IGKV3-15, IGLV2-18, IGHA1, IGKV3-11, S100A10, CD74, MTRNR2L12, IGKV1-5, LYZ, NELL2, IGLV1-51, IGLV2-23, MNDA, CALR, IGKV3-20 | 139 | 2313 | 20795 | 4.33E+00 | 8.15E-25 | 8.15E-25 | 7.27E-25 |
| GOTERM_BP_DIRECT | GO:0002250~adaptive immune response | 33 | 23.24 | 1.47E-22 | IGHV4-34, IGLV3-1, IGHV4-39, CTSS, JCHAIN, IGLV3-9, IGLV1-40, IGLV6-57, IGKC, IGLV2-14, IGLV3-21, IGKV3-15, IGLV2-18, CTSH, IGHA1, IGKV3-11, SLAMF1, HLA-DPA1, IGHV5-51, HLA-DRB5, IGKV1-5, IGLV1-51, CD8B, CD8A, IGLV2-23, HLA-DPB1, IGKV4-1, HLA-DRA, IGKV3-20, HLA-DRB1, IGKV2-30, IGLV3-19, HLA-DQB1 | 134 | 492 | 19478 | 9.75E+00 | 1.59E-19 | 7.97E-20 | 7.81E-20 |
| GOTERM_MF_DIRECT | GO:0003823~antigen binding | 22 | 15.49 | 2.24E-22 | FCN1, IGHV5-51, IGKV1-5, IGHV4-34, IGLV3-1, IGHV4-39, JCHAIN, IGLV1-51, IGLV1-40, IGLV6-57, IGKC, IGLV2-14, IGLV3-21, IGLV2-23, IGKV4-1, IGKV3-15, IGHA1, IGKV3-11, IGKV3-20, IGKV2-30, IGLV3-19, SLAMF1 | 135 | 140 | 19253 | 2.24E+01 | 8.06E-20 | 8.06E-20 | 7.75E-20 |
| UP_KW_BIOLOGICAL_PROCESS | KW-0391~Immunity | 43 | 30.28 | 2.32E-22 | FCN1, LST1, IGHV4-34, IGLV3-1, IGHV4-39, HMGB1, LGALS3, IGLV3-9, IGLV1-40, SEC14L1, IGLV6-57, IGKC, IGLV2-14, IGLV3-21, IGKV3-15, IGLV2-18, IGHA1, IGKV3-11, SLAMF1, HLA-DPA1, KLRG1, CD74, IGHV5-51, HLA-DRB5, FCER1G, IL4R, IGKV1-5, TYROBP, IGLV1-51, CD8B, JAML, CD8A, IGLV2-23, HLA-DPB1, IGKV4-1, HLA-DRA, S100A9, S100A8, IGKV3-20, HLA-DRB1, IGKV2-30, IGLV3-19, HLA-DQB1 | 89 | 980 | 11523 | 5.68E+00 | 1.07E-20 | 1.07E-20 | 1.02E-20 |
| GOTERM_CC_DIRECT | GO:0070062~extracellular exosome | 60 | 42.25 | 3.56E-22 | ITGB1, APP, CLIC3, CD82, RPLP0, JCHAIN, ACTG1, SPN, LGALS3, LGALS1, FTH1, CTSH, CLIC1, TPM4, ANXA2, SSR4, APLP2, STX7, SND1, DNAJC3, CANX, S100A4, TLN1, PFN1, PPIB, S100A9, PPIA, S100A8, VCL, IGKV2-30, IGLV3-19, FTL, GRN, AHNAK, GSTP1, STXBP2, FGL2, HSP90B1, TTN, CST3, LMAN1, IGKC, IGLV2-14, IGLV3-21, PSAP, IGHA1, IGKV3-11, S100A10, SLAMF1, CD74, HLA-DRB5, HSPA5, IGKV1-5, LYZ, IGLV1-51, HLA-DRA, MNDA, CALR, HLA-DRB1, IGKV3-20 | 139 | 2242 | 20795 | 4.00E+00 | 1.13E-19 | 5.63E-20 | 5.02E-20 |
| UP_KW_BIOLOGICAL_PROCESS | KW-1064~Adaptive immunity | 32 | 22.54 | 3.98E-20 | IGHV4-34, IGLV3-1, IGHV4-39, HMGB1, IGLV3-9, IGLV1-40, IGLV6-57, IGKC, IGLV2-14, IGLV3-21, IGKV3-15, IGLV2-18, IGHA1, IGKV3-11, SLAMF1, HLA-DPA1, CD74, IGHV5-51, HLA-DRB5, IGKV1-5, IGLV1-51, CD8B, CD8A, IGLV2-23, HLA-DPB1, IGKV4-1, HLA-DRA, IGKV3-20, HLA-DRB1, IGKV2-30, IGLV3-19, HLA-DQB1 | 89 | 521 | 11523 | 7.95E+00 | 1.83E-18 | 9.15E-19 | 8.75E-19 |
| UP_KW_CELLULAR_COMPONENT | KW-0964~Secreted | 60 | 42.25 | 5.73E-20 | FCN1, APP, CLIC3, IGHV4-34, PRF1, IGHV4-39, MTRNR2L8, HMGB1, CTSS, JCHAIN, LGALS3, LGALS1, GNLY, SRGN, IGHV5-51, IL4R, ANXA2, TNFRSF1B, VCAN, CD8B, CD8A, IGKV4-1, S100A4, S100A9, PPIA, S100A8, IGKV2-30, IGLV3-19, GRN, TSHZ2, FGL2, IGLV3-1, AOAH, CST7, CST3, IGLV3-9, IGLV1-40, IGKC, IGLV6-57, IGLV2-14, IGLV3-21, CCL5, PSAP, IGKV3-15, IGLV2-18, IGHA1, IGKV3-11, SLAMF1, IL32, CD74, GZMA, MTRNR2L12, IGKV1-5, LYZ, NELL2, GZMK, IGLV1-51, IGLV2-23, CALR, IGKV3-20 | 136 | 2217 | 18049 | 3.59E+00 | 1.66E-18 | 1.66E-18 | 1.20E-18 |
| UP_SEQ_FEATURE | REGION:Complementarity-determining-3 | 19 | 13.38 | 1.33E-18 | IGHV5-51, IGKV1-5, IGHV4-34, IGLV3-1, IGHV4-39, IGLV3-9, IGLV1-51, IGLV1-40, IGLV6-57, IGLV2-14, IGLV3-21, IGLV2-23, IGKV4-1, IGKV3-15, IGLV2-18, IGKV3-11, IGKV3-20, IGKV2-30, IGLV3-19 | 136 | 139 | 20675 | 2.08E+01 | 1.58E-15 | 3.90E-16 | 3.85E-16 |
| UP_SEQ_FEATURE | REGION:Framework-1 | 19 | 13.38 | 1.74E-18 | IGHV5-51, IGKV1-5, IGHV4-34, IGLV3-1, IGHV4-39, IGLV3-9, IGLV1-51, IGLV1-40, IGLV6-57, IGLV2-14, IGLV3-21, IGLV2-23, IGKV4-1, IGKV3-15, IGLV2-18, IGKV3-11, IGKV3-20, IGKV2-30, IGLV3-19 | 136 | 141 | 20675 | 2.05E+01 | 2.05E-15 | 3.90E-16 | 3.85E-16 |
| UP_SEQ_FEATURE | REGION:Complementarity-determining-2 | 19 | 13.38 | 1.98E-18 | IGHV5-51, IGKV1-5, IGHV4-34, IGLV3-1, IGHV4-39, IGLV3-9, IGLV1-51, IGLV1-40, IGLV6-57, IGLV2-14, IGLV3-21, IGLV2-23, IGKV4-1, IGKV3-15, IGLV2-18, IGKV3-11, IGKV3-20, IGKV2-30, IGLV3-19 | 136 | 142 | 20675 | 2.03E+01 | 2.34E-15 | 3.90E-16 | 3.85E-16 |
| UP_SEQ_FEATURE | REGION:Framework-3 | 19 | 13.38 | 1.98E-18 | IGHV5-51, IGKV1-5, IGHV4-34, IGLV3-1, IGHV4-39, IGLV3-9, IGLV1-51, IGLV1-40, IGLV6-57, IGLV2-14, IGLV3-21, IGLV2-23, IGKV4-1, IGKV3-15, IGLV2-18, IGKV3-11, IGKV3-20, IGKV2-30, IGLV3-19 | 136 | 142 | 20675 | 2.03E+01 | 2.34E-15 | 3.90E-16 | 3.85E-16 |
| UP_SEQ_FEATURE | REGION:Framework-2 | 19 | 13.38 | 1.98E-18 | IGHV5-51, IGKV1-5, IGHV4-34, IGLV3-1, IGHV4-39, IGLV3-9, IGLV1-51, IGLV1-40, IGLV6-57, IGLV2-14, IGLV3-21, IGLV2-23, IGKV4-1, IGKV3-15, IGLV2-18, IGKV3-11, IGKV3-20, IGKV2-30, IGLV3-19 | 136 | 142 | 20675 | 2.03E+01 | 2.34E-15 | 3.90E-16 | 3.85E-16 |
| UP_SEQ_FEATURE | REGION:Complementarity-determining-1 | 19 | 13.38 | 1.98E-18 | IGHV5-51, IGKV1-5, IGHV4-34, IGLV3-1, IGHV4-39, IGLV3-9, IGLV1-51, IGLV1-40, IGLV6-57, IGLV2-14, IGLV3-21, IGLV2-23, IGKV4-1, IGKV3-15, IGLV2-18, IGKV3-11, IGKV3-20, IGKV2-30, IGLV3-19 | 136 | 142 | 20675 | 2.03E+01 | 2.34E-15 | 3.90E-16 | 3.85E-16 |
| UP_SEQ_FEATURE | DOMAIN:Ig-like | 32 | 22.54 | 4.64E-18 | IGHV4-34, IGLV3-1, IGHV4-39, TTN, IGLV3-9, IGLV1-40, IGLV6-57, IGKC, IGLV2-14, IGLV3-21, IGKV3-15, IGLV2-18, IGKV3-11, SLAMF1, HLA-DPA1, IGHV5-51, HLA-DRB5, IGKV1-5, VCAN, IGLV1-51, CD8B, JAML, CD8A, IGLV2-23, HLA-DPB1, IGKV4-1, HLA-DRA, IGKV3-20, HLA-DRB1, IGKV2-30, IGLV3-19, HLA-DQB1 | 136 | 673 | 20675 | 7.23E+00 | 5.48E-15 | 7.84E-16 | 7.75E-16 |
| UP_KW_CELLULAR_COMPONENT | KW-1280~Immunoglobulin | 21 | 14.79 | 1.56E-17 | IGHV5-51, IGKV1-5, IGHV4-34, IGLV3-1, IGHV4-39, IGLV3-9, IGLV1-51, IGLV1-40, IGLV6-57, IGKC, IGLV2-14, IGLV3-21, IGLV2-23, IGKV4-1, IGKV3-15, IGLV2-18, IGHA1, IGKV3-11, IGKV3-20, IGKV2-30, IGLV3-19 | 136 | 192 | 18049 | 1.45E+01 | 4.53E-16 | 2.27E-16 | 1.64E-16 |
| INTERPRO | IPR007110:Ig-like_dom | 33 | 23.24 | 2.96E-17 | IGHV4-34, IGLV3-1, IGHV4-39, TTN, IGLV3-9, IGLV1-40, IGLV6-57, IGKC, IGLV2-14, IGLV3-21, IGKV3-15, IGLV2-18, IGHA1, IGKV3-11, SLAMF1, HLA-DPA1, IGHV5-51, HLA-DRB5, IGKV1-5, VCAN, IGLV1-51, CD8B, JAML, CD8A, IGLV2-23, HLA-DPB1, IGKV4-1, HLA-DRA, IGKV3-20, HLA-DRB1, IGKV2-30, IGLV3-19, HLA-DQB1 | 138 | 768 | 20808 | 6.48E+00 | 1.42E-14 | 1.02E-14 | 9.79E-15 |
| INTERPRO | IPR050150:IgV_Light_Chain | 16 | 11.27 | 4.24E-17 | IGKV1-5, IGLV3-1, IGLV3-9, IGLV1-51, IGLV1-40, IGLV6-57, IGLV2-14, IGLV3-21, IGLV2-23, IGKV4-1, IGKV3-15, IGLV2-18, IGKV3-11, IGKV3-20, IGKV2-30, IGLV3-19 | 138 | 93 | 20808 | 2.59E+01 | 2.03E-14 | 1.02E-14 | 9.79E-15 |
| UP_KW_DOMAIN | KW-0732~Signal | 80 | 56.34 | 3.73E-16 | FCN1, ITGB1, APP, CD82, MT-CO1, LST1, IGHV4-34, PRF1, TXNDC11, IGHV4-39, CTSW, CLINT1, CTSS, JCHAIN, SPN, GNLY, CTSH, HLA-DPA1, SRGN, NUP214, IGHV5-51, FCER1G, IL4R, SSR4, APLP2, TNFRSF1B, ADAM19, ERN1, DNAJC3, VCAN, DNAJC1, TYROBP, CD8B, JAML, CD8A, CANX, IGKV4-1, PPIB, IGKV2-30, IGLV3-19, HLA-DQB1, GRN, CLDND1, RPN1, FGL2, IGLV3-1, AOAH, CST7, MESD, HSP90B1, CST3, LMAN1, IGLV3-9, IGLV1-40, IGLV6-57, IGLV2-14, IGLV3-21, CCL5, PSAP, IGKV3-15, IGLV2-18, IGKV3-11, SLAMF1, IL32, HLA-DRB5, HSPA5, GZMA, IGKV1-5, LYZ, NELL2, GZMK, PTPRE, IGLV1-51, GOLGB1, IGLV2-23, HLA-DPB1, HLA-DRA, CALR, HLA-DRB1, IGKV3-20 | 120 | 4415 | 14625 | 2.21E+00 | 6.66E-15 | 7.46E-15 | 7.46E-15 |
| GOTERM_CC_DIRECT | GO:0019814~immunoglobulin complex | 19 | 13.38 | 7.42E-16 | IGHV5-51, IGKV1-5, IGHV4-34, IGLV3-1, IGHV4-39, IGLV3-9, IGLV1-51, IGLV1-40, IGLV6-57, IGLV2-14, IGLV3-21, IGLV2-23, IGKV4-1, IGKV3-15, IGLV2-18, IGKV3-11, IGKV3-20, IGKV2-30, IGLV3-19 | 139 | 194 | 20795 | 1.47E+01 | 2.46E-13 | 7.82E-14 | 6.98E-14 |
| INTERPRO | IPR036179:Ig-like_dom_sf | 33 | 23.24 | 1.30E-15 | IGHV4-34, IGLV3-1, IGHV4-39, TTN, IGLV3-9, IGLV1-40, IGLV6-57, IGKC, IGLV2-14, IGLV3-21, IGKV3-15, IGLV2-18, IGHA1, IGKV3-11, SLAMF1, HLA-DPA1, IGHV5-51, HLA-DRB5, IGKV1-5, VCAN, IGLV1-51, CD8B, JAML, CD8A, IGLV2-23, HLA-DPB1, IGKV4-1, HLA-DRA, IGKV3-20, HLA-DRB1, IGKV2-30, IGLV3-19, HLA-DQB1 | 138 | 872 | 20808 | 5.71E+00 | 6.38E-13 | 2.08E-13 | 2.00E-13 |
| UP_KW_PTM | KW-1015~Disulfide bond | 77 | 54.23 | 1.37E-14 | FCN1, ITGB1, APP, CLIC3, IGHV4-34, PRF1, TXNDC11, IGHV4-39, CTSW, HMGB1, CTSS, JCHAIN, LGALS3, GNLY, CTSH, CLIC1, CCR4, HLA-DPA1, SRGN, IGHV5-51, FCER1G, IL4R, SSR4, APLP2, TNFRSF1B, ADAM19, ERN1, DNAJC3, VCAN, TYROBP, CD8B, JAML, CD8A, CANX, IGKV4-1, IGKV2-30, IGLV3-19, HLA-DQB1, GRN, FGL2, IGLV3-1, AOAH, CST7, HSP90B1, TTN, CST3, LMAN1, IGLV3-9, IGLV1-40, IGKC, IGLV6-57, CUX1, IGLV2-14, IGLV3-21, CCL5, PSAP, IGKV3-15, IGLV2-18, IGHA1, IGKV3-11, SLAMF1, KLRG1, CD74, HLA-DRB5, GZMA, IGKV1-5, LYZ, NELL2, GZMK, IGLV1-51, GOLGB1, IGLV2-23, HLA-DPB1, HLA-DRA, CALR, HLA-DRB1, IGKV3-20 | 127 | 3956 | 14316 | 2.19E+00 | 2.59E-13 | 2.74E-13 | 2.19E-13 |
| INTERPRO | IPR013106:Ig_V-set | 24 | 16.90 | 1.11E-13 | IGHV5-51, IGKV1-5, IGHV4-34, IGLV3-1, IGHV4-39, TTN, IGLV3-9, VCAN, IGLV1-51, IGLV1-40, JAML, IGLV6-57, CD8B, IGLV2-14, IGLV3-21, CD8A, IGLV2-23, IGKV4-1, IGKV3-15, IGLV2-18, IGKV3-11, IGKV3-20, IGKV2-30, IGLV3-19 | 138 | 485 | 20808 | 7.46E+00 | 5.30E-11 | 1.32E-11 | 1.28E-11 |
| INTERPRO | IPR013783:Ig-like_fold | 34 | 23.94 | 2.50E-13 | IGHV4-34, IGLV3-1, IGHV4-39, TTN, IGLV3-9, IGLV1-40, IGLV6-57, IGKC, IGLV2-14, IGLV3-21, IGKV3-15, IGLV2-18, IGHA1, IGKV3-11, SLAMF1, HLA-DPA1, IGHV5-51, HLA-DRB5, IL4R, IGKV1-5, VCAN, IGLV1-51, CD8B, JAML, CD8A, IGLV2-23, HLA-DPB1, IGKV4-1, HLA-DRA, IGKV3-20, HLA-DRB1, IGKV2-30, IGLV3-19, HLA-DQB1 | 138 | 1127 | 20808 | 4.55E+00 | 1.20E-10 | 2.39E-11 | 2.31E-11 |
| SMART | SM00406:IGv | 22 | 15.49 | 6.12E-13 | IGHV5-51, IGKV1-5, IGHV4-34, IGLV3-1, IGHV4-39, TTN, IGLV3-9, IGLV1-51, IGLV1-40, IGLV6-57, CD8B, IGLV2-14, IGLV3-21, CD8A, IGLV2-23, IGKV4-1, IGKV3-15, IGLV2-18, IGKV3-11, IGKV3-20, IGKV2-30, IGLV3-19 | 94 | 334 | 10706 | 7.50E+00 | 5.57E-11 | 5.57E-11 | 5.20E-11 |
| KEGG_PATHWAY | hsa04612:Antigen processing and presentation | 13 | 9.15 | 2.66E-12 | CD74, HLA-DRB5, HSPA5, CTSS, CD8B, CD8A, CANX, HLA-DPB1, HLA-DRA, CALR, HLA-DRB1, HLA-DPA1, HLA-DQB1 | 74 | 81 | 8534 | 1.85E+01 | 4.55E-10 | 4.55E-10 | 3.94E-10 |
| GOTERM_CC_DIRECT | GO:0005886~plasma membrane | 76 | 53.52 | 7.92E-12 | FCN1, ITGB1, APP, CLIC3, CD82, LST1, IGHV4-34, PRF1, IGHV4-39, HMGB1, CLINT1, ACTG1, SPN, LGALS3, LGALS1, CLIC1, CCR4, HLA-DPA1, RALGPS2, IGHV5-51, FCER1G, IL4R, ANXA2, APLP2, STX7, TNFRSF1B, ADAM19, MS4A6A, DNAJC1, TYROBP, CD8B, JAML, CD8A, IGKV4-1, TLN1, S100A9, S100A8, VCL, IGKV2-30, IGLV3-19, HLA-DQB1, GRN, AHNAK, STXBP2, IGLV3-1, MESD, CST3, IGLV3-9, IGLV1-40, IGKC, IGLV6-57, IGLV2-14, IGLV3-21, PSAP, IGKV3-15, IGLV2-18, IGHA1, IGKV3-11, S100A10, SLAMF1, KLRG1, CD74, HLA-DRB5, HSPA5, IGKV1-5, NKG7, NELL2, PTPRE, IGLV1-51, IGLV2-23, HLA-DPB1, MDM2, HLA-DRA, HLA-DRB1, MYO1F, IGKV3-20 | 139 | 5597 | 20795 | 2.03E+00 | 2.50E-09 | 6.26E-10 | 5.58E-10 |
| GOTERM_BP_DIRECT | GO:0019882~antigen processing and presentation | 10 | 7.04 | 1.77E-11 | CD74, HLA-DRB5, CD8A, HLA-DPB1, HLA-DRA, CTSH, CTSS, HLA-DRB1, HLA-DPA1, HLA-DQB1 | 134 | 45 | 19478 | 3.23E+01 | 1.92E-08 | 6.40E-09 | 6.27E-09 |
| GOTERM_BP_DIRECT | GO:0019886~antigen processing and presentation of exogenous peptide antigen via MHC class II | 9 | 6.34 | 2.64E-11 | CD74, HLA-DRB5, FCER1G, HLA-DPB1, HLA-DRA, CTSS, HLA-DRB1, HLA-DPA1, HLA-DQB1 | 134 | 31 | 19478 | 4.22E+01 | 2.87E-08 | 7.18E-09 | 7.03E-09 |
| GOTERM_CC_DIRECT | GO:0098553~lumenal side of endoplasmic reticulum membrane | 9 | 6.34 | 6.20E-11 | CD74, HLA-DRB5, CANX, HLA-DPB1, HLA-DRA, CALR, HLA-DRB1, HLA-DPA1, HLA-DQB1 | 139 | 35 | 20795 | 3.85E+01 | 1.96E-08 | 3.92E-09 | 3.50E-09 |
| GOTERM_CC_DIRECT | GO:0072562~blood microparticle | 13 | 9.15 | 3.04E-10 | IGKV1-5, ACTG1, JCHAIN, IGKC, IGLV3-21, IGKV4-1, IGKV3-15, PFN1, IGHA1, IGKV3-11, CLIC1, IGKV3-20, IGKV2-30 | 139 | 148 | 20795 | 1.31E+01 | 9.61E-08 | 1.60E-08 | 1.43E-08 |
| GOTERM_CC_DIRECT | GO:0005615~extracellular space | 38 | 26.76 | 8.36E-10 | FCN1, APP, GRN, GSTP1, FGL2, CTSW, HMGB1, CST7, CTSS, ACTG1, JCHAIN, SPN, CST3, LGALS3, LGALS1, IGKC, GNLY, CCL5, PSAP, CTSH, IGHA1, CLIC1, S100A10, IL32, SRGN, ANXA2, GZMA, LYZ, NELL2, GZMK, VCAN, S100A4, CALR, S100A9, PPIA, S100A8, IGKV3-20, HLA-DRB1 | 139 | 1867 | 20795 | 3.04E+00 | 2.64E-07 | 3.77E-08 | 3.37E-08 |
| UP_KW_DOMAIN | KW-0393~Immunoglobulin domain | 27 | 19.01 | 1.84E-09 | IGHV4-34, IGLV3-1, IGHV4-39, TTN, IGLV3-9, IGLV1-40, IGLV6-57, IGKC, IGLV2-14, IGLV3-21, IGKV3-15, IGLV2-18, IGHA1, IGKV3-11, SLAMF1, IGHV5-51, IGKV1-5, VCAN, IGLV1-51, CD8B, JAML, CD8A, IGLV2-23, IGKV4-1, IGKV3-20, IGKV2-30, IGLV3-19 | 120 | 825 | 14625 | 3.99E+00 | 3.68E-08 | 1.84E-08 | 1.84E-08 |
| INTERPRO | IPR003599:Ig_sub | 20 | 14.08 | 2.11E-09 | IGKV1-5, IGLV3-1, TTN, IGLV3-9, VCAN, IGLV1-51, IGLV1-40, JAML, IGLV6-57, CD8B, IGLV2-14, IGLV3-21, CD8A, IGLV2-23, IGKV4-1, IGKV3-15, IGLV2-18, IGKV3-11, IGKV3-20, IGLV3-19 | 138 | 531 | 20808 | 5.68E+00 | 1.01E-06 | 1.69E-07 | 1.63E-07 |
| GOTERM_CC_DIRECT | GO:0044194~cytolytic granule | 6 | 4.23 | 5.35E-09 | SRGN, GNLY, STXBP2, PRF1, NKG7, CALR | 139 | 11 | 20795 | 8.16E+01 | 1.69E-06 | 2.11E-07 | 1.89E-07 |
| GOTERM_CC_DIRECT | GO:0062023~collagen-containing extracellular matrix | 17 | 11.97 | 6.46E-09 | FCN1, ANXA2, FGL2, CTSS, HSP90B1, ADAM19, LGALS3, LMAN1, VCAN, LGALS1, PSAP, CTSH, S100A4, CALR, S100A9, S100A8, S100A10 | 139 | 387 | 20795 | 6.57E+00 | 2.04E-06 | 2.27E-07 | 2.02E-07 |
| KEGG_PATHWAY | hsa04145:Phagosome | 13 | 9.15 | 7.96E-09 | ITGB1, HLA-DRB5, STX7, CTSS, ACTG1, SEC61A1, CANX, HLA-DPB1, HLA-DRA, CALR, HLA-DRB1, HLA-DPA1, HLA-DQB1 | 74 | 159 | 8534 | 9.43E+00 | 1.36E-06 | 6.80E-07 | 5.89E-07 |
| GOTERM_CC_DIRECT | GO:1904813~ficolin-1-rich granule lumen | 11 | 7.75 | 1.19E-08 | FCN1, CST3, GSTP1, FTH1, FGL2, CTSH, MNDA, HMGB1, PPIA, CTSS, VCL | 139 | 126 | 20795 | 1.31E+01 | 3.77E-06 | 3.77E-07 | 3.37E-07 |
| GOTERM_CC_DIRECT | GO:0009986~cell surface | 21 | 14.79 | 1.55E-08 | ITGB1, CD74, APP, CLDND1, FCER1G, HSPA5, ANXA2, HMGB1, SPN, LGALS3, TYROBP, CD8B, HLA-DPB1, HLA-DRA, CALR, TLN1, HLA-DRB1, SLAMF1, HLA-DPA1, S100A10, HLA-DQB1 | 139 | 662 | 20795 | 4.75E+00 | 4.91E-06 | 4.16E-07 | 3.71E-07 |
| GOTERM_CC_DIRECT | GO:0042613~MHC class II protein complex | 7 | 4.93 | 1.58E-08 | CD74, HLA-DRB5, HLA-DPB1, HLA-DRA, HLA-DRB1, HLA-DPA1, HLA-DQB1 | 139 | 26 | 20795 | 4.03E+01 | 4.99E-06 | 4.16E-07 | 3.71E-07 |
| KEGG_PATHWAY | hsa04141:Protein processing in endoplasmic reticulum | 13 | 9.15 | 1.70E-08 | HSPA5, SSR4, RPN1, RRBP1, UBE2J1, HSP90B1, ERN1, DNAJC3, SEC61A1, LMAN1, DNAJC1, CANX, CALR | 74 | 170 | 8534 | 8.82E+00 | 2.90E-06 | 9.67E-07 | 8.37E-07 |
| GOTERM_MF_DIRECT | GO:0023026~MHC class II protein complex binding | 7 | 4.93 | 2.67E-08 | CD74, HLA-DRB5, HLA-DPB1, HLA-DRA, HLA-DRB1, HLA-DPA1, HLA-DQB1 | 135 | 27 | 19253 | 3.70E+01 | 9.60E-06 | 4.80E-06 | 4.61E-06 |
| GOTERM_BP_DIRECT | GO:0002503~peptide antigen assembly with MHC class II protein complex | 6 | 4.23 | 5.66E-08 | HLA-DRB5, HLA-DPB1, HLA-DRA, HLA-DRB1, HLA-DPA1, HLA-DQB1 | 134 | 16 | 19478 | 5.45E+01 | 6.15E-05 | 1.23E-05 | 1.21E-05 |
| GOTERM_CC_DIRECT | GO:0012507~ER to Golgi transport vesicle membrane | 8 | 5.63 | 1.10E-07 | CD74, LMAN1, HLA-DRB5, HLA-DPB1, HLA-DRA, HLA-DRB1, HLA-DPA1, HLA-DQB1 | 139 | 58 | 20795 | 2.06E+01 | 3.48E-05 | 2.68E-06 | 2.39E-06 |
| SMART | SM00409:IG | 20 | 14.08 | 1.11E-07 | IGKV1-5, IGLV3-1, TTN, IGLV3-9, VCAN, IGLV1-51, IGLV1-40, JAML, IGLV6-57, CD8B, IGLV2-14, IGLV3-21, CD8A, IGLV2-23, IGKV4-1, IGKV3-15, IGLV2-18, IGKV3-11, IGKV3-20, IGLV3-19 | 94 | 531 | 10706 | 4.29E+00 | 1.01E-05 | 5.03E-06 | 4.70E-06 |
| UP_SEQ_FEATURE | DOMAIN:Immunoglobulin C1-set | 6 | 4.23 | 1.19E-07 | HLA-DRB5, HLA-DPB1, HLA-DRA, HLA-DRB1, HLA-DPA1, HLA-DQB1 | 136 | 19 | 20675 | 4.80E+01 | 1.41E-04 | 1.76E-05 | 1.74E-05 |
| UP_KW_CELLULAR_COMPONENT | KW-0458~Lysosome | 17 | 11.97 | 1.70E-07 | SRGN, CD74, GRN, HLA-DRB5, PRF1, NKG7, CTSS, FTH1, PSAP, HLA-DPB1, CTSH, HLA-DRA, CALR, HLA-DRB1, HLA-DPA1, HLA-DQB1, FTL | 136 | 436 | 18049 | 5.17E+00 | 4.93E-06 | 1.64E-06 | 1.19E-06 |
| GOTERM_BP_DIRECT | GO:0002504~antigen processing and presentation of peptide or polysaccharide antigen via MHC class II | 6 | 4.23 | 1.97E-07 | HLA-DRB5, HLA-DPB1, HLA-DRA, HLA-DRB1, HLA-DPA1, HLA-DQB1 | 134 | 20 | 19478 | 4.36E+01 | 2.14E-04 | 3.56E-05 | 3.49E-05 |
| KEGG_PATHWAY | hsa05310:Asthma | 7 | 4.93 | 2.42E-07 | HLA-DRB5, FCER1G, HLA-DPB1, HLA-DRA, HLA-DRB1, HLA-DPA1, HLA-DQB1 | 74 | 32 | 8534 | 2.52E+01 | 4.13E-05 | 1.03E-05 | 8.94E-06 |
| UP_KW_CELLULAR_COMPONENT | KW-0491~MHC II | 6 | 4.23 | 3.08E-07 | HLA-DRB5, HLA-DPB1, HLA-DRA, HLA-DRB1, HLA-DPA1, HLA-DQB1 | 136 | 20 | 18049 | 3.98E+01 | 8.93E-06 | 2.23E-06 | 1.62E-06 |
| INTERPRO | IPR003006:Ig/MHC_CS | 8 | 5.63 | 3.53E-07 | HLA-DRB5, IGKC, HLA-DPB1, HLA-DRA, IGHA1, HLA-DRB1, HLA-DPA1, HLA-DQB1 | 138 | 69 | 20808 | 1.75E+01 | 1.69E-04 | 2.42E-05 | 2.33E-05 |
| GOTERM_BP_DIRECT | GO:0140507~granzyme-mediated programmed cell death signaling pathway | 5 | 3.52 | 4.23E-07 | SRGN, GZMK, GZMA, PRF1, NKG7 | 134 | 10 | 19478 | 7.27E+01 | 4.59E-04 | 6.56E-05 | 6.43E-05 |
| INTERPRO | IPR014745:MHC_II_a/b_N | 6 | 4.23 | 4.42E-07 | HLA-DRB5, HLA-DPB1, HLA-DRA, HLA-DRB1, HLA-DPA1, HLA-DQB1 | 138 | 24 | 20808 | 3.77E+01 | 2.12E-04 | 2.65E-05 | 2.55E-05 |
| GOTERM_CC_DIRECT | GO:0016020~membrane | 64 | 45.07 | 4.94E-07 | ITGB1, APP, CLIC3, CD82, MT-CO1, LST1, RPLP0, PRF1, CTSW, CLINT1, ACTG1, SPN, SEC61A1, LGALS3, FTH1, CLIC1, HLA-DPA1, TMPO, IL4R, TPM4, ANXA2, APLP2, TNFRSF1B, SND1, ADAM19, DNAJC3, MS4A6A, VCAN, DNAJC1, TYROBP, CD8B, JAML, CANX, MT-CO2, PFN1, PPIB, PPIA, HLA-DQB1, FTL, GRN, CLDND1, AHNAK, RPN1, RRBP1, HSP90B1, MTDH, LMAN1, RAB11FIP1, PSAP, S100A10, SLAMF1, KLRG1, IL32, CD74, HLA-DRB5, HSPA5, NKG7, NELL2, PTPRE, GOLGB1, HLA-DPB1, HLA-DRA, CALR, HLA-DRB1 | 139 | 5415 | 20795 | 1.77E+00 | 1.56E-04 | 1.12E-05 | 9.95E-06 |
| UP_KW_CELLULAR_COMPONENT | KW-0256~Endoplasmic reticulum | 30 | 21.13 | 5.84E-07 | APP, RPN1, TXNDC11, RRBP1, CTSW, HMGB1, MESD, UBE2J1, HSP90B1, MTDH, LMAN1, SEC61A1, CLIC1, HLA-DPA1, CD74, HLA-DRB5, SSR4, HSPA5, FOS, ERN1, DNAJC3, SPCS3, DNAJC1, CANX, HLA-DPB1, HLA-DRA, CALR, PPIB, HLA-DRB1, HLA-DQB1 | 136 | 1426 | 18049 | 2.79E+00 | 1.69E-05 | 3.39E-06 | 2.45E-06 |
| GOTERM_BP_DIRECT | GO:0034975~protein folding in endoplasmic reticulum | 5 | 3.52 | 6.61E-07 | DNAJC3, HSPA5, CANX, CALR, HSP90B1 | 134 | 11 | 19478 | 6.61E+01 | 7.18E-04 | 8.98E-05 | 8.79E-05 |
| UP_KW_LIGAND | KW-0106~Calcium | 20 | 14.08 | 7.03E-07 | ITGB1, FCN1, TPM4, ANXA2, MT-CO1, PRF1, AOAH, AIF1, TTN, HSP90B1, NELL2, VCAN, TYROBP, CANX, S100A4, CALR, S100A9, S100A8, HLA-DQB1, FTL | 42 | 990 | 6987 | 3.36E+00 | 1.27E-05 | 1.27E-05 | 1.27E-05 |
| GOTERM_MF_DIRECT | GO:0050786~RAGE receptor binding | 5 | 3.52 | 7.13E-07 | APP, S100A4, HMGB1, S100A9, S100A8 | 135 | 11 | 19253 | 6.48E+01 | 2.57E-04 | 8.55E-05 | 8.22E-05 |
| GOTERM_CC_DIRECT | GO:0030666~endocytic vesicle membrane | 8 | 5.63 | 7.26E-07 | CD74, HLA-DRB5, MDM2, HLA-DPB1, HLA-DRA, HLA-DRB1, HLA-DPA1, HLA-DQB1 | 139 | 76 | 20795 | 1.57E+01 | 2.29E-04 | 1.53E-05 | 1.37E-05 |
| KEGG_PATHWAY | hsa04514:Cell adhesion molecules | 11 | 7.75 | 8.12E-07 | SPN, ITGB1, VCAN, HLA-DRB5, CD8B, CD8A, HLA-DPB1, HLA-DRA, HLA-DRB1, HLA-DPA1, HLA-DQB1 | 74 | 158 | 8534 | 8.03E+00 | 1.39E-04 | 2.37E-05 | 2.05E-05 |
| INTERPRO | IPR003597:Ig_C1-set | 8 | 5.63 | 8.23E-07 | HLA-DRB5, IGKC, HLA-DPB1, HLA-DRA, IGHA1, HLA-DRB1, HLA-DPA1, HLA-DQB1 | 138 | 78 | 20808 | 1.55E+01 | 3.94E-04 | 4.38E-05 | 4.22E-05 |
| KEGG_PATHWAY | hsa05330:Allograft rejection | 7 | 4.93 | 8.30E-07 | HLA-DRB5, HLA-DPB1, PRF1, HLA-DRA, HLA-DRB1, HLA-DPA1, HLA-DQB1 | 74 | 39 | 8534 | 2.07E+01 | 1.42E-04 | 2.37E-05 | 2.05E-05 |
| GOTERM_CC_DIRECT | GO:0030658~transport vesicle membrane | 7 | 4.93 | 8.47E-07 | CD74, HLA-DRB5, HLA-DPB1, HLA-DRA, HLA-DRB1, HLA-DPA1, HLA-DQB1 | 139 | 49 | 20795 | 2.14E+01 | 2.68E-04 | 1.67E-05 | 1.49E-05 |
| INTERPRO | IPR050160:MHC/Immunoglobulin | 6 | 4.23 | 1.44E-06 | HLA-DRB5, HLA-DPB1, HLA-DRA, HLA-DRB1, HLA-DPA1, HLA-DQB1 | 138 | 30 | 20808 | 3.02E+01 | 6.87E-04 | 6.88E-05 | 6.63E-05 |
| GOTERM_MF_DIRECT | GO:0032395~MHC class II receptor activity | 5 | 3.52 | 1.53E-06 | HLA-DPB1, HLA-DRA, HLA-DRB1, HLA-DPA1, HLA-DQB1 | 135 | 13 | 19253 | 5.49E+01 | 5.50E-04 | 1.38E-04 | 1.32E-04 |
| GOTERM_BP_DIRECT | GO:0051603~proteolysis involved in protein catabolic process | 7 | 4.93 | 1.60E-06 | DNAJC3, HSPA5, GZMA, MDM2, CTSH, CTSW, CTSS | 134 | 53 | 19478 | 1.92E+01 | 1.73E-03 | 1.93E-04 | 1.89E-04 |
| KEGG_PATHWAY | hsa04940:Type I diabetes mellitus | 7 | 4.93 | 1.74E-06 | HLA-DRB5, HLA-DPB1, PRF1, HLA-DRA, HLA-DRB1, HLA-DPA1, HLA-DQB1 | 74 | 44 | 8534 | 1.83E+01 | 2.97E-04 | 3.40E-05 | 2.94E-05 |
| KEGG_PATHWAY | hsa04640:Hematopoietic cell lineage | 9 | 6.34 | 1.92E-06 | HLA-DRB5, IL4R, CD8B, CD8A, HLA-DPB1, HLA-DRA, HLA-DRB1, HLA-DPA1, HLA-DQB1 | 74 | 100 | 8534 | 1.04E+01 | 3.29E-04 | 3.40E-05 | 2.94E-05 |
| KEGG_PATHWAY | hsa05416:Viral myocarditis | 8 | 5.63 | 1.95E-06 | HLA-DRB5, HLA-DPB1, PRF1, HLA-DRA, HLA-DRB1, ACTG1, HLA-DPA1, HLA-DQB1 | 74 | 70 | 8534 | 1.32E+01 | 3.34E-04 | 3.40E-05 | 2.94E-05 |
| KEGG_PATHWAY | hsa05332:Graft-versus-host disease | 7 | 4.93 | 1.99E-06 | HLA-DRB5, HLA-DPB1, PRF1, HLA-DRA, HLA-DRB1, HLA-DPA1, HLA-DQB1 | 74 | 45 | 8534 | 1.79E+01 | 3.40E-04 | 3.40E-05 | 2.94E-05 |
| GOTERM_BP_DIRECT | GO:0006968~cellular defense response | 7 | 4.93 | 2.47E-06 | SPN, ITGB1, TYROBP, GNLY, PRF1, MNDA, KLRG1 | 134 | 57 | 19478 | 1.79E+01 | 2.68E-03 | 2.68E-04 | 2.63E-04 |
| GOTERM_BP_DIRECT | GO:0030593~neutrophil chemotaxis | 7 | 4.93 | 3.03E-06 | LGALS3, FCER1G, JAML, PPIB, PPIA, S100A9, S100A8 | 134 | 59 | 19478 | 1.72E+01 | 3.29E-03 | 2.99E-04 | 2.93E-04 |
| SMART | SM00407:IGc1 | 8 | 5.63 | 3.65E-06 | HLA-DRB5, IGKC, HLA-DPB1, HLA-DRA, IGHA1, HLA-DRB1, HLA-DPA1, HLA-DQB1 | 94 | 75 | 10706 | 1.21E+01 | 3.32E-04 | 1.11E-04 | 1.03E-04 |
| KEGG_PATHWAY | hsa05140:Leishmaniasis | 8 | 5.63 | 4.44E-06 | ITGB1, HLA-DRB5, HLA-DPB1, HLA-DRA, FOS, HLA-DRB1, HLA-DPA1, HLA-DQB1 | 74 | 79 | 8534 | 1.17E+01 | 7.59E-04 | 6.90E-05 | 5.97E-05 |
| GOTERM_CC_DIRECT | GO:0005783~endoplasmic reticulum | 24 | 16.90 | 4.83E-06 | APP, GRN, HSPA5, SSR4, RPN1, RPLP0, CTSW, RRBP1, HMGB1, FOS, CST7, MESD, MTDH, HSP90B1, ERN1, DNAJC3, CST3, SPCS3, LMAN1, DNAJC1, CANX, CALR, PPIB, CLIC1 | 139 | 1213 | 20795 | 2.96E+00 | 1.52E-03 | 8.97E-05 | 8.00E-05 |
| GOTERM_BP_DIRECT | GO:0050778~positive regulation of immune response | 6 | 4.23 | 5.03E-06 | HLA-DRB5, HLA-DPB1, HLA-DRA, HLA-DRB1, HLA-DPA1, HLA-DQB1 | 134 | 37 | 19478 | 2.36E+01 | 5.46E-03 | 4.56E-04 | 4.47E-04 |
| GOTERM_BP_DIRECT | GO:0050870~positive regulation of T cell activation | 6 | 4.23 | 5.77E-06 | HLA-DRB5, HLA-DPB1, HLA-DRA, HLA-DRB1, HLA-DPA1, HLA-DQB1 | 134 | 38 | 19478 | 2.30E+01 | 6.25E-03 | 4.82E-04 | 4.72E-04 |
| KEGG_PATHWAY | hsa05320:Autoimmune thyroid disease | 7 | 4.93 | 5.94E-06 | HLA-DRB5, HLA-DPB1, PRF1, HLA-DRA, HLA-DRB1, HLA-DPA1, HLA-DQB1 | 74 | 54 | 8534 | 1.49E+01 | 1.01E-03 | 8.46E-05 | 7.32E-05 |
| UP_SEQ_FEATURE | DOMAIN:Ig-like C1-type | 6 | 4.23 | 6.03E-06 | HLA-DRB5, HLA-DPB1, HLA-DRA, HLA-DRB1, HLA-DPA1, HLA-DQB1 | 136 | 40 | 20675 | 2.28E+01 | 7.11E-03 | 7.93E-04 | 7.84E-04 |
| UP_KW_CELLULAR_COMPONENT | KW-1003~Cell membrane | 55 | 38.73 | 6.23E-06 | FCN1, ITGB1, APP, CLIC3, CD82, IGHV4-34, PRF1, IGHV4-39, HMGB1, CLIC1, CCR4, HLA-DPA1, RALGPS2, IGHV5-51, FCER1G, IL4R, APLP2, TNFRSF1B, TYROBP, CD8B, JAML, CD8A, IGKV4-1, TLN1, S100A9, S100A8, VCL, IGKV2-30, IGLV3-19, HLA-DQB1, IGLV3-1, AIF1, IGLV3-9, IGLV1-40, IGKC, IGLV6-57, IGLV2-14, IGLV3-21, IGKV3-15, IGLV2-18, IGHA1, IGKV3-11, SLAMF1, KLRG1, CD74, HLA-DRB5, IGKV1-5, NKG7, PTPRE, IGLV1-51, IGLV2-23, HLA-DPB1, HLA-DRA, HLA-DRB1, IGKV3-20 | 136 | 4134 | 18049 | 1.77E+00 | 1.81E-04 | 3.01E-05 | 2.18E-05 |
| GOTERM_CC_DIRECT | GO:0030669~clathrin-coated endocytic vesicle membrane | 7 | 4.93 | 7.70E-06 | CD74, HLA-DRB5, HLA-DPB1, HLA-DRA, HLA-DRB1, HLA-DPA1, HLA-DQB1 | 139 | 71 | 20795 | 1.47E+01 | 2.43E-03 | 1.35E-04 | 1.21E-04 |
| GOTERM_CC_DIRECT | GO:0032588~trans-Golgi network membrane | 8 | 5.63 | 9.87E-06 | CD74, APP, HLA-DRB5, HLA-DPB1, HLA-DRA, HLA-DRB1, HLA-DPA1, HLA-DQB1 | 139 | 112 | 20795 | 1.07E+01 | 3.11E-03 | 1.64E-04 | 1.46E-04 |
| KEGG_PATHWAY | hsa04658:Th1 and Th2 cell differentiation | 8 | 5.63 | 1.32E-05 | HLA-DRB5, IL4R, HLA-DPB1, HLA-DRA, FOS, HLA-DRB1, HLA-DPA1, HLA-DQB1 | 74 | 93 | 8534 | 9.92E+00 | 2.25E-03 | 1.73E-04 | 1.50E-04 |
| GOTERM_CC_DIRECT | GO:0034774~secretory granule lumen | 8 | 5.63 | 1.39E-05 | FCN1, GSTP1, CTSH, HMGB1, PPIA, S100A9, VCL, S100A8 | 139 | 118 | 20795 | 1.01E+01 | 4.38E-03 | 2.20E-04 | 1.96E-04 |
| INTERPRO | IPR011162:MHC_I/II-like_Ag-recog | 6 | 4.23 | 1.41E-05 | HLA-DRB5, HLA-DPB1, HLA-DRA, HLA-DRB1, HLA-DPA1, HLA-DQB1 | 138 | 47 | 20808 | 1.92E+01 | 6.74E-03 | 6.15E-04 | 5.93E-04 |
| KEGG_PATHWAY | hsa05164:Influenza A | 10 | 7.04 | 1.50E-05 | DNAJC3, HLA-DRB5, CDK6, CCL5, HLA-DPB1, HLA-DRA, HLA-DRB1, ACTG1, HLA-DPA1, HLA-DQB1 | 74 | 173 | 8534 | 6.67E+00 | 2.56E-03 | 1.73E-04 | 1.50E-04 |
| KEGG_PATHWAY | hsa05323:Rheumatoid arthritis | 8 | 5.63 | 1.52E-05 | HLA-DRB5, CCL5, HLA-DPB1, HLA-DRA, FOS, HLA-DRB1, HLA-DPA1, HLA-DQB1 | 74 | 95 | 8534 | 9.71E+00 | 2.59E-03 | 1.73E-04 | 1.50E-04 |
| GOTERM_CC_DIRECT | GO:0001772~immunological synapse | 6 | 4.23 | 1.63E-05 | LGALS3, GZMA, STX7, PRF1, HLA-DRA, HLA-DRB1 | 139 | 48 | 20795 | 1.87E+01 | 5.14E-03 | 2.45E-04 | 2.19E-04 |
| KEGG_PATHWAY | hsa05166:Human T-cell leukemia virus 1 infection | 11 | 7.75 | 1.86E-05 | HLA-DRB5, CCND2, CANX, HLA-DPB1, HLA-DRA, FOS, CALR, TLN1, HLA-DRB1, HLA-DPA1, HLA-DQB1 | 74 | 224 | 8534 | 5.66E+00 | 3.17E-03 | 1.94E-04 | 1.68E-04 |
| UP_KW_MOLECULAR_FUNCTION | KW-0143~Chaperone | 10 | 7.04 | 1.91E-05 | DNAJC3, SEC61A1, CD74, DNAJC1, HSPA5, CANX, ANP32E, CALR, MESD, HSP90B1 | 75 | 245 | 11952 | 6.50E+00 | 1.07E-03 | 1.07E-03 | 1.07E-03 |
| KEGG_PATHWAY | hsa05321:Inflammatory bowel disease | 7 | 4.93 | 1.93E-05 | HLA-DRB5, IL4R, HLA-DPB1, HLA-DRA, HLA-DRB1, HLA-DPA1, HLA-DQB1 | 74 | 66 | 8534 | 1.22E+01 | 3.29E-03 | 1.94E-04 | 1.68E-04 |
| GOTERM_MF_DIRECT | GO:0042605~peptide antigen binding | 6 | 4.23 | 2.04E-05 | HLA-DRB5, HLA-DPB1, HLA-DRA, HLA-DRB1, HLA-DPA1, HLA-DQB1 | 135 | 48 | 19253 | 1.78E+01 | 7.32E-03 | 1.47E-03 | 1.41E-03 |
| GOTERM_CC_DIRECT | GO:0005790~smooth endoplasmic reticulum | 5 | 3.52 | 2.11E-05 | DNAJC3, APP, CALR, PPIB, HSP90B1 | 139 | 25 | 20795 | 2.99E+01 | 6.64E-03 | 3.03E-04 | 2.70E-04 |
| UP_SEQ_FEATURE | REGION:Beta-2 | 4 | 2.82 | 2.22E-05 | HLA-DRB5, HLA-DPB1, HLA-DRB1, HLA-DQB1 | 136 | 9 | 20675 | 6.76E+01 | 2.59E-02 | 2.39E-03 | 2.36E-03 |
| UP_SEQ_FEATURE | REGION:Beta-1 | 4 | 2.82 | 2.22E-05 | HLA-DRB5, HLA-DPB1, HLA-DRB1, HLA-DQB1 | 136 | 9 | 20675 | 6.76E+01 | 2.59E-02 | 2.39E-03 | 2.36E-03 |
| GOTERM_BP_DIRECT | GO:0006457~protein folding | 9 | 6.34 | 2.84E-05 | LMAN1, DNAJC1, CANX, ANP32E, CALR, PPIB, MESD, PPIA, HSP90B1 | 134 | 176 | 19478 | 7.43E+00 | 3.04E-02 | 2.21E-03 | 2.16E-03 |
| GOTERM_CC_DIRECT | GO:0005925~focal adhesion | 13 | 9.15 | 3.26E-05 | ITGB1, TPM4, HSPA5, AHNAK, RPLP0, ACTG1, HSP90B1, CALR, PFN1, PPIB, TLN1, PPIA, VCL | 139 | 433 | 20795 | 4.49E+00 | 1.03E-02 | 4.49E-04 | 4.00E-04 |
| GOTERM_MF_DIRECT | GO:0051082~unfolded protein binding | 8 | 5.63 | 3.34E-05 | ERN1, LMAN1, HSPA5, CANX, CALR, PPIB, PPIA, HSP90B1 | 135 | 129 | 19253 | 8.84E+00 | 1.19E-02 | 2.00E-03 | 1.92E-03 |
| KEGG_PATHWAY | hsa04659:Th17 cell differentiation | 8 | 5.63 | 3.72E-05 | HLA-DRB5, IL4R, HLA-DPB1, HLA-DRA, FOS, HLA-DRB1, HLA-DPA1, HLA-DQB1 | 74 | 109 | 8534 | 8.46E+00 | 6.34E-03 | 3.54E-04 | 3.06E-04 |
| GOTERM_CC_DIRECT | GO:0005788~endoplasmic reticulum lumen | 11 | 7.75 | 5.34E-05 | DNAJC3, CST3, APP, VCAN, LGALS1, HSPA5, APLP2, CANX, CALR, PPIB, HSP90B1 | 139 | 318 | 20795 | 5.17E+00 | 1.67E-02 | 7.03E-04 | 6.27E-04 |
| KEGG_PATHWAY | hsa05169:Epstein-Barr virus infection | 10 | 7.04 | 5.52E-05 | HLA-DRB5, CDK6, CCND2, MDM2, HLA-DPB1, HLA-DRA, CALR, HLA-DRB1, HLA-DPA1, HLA-DQB1 | 74 | 204 | 8534 | 5.65E+00 | 9.39E-03 | 4.96E-04 | 4.30E-04 |
| GOTERM_MF_DIRECT | GO:0005509~calcium ion binding | 17 | 11.97 | 5.65E-05 | ITGB1, TPM4, HSPA5, ANXA2, PRF1, AOAH, AIF1, TTN, HSP90B1, NELL2, VCAN, CANX, S100A4, CALR, S100A9, S100A8, S100A10 | 135 | 739 | 19253 | 3.28E+00 | 2.01E-02 | 2.90E-03 | 2.79E-03 |
| GOTERM_CC_DIRECT | GO:0042470~melanosome | 7 | 4.93 | 6.12E-05 | ITGB1, HSPA5, ANXA2, RPN1, PPIB, SND1, HSP90B1 | 139 | 102 | 20795 | 1.03E+01 | 1.91E-02 | 7.73E-04 | 6.90E-04 |
| KEGG_PATHWAY | hsa04672:Intestinal immune network for IgA production | 6 | 4.23 | 6.26E-05 | HLA-DRB5, HLA-DPB1, HLA-DRA, HLA-DRB1, HLA-DPA1, HLA-DQB1 | 74 | 50 | 8534 | 1.38E+01 | 1.07E-02 | 5.35E-04 | 4.63E-04 |
| UP_KW_LIGAND | KW-0430~Lectin | 8 | 5.63 | 7.44E-05 | FCN1, LGALS3, LMAN1, VCAN, LGALS1, CANX, CALR, KLRG1 | 42 | 181 | 6987 | 7.35E+00 | 1.34E-03 | 6.70E-04 | 6.70E-04 |
| GOTERM_CC_DIRECT | GO:0005765~lysosomal membrane | 12 | 8.45 | 7.70E-05 | CD74, GRN, HLA-DRB5, AHNAK, ANXA2, STX7, PSAP, HLA-DPB1, HLA-DRA, HLA-DRB1, HLA-DPA1, HLA-DQB1 | 139 | 401 | 20795 | 4.48E+00 | 2.40E-02 | 9.36E-04 | 8.35E-04 |
| GOTERM_BP_DIRECT | GO:0016064~immunoglobulin mediated immune response | 7 | 4.93 | 8.83E-05 | IGHV5-51, CD74, IL4R, FCER1G, IGKC, IGHV4-34, IGHV4-39 | 134 | 106 | 19478 | 9.60E+00 | 9.15E-02 | 6.40E-03 | 6.27E-03 |
| UP_SEQ_FEATURE | DOMAIN:MHC class II beta chain N-terminal | 4 | 2.82 | 1.17E-04 | HLA-DRB5, HLA-DPB1, HLA-DRB1, HLA-DQB1 | 136 | 15 | 20675 | 4.05E+01 | 1.29E-01 | 1.15E-02 | 1.14E-02 |
| GOTERM_BP_DIRECT | GO:0046598~positive regulation of viral entry into host cell | 4 | 2.82 | 1.33E-04 | CD74, LGALS1, HMGB1, HLA-DRB1 | 134 | 15 | 19478 | 3.88E+01 | 1.35E-01 | 9.06E-03 | 8.88E-03 |
| KEGG_PATHWAY | hsa05152:Tuberculosis | 9 | 6.34 | 1.51E-04 | CD74, HLA-DRB5, FCER1G, HLA-DPB1, HLA-DRA, CTSS, HLA-DRB1, HLA-DPA1, HLA-DQB1 | 74 | 182 | 8534 | 5.70E+00 | 2.55E-02 | 1.17E-03 | 1.02E-03 |
| KEGG_PATHWAY | hsa05168:Herpes simplex virus 1 infection | 9 | 6.34 | 1.51E-04 | CD74, HLA-DRB5, CCL5, HLA-DPB1, HLA-DRA, CALR, HLA-DRB1, HLA-DPA1, HLA-DQB1 | 74 | 182 | 8534 | 5.70E+00 | 2.55E-02 | 1.17E-03 | 1.02E-03 |
| GOTERM_MF_DIRECT | GO:0042056~chemoattractant activity | 5 | 3.52 | 1.86E-04 | LGALS3, APP, CCL5, S100A4, HMGB1 | 135 | 41 | 19253 | 1.74E+01 | 6.48E-02 | 8.37E-03 | 8.05E-03 |
| INTERPRO | IPR000353:MHC_II_b_N | 4 | 2.82 | 2.12E-04 | HLA-DRB5, HLA-DPB1, HLA-DRB1, HLA-DQB1 | 138 | 18 | 20808 | 3.35E+01 | 9.65E-02 | 8.46E-03 | 8.16E-03 |
| GOTERM_CC_DIRECT | GO:0071748~monomeric IgA immunoglobulin complex | 3 | 2.11 | 2.60E-04 | IGHA1, IGKV3-20, JCHAIN | 139 | 4 | 20795 | 1.12E+02 | 7.89E-02 | 3.04E-03 | 2.72E-03 |
| UP_KW_CELLULAR_COMPONENT | KW-0472~Membrane | 84 | 59.15 | 2.61E-04 | FCN1, ITGB1, APP, CLIC3, CD82, MT-CO1, LST1, IGHV4-34, PRF1, TXNDC11, IGHV4-39, HMGB1, CLINT1, SPN, SEC61A1, CCND2, FTH1, CLIC1, CCR4, HLA-DPA1, RALGPS2, TMPO, IGHV5-51, FCER1G, IL4R, SSR4, APLP2, STX7, TNFRSF1B, ADAM19, ERN1, MS4A6A, DNAJC1, TYROBP, CD8B, JAML, CD8A, CANX, IGKV4-1, MT-CO2, TLN1, S100A9, S100A8, VCL, IGKV2-30, IGLV3-19, HLA-DQB1, CLDND1, RPN1, IGLV3-1, RRBP1, AIF1, UBE2J1, MTDH, LMAN1, IGLV3-9, IGLV1-40, RAB11FIP1, IGKC, IGLV6-57, CUX1, IGLV2-14, IGLV3-21, IGKV3-15, IGLV2-18, IGHA1, IGKV3-11, S100A10, SLAMF1, KLRG1, CD74, HLA-DRB5, IGKV1-5, NKG7, NELL2, SPCS3, PTPRE, IGLV1-51, GOLGB1, IGLV2-23, HLA-DPB1, HLA-DRA, HLA-DRB1, IGKV3-20 | 136 | 8353 | 18049 | 1.33E+00 | 7.54E-03 | 1.08E-03 | 7.83E-04 |
| GOTERM_BP_DIRECT | GO:1905686~positive regulation of plasma membrane repair | 3 | 2.11 | 2.75E-04 | AHNAK, ANXA2, S100A10 | 134 | 4 | 19478 | 1.09E+02 | 2.59E-01 | 1.76E-02 | 1.72E-02 |
| GOTERM_CC_DIRECT | GO:0005764~lysosome | 10 | 7.04 | 2.79E-04 | CD74, APP, GRN, STX7, PSAP, HLA-DRA, CTSH, CTSW, CST7, CTSS | 139 | 318 | 20795 | 4.70E+00 | 8.44E-02 | 3.15E-03 | 2.81E-03 |
| GOTERM_BP_DIRECT | GO:0070527~platelet aggregation | 5 | 3.52 | 3.47E-04 | TLN1, PPIA, CLIC1, VCL, ACTG1 | 134 | 49 | 19478 | 1.48E+01 | 3.14E-01 | 2.10E-02 | 2.05E-02 |
| GOTERM_CC_DIRECT | GO:0035578~azurophil granule lumen | 6 | 4.23 | 3.52E-04 | DNAJC3, GRN, ANXA2, MNDA, LYZ, FTL | 139 | 91 | 20795 | 9.86E+00 | 1.05E-01 | 3.84E-03 | 3.43E-03 |
| KEGG_PATHWAY | hsa05145:Toxoplasmosis | 7 | 4.93 | 3.72E-04 | ITGB1, HLA-DRB5, HLA-DPB1, HLA-DRA, HLA-DRB1, HLA-DPA1, HLA-DQB1 | 74 | 112 | 8534 | 7.21E+00 | 6.17E-02 | 2.77E-03 | 2.39E-03 |
| GOTERM_MF_DIRECT | GO:0045296~cadherin binding | 10 | 7.04 | 4.01E-04 | ITGB1, AHNAK, HSPA5, PFN1, TLN1, CLINT1, CLIC1, SND1, VCL, TMPO | 135 | 319 | 19253 | 4.47E+00 | 1.34E-01 | 1.60E-02 | 1.54E-02 |
| GOTERM_CC_DIRECT | GO:0071751~secretory IgA immunoglobulin complex | 3 | 2.11 | 4.32E-04 | IGHA1, IGKV3-20, JCHAIN | 139 | 5 | 20795 | 8.98E+01 | 1.28E-01 | 4.52E-03 | 4.03E-03 |
| GOTERM_BP_DIRECT | GO:2000406~positive regulation of T cell migration | 4 | 2.82 | 4.36E-04 | SPN, APP, CCL5, AIF1 | 134 | 22 | 19478 | 2.64E+01 | 3.77E-01 | 2.29E-02 | 2.24E-02 |
| GOTERM_BP_DIRECT | GO:0050918~positive chemotaxis | 5 | 3.52 | 4.36E-04 | LGALS3, APP, CCL5, S100A4, HMGB1 | 134 | 52 | 19478 | 1.40E+01 | 3.78E-01 | 2.29E-02 | 2.24E-02 |
| GOTERM_BP_DIRECT | GO:0031640~killing of cells of another organism | 6 | 4.23 | 4.42E-04 | LGALS3, GNLY, GZMA, PRF1, NKG7, LYZ | 134 | 93 | 19478 | 9.38E+00 | 3.82E-01 | 2.29E-02 | 2.24E-02 |
| GOTERM_CC_DIRECT | GO:0009897~external side of plasma membrane | 11 | 7.75 | 4.43E-04 | SPN, ITGB1, FCN1, CD74, IL4R, FCER1G, CD8A, CALR, CCR4, HLA-DRB1, SLAMF1 | 139 | 413 | 20795 | 3.98E+00 | 1.31E-01 | 4.52E-03 | 4.03E-03 |
| SMART | SM00921:MHC_II_beta | 4 | 2.82 | 4.71E-04 | HLA-DRB5, HLA-DPB1, HLA-DRB1, HLA-DQB1 | 94 | 18 | 10706 | 2.53E+01 | 4.20E-02 | 1.07E-02 | 1.00E-02 |
| GOTERM_BP_DIRECT | GO:0090026~positive regulation of monocyte chemotaxis | 4 | 2.82 | 4.99E-04 | APP, CCL5, HMGB1, AIF1 | 134 | 23 | 19478 | 2.53E+01 | 4.19E-01 | 2.46E-02 | 2.41E-02 |
| UP_SEQ_FEATURE | TOPO_DOM:Lumenal | 14 | 9.86 | 5.20E-04 | SSR4, RPN1, RRBP1, UBE2J1, MTDH, ERN1, SEC61A1, SPCS3, LMAN1, DNAJC1, CUX1, GOLGB1, CANX, TMPO | 136 | 680 | 20675 | 3.13E+00 | 4.59E-01 | 4.44E-02 | 4.39E-02 |
| GOTERM_CC_DIRECT | GO:1904724~tertiary granule lumen | 5 | 3.52 | 5.22E-04 | CST3, FTH1, CTSH, LYZ, CTSS | 139 | 56 | 20795 | 1.34E+01 | 1.52E-01 | 5.15E-03 | 4.60E-03 |
| UP_SEQ_FEATURE | MOTIF:Prevents secretion from ER | 5 | 3.52 | 5.25E-04 | HSPA5, CALR, PPIB, MESD, HSP90B1 | 136 | 57 | 20675 | 1.33E+01 | 4.63E-01 | 4.44E-02 | 4.39E-02 |
| INTERPRO | IPR001751:S100/CaBP7/8-like_CS | 4 | 2.82 | 5.78E-04 | S100A4, S100A9, S100A8, S100A10 | 138 | 25 | 20808 | 2.41E+01 | 2.42E-01 | 2.13E-02 | 2.05E-02 |
| GOTERM_MF_DIRECT | GO:0043236~laminin binding | 4 | 2.82 | 5.99E-04 | ITGB1, LGALS3, LGALS1, CTSS | 135 | 24 | 19253 | 2.38E+01 | 1.94E-01 | 2.16E-02 | 2.07E-02 |
| INTERPRO | IPR011001:Saposin-like | 3 | 2.11 | 6.34E-04 | GNLY, PSAP, AOAH | 138 | 6 | 20808 | 7.54E+01 | 2.62E-01 | 2.17E-02 | 2.09E-02 |
| GOTERM_CC_DIRECT | GO:0031902~late endosome membrane | 7 | 4.93 | 6.37E-04 | HLA-DRB5, ANXA2, HLA-DPB1, HLA-DRA, HLA-DRB1, HLA-DPA1, HLA-DQB1 | 139 | 157 | 20795 | 6.67E+00 | 1.82E-01 | 6.10E-03 | 5.45E-03 |
| GOTERM_MF_DIRECT | GO:0030246~carbohydrate binding | 8 | 5.63 | 7.57E-04 | FCN1, LGALS3, LMAN1, VCAN, LGALS1, CANX, CALR, KLRG1 | 135 | 214 | 19253 | 5.33E+00 | 2.39E-01 | 2.31E-02 | 2.22E-02 |
| GOTERM_BP_DIRECT | GO:0006954~inflammatory response | 11 | 7.75 | 7.75E-04 | CCL5, NKG7, FOS, HMGB1, LYZ, TNFRSF1B, CCR4, AIF1, S100A9, S100A8, KLRG1 | 134 | 432 | 19478 | 3.70E+00 | 5.69E-01 | 3.66E-02 | 3.59E-02 |
| GOTERM_CC_DIRECT | GO:0031982~vesicle | 7 | 4.93 | 8.01E-04 | CST3, AHNAK, ANXA2, GSTP1, STX7, PPIA, CLIC1 | 139 | 164 | 20795 | 6.39E+00 | 2.24E-01 | 7.44E-03 | 6.64E-03 |
| GOTERM_MF_DIRECT | GO:0044183~protein folding chaperone | 5 | 3.52 | 8.09E-04 | CD74, HSPA5, ANP32E, CALR, HSP90B1 | 135 | 60 | 19253 | 1.19E+01 | 2.53E-01 | 2.31E-02 | 2.22E-02 |
| INTERPRO | IPR013787:S100_Ca-bd_sub | 4 | 2.82 | 8.11E-04 | S100A4, S100A9, S100A8, S100A10 | 138 | 28 | 20808 | 2.15E+01 | 3.22E-01 | 2.49E-02 | 2.40E-02 |
| UP_SEQ_FEATURE | DOMAIN:Cathepsin propeptide inhibitor | 3 | 2.11 | 8.70E-04 | CTSH, CTSW, CTSS | 136 | 7 | 20675 | 6.52E+01 | 6.43E-01 | 6.43E-02 | 6.36E-02 |
| UP_SEQ_FEATURE | DOMAIN:Saposin B-type | 3 | 2.11 | 8.70E-04 | GNLY, PSAP, AOAH | 136 | 7 | 20675 | 6.52E+01 | 6.43E-01 | 6.43E-02 | 6.36E-02 |
| GOTERM_MF_DIRECT | GO:0051087~protein-folding chaperone binding | 6 | 4.23 | 8.75E-04 | DNAJC3, APP, GRN, DNAJC1, HSPA5, CALR | 135 | 106 | 19253 | 8.07E+00 | 2.70E-01 | 2.31E-02 | 2.22E-02 |
| INTERPRO | IPR008139:SaposinB_dom | 3 | 2.11 | 8.84E-04 | GNLY, PSAP, AOAH | 138 | 7 | 20808 | 6.46E+01 | 3.45E-01 | 2.49E-02 | 2.40E-02 |
| INTERPRO | IPR001003:MHC_II_a_N | 3 | 2.11 | 8.84E-04 | HLA-DPB1, HLA-DRA, HLA-DPA1 | 138 | 7 | 20808 | 6.46E+01 | 3.45E-01 | 2.49E-02 | 2.40E-02 |
| GOTERM_MF_DIRECT | GO:0005178~integrin binding | 7 | 4.93 | 8.97E-04 | ITGB1, APP, JAML, HMGB1, CALR, TLN1, PPIA | 135 | 160 | 19253 | 6.24E+00 | 2.76E-01 | 2.31E-02 | 2.22E-02 |
| GOTERM_BP_DIRECT | GO:0042102~positive regulation of T cell proliferation | 5 | 3.52 | 9.06E-04 | SPN, CCL5, HLA-DPB1, AIF1, HLA-DPA1 | 134 | 63 | 19478 | 1.15E+01 | 6.27E-01 | 4.10E-02 | 4.02E-02 |
| GOTERM_BP_DIRECT | GO:0050729~positive regulation of inflammatory response | 6 | 4.23 | 1.03E-03 | APP, LGALS1, NKG7, NEAT1, S100A9, S100A8 | 134 | 112 | 19478 | 7.79E+00 | 6.74E-01 | 4.48E-02 | 4.39E-02 |
| KEGG_PATHWAY | hsa04210:Apoptosis | 7 | 4.93 | 1.04E-03 | ERN1, PRF1, CTSH, CTSW, FOS, CTSS, ACTG1 | 74 | 136 | 8534 | 5.94E+00 | 1.63E-01 | 7.41E-03 | 6.42E-03 |
| SMART | SM00741:SapB | 3 | 2.11 | 1.09E-03 | GNLY, PSAP, AOAH | 94 | 6 | 10706 | 5.69E+01 | 9.49E-02 | 1.98E-02 | 1.85E-02 |
| GOTERM_MF_DIRECT | GO:0002020~protease binding | 6 | 4.23 | 1.12E-03 | ITGB1, CST3, ANXA2, PSAP, CST7, TTN | 135 | 112 | 19253 | 7.64E+00 | 3.32E-01 | 2.66E-02 | 2.56E-02 |
| INTERPRO | IPR039417:Peptidase_C1A_papain-like | 3 | 2.11 | 1.17E-03 | CTSH, CTSW, CTSS | 138 | 8 | 20808 | 5.65E+01 | 4.30E-01 | 2.96E-02 | 2.85E-02 |
| INTERPRO | IPR013201:Prot_inhib_I29 | 3 | 2.11 | 1.17E-03 | CTSH, CTSW, CTSS | 138 | 8 | 20808 | 5.65E+01 | 4.30E-01 | 2.96E-02 | 2.85E-02 |
| GOTERM_MF_DIRECT | GO:0042802~identical protein binding | 25 | 17.61 | 1.18E-03 | APP, AHNAK, PRF1, MESD, SAT1, ACTG1, TTN, CST3, CCL5, FTH1, PSAP, SLAMF1, CD74, FCER1G, TPM4, ANXA2, APLP2, FOS, LYZ, NELL2, ERN1, TYROBP, MDM2, S100A4, FTL | 135 | 1777 | 19253 | 2.01E+00 | 3.47E-01 | 2.66E-02 | 2.56E-02 |
| SMART | SM00848:Inhibitor_I29 | 3 | 2.11 | 1.52E-03 | CTSH, CTSW, CTSS | 94 | 7 | 10706 | 4.88E+01 | 1.30E-01 | 1.98E-02 | 1.85E-02 |
| SMART | SM00920:MHC_II_alpha | 3 | 2.11 | 1.52E-03 | HLA-DPB1, HLA-DRA, HLA-DPA1 | 94 | 7 | 10706 | 4.88E+01 | 1.30E-01 | 1.98E-02 | 1.85E-02 |
| KEGG_PATHWAY | hsa05150:Staphylococcus aureus infection | 6 | 4.23 | 1.74E-03 | HLA-DRB5, HLA-DPB1, HLA-DRA, HLA-DRB1, HLA-DPA1, HLA-DQB1 | 74 | 102 | 8534 | 6.78E+00 | 2.58E-01 | 1.19E-02 | 1.03E-02 |
| SMART | SM01394:S_100 | 4 | 2.82 | 1.78E-03 | S100A4, S100A9, S100A8, S100A10 | 94 | 28 | 10706 | 1.63E+01 | 1.49E-01 | 2.02E-02 | 1.89E-02 |
| UP_SEQ_FEATURE | TOPO_DOM:Cytoplasmic | 41 | 28.87 | 1.81E-03 | ITGB1, APP, CD82, RPN1, RRBP1, UBE2J1, MTDH, SPN, LMAN1, SEC61A1, CUX1, IGHA1, CCR4, SLAMF1, HLA-DPA1, KLRG1, CD74, HLA-DRB5, FCER1G, IL4R, SSR4, APLP2, STX7, TNFRSF1B, NELL2, ADAM19, ERN1, SPCS3, PTPRE, MS4A6A, DNAJC1, TYROBP, CD8B, JAML, CD8A, GOLGB1, CANX, HLA-DPB1, HLA-DRA, HLA-DRB1, HLA-DQB1 | 136 | 3923 | 20675 | 1.59E+00 | 8.83E-01 | 1.26E-01 | 1.25E-01 |
| INTERPRO | IPR025661:Pept_asp_AS | 3 | 2.11 | 1.87E-03 | CTSH, CTSW, CTSS | 138 | 10 | 20808 | 4.52E+01 | 5.92E-01 | 4.48E-02 | 4.32E-02 |
| UP_SEQ_FEATURE | REGION:Connecting peptide | 4 | 2.82 | 1.94E-03 | HLA-DPB1, HLA-DRA, HLA-DPA1, HLA-DQB1 | 136 | 38 | 20675 | 1.60E+01 | 9.00E-01 | 1.28E-01 | 1.26E-01 |
| GOTERM_BP_DIRECT | GO:0010628~positive regulation of gene expression | 11 | 7.75 | 1.95E-03 | IL32, ADAM19, CD74, APP, TYROBP, CDK6, MDM2, CTSH, CALR, ACTG1, TTN | 134 | 489 | 19478 | 3.27E+00 | 8.81E-01 | 8.17E-02 | 8.01E-02 |
| UP_KW_PTM | KW-0873~Pyrrolidone carboxylic acid | 6 | 4.23 | 2.06E-03 | IGLV1-51, IGLV1-40, IGLV2-14, IGLV2-23, IGLV2-18, JCHAIN | 127 | 102 | 14316 | 6.63E+00 | 3.84E-02 | 2.06E-02 | 1.65E-02 |
| UP_KW_BIOLOGICAL_PROCESS | KW-0945~Host-virus interaction | 14 | 9.86 | 2.26E-03 | ITGB1, NUP214, HSPA5, ANXA2, HMGB1, SND1, NELL2, CANX, MDM2, HLA-DRA, TOP1, TLN1, PPIA, SLAMF1 | 89 | 695 | 11523 | 2.61E+00 | 9.90E-02 | 3.47E-02 | 3.32E-02 |
| INTERPRO | IPR025660:Pept_his_AS | 3 | 2.11 | 2.28E-03 | CTSH, CTSW, CTSS | 138 | 11 | 20808 | 4.11E+01 | 6.64E-01 | 5.19E-02 | 5.01E-02 |
| GOTERM_CC_DIRECT | GO:0034663~endoplasmic reticulum chaperone complex | 3 | 2.11 | 2.31E-03 | HSPA5, PPIB, HSP90B1 | 139 | 11 | 20795 | 4.08E+01 | 5.19E-01 | 2.09E-02 | 1.86E-02 |
| GOTERM_MF_DIRECT | GO:0048306~calcium-dependent protein binding | 5 | 3.52 | 2.58E-03 | ANXA2, S100A4, S100A9, S100A8, S100A10 | 135 | 82 | 19253 | 8.70E+00 | 6.05E-01 | 5.46E-02 | 5.25E-02 |
| UP_SEQ_FEATURE | DOMAIN:Peptidase C1A papain C-terminal | 3 | 2.11 | 2.68E-03 | CTSH, CTSW, CTSS | 136 | 12 | 20675 | 3.80E+01 | 9.58E-01 | 1.67E-01 | 1.65E-01 |
| GOTERM_CC_DIRECT | GO:0045121~membrane raft | 7 | 4.93 | 2.88E-03 | ITGB1, APP, AHNAK, ANXA2, TNFRSF1B, VCL, S100A10 | 139 | 211 | 20795 | 4.96E+00 | 5.98E-01 | 2.53E-02 | 2.26E-02 |
| GOTERM_BP_DIRECT | GO:0034121~regulation of toll-like receptor signaling pathway | 3 | 2.11 | 2.92E-03 | APP, S100A9, S100A8 | 134 | 12 | 19478 | 3.63E+01 | 9.58E-01 | 1.18E-01 | 1.15E-01 |
| INTERPRO | IPR001039:MHC_I_a_a1/a2 | 3 | 2.11 | 3.20E-03 | HLA-DPB1, HLA-DRB1, HLA-DQB1 | 138 | 13 | 20808 | 3.48E+01 | 7.85E-01 | 6.39E-02 | 6.16E-02 |
| INTERPRO | IPR013128:Peptidase_C1A | 3 | 2.11 | 3.20E-03 | CTSH, CTSW, CTSS | 138 | 13 | 20808 | 3.48E+01 | 7.85E-01 | 6.39E-02 | 6.16E-02 |
| INTERPRO | IPR000668:Peptidase_C1A_C | 3 | 2.11 | 3.20E-03 | CTSH, CTSW, CTSS | 138 | 13 | 20808 | 3.48E+01 | 7.85E-01 | 6.39E-02 | 6.16E-02 |
| GOTERM_BP_DIRECT | GO:0032722~positive regulation of chemokine production | 4 | 2.82 | 3.58E-03 | CD74, APP, IL4R, AIF1 | 134 | 45 | 19478 | 1.29E+01 | 9.80E-01 | 1.39E-01 | 1.36E-01 |
| GOTERM_BP_DIRECT | GO:0036503~ERAD pathway | 5 | 3.52 | 4.25E-03 | HSPA5, CANX, CALR, HSP90B1, UBE2J1 | 134 | 96 | 19478 | 7.57E+00 | 9.90E-01 | 1.59E-01 | 1.56E-01 |
| GOTERM_CC_DIRECT | GO:0005770~late endosome | 6 | 4.23 | 4.43E-03 | CD74, GRN, STX7, PSAP, CST7, CTSS | 139 | 161 | 20795 | 5.58E+00 | 7.54E-01 | 3.79E-02 | 3.38E-02 |
| UP_SEQ_FEATURE | CARBOHYD:N-linked (GlcNAc...) asparagine | 43 | 30.28 | 5.24E-03 | ITGB1, FCN1, APP, GRN, CLDND1, CD82, RPN1, IGHV4-34, FGL2, PRF1, CTSW, AOAH, CST7, MESD, CTSS, HSP90B1, SPN, PSAP, CTSH, CCR4, SLAMF1, HLA-DPA1, KLRG1, CD74, HLA-DRB5, IL4R, GZMA, TNFRSF1B, NELL2, ADAM19, ERN1, SPCS3, PTPRE, VCAN, CD8B, JAML, HLA-DPB1, HLA-DRA, CALR, PPIB, PPIA, HLA-DRB1, HLA-DQB1 | 136 | 4423 | 20675 | 1.48E+00 | 9.98E-01 | 3.10E-01 | 3.07E-01 |
| GOTERM_BP_DIRECT | GO:0032755~positive regulation of interleukin-6 production | 5 | 3.52 | 5.27E-03 | CD74, APP, TYROBP, HMGB1, AIF1 | 134 | 102 | 19478 | 7.13E+00 | 9.97E-01 | 1.91E-01 | 1.87E-01 |
| SMART | SM00645:Pept_C1 | 3 | 2.11 | 5.47E-03 | CTSH, CTSW, CTSS | 94 | 13 | 10706 | 2.63E+01 | 3.93E-01 | 5.53E-02 | 5.17E-02 |
| GOTERM_CC_DIRECT | GO:0098797~plasma membrane protein complex | 3 | 2.11 | 5.57E-03 | AHNAK, ANXA2, S100A10 | 139 | 17 | 20795 | 2.64E+01 | 8.29E-01 | 4.63E-02 | 4.13E-02 |
| GOTERM_BP_DIRECT | GO:0014002~astrocyte development | 3 | 2.11 | 5.88E-03 | CDK6, S100A9, S100A8 | 134 | 17 | 19478 | 2.57E+01 | 9.98E-01 | 2.06E-01 | 2.02E-01 |
| UP_KW_PTM | KW-0558~Oxidation | 4 | 2.82 | 6.36E-03 | APP, CCL5, HMGB1, ACTG1 | 127 | 43 | 14316 | 1.05E+01 | 1.14E-01 | 4.24E-02 | 3.39E-02 |
| GOTERM_MF_DIRECT | GO:0042803~protein homodimerization activity | 13 | 9.15 | 6.36E-03 | APP, FCER1G, TPM4, GZMA, CST7, TTN, JCHAIN, ERN1, TYROBP, JAML, CCL5, PSAP, S100A10 | 135 | 753 | 19253 | 2.46E+00 | 9.00E-01 | 1.27E-01 | 1.22E-01 |
| GOTERM_BP_DIRECT | GO:0042742~defense response to bacterium | 6 | 4.23 | 6.41E-03 | SPN, FCER1G, GNLY, LYZ, S100A9, S100A8 | 134 | 171 | 19478 | 5.10E+00 | 9.99E-01 | 2.18E-01 | 2.13E-01 |
| UP_KW_CELLULAR_COMPONENT | KW-0967~Endosome | 13 | 9.15 | 6.45E-03 | ITGB1, CD74, APP, HLA-DRB5, STX7, PRF1, HMGB1, RAB11FIP1, HLA-DPB1, HLA-DRA, HLA-DRB1, HLA-DPA1, HLA-DQB1 | 136 | 702 | 18049 | 2.46E+00 | 1.71E-01 | 2.34E-02 | 1.69E-02 |
| UP_SEQ_FEATURE | TRANSMEM:Helical | 50 | 35.21 | 6.91E-03 | ITGB1, APP, CLIC3, CD82, MT-CO1, LST1, TXNDC11, SPN, SEC61A1, FTH1, CCR4, HLA-DPA1, TMPO, FCER1G, IL4R, SSR4, APLP2, STX7, TNFRSF1B, ADAM19, ERN1, MS4A6A, DNAJC1, TYROBP, CD8B, JAML, CD8A, CANX, MT-CO2, HLA-DQB1, CLDND1, RPN1, RRBP1, UBE2J1, MTDH, LMAN1, CUX1, IGHA1, S100A10, SLAMF1, KLRG1, CD74, HLA-DRB5, NKG7, NELL2, PTPRE, GOLGB1, HLA-DPB1, HLA-DRA, HLA-DRB1 | 136 | 5451 | 20675 | 1.39E+00 | 1.00E+00 | 3.80E-01 | 3.76E-01 |
| KEGG_PATHWAY | hsa05322:Systemic lupus erythematosus | 6 | 4.23 | 7.01E-03 | HLA-DRB5, HLA-DPB1, HLA-DRA, HLA-DRB1, HLA-DPA1, HLA-DQB1 | 74 | 141 | 8534 | 4.91E+00 | 7.00E-01 | 4.61E-02 | 3.99E-02 |
| UP_SEQ_FEATURE | CROSSLNK:Glycyl lysine isopeptide (Lys-Gly) (interchain with G-Cter in SUMO1); alternate | 6 | 4.23 | 7.07E-03 | ITGB1, AHNAK, HSPA5, ANXA2, RPLP0, TOP1 | 136 | 183 | 20675 | 4.98E+00 | 1.00E+00 | 3.80E-01 | 3.76E-01 |
| GOTERM_MF_DIRECT | GO:0003723~RNA binding | 20 | 14.08 | 7.13E-03 | GRN, ANXA2, AHNAK, RPN1, RPLP0, RRBP1, HMGB1, SND1, MTDH, HSP90B1, LGALS3, LGALS1, GOLGB1, CANX, S100A4, CALR, TOP1, PFN1, PPIB, PPIA | 135 | 1485 | 19253 | 1.92E+00 | 9.24E-01 | 1.35E-01 | 1.30E-01 |
| GOTERM_MF_DIRECT | GO:0046914~transition metal ion binding | 3 | 2.11 | 7.61E-03 | APP, APLP2, S100A4 | 135 | 19 | 19253 | 2.25E+01 | 9.36E-01 | 1.37E-01 | 1.32E-01 |
| GOTERM_BP_DIRECT | GO:0003094~glomerular filtration | 3 | 2.11 | 8.11E-03 | IGHA1, IGKV3-20, JCHAIN | 134 | 20 | 19478 | 2.18E+01 | 1.00E+00 | 2.67E-01 | 2.62E-01 |
| UP_KW_LIGAND | KW-0186~Copper | 4 | 2.82 | 8.13E-03 | APP, MT-CO1, APLP2, MT-CO2 | 42 | 71 | 6987 | 9.37E+00 | 1.37E-01 | 4.88E-02 | 4.88E-02 |
| UP_KW_BIOLOGICAL_PROCESS | KW-0204~Cytolysis | 3 | 2.11 | 8.15E-03 | GZMA, PRF1, NKG7 | 89 | 18 | 11523 | 2.16E+01 | 3.14E-01 | 9.37E-02 | 8.96E-02 |
| UP_SEQ_FEATURE | CROSSLNK:Glycyl lysine isopeptide (Lys-Gly) (interchain with G-Cter in SUMO2); alternate | 8 | 5.63 | 8.21E-03 | AHNAK, ANXA2, RPN1, RPLP0, TOP1, PFN1, PPIA, S100A10 | 136 | 350 | 20675 | 3.47E+00 | 1.00E+00 | 4.22E-01 | 4.17E-01 |
| BIOCARTA | h_eosinophilsPathway:The Role of Eosinophils in the Chemokine Network of Allergy | 3 | 2.11 | 8.64E-03 | CCL5, HLA-DRA, HLA-DRB1 | 31 | 8 | 1622 | 1.96E+01 | 5.73E-01 | 8.04E-01 | 8.04E-01 |
| GOTERM_BP_DIRECT | GO:0032930~positive regulation of superoxide anion generation | 3 | 2.11 | 8.93E-03 | APP, TYROBP, GSTP1 | 134 | 21 | 19478 | 2.08E+01 | 1.00E+00 | 2.85E-01 | 2.80E-01 |
| GOTERM_BP_DIRECT | GO:0050852~T cell receptor signaling pathway | 5 | 3.52 | 9.55E-03 | CD8B, CD8A, HLA-DPB1, HLA-DRB1, HLA-DQB1 | 134 | 121 | 19478 | 6.01E+00 | 1.00E+00 | 2.96E-01 | 2.90E-01 |
| GOTERM_BP_DIRECT | GO:0001934~positive regulation of protein phosphorylation | 5 | 3.52 | 1.10E-02 | CD74, APP, CCND2, PPIA, HLA-DRB1 | 134 | 126 | 19478 | 5.77E+00 | 1.00E+00 | 3.31E-01 | 3.24E-01 |
| OMIM_DISEASE | 126200~Multiple sclerosis, susceptibility to, 1 | 2 | 1.41 | 1.11E-02 | HLA-DRB1, HLA-DQB1 | 35 | 2 | 6103 | 1.74E+02 | 5.11E-01 | 7.11E-01 | 7.11E-01 |
| UP_KW_PTM | KW-0654~Proteoglycan | 5 | 3.52 | 1.13E-02 | SRGN, CD74, APP, VCAN, APLP2 | 127 | 99 | 14316 | 5.69E+00 | 1.95E-01 | 5.66E-02 | 4.53E-02 |
| GOTERM_CC_DIRECT | GO:0048471~perinuclear region of cytoplasm | 12 | 8.45 | 1.14E-02 | ITGB1, APP, STX7, S100A4, CALR, PPIB, TNFRSF1B, CLINT1, AIF1, CLIC1, HSP90B1, MTDH | 139 | 750 | 20795 | 2.39E+00 | 9.74E-01 | 9.26E-02 | 8.27E-02 |
| GOTERM_BP_DIRECT | GO:0051893~regulation of focal adhesion assembly | 3 | 2.11 | 1.16E-02 | TLN1, VCL, ACTG1 | 134 | 24 | 19478 | 1.82E+01 | 1.00E+00 | 3.40E-01 | 3.33E-01 |
| UP_SEQ_FEATURE | MUTAGEN:L->R: Decreases the interaction with CD4. | 2 | 1.41 | 1.30E-02 | HLA-DRA, HLA-DRB1 | 136 | 2 | 20675 | 1.52E+02 | 1.00E+00 | 5.94E-01 | 5.88E-01 |
| UP_SEQ_FEATURE | SITE:Required for Cu(2+) reduction | 2 | 1.41 | 1.30E-02 | APP, APLP2 | 136 | 2 | 20675 | 1.52E+02 | 1.00E+00 | 5.94E-01 | 5.88E-01 |
| UP_SEQ_FEATURE | CARBOHYD:O-linked (GalNAc...) serine | 4 | 2.82 | 1.31E-02 | SPN, CD74, IGHA1, TNFRSF1B | 136 | 75 | 20675 | 8.11E+00 | 1.00E+00 | 5.94E-01 | 5.88E-01 |
| GOTERM_CC_DIRECT | GO:0045335~phagocytic vesicle | 4 | 2.82 | 1.32E-02 | CD82, CTSS, ACTG1, SLAMF1 | 139 | 74 | 20795 | 8.09E+00 | 9.85E-01 | 9.42E-02 | 8.40E-02 |
| GOTERM_CC_DIRECT | GO:0070288~ferritin complex | 2 | 1.41 | 1.32E-02 | FTH1, FTL | 139 | 2 | 20795 | 1.50E+02 | 9.85E-01 | 9.42E-02 | 8.40E-02 |
| GOTERM_CC_DIRECT | GO:1990660~calprotectin complex | 2 | 1.41 | 1.32E-02 | S100A9, S100A8 | 139 | 2 | 20795 | 1.50E+02 | 9.85E-01 | 9.42E-02 | 8.40E-02 |
| GOTERM_CC_DIRECT | GO:1990665~AnxA2-p11 complex | 2 | 1.41 | 1.32E-02 | ANXA2, S100A10 | 139 | 2 | 20795 | 1.50E+02 | 9.85E-01 | 9.42E-02 | 8.40E-02 |
| GOTERM_CC_DIRECT | GO:0010008~endosome membrane | 7 | 4.93 | 1.34E-02 | ITGB1, HLA-DRB5, HLA-DPB1, HLA-DRA, HLA-DRB1, HLA-DPA1, HLA-DQB1 | 139 | 292 | 20795 | 3.59E+00 | 9.86E-01 | 9.42E-02 | 8.40E-02 |
| GOTERM_CC_DIRECT | GO:0031901~early endosome membrane | 6 | 4.23 | 1.34E-02 | APP, CD8B, STX7, HLA-DPB1, HLA-DRA, HLA-DQB1 | 139 | 211 | 20795 | 4.25E+00 | 9.86E-01 | 9.42E-02 | 8.40E-02 |
| GOTERM_BP_DIRECT | GO:0070206~protein trimerization | 2 | 1.41 | 1.36E-02 | CD74, APP | 134 | 2 | 19478 | 1.45E+02 | 1.00E+00 | 3.57E-01 | 3.50E-01 |
| GOTERM_BP_DIRECT | GO:0070488~neutrophil aggregation | 2 | 1.41 | 1.36E-02 | S100A9, S100A8 | 134 | 2 | 19478 | 1.45E+02 | 1.00E+00 | 3.57E-01 | 3.50E-01 |
| GOTERM_BP_DIRECT | GO:0001909~leukocyte mediated cytotoxicity | 2 | 1.41 | 1.36E-02 | STXBP2, PRF1 | 134 | 2 | 19478 | 1.45E+02 | 1.00E+00 | 3.57E-01 | 3.50E-01 |
| GOTERM_BP_DIRECT | GO:0002491~antigen processing and presentation of endogenous peptide antigen via MHC class II | 2 | 1.41 | 1.36E-02 | HLA-DRA, HLA-DRB1 | 134 | 2 | 19478 | 1.45E+02 | 1.00E+00 | 3.57E-01 | 3.50E-01 |
| GOTERM_MF_DIRECT | GO:0005515~protein binding | 108 | 76.06 | 1.38E-02 | FCN1, APP, CD82, MT-CO1, PRF1, TXNDC11, SAT1, ACTG1, LGALS3, LGALS1, CCND2, FTH1, TMPO, NUP214, IL4R, TPM4, APLP2, STX7, SND1, ADAM19, ERN1, TYROBP, JAML, CD8B, CD8A, DDIT4, S100A4, MT-CO2, PFN1, PPIB, TLN1, PPIA, S100A9, S100A8, VCL, FTL, SP140, RPN1, TSHZ2, STXBP2, UBE2J1, HSP90B1, MTDH, SEC14L1, IGLV6-57, PSAP, SLAMF1, S100A10, HLA-DRB5, HSPA5, NKG7, PTPRE, CDK6, GOLGB1, MDM2, ANP32E, HLA-DRA, CALR, HLA-DRB1, ITGB1, CLIC3, RPLP0, HMGB1, CLINT1, CTSS, SPN, SEC61A1, GNLY, CTSH, CLIC1, CCR4, HLA-DPA1, RALGPS2, SRGN, FCER1G, ANXA2, FOS, TNFRSF1B, MS4A6A, VCAN, DNAJC1, CANX, TOP1, HLA-DQB1, GRN, CLDND1, AHNAK, GSTP1, FGL2, RRBP1, AOAH, CST7, MESD, AIF1, TTN, CST3, LMAN1, CCL5, KLRG1, IL32, CD74, GZMA, NELL2, GZMK, SPCS3, HLA-DPB1, MNDA, MYO1F | 135 | 13690 | 19253 | 1.13E+00 | 9.93E-01 | 2.19E-01 | 2.11E-01 |
| GOTERM_BP_DIRECT | GO:0061844~antimicrobial humoral immune response mediated by antimicrobial peptide | 5 | 3.52 | 1.38E-02 | LGALS3, APP, GNLY, CCL5, S100A9 | 134 | 135 | 19478 | 5.38E+00 | 1.00E+00 | 3.57E-01 | 3.50E-01 |
| GOTERM_MF_DIRECT | GO:0097493~structural molecule activity conferring elasticity | 2 | 1.41 | 1.39E-02 | AHNAK, TTN | 135 | 2 | 19253 | 1.43E+02 | 9.93E-01 | 2.19E-01 | 2.11E-01 |
| GOTERM_MF_DIRECT | GO:0050750~low-density lipoprotein particle receptor binding | 3 | 2.11 | 1.40E-02 | APP, MESD, HSP90B1 | 135 | 26 | 19253 | 1.65E+01 | 9.94E-01 | 2.19E-01 | 2.11E-01 |
| UP_SEQ_FEATURE | PROPEP:Activation peptide | 4 | 2.82 | 1.40E-02 | GZMK, GZMA, CTSH, CTSS | 136 | 77 | 20675 | 7.90E+00 | 1.00E+00 | 6.14E-01 | 6.07E-01 |
| KEGG_PATHWAY | hsa04820:Cytoskeleton in muscle cells | 7 | 4.93 | 1.41E-02 | ITGB1, VCAN, TPM4, TLN1, VCL, ACTG1, TTN | 74 | 232 | 8534 | 3.48E+00 | 9.13E-01 | 8.96E-02 | 7.75E-02 |
| GOTERM_BP_DIRECT | GO:0030335~positive regulation of cell migration | 7 | 4.93 | 1.43E-02 | ITGB1, GRN, HSPA5, CCL5, CTSH, AIF1, ACTG1 | 134 | 288 | 19478 | 3.53E+00 | 1.00E+00 | 3.60E-01 | 3.53E-01 |
| INTERPRO | IPR050208:MHC_class-I_related | 3 | 2.11 | 1.45E-02 | HLA-DPB1, HLA-DRB1, HLA-DQB1 | 138 | 28 | 20808 | 1.62E+01 | 9.99E-01 | 2.41E-01 | 2.32E-01 |
| GOTERM_BP_DIRECT | GO:0006915~apoptotic process | 11 | 7.75 | 1.46E-02 | SRGN, APP, LGALS1, GZMA, DDIT4, MDM2, PRF1, CTSH, PPIA, S100A9, S100A8 | 134 | 656 | 19478 | 2.44E+00 | 1.00E+00 | 3.60E-01 | 3.53E-01 |
| UP_KW_CELLULAR_COMPONENT | KW-0034~Amyloid | 3 | 2.11 | 1.49E-02 | CST3, APP, LYZ | 136 | 25 | 18049 | 1.59E+01 | 3.53E-01 | 4.80E-02 | 3.47E-02 |
| UP_KW_PTM | KW-0865~Zymogen | 7 | 4.93 | 1.52E-02 | ADAM19, GZMK, GZMA, CTSH, CTSW, AOAH, CTSS | 127 | 227 | 14316 | 3.48E+00 | 2.52E-01 | 6.07E-02 | 4.86E-02 |
| GOTERM_BP_DIRECT | GO:0019731~antibacterial humoral response | 4 | 2.82 | 1.52E-02 | APP, IGHA1, IGKV3-20, JCHAIN | 134 | 76 | 19478 | 7.65E+00 | 1.00E+00 | 3.68E-01 | 3.60E-01 |
| INTERPRO | IPR037055:MHC_I-like_Ag-recog_sf | 3 | 2.11 | 1.56E-02 | HLA-DPB1, HLA-DRB1, HLA-DQB1 | 138 | 29 | 20808 | 1.56E+01 | 9.99E-01 | 2.41E-01 | 2.32E-01 |
| INTERPRO | IPR011161:MHC_I-like_Ag-recog | 3 | 2.11 | 1.56E-02 | HLA-DPB1, HLA-DRB1, HLA-DQB1 | 138 | 29 | 20808 | 1.56E+01 | 9.99E-01 | 2.41E-01 | 2.32E-01 |
| GOTERM_CC_DIRECT | GO:0005793~endoplasmic reticulum-Golgi intermediate compartment | 4 | 2.82 | 1.62E-02 | LMAN1, HSPA5, GOLGB1, HMGB1 | 139 | 80 | 20795 | 7.48E+00 | 9.94E-01 | 1.11E-01 | 9.93E-02 |
| GOTERM_BP_DIRECT | GO:0032729~positive regulation of type II interferon production | 4 | 2.82 | 1.63E-02 | APP, HLA-DPB1, SLAMF1, HLA-DPA1 | 134 | 78 | 19478 | 7.45E+00 | 1.00E+00 | 3.71E-01 | 3.63E-01 |
| GOTERM_BP_DIRECT | GO:0071346~cellular response to type II interferon | 4 | 2.82 | 1.63E-02 | CCL5, AIF1, ACTG1, HLA-DPA1 | 134 | 78 | 19478 | 7.45E+00 | 1.00E+00 | 3.71E-01 | 3.63E-01 |
| GOTERM_BP_DIRECT | GO:0045087~innate immune response | 10 | 7.04 | 1.64E-02 | LGALS3, APP, SEC14L1, FCER1G, HMGB1, S100A9, S100A8, SLAMF1, JCHAIN, KLRG1 | 134 | 570 | 19478 | 2.55E+00 | 1.00E+00 | 3.71E-01 | 3.63E-01 |
| BIOCARTA | h_mhcPathway:Antigen Processing and Presentation | 3 | 2.11 | 1.64E-02 | CD74, HLA-DRA, HLA-DRB1 | 31 | 11 | 1622 | 1.43E+01 | 8.02E-01 | 8.04E-01 | 8.04E-01 |
| UP_KW_BIOLOGICAL_PROCESS | KW-0399~Innate immunity | 9 | 6.34 | 1.70E-02 | FCN1, LGALS3, SEC14L1, FCER1G, HMGB1, S100A9, S100A8, SLAMF1, KLRG1 | 89 | 431 | 11523 | 2.70E+00 | 5.45E-01 | 1.56E-01 | 1.49E-01 |
| GOTERM_CC_DIRECT | GO:0005768~endosome | 7 | 4.93 | 1.70E-02 | APP, GRN, ANXA2, STX7, HMGB1, CST7, CLINT1 | 139 | 308 | 20795 | 3.40E+00 | 9.96E-01 | 1.14E-01 | 1.02E-01 |
| INTERPRO | IPR040079:Glutathione_S-Trfase | 3 | 2.11 | 1.77E-02 | CLIC3, GSTP1, CLIC1 | 138 | 31 | 20808 | 1.46E+01 | 1.00E+00 | 2.41E-01 | 2.32E-01 |
| GOTERM_MF_DIRECT | GO:0051015~actin filament binding | 6 | 4.23 | 1.80E-02 | TPM4, TLN1, AIF1, VCL, MYO1F, TTN | 135 | 217 | 19253 | 3.94E+00 | 9.99E-01 | 2.70E-01 | 2.59E-01 |
| UP_SEQ_FEATURE | REGION:Interaction with PPIB | 2 | 1.41 | 1.95E-02 | CANX, CALR | 136 | 3 | 20675 | 1.01E+02 | 1.00E+00 | 7.19E-01 | 7.12E-01 |
| UP_SEQ_FEATURE | DOMAIN:E2 | 2 | 1.41 | 1.95E-02 | APP, APLP2 | 136 | 3 | 20675 | 1.01E+02 | 1.00E+00 | 7.19E-01 | 7.12E-01 |
| UP_SEQ_FEATURE | REGION:GFLD subdomain | 2 | 1.41 | 1.95E-02 | APP, APLP2 | 136 | 3 | 20675 | 1.01E+02 | 1.00E+00 | 7.19E-01 | 7.12E-01 |
| UP_SEQ_FEATURE | DOMAIN:E1 | 2 | 1.41 | 1.95E-02 | APP, APLP2 | 136 | 3 | 20675 | 1.01E+02 | 1.00E+00 | 7.19E-01 | 7.12E-01 |
| UP_SEQ_FEATURE | REGION:CuBD subdomain | 2 | 1.41 | 1.95E-02 | APP, APLP2 | 136 | 3 | 20675 | 1.01E+02 | 1.00E+00 | 7.19E-01 | 7.12E-01 |
| GOTERM_CC_DIRECT | GO:0030670~phagocytic vesicle membrane | 4 | 2.82 | 1.96E-02 | RAB11FIP1, HLA-DPB1, CALR, HLA-DQB1 | 139 | 86 | 20795 | 6.96E+00 | 9.98E-01 | 1.25E-01 | 1.12E-01 |
| INTERPRO | IPR019744:APP_CUBD_CS | 2 | 1.41 | 1.96E-02 | APP, APLP2 | 138 | 3 | 20808 | 1.01E+02 | 1.00E+00 | 2.41E-01 | 2.32E-01 |
| INTERPRO | IPR024329:Amyloid_glyco_E2_domain | 2 | 1.41 | 1.96E-02 | APP, APLP2 | 138 | 3 | 20808 | 1.01E+02 | 1.00E+00 | 2.41E-01 | 2.32E-01 |
| INTERPRO | IPR036669:Amyloid_Cu-bd_sf | 2 | 1.41 | 1.96E-02 | APP, APLP2 | 138 | 3 | 20808 | 1.01E+02 | 1.00E+00 | 2.41E-01 | 2.32E-01 |
| INTERPRO | IPR008155:Amyloid_glyco | 2 | 1.41 | 1.96E-02 | APP, APLP2 | 138 | 3 | 20808 | 1.01E+02 | 1.00E+00 | 2.41E-01 | 2.32E-01 |
| INTERPRO | IPR036176:E2_sf | 2 | 1.41 | 1.96E-02 | APP, APLP2 | 138 | 3 | 20808 | 1.01E+02 | 1.00E+00 | 2.41E-01 | 2.32E-01 |
| INTERPRO | IPR019543:APP_amyloid_C | 2 | 1.41 | 1.96E-02 | APP, APLP2 | 138 | 3 | 20808 | 1.01E+02 | 1.00E+00 | 2.41E-01 | 2.32E-01 |
| INTERPRO | IPR008154:Amyloid_glyco_extra | 2 | 1.41 | 1.96E-02 | APP, APLP2 | 138 | 3 | 20808 | 1.01E+02 | 1.00E+00 | 2.41E-01 | 2.32E-01 |
| INTERPRO | IPR015849:Amyloid_glyco_heparin-bd | 2 | 1.41 | 1.96E-02 | APP, APLP2 | 138 | 3 | 20808 | 1.01E+02 | 1.00E+00 | 2.41E-01 | 2.32E-01 |
| INTERPRO | IPR036454:Amyloid_glyco_heparin-bd_sf | 2 | 1.41 | 1.96E-02 | APP, APLP2 | 138 | 3 | 20808 | 1.01E+02 | 1.00E+00 | 2.41E-01 | 2.32E-01 |
| INTERPRO | IPR011178:Amyloid_glyco_Cu-bd | 2 | 1.41 | 1.96E-02 | APP, APLP2 | 138 | 3 | 20808 | 1.01E+02 | 1.00E+00 | 2.41E-01 | 2.32E-01 |
| INTERPRO | IPR019745:Amyloid_glyco_intracell_CS | 2 | 1.41 | 1.96E-02 | APP, APLP2 | 138 | 3 | 20808 | 1.01E+02 | 1.00E+00 | 2.41E-01 | 2.32E-01 |
| GOTERM_MF_DIRECT | GO:0031625~ubiquitin protein ligase binding | 7 | 4.93 | 1.97E-02 | HSPA5, MDM2, CALR, TNFRSF1B, VCL, ACTG1, UBE2J1 | 135 | 304 | 19253 | 3.28E+00 | 9.99E-01 | 2.84E-01 | 2.73E-01 |
| KEGG_PATHWAY | hsa05131:Shigellosis | 7 | 4.93 | 1.97E-02 | ITGB1, CCL5, MDM2, PFN1, TLN1, VCL, ACTG1 | 74 | 250 | 8534 | 3.23E+00 | 9.67E-01 | 1.18E-01 | 1.03E-01 |
| GOTERM_CC_DIRECT | GO:0071756~pentameric IgM immunoglobulin complex | 2 | 1.41 | 1.98E-02 | IGKV3-20, JCHAIN | 139 | 3 | 20795 | 9.97E+01 | 9.98E-01 | 1.25E-01 | 1.12E-01 |
| GOTERM_CC_DIRECT | GO:0071752~secretory dimeric IgA immunoglobulin complex | 2 | 1.41 | 1.98E-02 | IGHA1, JCHAIN | 139 | 3 | 20795 | 9.97E+01 | 9.98E-01 | 1.25E-01 | 1.12E-01 |
| GOTERM_BP_DIRECT | GO:0034394~protein localization to cell surface | 3 | 2.11 | 2.01E-02 | FCN1, MESD, VCL | 134 | 32 | 19478 | 1.36E+01 | 1.00E+00 | 4.34E-01 | 4.25E-01 |
| KEGG_PATHWAY | hsa05132:Salmonella infection | 7 | 4.93 | 2.01E-02 | AHNAK, ANXA2, FOS, PFN1, HSP90B1, ACTG1, S100A10 | 74 | 251 | 8534 | 3.22E+00 | 9.69E-01 | 1.18E-01 | 1.03E-01 |
| GOTERM_BP_DIRECT | GO:0002469~myeloid dendritic cell antigen processing and presentation | 2 | 1.41 | 2.03E-02 | HLA-DRA, HLA-DRB1 | 134 | 3 | 19478 | 9.69E+01 | 1.00E+00 | 4.34E-01 | 4.25E-01 |
| GOTERM_BP_DIRECT | GO:0002577~regulation of antigen processing and presentation | 2 | 1.41 | 2.03E-02 | CST7, CTSS | 134 | 3 | 19478 | 9.69E+01 | 1.00E+00 | 4.34E-01 | 4.25E-01 |
| INTERPRO | IPR013320:ConA-like_dom_sf | 6 | 4.23 | 2.04E-02 | NELL2, LGALS3, LMAN1, LGALS1, CANX, CALR | 138 | 237 | 20808 | 3.82E+00 | 1.00E+00 | 2.45E-01 | 2.36E-01 |
| UP_KW_DISEASE | KW-1008~Amyloidosis | 3 | 2.11 | 2.07E-02 | CST3, APP, LYZ | 34 | 33 | 4859 | 1.30E+01 | 4.66E-01 | 6.21E-01 | 6.21E-01 |
| INTERPRO | IPR010987:Glutathione-S-Trfase_C-like | 3 | 2.11 | 2.22E-02 | CLIC3, GSTP1, CLIC1 | 138 | 35 | 20808 | 1.29E+01 | 1.00E+00 | 2.60E-01 | 2.50E-01 |
| GOTERM_BP_DIRECT | GO:0035633~maintenance of blood-brain barrier | 3 | 2.11 | 2.25E-02 | ITGB1, VCL, ACTG1 | 134 | 34 | 19478 | 1.28E+01 | 1.00E+00 | 4.71E-01 | 4.61E-01 |
| GOTERM_CC_DIRECT | GO:0000139~Golgi membrane | 11 | 7.75 | 2.32E-02 | CD74, LMAN1, HLA-DRB5, CUX1, GOLGB1, LST1, HLA-DPB1, HLA-DRA, HLA-DRB1, HLA-DPA1, HLA-DQB1 | 139 | 727 | 20795 | 2.26E+00 | 9.99E-01 | 1.44E-01 | 1.28E-01 |
| UP_SEQ_FEATURE | DOMAIN:GST C-terminal | 3 | 2.11 | 2.43E-02 | CLIC3, GSTP1, CLIC1 | 136 | 37 | 20675 | 1.23E+01 | 1.00E+00 | 8.50E-01 | 8.41E-01 |
| GOTERM_BP_DIRECT | GO:0050832~defense response to fungus | 3 | 2.11 | 2.51E-02 | GNLY, S100A9, S100A8 | 134 | 36 | 19478 | 1.21E+01 | 1.00E+00 | 4.74E-01 | 4.64E-01 |
| GOTERM_BP_DIRECT | GO:0043066~negative regulation of apoptotic process | 9 | 6.34 | 2.51E-02 | DNAJC3, CD74, CCND2, HSPA5, GSTP1, MDM2, CTSH, HSP90B1, MTDH | 134 | 515 | 19478 | 2.54E+00 | 1.00E+00 | 4.74E-01 | 4.64E-01 |
| SMART | SM00006:A4_EXTRA | 2 | 1.41 | 2.58E-02 | APP, APLP2 | 94 | 3 | 10706 | 7.59E+01 | 9.08E-01 | 2.35E-01 | 2.20E-01 |
| UP_SEQ_FEATURE | TRANSMEM:Helical; Name=I | 2 | 1.41 | 2.59E-02 | MT-CO1, MT-CO2 | 136 | 4 | 20675 | 7.60E+01 | 1.00E+00 | 8.50E-01 | 8.41E-01 |
| UP_SEQ_FEATURE | REGION:Required for insertion into the membrane | 2 | 1.41 | 2.59E-02 | CLIC3, CLIC1 | 136 | 4 | 20675 | 7.60E+01 | 1.00E+00 | 8.50E-01 | 8.41E-01 |
| UP_SEQ_FEATURE | TRANSMEM:Helical; Name=II | 2 | 1.41 | 2.59E-02 | MT-CO1, MT-CO2 | 136 | 4 | 20675 | 7.60E+01 | 1.00E+00 | 8.50E-01 | 8.41E-01 |
| INTERPRO | IPR008138:SapB_2 | 2 | 1.41 | 2.61E-02 | GNLY, PSAP | 138 | 4 | 20808 | 7.54E+01 | 1.00E+00 | 2.78E-01 | 2.68E-01 |
| INTERPRO | IPR018124:Calret/calnex_CS | 2 | 1.41 | 2.61E-02 | CANX, CALR | 138 | 4 | 20808 | 7.54E+01 | 1.00E+00 | 2.78E-01 | 2.68E-01 |
| INTERPRO | IPR009033:Calreticulin/calnexin_P_dom_sf | 2 | 1.41 | 2.61E-02 | CANX, CALR | 138 | 4 | 20808 | 7.54E+01 | 1.00E+00 | 2.78E-01 | 2.68E-01 |
| INTERPRO | IPR001580:Calret/calnex | 2 | 1.41 | 2.61E-02 | CANX, CALR | 138 | 4 | 20808 | 7.54E+01 | 1.00E+00 | 2.78E-01 | 2.68E-01 |
| GOTERM_CC_DIRECT | GO:0005789~endoplasmic reticulum membrane | 15 | 10.56 | 2.61E-02 | HSPA5, SSR4, RPN1, TXNDC11, RRBP1, UBE2J1, MTDH, HSP90B1, ERN1, SEC61A1, SPCS3, LMAN1, DNAJC1, CANX, CALR | 139 | 1186 | 20795 | 1.89E+00 | 1.00E+00 | 1.54E-01 | 1.37E-01 |
| KEGG_PATHWAY | hsa04115:p53 signaling pathway | 4 | 2.82 | 2.61E-02 | CDK6, CCND2, CD82, MDM2 | 74 | 75 | 8534 | 6.15E+00 | 9.89E-01 | 1.49E-01 | 1.29E-01 |
| GOTERM_CC_DIRECT | GO:0070820~tertiary granule | 2 | 1.41 | 2.63E-02 | STX7, STXBP2 | 139 | 4 | 20795 | 7.48E+01 | 1.00E+00 | 1.54E-01 | 1.37E-01 |
| GOTERM_CC_DIRECT | GO:0120281~autolysosome membrane | 2 | 1.41 | 2.63E-02 | HLA-DRA, HLA-DRB1 | 139 | 4 | 20795 | 7.48E+01 | 1.00E+00 | 1.54E-01 | 1.37E-01 |
| GOTERM_BP_DIRECT | GO:0050821~protein stabilization | 6 | 4.23 | 2.70E-02 | CD74, GRN, TYROBP, CALR, PFN1, PPIB | 134 | 246 | 19478 | 3.55E+00 | 1.00E+00 | 4.74E-01 | 4.64E-01 |
| GOTERM_BP_DIRECT | GO:0150003~regulation of spontaneous synaptic transmission | 2 | 1.41 | 2.70E-02 | ITGB1, APP | 134 | 4 | 19478 | 7.27E+01 | 1.00E+00 | 4.74E-01 | 4.64E-01 |
| GOTERM_BP_DIRECT | GO:2000516~positive regulation of CD4-positive, alpha-beta T cell activation | 2 | 1.41 | 2.70E-02 | HLA-DRA, HLA-DRB1 | 134 | 4 | 19478 | 7.27E+01 | 1.00E+00 | 4.74E-01 | 4.64E-01 |
| GOTERM_BP_DIRECT | GO:1902685~positive regulation of receptor localization to synapse | 2 | 1.41 | 2.70E-02 | TYROBP, STX7 | 134 | 4 | 19478 | 7.27E+01 | 1.00E+00 | 4.74E-01 | 4.64E-01 |
| GOTERM_BP_DIRECT | GO:0002265~astrocyte activation involved in immune response | 2 | 1.41 | 2.70E-02 | APP, GRN | 134 | 4 | 19478 | 7.27E+01 | 1.00E+00 | 4.74E-01 | 4.64E-01 |
| GOTERM_BP_DIRECT | GO:0045622~regulation of T-helper cell differentiation | 2 | 1.41 | 2.70E-02 | HLA-DRA, HLA-DRB1 | 134 | 4 | 19478 | 7.27E+01 | 1.00E+00 | 4.74E-01 | 4.64E-01 |
| GOTERM_BP_DIRECT | GO:1903901~negative regulation of viral life cycle | 2 | 1.41 | 2.70E-02 | IL32, PPIA | 134 | 4 | 19478 | 7.27E+01 | 1.00E+00 | 4.74E-01 | 4.64E-01 |
| GOTERM_BP_DIRECT | GO:0071677~positive regulation of mononuclear cell migration | 2 | 1.41 | 2.70E-02 | LGALS3, AIF1 | 134 | 4 | 19478 | 7.27E+01 | 1.00E+00 | 4.74E-01 | 4.64E-01 |
| GOTERM_CC_DIRECT | GO:0043202~lysosomal lumen | 4 | 2.82 | 2.75E-02 | CD74, VCAN, PSAP, CTSS | 139 | 98 | 20795 | 6.11E+00 | 1.00E+00 | 1.58E-01 | 1.41E-01 |
| GOTERM_MF_DIRECT | GO:0035662~Toll-like receptor 4 binding | 2 | 1.41 | 2.76E-02 | S100A9, S100A8 | 135 | 4 | 19253 | 7.13E+01 | 1.00E+00 | 3.81E-01 | 3.67E-01 |
| GOTERM_BP_DIRECT | GO:0001916~positive regulation of T cell mediated cytotoxicity | 3 | 2.11 | 2.77E-02 | STX7, HLA-DRA, HLA-DRB1 | 134 | 38 | 19478 | 1.15E+01 | 1.00E+00 | 4.78E-01 | 4.69E-01 |
| GOTERM_CC_DIRECT | GO:0001726~ruffle | 4 | 2.82 | 2.83E-02 | ITGB1, CDK6, TLN1, AIF1 | 139 | 99 | 20795 | 6.04E+00 | 1.00E+00 | 1.59E-01 | 1.42E-01 |
| KEGG_PATHWAY | hsa03060:Protein export | 3 | 2.11 | 2.86E-02 | SEC61A1, SPCS3, HSPA5 | 74 | 31 | 8534 | 1.12E+01 | 9.93E-01 | 1.58E-01 | 1.37E-01 |
| GOTERM_CC_DIRECT | GO:0012505~endomembrane system | 7 | 4.93 | 2.91E-02 | LMAN1, APP, DNAJC1, RAB11FIP1, SSR4, APLP2, STX7 | 139 | 349 | 20795 | 3.00E+00 | 1.00E+00 | 1.61E-01 | 1.44E-01 |
| UP_SEQ_FEATURE | DISULFID:Interchain | 5 | 3.52 | 3.00E-02 | LGALS3, LMAN1, TYROBP, FCER1G, HSP90B1 | 136 | 179 | 20675 | 4.25E+00 | 1.00E+00 | 9.53E-01 | 9.43E-01 |
| KEGG_PATHWAY | hsa05135:Yersinia infection | 5 | 3.52 | 3.02E-02 | ITGB1, CD8B, CD8A, FOS, ACTG1 | 74 | 138 | 8534 | 4.18E+00 | 9.95E-01 | 1.61E-01 | 1.40E-01 |
| UP_KW_MOLECULAR_FUNCTION | KW-0389~IgE-binding protein | 2 | 1.41 | 3.06E-02 | LGALS3, FCER1G | 75 | 5 | 11952 | 6.37E+01 | 8.24E-01 | 6.55E-01 | 6.55E-01 |
| INTERPRO | IPR036282:Glutathione-S-Trfase_C_sf | 3 | 2.11 | 3.12E-02 | CLIC3, GSTP1, CLIC1 | 138 | 42 | 20808 | 1.08E+01 | 1.00E+00 | 3.05E-01 | 2.94E-01 |
| UP_SEQ_FEATURE | DOMAIN:MHC class II alpha chain N-terminal | 2 | 1.41 | 3.22E-02 | HLA-DRA, HLA-DPA1 | 136 | 5 | 20675 | 6.08E+01 | 1.00E+00 | 9.53E-01 | 9.43E-01 |
| UP_SEQ_FEATURE | REGION:4 X approximate repeats | 2 | 1.41 | 3.22E-02 | CANX, CALR | 136 | 5 | 20675 | 6.08E+01 | 1.00E+00 | 9.53E-01 | 9.43E-01 |
| UP_SEQ_FEATURE | DOMAIN:Ferritin-like diiron | 2 | 1.41 | 3.22E-02 | FTH1, FTL | 136 | 5 | 20675 | 6.08E+01 | 1.00E+00 | 9.53E-01 | 9.43E-01 |
| INTERPRO | IPR009040:Ferritin-like_diiron | 2 | 1.41 | 3.25E-02 | FTH1, FTL | 138 | 5 | 20808 | 6.03E+01 | 1.00E+00 | 3.05E-01 | 2.94E-01 |
| INTERPRO | IPR008331:Ferritin_DPS_dom | 2 | 1.41 | 3.25E-02 | FTH1, FTL | 138 | 5 | 20808 | 6.03E+01 | 1.00E+00 | 3.05E-01 | 2.94E-01 |
| INTERPRO | IPR001519:Ferritin | 2 | 1.41 | 3.25E-02 | FTH1, FTL | 138 | 5 | 20808 | 6.03E+01 | 1.00E+00 | 3.05E-01 | 2.94E-01 |
| INTERPRO | IPR014034:Ferritin_CS | 2 | 1.41 | 3.25E-02 | FTH1, FTL | 138 | 5 | 20808 | 6.03E+01 | 1.00E+00 | 3.05E-01 | 2.94E-01 |
| INTERPRO | IPR012347:Ferritin-like | 2 | 1.41 | 3.25E-02 | FTH1, FTL | 138 | 5 | 20808 | 6.03E+01 | 1.00E+00 | 3.05E-01 | 2.94E-01 |
| GOTERM_CC_DIRECT | GO:0042383~sarcolemma | 4 | 2.82 | 3.36E-02 | ITGB1, AHNAK, ANXA2, VCL | 139 | 106 | 20795 | 5.65E+00 | 1.00E+00 | 1.83E-01 | 1.64E-01 |
| GOTERM_BP_DIRECT | GO:0032831~positive regulation of CD4-positive, CD25-positive, alpha-beta regulatory T cell differentiation | 2 | 1.41 | 3.37E-02 | HLA-DRA, HLA-DRB1 | 134 | 5 | 19478 | 5.81E+01 | 1.00E+00 | 5.46E-01 | 5.35E-01 |
| GOTERM_BP_DIRECT | GO:0001765~membrane raft assembly | 2 | 1.41 | 3.37E-02 | ANXA2, S100A10 | 134 | 5 | 19478 | 5.81E+01 | 1.00E+00 | 5.46E-01 | 5.35E-01 |
| GOTERM_BP_DIRECT | GO:0002282~microglial cell activation involved in immune response | 2 | 1.41 | 3.37E-02 | GRN, TYROBP | 134 | 5 | 19478 | 5.81E+01 | 1.00E+00 | 5.46E-01 | 5.35E-01 |
| GOTERM_BP_DIRECT | GO:0051208~sequestering of calcium ion | 2 | 1.41 | 3.37E-02 | CALR, HSP90B1 | 134 | 5 | 19478 | 5.81E+01 | 1.00E+00 | 5.46E-01 | 5.35E-01 |
| GOTERM_MF_DIRECT | GO:0097100~supercoiled DNA binding | 2 | 1.41 | 3.43E-02 | HMGB1, TOP1 | 135 | 5 | 19253 | 5.70E+01 | 1.00E+00 | 4.26E-01 | 4.09E-01 |
| GOTERM_MF_DIRECT | GO:1905172~RISC complex binding | 2 | 1.41 | 3.43E-02 | NEAT1, SND1 | 135 | 5 | 19253 | 5.70E+01 | 1.00E+00 | 4.26E-01 | 4.09E-01 |
| GOTERM_MF_DIRECT | GO:0019863~IgE binding | 2 | 1.41 | 3.43E-02 | LGALS3, FCER1G | 135 | 5 | 19253 | 5.70E+01 | 1.00E+00 | 4.26E-01 | 4.09E-01 |
| UP_KW_MOLECULAR_FUNCTION | KW-0929~Antimicrobial | 4 | 2.82 | 3.51E-02 | GNLY, LYZ, S100A9, S100A8 | 75 | 116 | 11952 | 5.50E+00 | 8.65E-01 | 6.55E-01 | 6.55E-01 |
| UP_KW_BIOLOGICAL_PROCESS | KW-0053~Apoptosis | 10 | 7.04 | 3.54E-02 | SRGN, ERN1, APP, LGALS1, DDIT4, MDM2, TNFRSF1B, PPIA, S100A9, S100A8 | 89 | 589 | 11523 | 2.20E+00 | 8.09E-01 | 2.59E-01 | 2.47E-01 |
| GOTERM_CC_DIRECT | GO:0031965~nuclear membrane | 6 | 4.23 | 3.61E-02 | DNAJC1, CCND2, CANX, CLIC1, TMPO, MTDH | 139 | 274 | 20795 | 3.28E+00 | 1.00E+00 | 1.94E-01 | 1.73E-01 |
| GOTERM_MF_DIRECT | GO:0008234~cysteine-type peptidase activity | 3 | 2.11 | 3.76E-02 | CTSH, CTSW, CTSS | 135 | 44 | 19253 | 9.72E+00 | 1.00E+00 | 4.51E-01 | 4.34E-01 |
| UP_SEQ_FEATURE | MOTIF:G-site | 2 | 1.41 | 3.85E-02 | CLIC3, CLIC1 | 136 | 6 | 20675 | 5.07E+01 | 1.00E+00 | 1.00E+00 | 9.90E-01 |
| KEGG_PATHWAY | hsa05014:Amyotrophic lateral sclerosis | 8 | 5.63 | 3.86E-02 | ERN1, NUP214, HSPA5, MT-CO1, MT-CO2, PFN1, TNFRSF1B, ACTG1 | 74 | 371 | 8534 | 2.49E+00 | 9.99E-01 | 2.00E-01 | 1.73E-01 |
| INTERPRO | IPR053823:CLIC_N | 2 | 1.41 | 3.89E-02 | CLIC3, CLIC1 | 138 | 6 | 20808 | 5.03E+01 | 1.00E+00 | 3.51E-01 | 3.39E-01 |
| INTERPRO | IPR002946:CLIC | 2 | 1.41 | 3.89E-02 | CLIC3, CLIC1 | 138 | 6 | 20808 | 5.03E+01 | 1.00E+00 | 3.51E-01 | 3.39E-01 |
| GOTERM_CC_DIRECT | GO:0005784~Sec61 translocon complex | 2 | 1.41 | 3.92E-02 | SEC61A1, SSR4 | 139 | 6 | 20795 | 4.99E+01 | 1.00E+00 | 2.06E-01 | 1.84E-01 |
| GOTERM_BP_DIRECT | GO:0045214~sarcomere organization | 3 | 2.11 | 3.94E-02 | ITGB1, ACTG1, TTN | 134 | 46 | 19478 | 9.48E+00 | 1.00E+00 | 6.21E-01 | 6.08E-01 |
| GOTERM_BP_DIRECT | GO:0032689~negative regulation of type II interferon production | 3 | 2.11 | 3.94E-02 | HMGB1, HLA-DRB1, SLAMF1 | 134 | 46 | 19478 | 9.48E+00 | 1.00E+00 | 6.21E-01 | 6.08E-01 |
| GOTERM_BP_DIRECT | GO:0000413~protein peptidyl-prolyl isomerization | 2 | 1.41 | 4.03E-02 | PPIB, PPIA | 134 | 6 | 19478 | 4.85E+01 | 1.00E+00 | 6.26E-01 | 6.13E-01 |
| GOTERM_BP_DIRECT | GO:0032760~positive regulation of tumor necrosis factor production | 4 | 2.82 | 4.14E-02 | SPN, APP, TYROBP, HMGB1 | 134 | 112 | 19478 | 5.19E+00 | 1.00E+00 | 6.34E-01 | 6.21E-01 |
| UP_KW_BIOLOGICAL_PROCESS | KW-0145~Chemotaxis | 4 | 2.82 | 4.30E-02 | CCL5, HMGB1, S100A9, S100A8 | 89 | 102 | 11523 | 5.08E+00 | 8.68E-01 | 2.59E-01 | 2.47E-01 |
| UP_SEQ_FEATURE | REPEAT:38 | 2 | 1.41 | 4.48E-02 | NUP214, RRBP1 | 136 | 7 | 20675 | 4.34E+01 | 1.00E+00 | 1.00E+00 | 9.90E-01 |
| UP_SEQ_FEATURE | CARBOHYD:O-linked (GalNAc...) serine; partial | 2 | 1.41 | 4.48E-02 | APP, CCL5 | 136 | 7 | 20675 | 4.34E+01 | 1.00E+00 | 1.00E+00 | 9.90E-01 |
| UP_SEQ_FEATURE | REPEAT:37 | 2 | 1.41 | 4.48E-02 | NUP214, RRBP1 | 136 | 7 | 20675 | 4.34E+01 | 1.00E+00 | 1.00E+00 | 9.90E-01 |
| UP_KW_BIOLOGICAL_PROCESS | KW-0409~Iron storage | 2 | 1.41 | 4.50E-02 | FTH1, FTL | 89 | 6 | 11523 | 4.32E+01 | 8.80E-01 | 2.59E-01 | 2.47E-01 |
| INTERPRO | IPR036723:Alpha-catenin/vinculin-like_sf | 2 | 1.41 | 4.52E-02 | TLN1, VCL | 138 | 7 | 20808 | 4.31E+01 | 1.00E+00 | 4.01E-01 | 3.87E-01 |
| GOTERM_CC_DIRECT | GO:0097433~dense body | 2 | 1.41 | 4.55E-02 | SND1, ACTG1 | 139 | 7 | 20795 | 4.27E+01 | 1.00E+00 | 2.32E-01 | 2.07E-01 |
| GOTERM_CC_DIRECT | GO:0071745~IgA immunoglobulin complex | 2 | 1.41 | 4.55E-02 | IGKC, IGHA1 | 139 | 7 | 20795 | 4.27E+01 | 1.00E+00 | 2.32E-01 | 2.07E-01 |
| GOTERM_BP_DIRECT | GO:0035425~autocrine signaling | 2 | 1.41 | 4.68E-02 | S100A9, S100A8 | 134 | 7 | 19478 | 4.15E+01 | 1.00E+00 | 6.97E-01 | 6.83E-01 |
| GOTERM_BP_DIRECT | GO:0060267~positive regulation of respiratory burst | 2 | 1.41 | 4.68E-02 | IGHA1, JCHAIN | 134 | 7 | 19478 | 4.15E+01 | 1.00E+00 | 6.97E-01 | 6.83E-01 |
| KEGG_PATHWAY | hsa04657:IL-17 signaling pathway | 4 | 2.82 | 4.75E-02 | FOS, S100A9, S100A8, HSP90B1 | 74 | 95 | 8534 | 4.86E+00 | 1.00E+00 | 2.39E-01 | 2.07E-01 |
| GOTERM_MF_DIRECT | GO:0050544~arachidonate binding | 2 | 1.41 | 4.77E-02 | S100A9, S100A8 | 135 | 7 | 19253 | 4.07E+01 | 1.00E+00 | 5.54E-01 | 5.33E-01 |
| UP_KW_MOLECULAR_FUNCTION | KW-0646~Protease inhibitor | 4 | 2.82 | 4.84E-02 | CST3, APP, APLP2, CST7 | 75 | 132 | 11952 | 4.83E+00 | 9.38E-01 | 6.78E-01 | 6.78E-01 |
| GOTERM_BP_DIRECT | GO:0051092~positive regulation of NF-kappaB transcription factor activity | 4 | 2.82 | 4.91E-02 | PPIA, S100A9, S100A8, MTDH | 134 | 120 | 19478 | 4.85E+00 | 1.00E+00 | 7.03E-01 | 6.89E-01 |
| KEGG_PATHWAY | hsa04216:Ferroptosis | 3 | 2.11 | 4.99E-02 | FTH1, SAT1, FTL | 74 | 42 | 8534 | 8.24E+00 | 1.00E+00 | 2.44E-01 | 2.11E-01 |
| UP_SEQ_FEATURE | REPEAT:41 | 2 | 1.41 | 5.11E-02 | NUP214, RRBP1 | 136 | 8 | 20675 | 3.80E+01 | 1.00E+00 | 1.00E+00 | 9.90E-01 |
| UP_SEQ_FEATURE | REPEAT:40 | 2 | 1.41 | 5.11E-02 | NUP214, RRBP1 | 136 | 8 | 20675 | 3.80E+01 | 1.00E+00 | 1.00E+00 | 9.90E-01 |
| BIOCARTA | h_th1th2Pathway:Th1/Th2 Differentiation | 3 | 2.11 | 5.11E-02 | IL4R, HLA-DRA, HLA-DRB1 | 31 | 20 | 1622 | 7.85E+00 | 9.94E-01 | 1.00E+00 | 1.00E+00 |
| GOTERM_BP_DIRECT | GO:0043410~positive regulation of MAPK cascade | 5 | 3.52 | 5.14E-02 | CD74, APP, HMGB1, PPIA, HLA-DRB1 | 134 | 204 | 19478 | 3.56E+00 | 1.00E+00 | 7.03E-01 | 6.89E-01 |
| INTERPRO | IPR050373:Fibrinogen_C-term_domain | 2 | 1.41 | 5.15E-02 | FCN1, FGL2 | 138 | 8 | 20808 | 3.77E+01 | 1.00E+00 | 4.40E-01 | 4.25E-01 |
| INTERPRO | IPR010579:MHC_I_a_C | 2 | 1.41 | 5.15E-02 | HLA-DPB1, HLA-DQB1 | 138 | 8 | 20808 | 3.77E+01 | 1.00E+00 | 4.40E-01 | 4.25E-01 |
| UP_SEQ_FEATURE | CARBOHYD:O-linked (Xyl...) (chondroitin sulfate) serine | 3 | 2.11 | 5.18E-02 | CD74, VCAN, APLP2 | 136 | 56 | 20675 | 8.14E+00 | 1.00E+00 | 1.00E+00 | 9.90E-01 |
| KEGG_PATHWAY | hsa05410:Hypertrophic cardiomyopathy | 4 | 2.82 | 5.26E-02 | ITGB1, TPM4, ACTG1, TTN | 74 | 99 | 8534 | 4.66E+00 | 1.00E+00 | 2.50E-01 | 2.16E-01 |
| GOTERM_BP_DIRECT | GO:2000510~positive regulation of dendritic cell chemotaxis | 2 | 1.41 | 5.33E-02 | CALR, SLAMF1 | 134 | 8 | 19478 | 3.63E+01 | 1.00E+00 | 7.03E-01 | 6.89E-01 |
| GOTERM_BP_DIRECT | GO:1903979~negative regulation of microglial cell activation | 2 | 1.41 | 5.33E-02 | GRN, CST7 | 134 | 8 | 19478 | 3.63E+01 | 1.00E+00 | 7.03E-01 | 6.89E-01 |
| GOTERM_BP_DIRECT | GO:0031204~post-translational protein targeting to membrane, translocation | 2 | 1.41 | 5.33E-02 | SEC61A1, HSPA5 | 134 | 8 | 19478 | 3.63E+01 | 1.00E+00 | 7.03E-01 | 6.89E-01 |
| GOTERM_BP_DIRECT | GO:0010756~positive regulation of plasminogen activation | 2 | 1.41 | 5.33E-02 | ANXA2, S100A10 | 134 | 8 | 19478 | 3.63E+01 | 1.00E+00 | 7.03E-01 | 6.89E-01 |
| GOTERM_BP_DIRECT | GO:0036498~IRE1-mediated unfolded protein response | 2 | 1.41 | 5.33E-02 | ERN1, HSPA5 | 134 | 8 | 19478 | 3.63E+01 | 1.00E+00 | 7.03E-01 | 6.89E-01 |
| GOTERM_BP_DIRECT | GO:0002291~T cell activation via T cell receptor contact with antigen bound to MHC molecule on antigen presenting cell | 2 | 1.41 | 5.33E-02 | TYROBP, FGL2 | 134 | 8 | 19478 | 3.63E+01 | 1.00E+00 | 7.03E-01 | 6.89E-01 |
| GOTERM_BP_DIRECT | GO:0010466~negative regulation of peptidase activity | 2 | 1.41 | 5.33E-02 | CST3, CST7 | 134 | 8 | 19478 | 3.63E+01 | 1.00E+00 | 7.03E-01 | 6.89E-01 |
| GOTERM_BP_DIRECT | GO:0070374~positive regulation of ERK1 and ERK2 cascade | 5 | 3.52 | 5.37E-02 | CD74, APP, HMGB1, HLA-DRB1, SLAMF1 | 134 | 207 | 19478 | 3.51E+00 | 1.00E+00 | 7.03E-01 | 6.89E-01 |
| GOTERM_MF_DIRECT | GO:0042609~CD4 receptor binding | 2 | 1.41 | 5.44E-02 | CD74, HLA-DRB1 | 135 | 8 | 19253 | 3.57E+01 | 1.00E+00 | 6.11E-01 | 5.88E-01 |
| GOTERM_CC_DIRECT | GO:0043235~receptor complex | 5 | 3.52 | 5.68E-02 | ITGB1, APP, IL4R, CD8B, CD8A | 139 | 217 | 20795 | 3.45E+00 | 1.00E+00 | 2.85E-01 | 2.54E-01 |
| UP_SEQ_FEATURE | REPEAT:35 | 2 | 1.41 | 5.73E-02 | NUP214, RRBP1 | 136 | 9 | 20675 | 3.38E+01 | 1.00E+00 | 1.00E+00 | 9.90E-01 |
| UP_SEQ_FEATURE | SITE:Reactive site | 2 | 1.41 | 5.73E-02 | CST3, CST7 | 136 | 9 | 20675 | 3.38E+01 | 1.00E+00 | 1.00E+00 | 9.90E-01 |
| INTERPRO | IPR009078:Ferritin-like_SF | 2 | 1.41 | 5.77E-02 | FTH1, FTL | 138 | 9 | 20808 | 3.35E+01 | 1.00E+00 | 4.77E-01 | 4.60E-01 |
| INTERPRO | IPR036249:Thioredoxin-like_sf | 4 | 2.82 | 5.77E-02 | CLIC3, GSTP1, TXNDC11, CLIC1 | 138 | 133 | 20808 | 4.53E+00 | 1.00E+00 | 4.77E-01 | 4.60E-01 |
| UP_KW_CELLULAR_COMPONENT | KW-0968~Cytoplasmic vesicle | 11 | 7.75 | 5.78E-02 | APP, RAB11FIP1, CD82, FTH1, HLA-DRA, AOAH, CALR, CLINT1, CTSS, HLA-DRB1, FTL | 136 | 757 | 18049 | 1.93E+00 | 8.22E-01 | 1.68E-01 | 1.21E-01 |
| GOTERM_BP_DIRECT | GO:0006935~chemotaxis | 4 | 2.82 | 5.86E-02 | SPN, CCL5, HMGB1, CCR4 | 134 | 129 | 19478 | 4.51E+00 | 1.00E+00 | 7.47E-01 | 7.32E-01 |
| UP_KW_BIOLOGICAL_PROCESS | KW-0395~Inflammatory response | 5 | 3.52 | 5.92E-02 | CCL5, NKG7, HMGB1, S100A9, S100A8 | 89 | 192 | 11523 | 3.37E+00 | 9.40E-01 | 3.03E-01 | 2.89E-01 |
| UP_SEQ_FEATURE | DOMAIN:EGF-like | 5 | 3.52 | 5.94E-02 | NELL2, ADAM19, ITGB1, VCAN, PRF1 | 136 | 224 | 20675 | 3.39E+00 | 1.00E+00 | 1.00E+00 | 9.90E-01 |
| GOTERM_BP_DIRECT | GO:0032873~negative regulation of stress-activated MAPK cascade | 2 | 1.41 | 5.98E-02 | GSTP1, PPIA | 134 | 9 | 19478 | 3.23E+01 | 1.00E+00 | 7.47E-01 | 7.32E-01 |
| GOTERM_BP_DIRECT | GO:0002283~neutrophil activation involved in immune response | 2 | 1.41 | 5.98E-02 | TYROBP, FCER1G | 134 | 9 | 19478 | 3.23E+01 | 1.00E+00 | 7.47E-01 | 7.32E-01 |
| GOTERM_BP_DIRECT | GO:0043382~positive regulation of memory T cell differentiation | 2 | 1.41 | 5.98E-02 | HLA-DRA, HLA-DRB1 | 134 | 9 | 19478 | 3.23E+01 | 1.00E+00 | 7.47E-01 | 7.32E-01 |
| UP_SEQ_FEATURE | REPEAT:3 | 5 | 3.52 | 6.02E-02 | SRGN, NUP214, LGALS3, RRBP1, VCL | 136 | 225 | 20675 | 3.38E+00 | 1.00E+00 | 1.00E+00 | 9.90E-01 |
| KEGG_PATHWAY | hsa05414:Dilated cardiomyopathy | 4 | 2.82 | 6.07E-02 | ITGB1, TPM4, ACTG1, TTN | 74 | 105 | 8534 | 4.39E+00 | 1.00E+00 | 2.80E-01 | 2.43E-01 |
| GOTERM_BP_DIRECT | GO:0050853~B cell receptor signaling pathway | 3 | 2.11 | 6.15E-02 | IGKC, MNDA, IGHA1 | 134 | 59 | 19478 | 7.39E+00 | 1.00E+00 | 7.60E-01 | 7.45E-01 |
| UP_SEQ_FEATURE | CARBOHYD:O-linked (GalNAc...) threonine | 4 | 2.82 | 6.18E-02 | SPN, CD74, IGHA1, TNFRSF1B | 136 | 138 | 20675 | 4.41E+00 | 1.00E+00 | 1.00E+00 | 9.90E-01 |
| UP_SEQ_FEATURE | DOMAIN:Ig-like V-type | 4 | 2.82 | 6.29E-02 | VCAN, CD8B, CD8A, SLAMF1 | 136 | 139 | 20675 | 4.37E+00 | 1.00E+00 | 1.00E+00 | 9.90E-01 |
| GOTERM_BP_DIRECT | GO:0045665~negative regulation of neuron differentiation | 3 | 2.11 | 6.34E-02 | ITGB1, APP, CALR | 134 | 60 | 19478 | 7.27E+00 | 1.00E+00 | 7.61E-01 | 7.46E-01 |
| UP_SEQ_FEATURE | MOTIF:Secondary area of contact | 2 | 1.41 | 6.34E-02 | CST3, CST7 | 136 | 10 | 20675 | 3.04E+01 | 1.00E+00 | 1.00E+00 | 9.90E-01 |
| GOTERM_MF_DIRECT | GO:0001786~phosphatidylserine binding | 3 | 2.11 | 6.36E-02 | ANXA2, HMGB1, TLN1 | 135 | 59 | 19253 | 7.25E+00 | 1.00E+00 | 6.94E-01 | 6.67E-01 |
| GOTERM_CC_DIRECT | GO:0033018~sarcoplasmic reticulum lumen | 2 | 1.41 | 6.44E-02 | CALR, HSP90B1 | 139 | 10 | 20795 | 2.99E+01 | 1.00E+00 | 3.18E-01 | 2.84E-01 |
| GOTERM_BP_DIRECT | GO:0006265~DNA topological change | 2 | 1.41 | 6.62E-02 | HMGB1, TOP1 | 134 | 10 | 19478 | 2.91E+01 | 1.00E+00 | 7.61E-01 | 7.46E-01 |
| GOTERM_BP_DIRECT | GO:0045657~positive regulation of monocyte differentiation | 2 | 1.41 | 6.62E-02 | CD74, HLA-DRB1 | 134 | 10 | 19478 | 2.91E+01 | 1.00E+00 | 7.61E-01 | 7.46E-01 |
| GOTERM_BP_DIRECT | GO:0050863~regulation of T cell activation | 2 | 1.41 | 6.62E-02 | SPN, CCL5 | 134 | 10 | 19478 | 2.91E+01 | 1.00E+00 | 7.61E-01 | 7.46E-01 |
| GOTERM_BP_DIRECT | GO:0097067~cellular response to thyroid hormone stimulus | 2 | 1.41 | 6.62E-02 | CTSH, CTSS | 134 | 10 | 19478 | 2.91E+01 | 1.00E+00 | 7.61E-01 | 7.46E-01 |
| GOTERM_BP_DIRECT | GO:0002544~chronic inflammatory response | 2 | 1.41 | 6.62E-02 | S100A9, S100A8 | 134 | 10 | 19478 | 2.91E+01 | 1.00E+00 | 7.61E-01 | 7.46E-01 |
| GOTERM_BP_DIRECT | GO:0001649~osteoblast differentiation | 4 | 2.82 | 6.65E-02 | VCAN, TPM4, RRBP1, SND1 | 134 | 136 | 19478 | 4.28E+00 | 1.00E+00 | 7.61E-01 | 7.46E-01 |
| GOTERM_CC_DIRECT | GO:0055037~recycling endosome | 4 | 2.82 | 6.69E-02 | ITGB1, APP, RAB11FIP1, STX7 | 139 | 141 | 20795 | 4.24E+00 | 1.00E+00 | 3.25E-01 | 2.90E-01 |
| GOTERM_MF_DIRECT | GO:0015026~coreceptor activity | 3 | 2.11 | 6.74E-02 | ITGB1, CD8B, CD8A | 135 | 61 | 19253 | 7.01E+00 | 1.00E+00 | 6.94E-01 | 6.67E-01 |
| GOTERM_MF_DIRECT | GO:1904399~heparan sulfate binding | 2 | 1.41 | 6.75E-02 | APP, PPIA | 135 | 10 | 19253 | 2.85E+01 | 1.00E+00 | 6.94E-01 | 6.67E-01 |
| UP_KW_PTM | KW-1017~Isopeptide bond | 24 | 16.90 | 6.82E-02 | ITGB1, NUP214, APP, HSPA5, ANXA2, AHNAK, SSR4, RPN1, TSHZ2, RPLP0, RRBP1, HMGB1, FOS, SND1, ACTG1, CUX1, ANP32E, HLA-DRA, TOP1, PFN1, PPIA, HLA-DRB1, TMPO, S100A10 | 127 | 1900 | 14316 | 1.42E+00 | 7.39E-01 | 2.27E-01 | 1.82E-01 |
| GOTERM_BP_DIRECT | GO:0006959~humoral immune response | 3 | 2.11 | 6.90E-02 | HLA-DRB1, JCHAIN, HLA-DQB1 | 134 | 63 | 19478 | 6.92E+00 | 1.00E+00 | 7.81E-01 | 7.66E-01 |
| UP_SEQ_FEATURE | UNSURE:D or N | 2 | 1.41 | 6.95E-02 | MT-CO1, MT-CO2 | 136 | 11 | 20675 | 2.76E+01 | 1.00E+00 | 1.00E+00 | 9.90E-01 |
| UP_SEQ_FEATURE | DOMAIN:ITAM | 2 | 1.41 | 6.95E-02 | TYROBP, FCER1G | 136 | 11 | 20675 | 2.76E+01 | 1.00E+00 | 1.00E+00 | 9.90E-01 |
| UP_SEQ_FEATURE | REPEAT:33 | 2 | 1.41 | 6.95E-02 | NUP214, RRBP1 | 136 | 11 | 20675 | 2.76E+01 | 1.00E+00 | 1.00E+00 | 9.90E-01 |
| GOTERM_CC_DIRECT | GO:0044754~autolysosome | 2 | 1.41 | 7.06E-02 | FTH1, FTL | 139 | 11 | 20795 | 2.72E+01 | 1.00E+00 | 3.28E-01 | 2.93E-01 |
| GOTERM_CC_DIRECT | GO:0042582~azurophil granule | 2 | 1.41 | 7.06E-02 | STX7, STXBP2 | 139 | 11 | 20795 | 2.72E+01 | 1.00E+00 | 3.28E-01 | 2.93E-01 |
| GOTERM_CC_DIRECT | GO:0042612~MHC class I protein complex | 2 | 1.41 | 7.06E-02 | HLA-DPB1, HLA-DQB1 | 139 | 11 | 20795 | 2.72E+01 | 1.00E+00 | 3.28E-01 | 2.93E-01 |
| BIOCARTA | h_ecmPathway:Erk and PI-3 Kinase Are Necessary for Collagen Binding in Corneal Epithelia | 3 | 2.11 | 7.10E-02 | ITGB1, PFN1, TLN1 | 31 | 24 | 1622 | 6.54E+00 | 9.99E-01 | 1.00E+00 | 1.00E+00 |
| UP_SEQ_FEATURE | TRANSMEM:Helical; Anchor for type IV membrane protein | 3 | 2.11 | 7.11E-02 | CUX1, STX7, UBE2J1 | 136 | 67 | 20675 | 6.81E+00 | 1.00E+00 | 1.00E+00 | 9.90E-01 |
| UP_KW_DISEASE | KW-0523~Neurodegeneration | 7 | 4.93 | 7.18E-02 | DNAJC3, APP, GRN, FTH1, PSAP, PFN1, FTL | 34 | 439 | 4859 | 2.28E+00 | 8.93E-01 | 1.00E+00 | 1.00E+00 |
| GOTERM_BP_DIRECT | GO:0045063~T-helper 1 cell differentiation | 2 | 1.41 | 7.26E-02 | IL4R, HMGB1 | 134 | 11 | 19478 | 2.64E+01 | 1.00E+00 | 8.05E-01 | 7.89E-01 |
| GOTERM_BP_DIRECT | GO:0032757~positive regulation of interleukin-8 production | 3 | 2.11 | 7.29E-02 | FCN1, CD74, HMGB1 | 134 | 65 | 19478 | 6.71E+00 | 1.00E+00 | 8.05E-01 | 7.89E-01 |
| COG_ONTOLOGY | Cell division and chromosome partitioning | 3 | 2.11 | 7.37E-02 | NUP214, GOLGB1, TXNDC11 | 9 | 117 | 2025 | 5.77E+00 | 2.05E-01 | 2.21E-01 | 2.21E-01 |
| GOTERM_BP_DIRECT | GO:0043065~positive regulation of apoptotic process | 6 | 4.23 | 7.45E-02 | ITGB1, APP, LGALS1, GZMA, MNDA, HMGB1 | 134 | 328 | 19478 | 2.66E+00 | 1.00E+00 | 8.05E-01 | 7.89E-01 |
| GOTERM_BP_DIRECT | GO:0007417~central nervous system development | 4 | 2.82 | 7.47E-02 | APP, VCAN, GSTP1, APLP2 | 134 | 143 | 19478 | 4.07E+00 | 1.00E+00 | 8.05E-01 | 7.89E-01 |
| GOTERM_BP_DIRECT | GO:0043123~positive regulation of canonical NF-kappaB signal transduction | 5 | 3.52 | 7.48E-02 | CD74, LGALS1, S100A4, HLA-DRB1, MTDH | 134 | 232 | 19478 | 3.13E+00 | 1.00E+00 | 8.05E-01 | 7.89E-01 |
| GOTERM_BP_DIRECT | GO:0010595~positive regulation of endothelial cell migration | 3 | 2.11 | 7.68E-02 | NELL2, GRN, CALR | 134 | 67 | 19478 | 6.51E+00 | 1.00E+00 | 8.18E-01 | 8.02E-01 |
| GOTERM_CC_DIRECT | GO:0043231~intracellular membrane-bounded organelle | 12 | 8.45 | 7.79E-02 | APP, RAB11FIP1, CUX1, HSPA5, PSAP, CTSH, MNDA, PPIB, CLINT1, CTSS, HLA-DPA1, KLRG1 | 139 | 1022 | 20795 | 1.76E+00 | 1.00E+00 | 3.57E-01 | 3.18E-01 |
| GOTERM_BP_DIRECT | GO:2000343~positive regulation of chemokine (C-X-C motif) ligand 2 production | 2 | 1.41 | 7.90E-02 | CD74, HMGB1 | 134 | 12 | 19478 | 2.42E+01 | 1.00E+00 | 8.25E-01 | 8.08E-01 |
| GOTERM_BP_DIRECT | GO:0045060~negative thymic T cell selection | 2 | 1.41 | 7.90E-02 | SPN, CD74 | 134 | 12 | 19478 | 2.42E+01 | 1.00E+00 | 8.25E-01 | 8.08E-01 |
| UP_KW_CELLULAR_COMPONENT | KW-0490~MHC I | 2 | 1.41 | 7.93E-02 | HLA-DPB1, HLA-DQB1 | 136 | 11 | 18049 | 2.41E+01 | 9.09E-01 | 2.09E-01 | 1.51E-01 |
| GOTERM_MF_DIRECT | GO:0004197~cysteine-type endopeptidase activity | 3 | 2.11 | 7.93E-02 | CTSH, CTSW, CTSS | 135 | 67 | 19253 | 6.39E+00 | 1.00E+00 | 7.62E-01 | 7.32E-01 |
| UP_SEQ_FEATURE | TOPO_DOM:Extracellular | 27 | 19.01 | 8.03E-02 | ITGB1, APP, CD82, SPN, IGHA1, CCR4, SLAMF1, HLA-DPA1, KLRG1, CD74, HLA-DRB5, FCER1G, IL4R, APLP2, TNFRSF1B, NELL2, ADAM19, PTPRE, MS4A6A, TYROBP, CD8B, JAML, CD8A, HLA-DPB1, HLA-DRA, HLA-DRB1, HLA-DQB1 | 136 | 3012 | 20675 | 1.36E+00 | 1.00E+00 | 1.00E+00 | 9.90E-01 |
| GOTERM_MF_DIRECT | GO:0005049~nuclear export signal receptor activity | 2 | 1.41 | 8.04E-02 | NUP214, CALR | 135 | 12 | 19253 | 2.38E+01 | 1.00E+00 | 7.62E-01 | 7.32E-01 |
| GOTERM_MF_DIRECT | GO:0042608~T cell receptor binding | 2 | 1.41 | 8.04E-02 | HLA-DRA, HLA-DRB1 | 135 | 12 | 19253 | 2.38E+01 | 1.00E+00 | 7.62E-01 | 7.32E-01 |
| UP_SEQ_FEATURE | REPEAT:1 | 5 | 3.52 | 8.15E-02 | SRGN, NUP214, LGALS3, RRBP1, VCL | 136 | 250 | 20675 | 3.04E+00 | 1.00E+00 | 1.00E+00 | 9.90E-01 |
| UP_SEQ_FEATURE | REPEAT:32 | 2 | 1.41 | 8.17E-02 | NUP214, RRBP1 | 136 | 13 | 20675 | 2.34E+01 | 1.00E+00 | 1.00E+00 | 9.90E-01 |
| GOTERM_CC_DIRECT | GO:0071735~IgG immunoglobulin complex | 2 | 1.41 | 8.29E-02 | IGKC, IGHA1 | 139 | 13 | 20795 | 2.30E+01 | 1.00E+00 | 3.74E-01 | 3.34E-01 |
| UP_SEQ_FEATURE | REPEAT:2 | 5 | 3.52 | 8.43E-02 | SRGN, NUP214, LGALS3, RRBP1, VCL | 136 | 253 | 20675 | 3.00E+00 | 1.00E+00 | 1.00E+00 | 9.90E-01 |
| UP_SEQ_FEATURE | MOTIF:Nuclear localization signal | 7 | 4.93 | 8.46E-02 | SPN, AHNAK, SP140, MDM2, MNDA, SND1, TMPO | 136 | 465 | 20675 | 2.29E+00 | 1.00E+00 | 1.00E+00 | 9.90E-01 |
| GOTERM_BP_DIRECT | GO:0002474~antigen processing and presentation of peptide antigen via MHC class I | 2 | 1.41 | 8.52E-02 | HLA-DPB1, HLA-DQB1 | 134 | 13 | 19478 | 2.24E+01 | 1.00E+00 | 8.66E-01 | 8.48E-01 |
| GOTERM_BP_DIRECT | GO:1905146~lysosomal protein catabolic process | 2 | 1.41 | 8.52E-02 | CTSH, CST7 | 134 | 13 | 19478 | 2.24E+01 | 1.00E+00 | 8.66E-01 | 8.48E-01 |
| GOTERM_BP_DIRECT | GO:0033674~positive regulation of kinase activity | 2 | 1.41 | 8.52E-02 | CD74, HLA-DRB1 | 134 | 13 | 19478 | 2.24E+01 | 1.00E+00 | 8.66E-01 | 8.48E-01 |
| GOTERM_BP_DIRECT | GO:0006909~phagocytosis | 3 | 2.11 | 8.69E-02 | ITGB1, MESD, SLAMF1 | 134 | 72 | 19478 | 6.06E+00 | 1.00E+00 | 8.74E-01 | 8.57E-01 |
| INTERPRO | IPR028139:Humanin | 2 | 1.41 | 8.84E-02 | MTRNR2L12, MTRNR2L8 | 138 | 14 | 20808 | 2.15E+01 | 1.00E+00 | 7.05E-01 | 6.80E-01 |
| INTERPRO | IPR024936:Cyclophilin-type_PPIase | 2 | 1.41 | 8.84E-02 | PPIB, PPIA | 138 | 14 | 20808 | 2.15E+01 | 1.00E+00 | 7.05E-01 | 6.80E-01 |
| KEGG_PATHWAY | hsa04151:PI3K-Akt signaling pathway | 7 | 4.93 | 8.86E-02 | ITGB1, CDK6, CCND2, IL4R, DDIT4, MDM2, HSP90B1 | 74 | 362 | 8534 | 2.23E+00 | 1.00E+00 | 3.99E-01 | 3.45E-01 |
| GOTERM_BP_DIRECT | GO:0002523~leukocyte migration involved in inflammatory response | 2 | 1.41 | 9.15E-02 | S100A9, S100A8 | 134 | 14 | 19478 | 2.08E+01 | 1.00E+00 | 9.04E-01 | 8.86E-01 |
| GOTERM_BP_DIRECT | GO:1900272~negative regulation of long-term synaptic potentiation | 2 | 1.41 | 9.15E-02 | APP, TYROBP | 134 | 14 | 19478 | 2.08E+01 | 1.00E+00 | 9.04E-01 | 8.86E-01 |
| KEGG_PATHWAY | hsa04611:Platelet activation | 4 | 2.82 | 9.30E-02 | ITGB1, FCER1G, TLN1, ACTG1 | 74 | 126 | 8534 | 3.66E+00 | 1.00E+00 | 4.08E-01 | 3.53E-01 |
| GOTERM_MF_DIRECT | GO:0044548~S100 protein binding | 2 | 1.41 | 9.32E-02 | AHNAK, ANXA2 | 135 | 14 | 19253 | 2.04E+01 | 1.00E+00 | 8.39E-01 | 8.06E-01 |
| GOTERM_MF_DIRECT | GO:0008199~ferric iron binding | 2 | 1.41 | 9.32E-02 | FTH1, FTL | 135 | 14 | 19253 | 2.04E+01 | 1.00E+00 | 8.39E-01 | 8.06E-01 |
| UP_SEQ_FEATURE | REPEAT:31 | 2 | 1.41 | 9.36E-02 | NUP214, RRBP1 | 136 | 15 | 20675 | 2.03E+01 | 1.00E+00 | 1.00E+00 | 9.90E-01 |
| KEGG_PATHWAY | hsa04510:Focal adhesion | 5 | 3.52 | 9.55E-02 | ITGB1, CCND2, TLN1, VCL, ACTG1 | 74 | 203 | 8534 | 2.84E+00 | 1.00E+00 | 4.08E-01 | 3.53E-01 |
| UP_SEQ_FEATURE | REPEAT:5 | 4 | 2.82 | 9.63E-02 | SRGN, NUP214, LGALS3, RRBP1 | 136 | 167 | 20675 | 3.64E+00 | 1.00E+00 | 1.00E+00 | 9.90E-01 |
| GOTERM_MF_DIRECT | GO:0003779~actin binding | 6 | 4.23 | 9.70E-02 | ITGB1, TPM4, S100A4, PFN1, VCL, MYO1F | 135 | 349 | 19253 | 2.45E+00 | 1.00E+00 | 8.52E-01 | 8.19E-01 |
| GOTERM_BP_DIRECT | GO:0001913~T cell mediated cytotoxicity | 2 | 1.41 | 9.77E-02 | PRF1, CTSH | 134 | 15 | 19478 | 1.94E+01 | 1.00E+00 | 9.43E-01 | 9.24E-01 |
| GOTERM_BP_DIRECT | GO:0043518~negative regulation of DNA damage response, signal transduction by p53 class mediator | 2 | 1.41 | 9.77E-02 | CD74, MDM2 | 134 | 15 | 19478 | 1.94E+01 | 1.00E+00 | 9.43E-01 | 9.24E-01 |
| GOTERM_BP_DIRECT | GO:0045766~positive regulation of angiogenesis | 4 | 2.82 | 9.81E-02 | ITGB1, GRN, CTSH, MTDH | 134 | 161 | 19478 | 3.61E+00 | 1.00E+00 | 9.43E-01 | 9.24E-01 |
| UP_KW_PTM | KW-0325~Glycoprotein | 51 | 35.92 | 9.81E-02 | FCN1, ITGB1, APP, CD82, IGHV4-34, PRF1, CTSW, CTSS, JCHAIN, SPN, CTSH, CCR4, HLA-DPA1, SRGN, NUP214, IL4R, APLP2, TNFRSF1B, ADAM19, ERN1, VCAN, CD8B, JAML, CD8A, PPIB, PPIA, HLA-DQB1, GRN, CLDND1, RPN1, FGL2, AOAH, CST7, MESD, HSP90B1, CST3, CCL5, PSAP, IGHA1, SLAMF1, KLRG1, CD74, HLA-DRB5, GZMA, NELL2, SPCS3, PTPRE, HLA-DPB1, HLA-DRA, CALR, HLA-DRB1 | 127 | 4844 | 14316 | 1.19E+00 | 8.59E-01 | 2.80E-01 | 2.24E-01 |
| KEGG_PATHWAY | hsa05203:Viral carcinogenesis | 5 | 3.52 | 9.81E-02 | CDK6, CCND2, MDM2, CCR4, SND1 | 74 | 205 | 8534 | 2.81E+00 | 1.00E+00 | 4.09E-01 | 3.54E-01 |
| GOTERM_MF_DIRECT | GO:0030247~polysaccharide binding | 2 | 1.41 | 9.95E-02 | HLA-DRA, HLA-DRB1 | 135 | 15 | 19253 | 1.90E+01 | 1.00E+00 | 8.53E-01 | 8.20E-01 |
| UP_SEQ_FEATURE | REPEAT:1-4 | 2 | 1.41 | 9.95E-02 | CANX, CALR | 136 | 16 | 20675 | 1.90E+01 | 1.00E+00 | 1.00E+00 | 9.90E-01 |
| UP_SEQ_FEATURE | REGION:Alpha-2 | 2 | 1.41 | 9.95E-02 | HLA-DRA, HLA-DPA1 | 136 | 16 | 20675 | 1.90E+01 | 1.00E+00 | 1.00E+00 | 9.90E-01 |
| UP_SEQ_FEATURE | REGION:Alpha-1 | 2 | 1.41 | 9.95E-02 | HLA-DRA, HLA-DPA1 | 136 | 16 | 20675 | 1.90E+01 | 1.00E+00 | 1.00E+00 | 9.90E-01 |
