# Supplementary material for: Single-cell RNA sequencing unraveled immune-related expression heterogeneity and lymphoid cell development dysregulation in childhood asthma
Source: Front Immunol. 2026 Jan 2;16:1606650. doi: 10.3389/fimmu.2025.1606650 (PMC12807962; doi:10.3389/fimmu.2025.1606650)
Supplement: Supplementary file 5 [file Table4.docx]

**Supplementary Table 4.** GO results of 41 upregulated genes in CD4 T cells of Asthma 2 paitent

| Category | Term | Count | % | *P*-Value | Genes | List Total | Pop Hits | Pop Total | Fold Enrichment | Bonferroni | Benjamini | FDR |
| --- | --- | --- | --- | --- | --- | --- | --- | --- | --- | --- | --- | --- |
| GOTERM_CC_DIRECT | GO:0005576~extracellular region | 25 | 64.10 | 2.35E-14 | FCN1, IGHM, TSHZ2, CTSS, HSP90B1, TTN, JCHAIN, IGLV2-11, CST3, IGKV1-16, IGKC, PSAP, IGKV3-15, IL4R, MTRNR2L12, IGKV1-5, LYZ, NELL2, CD8B, MNDA, TLN1, S100A9, S100A8, IGKV3-20, IGLV3-19 | 39 | 2313 | 20795 | 5.76E+00 | 3.12E-12 | 3.12E-12 | 2.79E-12 |
| GOTERM_BP_DIRECT | GO:0006955~immune response | 15 | 38.46 | 6.53E-13 | IL4R, IGKV1-5, CTSS, JCHAIN, SPN, IGLV2-11, CST3, IGKV1-16, IGKC, CD8B, IGKV3-15, HLA-DRA, IGKV3-20, HLA-DRB1, IGLV3-19 | 39 | 537 | 19478 | 1.40E+01 | 2.32E-10 | 2.32E-10 | 2.25E-10 |
| GOTERM_MF_DIRECT | GO:0003823~antigen binding | 10 | 25.64 | 5.97E-12 | IGLV2-11, FCN1, IGHM, IGKV1-16, IGKC, IGKV1-5, IGKV3-15, IGKV3-20, IGLV3-19, JCHAIN | 39 | 140 | 19253 | 3.53E+01 | 9.14E-10 | 9.14E-10 | 9.08E-10 |
| UP_KW_BIOLOGICAL_PROCESS | KW-0391~Immunity | 17 | 43.59 | 3.54E-11 | FCN1, IGHM, IL4R, IGKV1-5, IGLV2-11, TYROBP, IGKV1-16, IGKC, CD8B, IGKV3-15, HLA-DRA, S100A9, S100A8, IGKV3-20, HLA-DRB1, IGLV3-19, KLRG1 | 28 | 980 | 11523 | 7.14E+00 | 7.43E-10 | 7.43E-10 | 7.08E-10 |
| GOTERM_BP_DIRECT | GO:0002250~adaptive immune response | 13 | 33.33 | 8.80E-11 | IGHM, IGKV1-5, CTSS, JCHAIN, IGLV2-11, IGKV1-16, IGKC, CD8B, IGKV3-15, HLA-DRA, IGKV3-20, HLA-DRB1, IGLV3-19 | 39 | 492 | 19478 | 1.32E+01 | 3.13E-08 | 1.56E-08 | 1.51E-08 |
| GOTERM_CC_DIRECT | GO:0070062~extracellular exosome | 21 | 53.85 | 2.03E-10 | IGHM, HSPA5, AHNAK, IGKV1-5, LYZ, TTN, HSP90B1, JCHAIN, SPN, CST3, IGKC, CANX, PSAP, HLA-DRA, MNDA, TLN1, S100A9, S100A8, IGKV3-20, HLA-DRB1, IGLV3-19 | 39 | 2242 | 20795 | 4.99E+00 | 2.71E-08 | 1.35E-08 | 1.21E-08 |
| UP_KW_CELLULAR_COMPONENT | KW-0964~Secreted | 21 | 53.85 | 5.78E-10 | FCN1, IGHM, IL4R, MTRNR2L12, TSHZ2, IGKV1-5, LYZ, CTSS, JCHAIN, IGLV2-11, NELL2, CST3, IGKV1-16, IGKC, CD8B, PSAP, IGKV3-15, S100A9, S100A8, IGKV3-20, IGLV3-19 | 37 | 2217 | 18049 | 4.62E+00 | 1.10E-08 | 1.10E-08 | 1.04E-08 |
| UP_SEQ_FEATURE | DOMAIN:Ig-like | 12 | 30.77 | 1.15E-08 | IGLV2-11, IGHM, IGKV1-16, IGKC, CD8B, IGKV1-5, IGKV3-15, HLA-DRA, IGKV3-20, HLA-DRB1, IGLV3-19, TTN | 37 | 673 | 20675 | 9.96E+00 | 7.60E-06 | 7.60E-06 | 7.56E-06 |
| INTERPRO | IPR007110:Ig-like_dom | 12 | 30.77 | 5.73E-08 | IGLV2-11, IGHM, IGKV1-16, IGKC, CD8B, IGKV1-5, IGKV3-15, HLA-DRA, IGKV3-20, HLA-DRB1, IGLV3-19, TTN | 38 | 768 | 20808 | 8.56E+00 | 9.80E-06 | 9.80E-06 | 9.68E-06 |
| UP_KW_CELLULAR_COMPONENT | KW-1280~Immunoglobulin | 8 | 20.51 | 8.88E-08 | IGLV2-11, IGHM, IGKV1-16, IGKC, IGKV1-5, IGKV3-15, IGKV3-20, IGLV3-19 | 37 | 192 | 18049 | 2.03E+01 | 1.69E-06 | 8.44E-07 | 8.00E-07 |
| UP_KW_BIOLOGICAL_PROCESS | KW-1064~Adaptive immunity | 11 | 28.21 | 1.38E-07 | IGLV2-11, IGHM, IGKV1-16, IGKC, CD8B, IGKV1-5, IGKV3-15, HLA-DRA, IGKV3-20, HLA-DRB1, IGLV3-19 | 28 | 521 | 11523 | 8.69E+00 | 2.90E-06 | 1.45E-06 | 1.38E-06 |
| INTERPRO | IPR036179:Ig-like_dom_sf | 12 | 30.77 | 2.07E-07 | IGLV2-11, IGHM, IGKV1-16, IGKC, CD8B, IGKV1-5, IGKV3-15, HLA-DRA, IGKV3-20, HLA-DRB1, IGLV3-19, TTN | 38 | 872 | 20808 | 7.54E+00 | 3.53E-05 | 1.77E-05 | 1.75E-05 |
| INTERPRO | IPR013783:Ig-like_fold | 13 | 33.33 | 3.15E-07 | IGHM, IL4R, IGKV1-5, TTN, IGLV2-11, IGKV1-16, IGKC, CD8B, IGKV3-15, HLA-DRA, IGKV3-20, HLA-DRB1, IGLV3-19 | 38 | 1127 | 20808 | 6.32E+00 | 5.39E-05 | 1.80E-05 | 1.78E-05 |
| UP_KW_PTM | KW-1015~Disulfide bond | 25 | 64.10 | 5.40E-07 | FCN1, IGHM, CTSS, HSP90B1, TTN, JCHAIN, IGLV2-11, CST3, IGKV1-16, IGKC, PSAP, IGKV3-15, KLRG1, IL4R, IGKV1-5, LYZ, NELL2, TYROBP, CD8B, GOLGB1, CANX, HLA-DRA, IGKV3-20, HLA-DRB1, IGLV3-19 | 36 | 3956 | 14316 | 2.51E+00 | 7.02E-06 | 7.56E-06 | 7.56E-06 |
| INTERPRO | IPR050150:IgV_Light_Chain | 6 | 15.38 | 6.23E-07 | IGLV2-11, IGKV1-16, IGKV1-5, IGKV3-15, IGKV3-20, IGLV3-19 | 38 | 93 | 20808 | 3.53E+01 | 1.06E-04 | 2.66E-05 | 2.63E-05 |
| KEGG_PATHWAY | hsa04612:Antigen processing and presentation | 6 | 15.38 | 1.23E-06 | HSPA5, CD8B, CANX, HLA-DRA, CTSS, HLA-DRB1 | 22 | 81 | 8534 | 2.87E+01 | 1.06E-04 | 1.06E-04 | 9.83E-05 |
| GOTERM_CC_DIRECT | GO:0019814~immunoglobulin complex | 7 | 17.95 | 1.31E-06 | IGLV2-11, IGHM, IGKV1-16, IGKV1-5, IGKV3-15, IGKV3-20, IGLV3-19 | 39 | 194 | 20795 | 1.92E+01 | 1.75E-04 | 5.82E-05 | 5.21E-05 |
| INTERPRO | IPR013106:Ig_V-set | 9 | 23.08 | 1.75E-06 | IGLV2-11, IGHM, IGKV1-16, CD8B, IGKV1-5, IGKV3-15, IGKV3-20, IGLV3-19, TTN | 38 | 485 | 20808 | 1.02E+01 | 3.00E-04 | 5.99E-05 | 5.92E-05 |
| SMART | SM00406:IGv | 9 | 23.08 | 2.65E-06 | IGLV2-11, IGHM, IGKV1-16, CD8B, IGKV1-5, IGKV3-15, IGKV3-20, IGLV3-19, TTN | 31 | 334 | 10706 | 9.31E+00 | 1.09E-04 | 1.09E-04 | 1.09E-04 |
| GOTERM_CC_DIRECT | GO:0005886~plasma membrane | 25 | 64.10 | 3.07E-06 | FCN1, IGHM, AHNAK, IGLV2-11, SPN, CST3, IGKV1-16, IGKC, PSAP, IGKV3-15, KLRG1, IL4R, HSPA5, IGKV1-5, NELL2, TYROBP, CD8B, HLA-DRA, TLN1, S100A9, S100A8, IGKV3-20, HLA-DRB1, IGLV3-19, MYO1F | 39 | 5597 | 20795 | 2.38E+00 | 4.08E-04 | 1.02E-04 | 9.12E-05 |
| UP_KW_DOMAIN | KW-0732~Signal | 24 | 61.54 | 3.27E-06 | FCN1, NUP214, IL4R, HSPA5, IGKV1-5, LYZ, CTSS, HSP90B1, JCHAIN, SPN, IGLV2-11, NELL2, CST3, TYROBP, IGKV1-16, CD8B, GOLGB1, CANX, PSAP, IGKV3-15, HLA-DRA, IGKV3-20, HLA-DRB1, IGLV3-19 | 34 | 4415 | 14625 | 2.34E+00 | 3.92E-05 | 3.92E-05 | 3.92E-05 |
| INTERPRO | IPR003599:Ig_sub | 9 | 23.08 | 3.43E-06 | IGLV2-11, IGHM, IGKV1-16, CD8B, IGKV1-5, IGKV3-15, IGKV3-20, IGLV3-19, TTN | 38 | 531 | 20808 | 9.28E+00 | 5.87E-04 | 9.78E-05 | 9.67E-05 |
| UP_SEQ_FEATURE | REGION:Complementarity-determining-3 | 6 | 15.38 | 4.07E-06 | IGLV2-11, IGKV1-16, IGKV1-5, IGKV3-15, IGKV3-20, IGLV3-19 | 37 | 139 | 20675 | 2.41E+01 | 2.70E-03 | 4.28E-04 | 4.26E-04 |
| UP_SEQ_FEATURE | REGION:Framework-1 | 6 | 15.38 | 4.37E-06 | IGLV2-11, IGKV1-16, IGKV1-5, IGKV3-15, IGKV3-20, IGLV3-19 | 37 | 141 | 20675 | 2.38E+01 | 2.89E-03 | 4.28E-04 | 4.26E-04 |
| UP_SEQ_FEATURE | REGION:Framework-3 | 6 | 15.38 | 4.52E-06 | IGLV2-11, IGKV1-16, IGKV1-5, IGKV3-15, IGKV3-20, IGLV3-19 | 37 | 142 | 20675 | 2.36E+01 | 2.99E-03 | 4.28E-04 | 4.26E-04 |
| UP_SEQ_FEATURE | REGION:Complementarity-determining-1 | 6 | 15.38 | 4.52E-06 | IGLV2-11, IGKV1-16, IGKV1-5, IGKV3-15, IGKV3-20, IGLV3-19 | 37 | 142 | 20675 | 2.36E+01 | 2.99E-03 | 4.28E-04 | 4.26E-04 |
| UP_SEQ_FEATURE | REGION:Complementarity-determining-2 | 6 | 15.38 | 4.52E-06 | IGLV2-11, IGKV1-16, IGKV1-5, IGKV3-15, IGKV3-20, IGLV3-19 | 37 | 142 | 20675 | 2.36E+01 | 2.99E-03 | 4.28E-04 | 4.26E-04 |
| UP_SEQ_FEATURE | REGION:Framework-2 | 6 | 15.38 | 4.52E-06 | IGLV2-11, IGKV1-16, IGKV1-5, IGKV3-15, IGKV3-20, IGLV3-19 | 37 | 142 | 20675 | 2.36E+01 | 2.99E-03 | 4.28E-04 | 4.26E-04 |
| GOTERM_CC_DIRECT | GO:0072562~blood microparticle | 6 | 15.38 | 7.09E-06 | IGHM, IGKC, IGKV1-5, IGKV3-15, IGKV3-20, JCHAIN | 39 | 148 | 20795 | 2.16E+01 | 9.42E-04 | 1.89E-04 | 1.69E-04 |
| GOTERM_CC_DIRECT | GO:0071756~pentameric IgM immunoglobulin complex | 3 | 7.69 | 9.74E-06 | IGHM, IGKV3-20, JCHAIN | 39 | 3 | 20795 | 5.33E+02 | 1.30E-03 | 2.16E-04 | 1.93E-04 |
| GOTERM_CC_DIRECT | GO:0005615~extracellular space | 14 | 35.90 | 1.50E-05 | FCN1, IGHM, LYZ, CTSS, JCHAIN, SPN, NELL2, CST3, IGKC, PSAP, S100A9, S100A8, IGKV3-20, HLA-DRB1 | 39 | 1867 | 20795 | 4.00E+00 | 1.99E-03 | 2.84E-04 | 2.54E-04 |
| UP_KW_DOMAIN | KW-0393~Immunoglobulin domain | 10 | 25.64 | 6.26E-05 | IGLV2-11, IGHM, IGKV1-16, IGKC, CD8B, IGKV1-5, IGKV3-15, IGKV3-20, IGLV3-19, TTN | 34 | 825 | 14625 | 5.21E+00 | 7.51E-04 | 3.75E-04 | 3.75E-04 |
| SMART | SM00409:IG | 9 | 23.08 | 7.71E-05 | IGLV2-11, IGHM, IGKV1-16, CD8B, IGKV1-5, IGKV3-15, IGKV3-20, IGLV3-19, TTN | 31 | 531 | 10706 | 5.85E+00 | 3.16E-03 | 1.58E-03 | 1.58E-03 |
| GOTERM_CC_DIRECT | GO:0009986~cell surface | 8 | 20.51 | 1.71E-04 | SPN, IGHM, TYROBP, HSPA5, CD8B, HLA-DRA, TLN1, HLA-DRB1 | 39 | 662 | 20795 | 6.44E+00 | 2.25E-02 | 2.85E-03 | 2.55E-03 |
| GOTERM_BP_DIRECT | GO:0006968~cellular defense response | 4 | 10.26 | 1.86E-04 | SPN, TYROBP, MNDA, KLRG1 | 39 | 57 | 19478 | 3.50E+01 | 6.40E-02 | 1.79E-02 | 1.73E-02 |
| GOTERM_BP_DIRECT | GO:0034975~protein folding in endoplasmic reticulum | 3 | 7.69 | 2.02E-04 | HSPA5, CANX, HSP90B1 | 39 | 11 | 19478 | 1.36E+02 | 6.91E-02 | 1.79E-02 | 1.73E-02 |
| INTERPRO | IPR003006:Ig/MHC_CS | 4 | 10.26 | 2.50E-04 | IGHM, IGKC, HLA-DRA, HLA-DRB1 | 38 | 69 | 20808 | 3.17E+01 | 4.19E-02 | 6.11E-03 | 6.04E-03 |
| INTERPRO | IPR003597:Ig_C1-set | 4 | 10.26 | 3.59E-04 | IGHM, IGKC, HLA-DRA, HLA-DRB1 | 38 | 78 | 20808 | 2.81E+01 | 5.96E-02 | 7.68E-03 | 7.59E-03 |
| UP_KW_LIGAND | KW-0106~Calcium | 8 | 20.51 | 4.63E-04 | NELL2, FCN1, TYROBP, CANX, S100A9, S100A8, HSP90B1, TTN | 13 | 990 | 6987 | 4.34E+00 | 3.24E-03 | 3.24E-03 | 3.24E-03 |
| GOTERM_BP_DIRECT | GO:0014002~astrocyte development | 3 | 7.69 | 4.95E-04 | CDK6, S100A9, S100A8 | 39 | 17 | 19478 | 8.81E+01 | 1.61E-01 | 3.51E-02 | 3.40E-02 |
| GOTERM_CC_DIRECT | GO:0062023~collagen-containing extracellular matrix | 6 | 15.38 | 6.59E-04 | FCN1, PSAP, S100A9, CTSS, S100A8, HSP90B1 | 39 | 387 | 20795 | 8.27E+00 | 8.39E-02 | 9.73E-03 | 8.71E-03 |
| UP_KW_CELLULAR_COMPONENT | KW-1003~Cell membrane | 18 | 46.15 | 1.14E-03 | FCN1, IGHM, IL4R, IGKV1-5, IGLV2-11, TYROBP, IGKV1-16, IGKC, CD8B, IGKV3-15, HLA-DRA, TLN1, S100A9, S100A8, IGKV3-20, HLA-DRB1, IGLV3-19, KLRG1 | 37 | 4134 | 18049 | 2.12E+00 | 2.14E-02 | 7.22E-03 | 6.84E-03 |
| SMART | SM00407:IGc1 | 4 | 10.26 | 1.17E-03 | IGHM, IGKC, HLA-DRA, HLA-DRB1 | 31 | 75 | 10706 | 1.84E+01 | 4.69E-02 | 1.60E-02 | 1.60E-02 |
| GOTERM_CC_DIRECT | GO:1904813~ficolin-1-rich granule lumen | 4 | 10.26 | 1.57E-03 | FCN1, CST3, MNDA, CTSS | 39 | 126 | 20795 | 1.69E+01 | 1.89E-01 | 2.09E-02 | 1.87E-02 |
| GOTERM_BP_DIRECT | GO:0019886~antigen processing and presentation of exogenous peptide antigen via MHC class II | 3 | 7.69 | 1.66E-03 | HLA-DRA, CTSS, HLA-DRB1 | 39 | 31 | 19478 | 4.83E+01 | 4.46E-01 | 9.84E-02 | 9.53E-02 |
| KEGG_PATHWAY | hsa04640:Hematopoietic cell lineage | 4 | 10.26 | 1.78E-03 | IL4R, CD8B, HLA-DRA, HLA-DRB1 | 22 | 100 | 8534 | 1.55E+01 | 1.42E-01 | 5.58E-02 | 5.19E-02 |
| GOTERM_CC_DIRECT | GO:0098553~lumenal side of endoplasmic reticulum membrane | 3 | 7.69 | 1.86E-03 | CANX, HLA-DRA, HLA-DRB1 | 39 | 35 | 20795 | 4.57E+01 | 2.20E-01 | 2.25E-02 | 2.01E-02 |
| KEGG_PATHWAY | hsa05166:Human T-cell leukemia virus 1 infection | 5 | 12.82 | 1.95E-03 | CCND2, CANX, HLA-DRA, TLN1, HLA-DRB1 | 22 | 224 | 8534 | 8.66E+00 | 1.54E-01 | 5.58E-02 | 5.19E-02 |
| GOTERM_MF_DIRECT | GO:0005509~calcium ion binding | 7 | 17.95 | 3.03E-03 | NELL2, HSPA5, CANX, S100A9, S100A8, HSP90B1, TTN | 39 | 739 | 19253 | 4.68E+00 | 3.71E-01 | 2.01E-01 | 2.00E-01 |
| UP_SEQ_FEATURE | MUTAGEN:L->R: Decreases the interaction with CD4. | 2 | 5.13 | 3.48E-03 | HLA-DRA, HLA-DRB1 | 37 | 2 | 20675 | 5.59E+02 | 9.01E-01 | 2.88E-01 | 2.87E-01 |
| GOTERM_BP_DIRECT | GO:0019882~antigen processing and presentation | 3 | 7.69 | 3.48E-03 | HLA-DRA, CTSS, HLA-DRB1 | 39 | 45 | 19478 | 3.33E+01 | 7.10E-01 | 1.54E-01 | 1.49E-01 |
| GOTERM_CC_DIRECT | GO:1990660~calprotectin complex | 2 | 5.13 | 3.65E-03 | S100A9, S100A8 | 39 | 2 | 20795 | 5.33E+02 | 3.85E-01 | 4.05E-02 | 3.62E-02 |
| BIOCARTA | h_th1th2Pathway:Th1/Th2 Differentiation | 3 | 7.69 | 3.87E-03 | IL4R, HLA-DRA, HLA-DRB1 | 9 | 20 | 1622 | 2.70E+01 | 9.94E-02 | 1.05E-01 | 1.05E-01 |
| GOTERM_BP_DIRECT | GO:0002491~antigen processing and presentation of endogenous peptide antigen via MHC class II | 2 | 5.13 | 3.90E-03 | HLA-DRA, HLA-DRB1 | 39 | 2 | 19478 | 4.99E+02 | 7.50E-01 | 1.54E-01 | 1.49E-01 |
| GOTERM_BP_DIRECT | GO:0070488~neutrophil aggregation | 2 | 5.13 | 3.90E-03 | S100A9, S100A8 | 39 | 2 | 19478 | 4.99E+02 | 7.50E-01 | 1.54E-01 | 1.49E-01 |
| GOTERM_MF_DIRECT | GO:0097493~structural molecule activity conferring elasticity | 2 | 5.13 | 3.94E-03 | AHNAK, TTN | 39 | 2 | 19253 | 4.94E+02 | 4.54E-01 | 2.01E-01 | 2.00E-01 |
| GOTERM_BP_DIRECT | GO:0042742~defense response to bacterium | 4 | 10.26 | 4.48E-03 | SPN, LYZ, S100A9, S100A8 | 39 | 171 | 19478 | 1.17E+01 | 7.97E-01 | 1.59E-01 | 1.54E-01 |
| GOTERM_CC_DIRECT | GO:1904724~tertiary granule lumen | 3 | 7.69 | 4.71E-03 | CST3, LYZ, CTSS | 39 | 56 | 20795 | 2.86E+01 | 4.66E-01 | 4.81E-02 | 4.31E-02 |
| GOTERM_BP_DIRECT | GO:0002469~myeloid dendritic cell antigen processing and presentation | 2 | 5.13 | 5.84E-03 | HLA-DRA, HLA-DRB1 | 39 | 3 | 19478 | 3.33E+02 | 8.75E-01 | 1.75E-01 | 1.69E-01 |
| GOTERM_BP_DIRECT | GO:0050853~B cell receptor signaling pathway | 3 | 7.69 | 5.91E-03 | IGHM, IGKC, MNDA | 39 | 59 | 19478 | 2.54E+01 | 8.78E-01 | 1.75E-01 | 1.69E-01 |
| KEGG_PATHWAY | hsa04514:Cell adhesion molecules | 4 | 10.26 | 6.48E-03 | SPN, CD8B, HLA-DRA, HLA-DRB1 | 22 | 158 | 8534 | 9.82E+00 | 4.28E-01 | 1.13E-01 | 1.06E-01 |
| KEGG_PATHWAY | hsa04145:Phagosome | 4 | 10.26 | 6.60E-03 | CANX, HLA-DRA, CTSS, HLA-DRB1 | 22 | 159 | 8534 | 9.76E+00 | 4.34E-01 | 1.13E-01 | 1.06E-01 |
| GOTERM_CC_DIRECT | GO:0120281~autolysosome membrane | 2 | 5.13 | 7.29E-03 | HLA-DRA, HLA-DRB1 | 39 | 4 | 20795 | 2.67E+02 | 6.22E-01 | 6.46E-02 | 5.78E-02 |
| GOTERM_CC_DIRECT | GO:0071748~monomeric IgA immunoglobulin complex | 2 | 5.13 | 7.29E-03 | IGKV3-20, JCHAIN | 39 | 4 | 20795 | 2.67E+02 | 6.22E-01 | 6.46E-02 | 5.78E-02 |
| GOTERM_BP_DIRECT | GO:2000516~positive regulation of CD4-positive, alpha-beta T cell activation | 2 | 5.13 | 7.78E-03 | HLA-DRA, HLA-DRB1 | 39 | 4 | 19478 | 2.50E+02 | 9.38E-01 | 1.97E-01 | 1.91E-01 |
| GOTERM_BP_DIRECT | GO:0045622~regulation of T-helper cell differentiation | 2 | 5.13 | 7.78E-03 | HLA-DRA, HLA-DRB1 | 39 | 4 | 19478 | 2.50E+02 | 9.38E-01 | 1.97E-01 | 1.91E-01 |
| GOTERM_MF_DIRECT | GO:0035662~Toll-like receptor 4 binding | 2 | 5.13 | 7.87E-03 | S100A9, S100A8 | 39 | 4 | 19253 | 2.47E+02 | 7.02E-01 | 3.01E-01 | 2.99E-01 |
| GOTERM_CC_DIRECT | GO:0071751~secretory IgA immunoglobulin complex | 2 | 5.13 | 9.10E-03 | IGKV3-20, JCHAIN | 39 | 5 | 20795 | 2.13E+02 | 7.04E-01 | 7.57E-02 | 6.77E-02 |
| GOTERM_BP_DIRECT | GO:0019731~antibacterial humoral response | 3 | 7.69 | 9.65E-03 | IGHM, IGKV3-20, JCHAIN | 39 | 76 | 19478 | 1.97E+01 | 9.68E-01 | 2.16E-01 | 2.09E-01 |
| GOTERM_BP_DIRECT | GO:0032831~positive regulation of CD4-positive, CD25-positive, alpha-beta regulatory T cell differentiation | 2 | 5.13 | 9.72E-03 | HLA-DRA, HLA-DRB1 | 39 | 5 | 19478 | 2.00E+02 | 9.69E-01 | 2.16E-01 | 2.09E-01 |
| UP_KW_MOLECULAR_FUNCTION | KW-0929~Antimicrobial | 3 | 7.69 | 1.03E-02 | LYZ, S100A9, S100A8 | 17 | 116 | 11952 | 1.82E+01 | 2.27E-01 | 2.56E-01 | 2.56E-01 |
| GOTERM_CC_DIRECT | GO:0071753~IgM immunoglobulin complex | 2 | 5.13 | 1.09E-02 | IGHM, IGKC | 39 | 6 | 20795 | 1.78E+02 | 7.68E-01 | 8.54E-02 | 7.64E-02 |
| KEGG_PATHWAY | hsa05321:Inflammatory bowel disease | 3 | 7.69 | 1.13E-02 | IL4R, HLA-DRA, HLA-DRB1 | 22 | 66 | 8534 | 1.76E+01 | 6.22E-01 | 1.54E-01 | 1.44E-01 |
| KEGG_PATHWAY | hsa05169:Epstein-Barr virus infection | 4 | 10.26 | 1.30E-02 | CDK6, CCND2, HLA-DRA, HLA-DRB1 | 22 | 204 | 8534 | 7.61E+00 | 6.76E-01 | 1.54E-01 | 1.44E-01 |
| GOTERM_BP_DIRECT | GO:0035425~autocrine signaling | 2 | 5.13 | 1.36E-02 | S100A9, S100A8 | 39 | 7 | 19478 | 1.43E+02 | 9.92E-01 | 2.84E-01 | 2.75E-01 |
| GOTERM_MF_DIRECT | GO:0050544~arachidonate binding | 2 | 5.13 | 1.37E-02 | S100A9, S100A8 | 39 | 7 | 19253 | 1.41E+02 | 8.80E-01 | 4.06E-01 | 4.04E-01 |
| KEGG_PATHWAY | hsa04918:Thyroid hormone synthesis | 3 | 7.69 | 1.44E-02 | HSPA5, CANX, HSP90B1 | 22 | 75 | 8534 | 1.55E+01 | 7.12E-01 | 1.54E-01 | 1.44E-01 |
| GOTERM_BP_DIRECT | GO:0036503~ERAD pathway | 3 | 7.69 | 1.51E-02 | HSPA5, CANX, HSP90B1 | 39 | 96 | 19478 | 1.56E+01 | 9.95E-01 | 2.97E-01 | 2.88E-01 |
| GOTERM_BP_DIRECT | GO:0043382~positive regulation of memory T cell differentiation | 2 | 5.13 | 1.74E-02 | HLA-DRA, HLA-DRB1 | 39 | 9 | 19478 | 1.11E+02 | 9.98E-01 | 3.26E-01 | 3.15E-01 |
| GOTERM_BP_DIRECT | GO:0002544~chronic inflammatory response | 2 | 5.13 | 1.93E-02 | S100A9, S100A8 | 39 | 10 | 19478 | 9.99E+01 | 9.99E-01 | 3.40E-01 | 3.30E-01 |
| GOTERM_CC_DIRECT | GO:0034774~secretory granule lumen | 3 | 7.69 | 1.96E-02 | FCN1, S100A9, S100A8 | 39 | 118 | 20795 | 1.36E+01 | 9.29E-01 | 1.34E-01 | 1.20E-01 |
| GOTERM_CC_DIRECT | GO:0034663~endoplasmic reticulum chaperone complex | 2 | 5.13 | 1.99E-02 | HSPA5, HSP90B1 | 39 | 11 | 20795 | 9.69E+01 | 9.31E-01 | 1.34E-01 | 1.20E-01 |
| GOTERM_BP_DIRECT | GO:0050729~positive regulation of inflammatory response | 3 | 7.69 | 2.01E-02 | NEAT1, S100A9, S100A8 | 39 | 112 | 19478 | 1.34E+01 | 9.99E-01 | 3.40E-01 | 3.30E-01 |
| GOTERM_CC_DIRECT | GO:0005788~endoplasmic reticulum lumen | 4 | 10.26 | 2.01E-02 | CST3, HSPA5, CANX, HSP90B1 | 39 | 318 | 20795 | 6.71E+00 | 9.33E-01 | 1.34E-01 | 1.20E-01 |
| UP_SEQ_FEATURE | CARBOHYD:N-linked (GlcNAc...) (complex) asparagine | 3 | 7.69 | 2.03E-02 | IGHM, PSAP, JCHAIN | 37 | 126 | 20675 | 1.33E+01 | 1.00E+00 | 1.00E+00 | 9.95E-01 |
| GOTERM_MF_DIRECT | GO:0002020~protease binding | 3 | 7.69 | 2.06E-02 | CST3, PSAP, TTN | 39 | 112 | 19253 | 1.32E+01 | 9.58E-01 | 4.06E-01 | 4.04E-01 |
| UP_KW_BIOLOGICAL_PROCESS | KW-0945~Host-virus interaction | 6 | 15.38 | 2.10E-02 | NELL2, NUP214, HSPA5, CANX, HLA-DRA, TLN1 | 28 | 695 | 11523 | 3.55E+00 | 3.60E-01 | 1.47E-01 | 1.40E-01 |
| GOTERM_MF_DIRECT | GO:0050786~RAGE receptor binding | 2 | 5.13 | 2.15E-02 | S100A9, S100A8 | 39 | 11 | 19253 | 8.98E+01 | 9.64E-01 | 4.06E-01 | 4.04E-01 |
| KEGG_PATHWAY | hsa04658:Th1 and Th2 cell differentiation | 3 | 7.69 | 2.16E-02 | IL4R, HLA-DRA, HLA-DRB1 | 22 | 93 | 8534 | 1.25E+01 | 8.47E-01 | 1.93E-01 | 1.80E-01 |
| KEGG_PATHWAY | hsa04657:IL-17 signaling pathway | 3 | 7.69 | 2.24E-02 | S100A9, S100A8, HSP90B1 | 22 | 95 | 8534 | 1.22E+01 | 8.58E-01 | 1.93E-01 | 1.80E-01 |
| GOTERM_BP_DIRECT | GO:0034121~regulation of toll-like receptor signaling pathway | 2 | 5.13 | 2.32E-02 | S100A9, S100A8 | 39 | 12 | 19478 | 8.32E+01 | 1.00E+00 | 3.74E-01 | 3.62E-01 |
| GOTERM_MF_DIRECT | GO:0042608~T cell receptor binding | 2 | 5.13 | 2.34E-02 | HLA-DRA, HLA-DRB1 | 39 | 12 | 19253 | 8.23E+01 | 9.73E-01 | 4.06E-01 | 4.04E-01 |
| GOTERM_BP_DIRECT | GO:0045087~innate immune response | 5 | 12.82 | 2.44E-02 | IGHM, S100A9, S100A8, JCHAIN, KLRG1 | 39 | 570 | 19478 | 4.38E+00 | 1.00E+00 | 3.77E-01 | 3.65E-01 |
| GOTERM_MF_DIRECT | GO:0032395~MHC class II receptor activity | 2 | 5.13 | 2.54E-02 | HLA-DRA, HLA-DRB1 | 39 | 13 | 19253 | 7.59E+01 | 9.80E-01 | 4.06E-01 | 4.04E-01 |
| GOTERM_MF_DIRECT | GO:0051082~unfolded protein binding | 3 | 7.69 | 2.68E-02 | HSPA5, CANX, HSP90B1 | 39 | 129 | 19253 | 1.15E+01 | 9.84E-01 | 4.06E-01 | 4.04E-01 |
| GOTERM_BP_DIRECT | GO:0002523~leukocyte migration involved in inflammatory response | 2 | 5.13 | 2.70E-02 | S100A9, S100A8 | 39 | 14 | 19478 | 7.13E+01 | 1.00E+00 | 3.99E-01 | 3.87E-01 |
| KEGG_PATHWAY | hsa04659:Th17 cell differentiation | 3 | 7.69 | 2.90E-02 | IL4R, HLA-DRA, HLA-DRB1 | 22 | 109 | 8534 | 1.07E+01 | 9.20E-01 | 2.27E-01 | 2.11E-01 |
| GOTERM_MF_DIRECT | GO:0030247~polysaccharide binding | 2 | 5.13 | 2.92E-02 | HLA-DRA, HLA-DRB1 | 39 | 15 | 19253 | 6.58E+01 | 9.89E-01 | 4.06E-01 | 4.04E-01 |
| GOTERM_BP_DIRECT | GO:0002503~peptide antigen assembly with MHC class II protein complex | 2 | 5.13 | 3.08E-02 | HLA-DRA, HLA-DRB1 | 39 | 16 | 19478 | 6.24E+01 | 1.00E+00 | 4.37E-01 | 4.23E-01 |
| UP_SEQ_FEATURE | DOMAIN:Immunoglobulin C1-set | 2 | 5.13 | 3.26E-02 | HLA-DRA, HLA-DRB1 | 37 | 19 | 20675 | 5.88E+01 | 1.00E+00 | 1.00E+00 | 9.95E-01 |
| UP_SEQ_FEATURE | MOTIF:ITIM motif | 2 | 5.13 | 3.43E-02 | IL4R, KLRG1 | 37 | 20 | 20675 | 5.59E+01 | 1.00E+00 | 1.00E+00 | 9.95E-01 |
| GOTERM_BP_DIRECT | GO:0030889~negative regulation of B cell proliferation | 2 | 5.13 | 3.46E-02 | TYROBP, MNDA | 39 | 18 | 19478 | 5.55E+01 | 1.00E+00 | 4.72E-01 | 4.57E-01 |
| INTERPRO | IPR050380:Immune_Resp_Modulators | 2 | 5.13 | 3.50E-02 | IGHM, IGKC | 38 | 20 | 20808 | 5.48E+01 | 9.98E-01 | 6.65E-01 | 6.57E-01 |
| UP_SEQ_FEATURE | DOMAIN:Ig-like 4 | 2 | 5.13 | 3.60E-02 | IGHM, TTN | 37 | 21 | 20675 | 5.32E+01 | 1.00E+00 | 1.00E+00 | 9.95E-01 |
| GOTERM_CC_DIRECT | GO:0005765~lysosomal membrane | 4 | 10.26 | 3.65E-02 | AHNAK, PSAP, HLA-DRA, HLA-DRB1 | 39 | 401 | 20795 | 5.32E+00 | 9.93E-01 | 2.31E-01 | 2.07E-01 |
| GOTERM_MF_DIRECT | GO:0034987~immunoglobulin receptor binding | 2 | 5.13 | 3.69E-02 | IGHM, JCHAIN | 39 | 19 | 19253 | 5.20E+01 | 9.97E-01 | 4.56E-01 | 4.53E-01 |
| UP_KW_LIGAND | KW-0430~Lectin | 3 | 7.69 | 3.71E-02 | FCN1, CANX, KLRG1 | 13 | 181 | 6987 | 8.91E+00 | 2.33E-01 | 1.30E-01 | 1.30E-01 |
| GOTERM_BP_DIRECT | GO:0002504~antigen processing and presentation of peptide or polysaccharide antigen via MHC class II | 2 | 5.13 | 3.83E-02 | HLA-DRA, HLA-DRB1 | 39 | 20 | 19478 | 4.99E+01 | 1.00E+00 | 4.86E-01 | 4.71E-01 |
| GOTERM_BP_DIRECT | GO:0003094~glomerular filtration | 2 | 5.13 | 3.83E-02 | IGKV3-20, JCHAIN | 39 | 20 | 19478 | 4.99E+01 | 1.00E+00 | 4.86E-01 | 4.71E-01 |
| GOTERM_MF_DIRECT | GO:0042834~peptidoglycan binding | 2 | 5.13 | 3.88E-02 | IGHM, JCHAIN | 39 | 20 | 19253 | 4.94E+01 | 9.98E-01 | 4.56E-01 | 4.53E-01 |
| BIOCARTA | h_eosinophilsPathway:The Role of Eosinophils in the Chemokine Network of Allergy | 2 | 5.13 | 3.89E-02 | HLA-DRA, HLA-DRB1 | 9 | 8 | 1622 | 4.51E+01 | 6.57E-01 | 2.05E-01 | 2.05E-01 |
| UP_KW_CELLULAR_COMPONENT | KW-0491~MHC II | 2 | 5.13 | 3.92E-02 | HLA-DRA, HLA-DRB1 | 37 | 20 | 18049 | 4.88E+01 | 5.32E-01 | 1.51E-01 | 1.43E-01 |
| GOTERM_CC_DIRECT | GO:0009897~external side of plasma membrane | 4 | 10.26 | 3.93E-02 | SPN, FCN1, IL4R, HLA-DRB1 | 39 | 413 | 20795 | 5.16E+00 | 9.95E-01 | 2.38E-01 | 2.13E-01 |
| UP_KW_MOLECULAR_FUNCTION | KW-0143~Chaperone | 3 | 7.69 | 4.16E-02 | HSPA5, CANX, HSP90B1 | 17 | 245 | 11952 | 8.61E+00 | 6.54E-01 | 5.20E-01 | 5.20E-01 |
| INTERPRO | IPR014745:MHC_II_a/b_N | 2 | 5.13 | 4.18E-02 | HLA-DRA, HLA-DRB1 | 38 | 24 | 20808 | 4.56E+01 | 9.99E-01 | 6.77E-01 | 6.69E-01 |
| INTERPRO | IPR001751:S100/CaBP7/8-like_CS | 2 | 5.13 | 4.35E-02 | S100A9, S100A8 | 38 | 25 | 20808 | 4.38E+01 | 1.00E+00 | 6.77E-01 | 6.69E-01 |
| BIOCARTA | h_bbcellPathway:Bystander B Cell Activation | 2 | 5.13 | 4.36E-02 | HLA-DRA, HLA-DRB1 | 9 | 9 | 1622 | 4.00E+01 | 7.00E-01 | 2.05E-01 | 2.05E-01 |
| GOTERM_CC_DIRECT | GO:0005925~focal adhesion | 4 | 10.26 | 4.42E-02 | AHNAK, HSPA5, TLN1, HSP90B1 | 39 | 433 | 20795 | 4.93E+00 | 9.98E-01 | 2.56E-01 | 2.29E-01 |
| GOTERM_BP_DIRECT | GO:0051493~regulation of cytoskeleton organization | 2 | 5.13 | 4.58E-02 | S100A9, S100A8 | 39 | 24 | 19478 | 4.16E+01 | 1.00E+00 | 5.61E-01 | 5.43E-01 |
| GOTERM_CC_DIRECT | GO:0042613~MHC class II protein complex | 2 | 5.13 | 4.65E-02 | HLA-DRA, HLA-DRB1 | 39 | 26 | 20795 | 4.10E+01 | 9.98E-01 | 2.58E-01 | 2.30E-01 |
| BIOCARTA | h_il5Pathway:IL 5 Signaling Pathway | 2 | 5.13 | 4.84E-02 | HLA-DRA, HLA-DRB1 | 9 | 10 | 1622 | 3.60E+01 | 7.38E-01 | 2.05E-01 | 2.05E-01 |
| INTERPRO | IPR013787:S100_Ca-bd_sub | 2 | 5.13 | 4.86E-02 | S100A9, S100A8 | 38 | 28 | 20808 | 3.91E+01 | 1.00E+00 | 6.84E-01 | 6.76E-01 |
| UP_KW_CELLULAR_COMPONENT | KW-0034~Amyloid | 2 | 5.13 | 4.87E-02 | CST3, LYZ | 37 | 25 | 18049 | 3.90E+01 | 6.13E-01 | 1.51E-01 | 1.43E-01 |
| GOTERM_BP_DIRECT | GO:0006954~inflammatory response | 4 | 10.26 | 5.16E-02 | LYZ, S100A9, S100A8, KLRG1 | 39 | 432 | 19478 | 4.62E+00 | 1.00E+00 | 6.11E-01 | 5.92E-01 |
| GOTERM_MF_DIRECT | GO:0023026~MHC class II protein complex binding | 2 | 5.13 | 5.20E-02 | HLA-DRA, HLA-DRB1 | 39 | 27 | 19253 | 3.66E+01 | 1.00E+00 | 5.68E-01 | 5.64E-01 |
| INTERPRO | IPR050160:MHC/Immunoglobulin | 2 | 5.13 | 5.20E-02 | HLA-DRA, HLA-DRB1 | 38 | 30 | 20808 | 3.65E+01 | 1.00E+00 | 6.84E-01 | 6.76E-01 |
| UP_SEQ_FEATURE | DOMAIN:Ig-like 3 | 2 | 5.13 | 5.26E-02 | IGHM, TTN | 37 | 31 | 20675 | 3.61E+01 | 1.00E+00 | 1.00E+00 | 9.95E-01 |
| UP_KW_CELLULAR_COMPONENT | KW-0472~Membrane | 23 | 58.97 | 5.28E-02 | FCN1, IGHM, IL4R, IGKV1-5, SPN, IGLV2-11, NELL2, TYROBP, CCND2, IGKV1-16, IGKC, CD8B, GOLGB1, CANX, IGKV3-15, HLA-DRA, TLN1, S100A9, S100A8, IGKV3-20, HLA-DRB1, IGLV3-19, KLRG1 | 37 | 8353 | 18049 | 1.34E+00 | 6.43E-01 | 1.51E-01 | 1.43E-01 |
| UP_KW_DISEASE | KW-1008~Amyloidosis | 2 | 5.13 | 5.31E-02 | CST3, LYZ | 9 | 33 | 4859 | 3.27E+01 | 6.04E-01 | 9.03E-01 | 9.03E-01 |
| BIOCARTA | h_mhcPathway:Antigen Processing and Presentation | 2 | 5.13 | 5.31E-02 | HLA-DRA, HLA-DRB1 | 9 | 11 | 1622 | 3.28E+01 | 7.71E-01 | 2.05E-01 | 2.05E-01 |
| BIOCARTA | h_tcraPathway:Lck and Fyn tyrosine kinases in initiation of TCR Activation | 2 | 5.13 | 5.31E-02 | HLA-DRA, HLA-DRB1 | 9 | 11 | 1622 | 3.28E+01 | 7.71E-01 | 2.05E-01 | 2.05E-01 |
| BIOCARTA | h_blymphocytePathway:B Lymphocyte Cell Surface Molecules | 2 | 5.13 | 5.31E-02 | HLA-DRA, HLA-DRB1 | 9 | 11 | 1622 | 3.28E+01 | 7.71E-01 | 2.05E-01 | 2.05E-01 |
| UP_KW_CELLULAR_COMPONENT | KW-0458~Lysosome | 4 | 10.26 | 5.56E-02 | PSAP, HLA-DRA, CTSS, HLA-DRB1 | 37 | 436 | 18049 | 4.48E+00 | 6.62E-01 | 1.51E-01 | 1.43E-01 |
| KEGG_PATHWAY | hsa04151:PI3K-Akt signaling pathway | 4 | 10.26 | 5.72E-02 | CDK6, IL4R, CCND2, HSP90B1 | 22 | 362 | 8534 | 4.29E+00 | 9.94E-01 | 4.08E-01 | 3.80E-01 |
| GOTERM_MF_DIRECT | GO:0031210~phosphatidylcholine binding | 2 | 5.13 | 5.76E-02 | IGHM, JCHAIN | 39 | 30 | 19253 | 3.29E+01 | 1.00E+00 | 5.87E-01 | 5.84E-01 |
| BIOCARTA | h_asbcellPathway:Antigen Dependent B Cell Activation | 2 | 5.13 | 6.25E-02 | HLA-DRA, HLA-DRB1 | 9 | 13 | 1622 | 2.77E+01 | 8.25E-01 | 2.11E-01 | 2.11E-01 |
| KEGG_PATHWAY | hsa04141:Protein processing in endoplasmic reticulum | 3 | 7.69 | 6.47E-02 | HSPA5, CANX, HSP90B1 | 22 | 170 | 8534 | 6.85E+00 | 9.97E-01 | 4.08E-01 | 3.80E-01 |
| GOTERM_MF_DIRECT | GO:0030246~carbohydrate binding | 3 | 7.69 | 6.66E-02 | FCN1, CANX, KLRG1 | 39 | 214 | 19253 | 6.92E+00 | 1.00E+00 | 6.14E-01 | 6.10E-01 |
| KEGG_PATHWAY | hsa05164:Influenza A | 3 | 7.69 | 6.67E-02 | CDK6, HLA-DRA, HLA-DRB1 | 22 | 173 | 8534 | 6.73E+00 | 9.97E-01 | 4.08E-01 | 3.80E-01 |
| UP_SEQ_FEATURE | DOMAIN:Ig-like C1-type | 2 | 5.13 | 6.74E-02 | HLA-DRA, HLA-DRB1 | 37 | 40 | 20675 | 2.79E+01 | 1.00E+00 | 1.00E+00 | 9.95E-01 |
| GOTERM_BP_DIRECT | GO:0050776~regulation of immune response | 2 | 5.13 | 6.79E-02 | SPN, CD8B | 39 | 36 | 19478 | 2.77E+01 | 1.00E+00 | 7.06E-01 | 6.84E-01 |
| GOTERM_BP_DIRECT | GO:0050832~defense response to fungus | 2 | 5.13 | 6.79E-02 | S100A9, S100A8 | 39 | 36 | 19478 | 2.77E+01 | 1.00E+00 | 7.06E-01 | 6.84E-01 |
| GOTERM_BP_DIRECT | GO:2001244~positive regulation of intrinsic apoptotic signaling pathway | 2 | 5.13 | 6.79E-02 | S100A9, S100A8 | 39 | 36 | 19478 | 2.77E+01 | 1.00E+00 | 7.06E-01 | 6.84E-01 |
| GOTERM_MF_DIRECT | GO:0051015~actin filament binding | 3 | 7.69 | 6.82E-02 | TLN1, MYO1F, TTN | 39 | 217 | 19253 | 6.82E+00 | 1.00E+00 | 6.14E-01 | 6.10E-01 |
| GOTERM_BP_DIRECT | GO:0050778~positive regulation of immune response | 2 | 5.13 | 6.98E-02 | HLA-DRA, HLA-DRB1 | 39 | 37 | 19478 | 2.70E+01 | 1.00E+00 | 7.06E-01 | 6.84E-01 |
| GOTERM_BP_DIRECT | GO:0050870~positive regulation of T cell activation | 2 | 5.13 | 7.16E-02 | HLA-DRA, HLA-DRB1 | 39 | 38 | 19478 | 2.63E+01 | 1.00E+00 | 7.06E-01 | 6.84E-01 |
| GOTERM_BP_DIRECT | GO:0001916~positive regulation of T cell mediated cytotoxicity | 2 | 5.13 | 7.16E-02 | HLA-DRA, HLA-DRB1 | 39 | 38 | 19478 | 2.63E+01 | 1.00E+00 | 7.06E-01 | 6.84E-01 |
| KEGG_PATHWAY | hsa05152:Tuberculosis | 3 | 7.69 | 7.29E-02 | HLA-DRA, CTSS, HLA-DRB1 | 22 | 182 | 8534 | 6.39E+00 | 9.99E-01 | 4.08E-01 | 3.80E-01 |
| UP_SEQ_FEATURE | DOMAIN:Ig-like 1 | 2 | 5.13 | 7.55E-02 | IGHM, TTN | 37 | 45 | 20675 | 2.48E+01 | 1.00E+00 | 1.00E+00 | 9.95E-01 |
| UP_SEQ_FEATURE | DOMAIN:Ig-like 2 | 2 | 5.13 | 7.55E-02 | IGHM, TTN | 37 | 45 | 20675 | 2.48E+01 | 1.00E+00 | 1.00E+00 | 9.95E-01 |
| SMART | SM01394:S_100 | 2 | 5.13 | 7.57E-02 | S100A9, S100A8 | 31 | 28 | 10706 | 2.47E+01 | 9.60E-01 | 7.75E-01 | 7.75E-01 |
| KEGG_PATHWAY | hsa05310:Asthma | 2 | 5.13 | 7.59E-02 | HLA-DRA, HLA-DRB1 | 22 | 32 | 8534 | 2.42E+01 | 9.99E-01 | 4.08E-01 | 3.80E-01 |
| GOTERM_BP_DIRECT | GO:0043542~endothelial cell migration | 2 | 5.13 | 7.70E-02 | S100A9, S100A8 | 39 | 41 | 19478 | 2.44E+01 | 1.00E+00 | 7.39E-01 | 7.16E-01 |
| UP_KW_BIOLOGICAL_PROCESS | KW-0399~Innate immunity | 4 | 10.26 | 7.83E-02 | FCN1, S100A9, S100A8, KLRG1 | 28 | 431 | 11523 | 3.82E+00 | 8.20E-01 | 4.11E-01 | 3.92E-01 |
| INTERPRO | IPR011162:MHC_I/II-like_Ag-recog | 2 | 5.13 | 8.03E-02 | HLA-DRA, HLA-DRB1 | 38 | 47 | 20808 | 2.33E+01 | 1.00E+00 | 9.81E-01 | 9.70E-01 |
| GOTERM_CC_DIRECT | GO:0000307~cyclin-dependent protein kinase holoenzyme complex | 2 | 5.13 | 8.08E-02 | CDK6, CCND2 | 39 | 46 | 20795 | 2.32E+01 | 1.00E+00 | 4.23E-01 | 3.78E-01 |
| GOTERM_MF_DIRECT | GO:0140662~ATP-dependent protein folding chaperone | 2 | 5.13 | 8.15E-02 | HSPA5, HSP90B1 | 39 | 43 | 19253 | 2.30E+01 | 1.00E+00 | 6.93E-01 | 6.88E-01 |
| GOTERM_BP_DIRECT | GO:0071526~semaphorin-plexin signaling pathway | 2 | 5.13 | 8.24E-02 | NELL2, TYROBP | 39 | 44 | 19478 | 2.27E+01 | 1.00E+00 | 7.70E-01 | 7.46E-01 |
| GOTERM_CC_DIRECT | GO:0001772~immunological synapse | 2 | 5.13 | 8.41E-02 | HLA-DRA, HLA-DRB1 | 39 | 48 | 20795 | 2.22E+01 | 1.00E+00 | 4.23E-01 | 3.78E-01 |
| GOTERM_CC_DIRECT | GO:0030658~transport vesicle membrane | 2 | 5.13 | 8.58E-02 | HLA-DRA, HLA-DRB1 | 39 | 49 | 20795 | 2.18E+01 | 1.00E+00 | 4.23E-01 | 3.78E-01 |
| BIOCARTA | h_ctla4Pathway:The Co-Stimulatory Signal During T-cell Activation | 2 | 5.13 | 9.01E-02 | HLA-DRA, HLA-DRB1 | 9 | 19 | 1622 | 1.90E+01 | 9.22E-01 | 2.70E-01 | 2.70E-01 |
| GOTERM_MF_DIRECT | GO:0042605~peptide antigen binding | 2 | 5.13 | 9.06E-02 | HLA-DRA, HLA-DRB1 | 39 | 48 | 19253 | 2.06E+01 | 1.00E+00 | 7.29E-01 | 7.25E-01 |
| KEGG_PATHWAY | hsa05330:Allograft rejection | 2 | 5.13 | 9.18E-02 | HLA-DRA, HLA-DRB1 | 22 | 39 | 8534 | 1.99E+01 | 1.00E+00 | 4.64E-01 | 4.32E-01 |
| GOTERM_BP_DIRECT | GO:0042130~negative regulation of T cell proliferation | 2 | 5.13 | 9.31E-02 | SPN, HLA-DRB1 | 39 | 50 | 19478 | 2.00E+01 | 1.00E+00 | 8.48E-01 | 8.22E-01 |
| UP_SEQ_FEATURE | MOTIF:Prevents secretion from ER | 2 | 5.13 | 9.47E-02 | HSPA5, HSP90B1 | 37 | 57 | 20675 | 1.96E+01 | 1.00E+00 | 1.00E+00 | 9.95E-01 |
| GOTERM_BP_DIRECT | GO:0051603~proteolysis involved in protein catabolic process | 2 | 5.13 | 9.84E-02 | HSPA5, CTSS | 39 | 53 | 19478 | 1.88E+01 | 1.00E+00 | 8.74E-01 | 8.47E-01 |
