# Supplementary material for: Single-cell RNA sequencing unraveled immune-related expression heterogeneity and lymphoid cell development dysregulation in childhood asthma
Source: Front Immunol. 2026 Jan 2;16:1606650. doi: 10.3389/fimmu.2025.1606650 (PMC12807962; doi:10.3389/fimmu.2025.1606650)
Supplement: Supplementary file 6 [file Table5.docx]

**Supplementary Table 5.** GO results of 37 upregulated genes in CD4 T cells of Asthma 3 paitent

| Category | Term | Count | % | *P-*Value | Genes | List Total | Pop Hits | Pop Total | Fold Enrichment | Bonferroni | Benjamini | FDR |
| --- | --- | --- | --- | --- | --- | --- | --- | --- | --- | --- | --- | --- |
| GOTERM_CC_DIRECT | GO:0005576~extracellular region | 26 | 70.27 | 2.12E-16 | FCN1, HBB, IGHV1-46, RETN, IFI30, CTSS, JCHAIN, IGLV2-8, CST3, HEBP2, IGLV2-14, IGKV1-12, S100A12, CAMP, TXNDC5, ANXA2, IGHV3-15, LYZ, VCAN, IGLV2-23, IGKV4-1, S100A4, MNDA, S100A9, S100A8, IGKV3-20 | 37 | 2313 | 20795 | 6.32E+00 | 2.46E-14 | 2.35E-14 | 2.03E-14 |
| UP_KW_BIOLOGICAL_PROCESS | KW-0391~Immunity | 17 | 45.95 | 3.05E-13 | FCN1, FCER1G, IGHV3-15, IGHV1-46, IFI30, IGLV2-8, TYROBP, IGLV2-14, IGLV2-23, IGKV1-12, IGKV4-1, S100A12, HLA-DRA, S100A9, S100A8, IGKV3-20, CAMP | 23 | 980 | 11523 | 8.69E+00 | 4.88E-12 | 4.88E-12 | 4.58E-12 |
| UP_KW_CELLULAR_COMPONENT | KW-0964~Secreted | 22 | 59.46 | 6.04E-11 | FCN1, ANXA2, IGHV3-15, IGHV1-46, RETN, IFI30, LYZ, CTSS, JCHAIN, IGLV2-8, CST3, VCAN, IGLV2-14, IGLV2-23, IGKV1-12, IGKV4-1, S100A12, S100A4, S100A9, S100A8, IGKV3-20, CAMP | 37 | 2217 | 18049 | 4.84E+00 | 1.15E-09 | 1.15E-09 | 1.15E-09 |
| GOTERM_MF_DIRECT | GO:0003823~antigen binding | 9 | 24.32 | 9.89E-11 | IGLV2-8, FCN1, IGLV2-14, IGLV2-23, IGKV4-1, IGHV3-15, IGHV1-46, IGKV3-20, JCHAIN | 35 | 140 | 19253 | 3.54E+01 | 9.89E-09 | 9.89E-09 | 9.49E-09 |
| UP_SEQ_FEATURE | REGION:Complementarity-determining-3 | 8 | 21.62 | 3.78E-09 | IGLV2-8, IGLV2-14, IGLV2-23, IGKV1-12, IGKV4-1, IGHV3-15, IGHV1-46, IGKV3-20 | 37 | 139 | 20675 | 3.22E+01 | 7.83E-07 | 1.51E-07 | 1.48E-07 |
| UP_SEQ_FEATURE | REGION:Framework-1 | 8 | 21.62 | 4.18E-09 | IGLV2-8, IGLV2-14, IGLV2-23, IGKV1-12, IGKV4-1, IGHV3-15, IGHV1-46, IGKV3-20 | 37 | 141 | 20675 | 3.17E+01 | 8.65E-07 | 1.51E-07 | 1.48E-07 |
| UP_SEQ_FEATURE | REGION:Complementarity-determining-1 | 8 | 21.62 | 4.39E-09 | IGLV2-8, IGLV2-14, IGLV2-23, IGKV1-12, IGKV4-1, IGHV3-15, IGHV1-46, IGKV3-20 | 37 | 142 | 20675 | 3.15E+01 | 9.09E-07 | 1.51E-07 | 1.48E-07 |
| UP_SEQ_FEATURE | REGION:Framework-3 | 8 | 21.62 | 4.39E-09 | IGLV2-8, IGLV2-14, IGLV2-23, IGKV1-12, IGKV4-1, IGHV3-15, IGHV1-46, IGKV3-20 | 37 | 142 | 20675 | 3.15E+01 | 9.09E-07 | 1.51E-07 | 1.48E-07 |
| UP_SEQ_FEATURE | REGION:Complementarity-determining-2 | 8 | 21.62 | 4.39E-09 | IGLV2-8, IGLV2-14, IGLV2-23, IGKV1-12, IGKV4-1, IGHV3-15, IGHV1-46, IGKV3-20 | 37 | 142 | 20675 | 3.15E+01 | 9.09E-07 | 1.51E-07 | 1.48E-07 |
| UP_SEQ_FEATURE | REGION:Framework-2 | 8 | 21.62 | 4.39E-09 | IGLV2-8, IGLV2-14, IGLV2-23, IGKV1-12, IGKV4-1, IGHV3-15, IGHV1-46, IGKV3-20 | 37 | 142 | 20675 | 3.15E+01 | 9.09E-07 | 1.51E-07 | 1.48E-07 |
| GOTERM_BP_DIRECT | GO:0006955~immune response | 11 | 29.73 | 8.72E-09 | IGLV2-8, CST3, IGLV2-14, IGLV2-23, IGKV1-12, IGKV4-1, HLA-DRA, IGHV1-46, IGKV3-20, CTSS, JCHAIN | 33 | 537 | 19478 | 1.21E+01 | 2.48E-06 | 2.48E-06 | 2.33E-06 |
| GOTERM_CC_DIRECT | GO:0019814~immunoglobulin complex | 8 | 21.62 | 3.66E-08 | IGLV2-8, IGLV2-14, IGLV2-23, IGKV1-12, IGKV4-1, IGHV3-15, IGHV1-46, IGKV3-20 | 37 | 194 | 20795 | 2.32E+01 | 4.07E-06 | 1.44E-06 | 1.24E-06 |
| GOTERM_CC_DIRECT | GO:0070062~extracellular exosome | 18 | 48.65 | 3.88E-08 | ANXA2, AHNAK, ALYREF, HBB, RETN, LYZ, JCHAIN, CST3, HEBP2, IGLV2-14, HLA-DRA, S100A4, MNDA, S100A9, S100A8, IGKV3-20, CAMP, TXNDC5 | 37 | 2242 | 20795 | 4.51E+00 | 4.31E-06 | 1.44E-06 | 1.24E-06 |
| GOTERM_BP_DIRECT | GO:0002250~adaptive immune response | 10 | 27.03 | 6.51E-08 | IGLV2-8, IGLV2-14, IGLV2-23, IGKV1-12, IGKV4-1, HLA-DRA, IGHV1-46, IGKV3-20, CTSS, JCHAIN | 33 | 492 | 19478 | 1.20E+01 | 1.85E-05 | 9.24E-06 | 8.69E-06 |
| UP_KW_CELLULAR_COMPONENT | KW-1280~Immunoglobulin | 8 | 21.62 | 8.88E-08 | IGLV2-8, IGLV2-14, IGLV2-23, IGKV1-12, IGKV4-1, IGHV3-15, IGHV1-46, IGKV3-20 | 37 | 192 | 18049 | 2.03E+01 | 1.69E-06 | 8.44E-07 | 8.44E-07 |
| GOTERM_CC_DIRECT | GO:0035578~azurophil granule lumen | 6 | 16.22 | 4.86E-07 | HEBP2, ANXA2, MNDA, RETN, LYZ, TXNDC5 | 37 | 91 | 20795 | 3.71E+01 | 5.40E-05 | 1.35E-05 | 1.17E-05 |
| INTERPRO | IPR050150:IgV_Light_Chain | 6 | 16.22 | 5.41E-07 | IGLV2-8, IGLV2-14, IGLV2-23, IGKV1-12, IGKV4-1, IGKV3-20 | 37 | 93 | 20808 | 3.63E+01 | 6.00E-05 | 6.00E-05 | 5.73E-05 |
| GOTERM_MF_DIRECT | GO:0050786~RAGE receptor binding | 4 | 10.81 | 8.22E-07 | S100A12, S100A4, S100A9, S100A8 | 35 | 11 | 19253 | 2.00E+02 | 8.22E-05 | 4.11E-05 | 3.95E-05 |
| INTERPRO | IPR013106:Ig_V-set | 9 | 24.32 | 1.40E-06 | IGLV2-8, VCAN, IGLV2-14, IGLV2-23, IGKV1-12, IGKV4-1, IGHV3-15, IGHV1-46, IGKV3-20 | 37 | 485 | 20808 | 1.04E+01 | 1.56E-04 | 7.78E-05 | 7.43E-05 |
| UP_SEQ_FEATURE | DOMAIN:Ig-like | 10 | 27.03 | 1.67E-06 | IGLV2-8, VCAN, IGLV2-14, IGLV2-23, IGKV1-12, IGKV4-1, HLA-DRA, IGHV3-15, IGHV1-46, IGKV3-20 | 37 | 673 | 20675 | 8.30E+00 | 3.45E-04 | 4.92E-05 | 4.81E-05 |
| GOTERM_CC_DIRECT | GO:1904724~tertiary granule lumen | 5 | 13.51 | 2.61E-06 | CST3, HBB, LYZ, CTSS, CAMP | 37 | 56 | 20795 | 5.02E+01 | 2.89E-04 | 5.78E-05 | 5.00E-05 |
| UP_KW_BIOLOGICAL_PROCESS | KW-1064~Adaptive immunity | 9 | 24.32 | 3.02E-06 | IGLV2-8, IGLV2-14, IGLV2-23, IGKV1-12, IGKV4-1, HLA-DRA, IGHV3-15, IGHV1-46, IGKV3-20 | 23 | 521 | 11523 | 8.65E+00 | 4.83E-05 | 2.41E-05 | 2.26E-05 |
| INTERPRO | IPR007110:Ig-like_dom | 10 | 27.03 | 4.66E-06 | IGLV2-8, VCAN, IGLV2-14, IGLV2-23, IGKV1-12, IGKV4-1, HLA-DRA, IGHV3-15, IGHV1-46, IGKV3-20 | 37 | 768 | 20808 | 7.32E+00 | 5.17E-04 | 1.72E-04 | 1.65E-04 |
| UP_KW_LIGAND | KW-0106~Calcium | 9 | 24.32 | 5.46E-06 | FCN1, VCAN, TYROBP, ANXA2, EFHD2, S100A12, S100A4, S100A9, S100A8 | 11 | 990 | 6987 | 5.77E+00 | 5.46E-05 | 5.46E-05 | 5.46E-05 |
| GOTERM_CC_DIRECT | GO:0005615~extracellular space | 14 | 37.84 | 7.57E-06 | FCN1, ANXA2, HBB, RETN, LYZ, CTSS, JCHAIN, CST3, VCAN, S100A4, S100A9, S100A8, IGKV3-20, CAMP | 37 | 1867 | 20795 | 4.21E+00 | 8.40E-04 | 1.40E-04 | 1.21E-04 |
| SMART | SM00406:IGv | 8 | 21.62 | 8.00E-06 | IGLV2-8, IGLV2-14, IGLV2-23, IGKV1-12, IGKV4-1, IGHV3-15, IGHV1-46, IGKV3-20 | 26 | 334 | 10706 | 9.86E+00 | 2.00E-04 | 2.00E-04 | 1.92E-04 |
| GOTERM_BP_DIRECT | GO:0042742~defense response to bacterium | 6 | 16.22 | 8.17E-06 | FCER1G, S100A12, LYZ, S100A9, CAMP, S100A8 | 33 | 171 | 19478 | 2.07E+01 | 2.32E-03 | 7.74E-04 | 7.28E-04 |
| INTERPRO | IPR001751:S100/CaBP7/8-like_CS | 4 | 10.81 | 1.07E-05 | S100A12, S100A4, S100A9, S100A8 | 37 | 25 | 20808 | 9.00E+01 | 1.18E-03 | 2.80E-04 | 2.67E-04 |
| GOTERM_MF_DIRECT | GO:0048306~calcium-dependent protein binding | 5 | 13.51 | 1.29E-05 | ANXA2, S100A12, S100A4, S100A9, S100A8 | 35 | 82 | 19253 | 3.35E+01 | 1.29E-03 | 4.29E-04 | 4.11E-04 |
| INTERPRO | IPR036179:Ig-like_dom_sf | 10 | 27.03 | 1.30E-05 | IGLV2-8, VCAN, IGLV2-14, IGLV2-23, IGKV1-12, IGKV4-1, HLA-DRA, IGHV3-15, IGHV1-46, IGKV3-20 | 37 | 872 | 20808 | 6.45E+00 | 1.44E-03 | 2.80E-04 | 2.67E-04 |
| UP_KW_MOLECULAR_FUNCTION | KW-0929~Antimicrobial | 5 | 13.51 | 1.40E-05 | S100A12, LYZ, S100A9, CAMP, S100A8 | 17 | 116 | 11952 | 3.03E+01 | 3.22E-04 | 3.22E-04 | 3.22E-04 |
| INTERPRO | IPR013787:S100_Ca-bd_sub | 4 | 10.81 | 1.51E-05 | S100A12, S100A4, S100A9, S100A8 | 37 | 28 | 20808 | 8.03E+01 | 1.68E-03 | 2.80E-04 | 2.67E-04 |
| GOTERM_BP_DIRECT | GO:0019886~antigen processing and presentation of exogenous peptide antigen via MHC class II | 4 | 10.81 | 1.75E-05 | FCER1G, HLA-DRA, IFI30, CTSS | 33 | 31 | 19478 | 7.62E+01 | 4.97E-03 | 1.25E-03 | 1.17E-03 |
| SMART | SM01394:S_100 | 4 | 10.81 | 3.55E-05 | S100A12, S100A4, S100A9, S100A8 | 26 | 28 | 10706 | 5.88E+01 | 8.86E-04 | 4.43E-04 | 4.25E-04 |
| UP_KW_PTM | KW-1015~Disulfide bond | 21 | 56.76 | 3.90E-05 | FCN1, FCER1G, IGHV3-15, IGHV1-46, RETN, IFI30, LYZ, CTSS, JCHAIN, IGLV2-8, CST3, VCAN, TYROBP, IGLV2-14, IGLV2-23, IGKV1-12, IGKV4-1, HLA-DRA, IGKV3-20, CAMP, TXNDC5 | 33 | 3956 | 14316 | 2.30E+00 | 5.07E-04 | 5.46E-04 | 5.46E-04 |
| GOTERM_BP_DIRECT | GO:0043542~endothelial cell migration | 4 | 10.81 | 4.12E-05 | ZEB2, S100A12, S100A9, S100A8 | 33 | 41 | 19478 | 5.76E+01 | 1.16E-02 | 2.34E-03 | 2.20E-03 |
| GOTERM_CC_DIRECT | GO:0062023~collagen-containing extracellular matrix | 7 | 18.92 | 4.85E-05 | FCN1, VCAN, ANXA2, S100A4, S100A9, CTSS, S100A8 | 37 | 387 | 20795 | 1.02E+01 | 5.37E-03 | 7.70E-04 | 6.66E-04 |
| GOTERM_CC_DIRECT | GO:1904813~ficolin-1-rich granule lumen | 5 | 13.51 | 6.51E-05 | FCN1, CST3, HBB, MNDA, CTSS | 37 | 126 | 20795 | 2.23E+01 | 7.20E-03 | 8.68E-04 | 7.51E-04 |
| GOTERM_CC_DIRECT | GO:0005886~plasma membrane | 22 | 59.46 | 7.04E-05 | FCN1, CDV3, FCER1G, ANXA2, AHNAK, BRI3, NKG7, IGHV3-15, IGHV1-46, IGLV2-8, CST3, ZEB2, TYROBP, IGLV2-14, IGLV2-23, IGKV1-12, IGKV4-1, S100A12, HLA-DRA, S100A9, S100A8, IGKV3-20 | 37 | 5597 | 20795 | 2.21E+00 | 7.78E-03 | 8.68E-04 | 7.51E-04 |
| UP_KW_DOMAIN | KW-0732~Signal | 22 | 59.46 | 7.21E-05 | FCN1, FCER1G, HBB, IGHV3-15, IGHV1-46, RETN, IFI30, LYZ, CTSS, JCHAIN, IGLV2-8, CST3, VCAN, TYROBP, IGLV2-14, IGLV2-23, IGKV1-12, IGKV4-1, HLA-DRA, IGKV3-20, CAMP, TXNDC5 | 34 | 4415 | 14625 | 2.14E+00 | 9.36E-04 | 9.37E-04 | 9.37E-04 |
| INTERPRO | IPR013783:Ig-like_fold | 10 | 27.03 | 9.69E-05 | IGLV2-8, VCAN, IGLV2-14, IGLV2-23, IGKV1-12, IGKV4-1, HLA-DRA, IGHV3-15, IGHV1-46, IGKV3-20 | 37 | 1127 | 20808 | 4.99E+00 | 1.07E-02 | 1.54E-03 | 1.47E-03 |
| GOTERM_BP_DIRECT | GO:0002544~chronic inflammatory response | 3 | 8.11 | 1.17E-04 | S100A9, CAMP, S100A8 | 33 | 10 | 19478 | 1.77E+02 | 3.26E-02 | 4.99E-03 | 4.69E-03 |
| GOTERM_BP_DIRECT | GO:0030593~neutrophil chemotaxis | 4 | 10.81 | 1.23E-04 | FCER1G, S100A12, S100A9, S100A8 | 33 | 59 | 19478 | 4.00E+01 | 3.43E-02 | 4.99E-03 | 4.69E-03 |
| INTERPRO | IPR003599:Ig_sub | 7 | 18.92 | 2.73E-04 | IGLV2-8, VCAN, IGLV2-14, IGLV2-23, IGKV1-12, IGKV4-1, IGKV3-20 | 37 | 531 | 20808 | 7.41E+00 | 2.98E-02 | 3.78E-03 | 3.61E-03 |
| UP_SEQ_FEATURE | DOMAIN:EF-hand 1 | 5 | 13.51 | 3.11E-04 | EFHD2, S100A12, S100A4, S100A9, S100A8 | 37 | 188 | 20675 | 1.49E+01 | 6.23E-02 | 7.44E-03 | 7.26E-03 |
| UP_SEQ_FEATURE | DOMAIN:EF-hand 2 | 5 | 13.51 | 3.23E-04 | EFHD2, S100A12, S100A4, S100A9, S100A8 | 37 | 190 | 20675 | 1.47E+01 | 6.48E-02 | 7.44E-03 | 7.26E-03 |
| UP_KW_DOMAIN | KW-0393~Immunoglobulin domain | 9 | 24.32 | 3.89E-04 | IGLV2-8, VCAN, IGLV2-14, IGLV2-23, IGKV1-12, IGKV4-1, IGHV3-15, IGHV1-46, IGKV3-20 | 34 | 825 | 14625 | 4.69E+00 | 5.05E-03 | 2.53E-03 | 2.53E-03 |
| GOTERM_BP_DIRECT | GO:0006954~inflammatory response | 6 | 16.22 | 6.44E-04 | NKG7, S100A12, FOS, LYZ, S100A9, S100A8 | 33 | 432 | 19478 | 8.20E+00 | 1.67E-01 | 2.29E-02 | 2.15E-02 |
| GOTERM_CC_DIRECT | GO:0043202~lysosomal lumen | 4 | 10.81 | 6.47E-04 | VCAN, IFI30, CTSS, TXNDC5 | 37 | 98 | 20795 | 2.29E+01 | 6.94E-02 | 7.19E-03 | 6.21E-03 |
| INTERPRO | IPR002048:EF_hand_dom | 5 | 13.51 | 6.81E-04 | EFHD2, S100A12, S100A4, S100A9, S100A8 | 37 | 233 | 20808 | 1.21E+01 | 7.28E-02 | 8.40E-03 | 8.02E-03 |
| GOTERM_BP_DIRECT | GO:0050729~positive regulation of inflammatory response | 4 | 10.81 | 8.13E-04 | NKG7, S100A12, S100A9, S100A8 | 33 | 112 | 19478 | 2.11E+01 | 2.06E-01 | 2.57E-02 | 2.41E-02 |
| GOTERM_CC_DIRECT | GO:0034774~secretory granule lumen | 4 | 10.81 | 1.11E-03 | FCN1, S100A12, S100A9, S100A8 | 37 | 118 | 20795 | 1.91E+01 | 1.16E-01 | 1.12E-02 | 9.68E-03 |
| UP_KW_BIOLOGICAL_PROCESS | KW-0399~Innate immunity | 6 | 16.22 | 1.11E-03 | FCN1, FCER1G, S100A12, S100A9, CAMP, S100A8 | 23 | 431 | 11523 | 6.97E+00 | 1.76E-02 | 5.93E-03 | 5.56E-03 |
| SMART | SM00409:IG | 7 | 18.92 | 1.14E-03 | IGLV2-8, VCAN, IGLV2-14, IGLV2-23, IGKV1-12, IGKV4-1, IGKV3-20 | 26 | 531 | 10706 | 5.43E+00 | 2.82E-02 | 9.52E-03 | 9.14E-03 |
| INTERPRO | IPR011992:EF-hand-dom_pair | 5 | 13.51 | 1.28E-03 | EFHD2, S100A12, S100A4, S100A9, S100A8 | 37 | 276 | 20808 | 1.02E+01 | 1.32E-01 | 1.42E-02 | 1.35E-02 |
| UP_KW_PTM | KW-0873~Pyrrolidone carboxylic acid | 4 | 10.81 | 1.50E-03 | IGLV2-8, IGLV2-14, IGLV2-23, JCHAIN | 33 | 102 | 14316 | 1.70E+01 | 1.93E-02 | 1.05E-02 | 1.05E-02 |
| GOTERM_BP_DIRECT | GO:0050832~defense response to fungus | 3 | 8.11 | 1.59E-03 | S100A12, S100A9, S100A8 | 33 | 36 | 19478 | 4.92E+01 | 3.64E-01 | 4.52E-02 | 4.25E-02 |
| GOTERM_MF_DIRECT | GO:0005509~calcium ion binding | 7 | 18.92 | 1.68E-03 | VCAN, ANXA2, EFHD2, S100A12, S100A4, S100A9, S100A8 | 35 | 739 | 19253 | 5.21E+00 | 1.55E-01 | 4.20E-02 | 4.03E-02 |
| GOTERM_CC_DIRECT | GO:0072562~blood microparticle | 4 | 10.81 | 2.12E-03 | IGKV4-1, HBB, IGKV3-20, JCHAIN | 37 | 148 | 20795 | 1.52E+01 | 2.10E-01 | 1.96E-02 | 1.70E-02 |
| GOTERM_BP_DIRECT | GO:0045087~innate immune response | 6 | 16.22 | 2.21E-03 | FCER1G, S100A12, S100A9, CAMP, S100A8, JCHAIN | 33 | 570 | 19478 | 6.21E+00 | 4.66E-01 | 5.70E-02 | 5.36E-02 |
| GOTERM_CC_DIRECT | GO:0031982~vesicle | 4 | 10.81 | 2.84E-03 | CST3, AHNAK, ANXA2, CAMP | 37 | 164 | 20795 | 1.37E+01 | 2.71E-01 | 2.43E-02 | 2.10E-02 |
| GOTERM_MF_DIRECT | GO:0042802~identical protein binding | 10 | 27.03 | 2.97E-03 | CST3, TYROBP, FCER1G, AHNAK, ANXA2, BRI3, S100A12, S100A4, FOS, LYZ | 35 | 1777 | 19253 | 3.10E+00 | 2.57E-01 | 5.94E-02 | 5.70E-02 |
| GOTERM_BP_DIRECT | GO:0070488~neutrophil aggregation | 2 | 5.41 | 3.28E-03 | S100A9, S100A8 | 33 | 2 | 19478 | 5.90E+02 | 6.07E-01 | 7.77E-02 | 7.30E-02 |
| GOTERM_CC_DIRECT | GO:1990660~calprotectin complex | 2 | 5.41 | 3.46E-03 | S100A9, S100A8 | 37 | 2 | 20795 | 5.62E+02 | 3.19E-01 | 2.74E-02 | 2.37E-02 |
| GOTERM_CC_DIRECT | GO:0071756~pentameric IgM immunoglobulin complex | 2 | 5.41 | 5.18E-03 | IGKV3-20, JCHAIN | 37 | 3 | 20795 | 3.75E+02 | 4.38E-01 | 3.69E-02 | 3.20E-02 |
| KEGG_PATHWAY | hsa05152:Tuberculosis | 4 | 10.81 | 5.20E-03 | FCER1G, HLA-DRA, CTSS, CAMP | 18 | 182 | 8534 | 1.04E+01 | 3.52E-01 | 3.23E-01 | 3.23E-01 |
| GOTERM_CC_DIRECT | GO:0035580~specific granule lumen | 3 | 8.11 | 5.33E-03 | RETN, LYZ, CAMP | 37 | 63 | 20795 | 2.68E+01 | 4.47E-01 | 3.69E-02 | 3.20E-02 |
| UP_KW_BIOLOGICAL_PROCESS | KW-0395~Inflammatory response | 4 | 10.81 | 5.55E-03 | NKG7, S100A12, S100A9, S100A8 | 23 | 192 | 11523 | 1.04E+01 | 8.53E-02 | 2.22E-02 | 2.08E-02 |
| GOTERM_BP_DIRECT | GO:1905686~positive regulation of plasma membrane repair | 2 | 5.41 | 6.56E-03 | AHNAK, ANXA2 | 33 | 4 | 19478 | 2.95E+02 | 8.46E-01 | 1.40E-01 | 1.32E-01 |
| GOTERM_CC_DIRECT | GO:0071748~monomeric IgA immunoglobulin complex | 2 | 5.41 | 6.91E-03 | IGKV3-20, JCHAIN | 37 | 4 | 20795 | 2.81E+02 | 5.37E-01 | 4.51E-02 | 3.90E-02 |
| GOTERM_BP_DIRECT | GO:0019731~antibacterial humoral response | 3 | 8.11 | 6.91E-03 | IGKV3-20, CAMP, JCHAIN | 33 | 76 | 19478 | 2.33E+01 | 8.60E-01 | 1.40E-01 | 1.32E-01 |
| GOTERM_MF_DIRECT | GO:0035662~Toll-like receptor 4 binding | 2 | 5.41 | 7.05E-03 | S100A9, S100A8 | 35 | 4 | 19253 | 2.75E+02 | 5.07E-01 | 1.17E-01 | 1.13E-01 |
| GOTERM_BP_DIRECT | GO:0035821~modulation of process of another organism | 2 | 5.41 | 8.19E-03 | S100A9, CAMP | 33 | 5 | 19478 | 2.36E+02 | 9.03E-01 | 1.45E-01 | 1.37E-01 |
| GOTERM_BP_DIRECT | GO:0002376~immune system process | 3 | 8.11 | 8.19E-03 | TYROBP, FCER1G, IFI30 | 33 | 83 | 19478 | 2.13E+01 | 9.03E-01 | 1.45E-01 | 1.37E-01 |
| GOTERM_CC_DIRECT | GO:0071751~secretory IgA immunoglobulin complex | 2 | 5.41 | 8.63E-03 | IGKV3-20, JCHAIN | 37 | 5 | 20795 | 2.25E+02 | 6.18E-01 | 5.32E-02 | 4.60E-02 |
| UP_KW_CELLULAR_COMPONENT | KW-1003~Cell membrane | 16 | 43.24 | 9.31E-03 | FCN1, FCER1G, NKG7, IGHV3-15, IGHV1-46, IGLV2-8, TYROBP, IGLV2-14, IGLV2-23, IGKV1-12, IGKV4-1, S100A12, HLA-DRA, S100A9, S100A8, IGKV3-20 | 37 | 4134 | 18049 | 1.89E+00 | 1.63E-01 | 5.10E-02 | 5.10E-02 |
| UP_KW_PTM | KW-0702~S-nitrosylation | 3 | 8.11 | 1.01E-02 | HBB, S100A9, S100A8 | 33 | 68 | 14316 | 1.91E+01 | 1.23E-01 | 4.69E-02 | 4.69E-02 |
| GOTERM_BP_DIRECT | GO:0031640~killing of cells of another organism | 3 | 8.11 | 1.02E-02 | NKG7, S100A12, LYZ | 33 | 93 | 19478 | 1.90E+01 | 9.45E-01 | 1.70E-01 | 1.60E-01 |
| UP_KW_CELLULAR_COMPONENT | KW-0458~Lysosome | 5 | 13.51 | 1.07E-02 | BRI3, NKG7, HLA-DRA, IFI30, CTSS | 37 | 436 | 18049 | 5.59E+00 | 1.86E-01 | 5.10E-02 | 5.10E-02 |
| KEGG_PATHWAY | hsa04612:Antigen processing and presentation | 3 | 8.11 | 1.10E-02 | HLA-DRA, IFI30, CTSS | 18 | 81 | 8534 | 1.76E+01 | 6.02E-01 | 3.23E-01 | 3.23E-01 |
| GOTERM_BP_DIRECT | GO:0042590~antigen processing and presentation of exogenous peptide antigen via MHC class I | 2 | 5.41 | 1.14E-02 | FCER1G, IFI30 | 33 | 7 | 19478 | 1.69E+02 | 9.62E-01 | 1.71E-01 | 1.61E-01 |
| GOTERM_BP_DIRECT | GO:0035425~autocrine signaling | 2 | 5.41 | 1.14E-02 | S100A9, S100A8 | 33 | 7 | 19478 | 1.69E+02 | 9.62E-01 | 1.71E-01 | 1.61E-01 |
| GOTERM_MF_DIRECT | GO:0050544~arachidonate binding | 2 | 5.41 | 1.23E-02 | S100A9, S100A8 | 35 | 7 | 19253 | 1.57E+02 | 7.10E-01 | 1.76E-01 | 1.69E-01 |
| GOTERM_BP_DIRECT | GO:0016064~immunoglobulin mediated immune response | 3 | 8.11 | 1.31E-02 | FCER1G, IGHV3-15, IGHV1-46 | 33 | 106 | 19478 | 1.67E+01 | 9.76E-01 | 1.86E-01 | 1.75E-01 |
| GOTERM_BP_DIRECT | GO:0002283~neutrophil activation involved in immune response | 2 | 5.41 | 1.47E-02 | TYROBP, FCER1G | 33 | 9 | 19478 | 1.31E+02 | 9.85E-01 | 1.99E-01 | 1.87E-01 |
| KEGG_PATHWAY | hsa04657:IL-17 signaling pathway | 3 | 8.11 | 1.50E-02 | FOS, S100A9, S100A8 | 18 | 95 | 8534 | 1.50E+01 | 7.14E-01 | 3.23E-01 | 3.23E-01 |
| KEGG_PATHWAY | hsa04970:Salivary secretion | 3 | 8.11 | 1.56E-02 | CST3, LYZ, CAMP | 18 | 97 | 8534 | 1.47E+01 | 7.28E-01 | 3.23E-01 | 3.23E-01 |
| GOTERM_BP_DIRECT | GO:0051092~positive regulation of NF-kappaB transcription factor activity | 3 | 8.11 | 1.65E-02 | S100A12, S100A9, S100A8 | 33 | 120 | 19478 | 1.48E+01 | 9.91E-01 | 2.14E-01 | 2.01E-01 |
| UP_SEQ_FEATURE | DOMAIN:ITAM | 2 | 5.41 | 1.90E-02 | TYROBP, FCER1G | 37 | 11 | 20675 | 1.02E+02 | 9.81E-01 | 3.93E-01 | 3.84E-01 |
| GOTERM_BP_DIRECT | GO:0034121~regulation of toll-like receptor signaling pathway | 2 | 5.41 | 1.95E-02 | S100A9, S100A8 | 33 | 12 | 19478 | 9.84E+01 | 9.96E-01 | 2.41E-01 | 2.27E-01 |
| GOTERM_BP_DIRECT | GO:0061844~antimicrobial humoral immune response mediated by antimicrobial peptide | 3 | 8.11 | 2.06E-02 | S100A12, S100A9, CAMP | 33 | 135 | 19478 | 1.31E+01 | 9.97E-01 | 2.44E-01 | 2.30E-01 |
| GOTERM_BP_DIRECT | GO:0002523~leukocyte migration involved in inflammatory response | 2 | 5.41 | 2.28E-02 | S100A9, S100A8 | 33 | 14 | 19478 | 8.43E+01 | 9.99E-01 | 2.59E-01 | 2.43E-01 |
| GOTERM_MF_DIRECT | GO:0044548~S100 protein binding | 2 | 5.41 | 2.44E-02 | AHNAK, ANXA2 | 35 | 14 | 19253 | 7.86E+01 | 9.16E-01 | 3.06E-01 | 2.93E-01 |
| GOTERM_BP_DIRECT | GO:0014002~astrocyte development | 2 | 5.41 | 2.76E-02 | S100A9, S100A8 | 33 | 17 | 19478 | 6.94E+01 | 1.00E+00 | 3.01E-01 | 2.83E-01 |
| GOTERM_CC_DIRECT | GO:0098797~plasma membrane protein complex | 2 | 5.41 | 2.90E-02 | AHNAK, ANXA2 | 37 | 17 | 20795 | 6.61E+01 | 9.62E-01 | 1.70E-01 | 1.47E-01 |
| GOTERM_BP_DIRECT | GO:0030889~negative regulation of B cell proliferation | 2 | 5.41 | 2.92E-02 | TYROBP, MNDA | 33 | 18 | 19478 | 6.56E+01 | 1.00E+00 | 3.07E-01 | 2.89E-01 |
| GOTERM_BP_DIRECT | GO:0003094~glomerular filtration | 2 | 5.41 | 3.24E-02 | IGKV3-20, JCHAIN | 33 | 20 | 19478 | 5.90E+01 | 1.00E+00 | 3.28E-01 | 3.09E-01 |
| GOTERM_BP_DIRECT | GO:0051493~regulation of cytoskeleton organization | 2 | 5.41 | 3.87E-02 | S100A9, S100A8 | 33 | 24 | 19478 | 4.92E+01 | 1.00E+00 | 3.79E-01 | 3.56E-01 |
| UP_SEQ_FEATURE | DISULFID:Interchain | 3 | 8.11 | 3.87E-02 | TYROBP, FCER1G, RETN | 37 | 179 | 20675 | 9.37E+00 | 1.00E+00 | 7.29E-01 | 7.11E-01 |
| INTERPRO | IPR018247:EF_Hand_1_Ca_BS | 3 | 8.11 | 3.91E-02 | S100A4, S100A9, S100A8 | 37 | 181 | 20808 | 9.32E+00 | 9.88E-01 | 3.77E-01 | 3.60E-01 |
| UP_KW_DISEASE | KW-1008~Amyloidosis | 2 | 5.41 | 4.01E-02 | CST3, LYZ | 7 | 33 | 4859 | 4.21E+01 | 3.88E-01 | 4.81E-01 | 4.81E-01 |
| INTERPRO | IPR046350:Cystatin_sf | 2 | 5.41 | 4.07E-02 | CST3, CAMP | 37 | 24 | 20808 | 4.69E+01 | 9.90E-01 | 3.77E-01 | 3.60E-01 |
| SMART | SM00054:EFh | 3 | 8.11 | 4.68E-02 | EFHD2, S100A4, S100A9 | 26 | 149 | 10706 | 8.29E+00 | 6.99E-01 | 2.93E-01 | 2.81E-01 |
| UP_SEQ_FEATURE | DOMAIN:EF-hand | 3 | 8.11 | 4.77E-02 | EFHD2, S100A4, S100A9 | 37 | 201 | 20675 | 8.34E+00 | 1.00E+00 | 8.23E-01 | 8.04E-01 |
| UP_KW_CELLULAR_COMPONENT | KW-0034~Amyloid | 2 | 5.41 | 4.87E-02 | CST3, LYZ | 37 | 25 | 18049 | 3.90E+01 | 6.13E-01 | 1.85E-01 | 1.85E-01 |
| GOTERM_CC_DIRECT | GO:0045121~membrane raft | 3 | 8.11 | 5.15E-02 | AHNAK, ANXA2, EFHD2 | 37 | 211 | 20795 | 7.99E+00 | 9.97E-01 | 2.86E-01 | 2.47E-01 |
| GOTERM_BP_DIRECT | GO:2001244~positive regulation of intrinsic apoptotic signaling pathway | 2 | 5.41 | 5.75E-02 | S100A9, S100A8 | 33 | 36 | 19478 | 3.28E+01 | 1.00E+00 | 5.45E-01 | 5.12E-01 |
| KEGG_PATHWAY | hsa05310:Asthma | 2 | 5.41 | 6.19E-02 | FCER1G, HLA-DRA | 18 | 32 | 8534 | 2.96E+01 | 9.95E-01 | 1.00E+00 | 1.00E+00 |
| GOTERM_BP_DIRECT | GO:0019882~antigen processing and presentation | 2 | 5.41 | 7.14E-02 | HLA-DRA, CTSS | 33 | 45 | 19478 | 2.62E+01 | 1.00E+00 | 6.47E-01 | 6.08E-01 |
| GOTERM_BP_DIRECT | GO:0030316~osteoclast differentiation | 2 | 5.41 | 7.29E-02 | TYROBP, FOS | 33 | 46 | 19478 | 2.57E+01 | 1.00E+00 | 6.47E-01 | 6.08E-01 |
| GOTERM_BP_DIRECT | GO:0014823~response to activity | 2 | 5.41 | 8.05E-02 | ANXA2, FOS | 33 | 51 | 19478 | 2.31E+01 | 1.00E+00 | 6.93E-01 | 6.52E-01 |
| KEGG_PATHWAY | hsa05132:Salmonella infection | 3 | 8.11 | 8.77E-02 | AHNAK, ANXA2, FOS | 18 | 251 | 8534 | 5.67E+00 | 1.00E+00 | 1.00E+00 | 1.00E+00 |
| UP_SEQ_FEATURE | DISULFID:Redox-active | 2 | 5.41 | 8.83E-02 | IFI30, TXNDC5 | 37 | 53 | 20675 | 2.11E+01 | 1.00E+00 | 1.00E+00 | 9.81E-01 |
| GOTERM_BP_DIRECT | GO:0006968~cellular defense response | 2 | 5.41 | 8.96E-02 | TYROBP, MNDA | 33 | 57 | 19478 | 2.07E+01 | 1.00E+00 | 7.48E-01 | 7.04E-01 |
| BIOCARTA | h_fcer1Pathway:Fc Epsilon Receptor I Signaling in Mast Cells | 2 | 5.41 | 9.28E-02 | FCER1G, FOS | 5 | 39 | 1622 | 1.66E+01 | 9.83E-01 | 1.00E+00 | 1.00E+00 |
