# Supplementary material for: Single-cell RNA sequencing unraveled immune-related expression heterogeneity and lymphoid cell development dysregulation in childhood asthma
Source: Front Immunol. 2026 Jan 2;16:1606650. doi: 10.3389/fimmu.2025.1606650 (PMC12807962; doi:10.3389/fimmu.2025.1606650)
Supplement: Supplementary file 7 [file Table6.docx]

**Supplementary Table 6.** GO results of 84 upregulated genes in CD8 T cells of Asthma 1 paitent

| Category | Term | Count | % | *P-*Value | Genes | List Total | Pop Hits | Pop Total | Fold Enrichment | Bonferroni | Benjamini | FDR |
| --- | --- | --- | --- | --- | --- | --- | --- | --- | --- | --- | --- | --- |
| GOTERM_BP_DIRECT | GO:0006955~immune response | 38 | 46.91 | 4.85E-37 | IGHV3-23, IGLV3-1, IGHV4-39, CST7, CTSS, JCHAIN, IGLV2-11, CST3, IGLV3-9, FCGR3A, IGLV1-40, IGLV6-57, IGKC, IGLV2-14, IGLV3-21, CCL4, IGKV3-15, IGLV2-18, SLAMF7, TRAV14DV4, IGHA1, IGKV3-11, HLA-DQA2, HLA-DPA1, HLA-DRB5, FCRL3, GZMA, IGKV1-5, PPBP, TNFRSF1B, TGFBR3, IGLV4-69, HLA-DPB1, IGKV4-1, FAS, HLA-DRA, IGKV3-20, IGLV3-19 | 79 | 537 | 19478 | 1.74E+01 | 2.55E-34 | 2.55E-34 | 2.49E-34 |
| GOTERM_BP_DIRECT | GO:0002250~adaptive immune response | 31 | 38.27 | 5.31E-28 | TRBV4-2, IGLV3-1, IGHV4-39, CTSS, JCHAIN, IGLV2-11, IGLV3-9, IGLV1-40, IGLV6-57, IGKC, IGLV2-14, IGLV3-21, IGKV3-15, IGLV2-18, SLAMF7, TRAV14DV4, KLRC1, IGHA1, IGKV3-11, HLA-DQA2, HLA-DPA1, HLA-DRB5, TRDV2, IGKV1-5, IGLV4-69, HLA-DPB1, IGKV4-1, HLA-DRA, KLRD1, IGKV3-20, IGLV3-19 | 79 | 492 | 19478 | 1.55E+01 | 2.79E-25 | 1.39E-25 | 1.36E-25 |
| UP_KW_BIOLOGICAL_PROCESS | KW-0391~Immunity | 38 | 46.91 | 4.33E-25 | FCN1, TRBV4-2, IGHV3-23, IGLV3-1, IGHV4-39, IGLV2-11, LGALS3, IGLV3-9, FCGR3A, IGLV1-40, IGLV6-57, IGKC, IGLV2-14, IGLV3-21, IGKV3-15, IGLV2-18, SLAMF7, TRAV14DV4, MAP3K8, KLRC1, IGHA1, IGKV3-11, HLA-DQA2, HLA-DPA1, KLRG1, HLA-DRB5, TRDV2, IGKV1-5, IGLV4-69, TYROBP, HLA-DPB1, IGKV4-1, HLA-DRA, KLRD1, S100A9, S100A8, IGKV3-20, IGLV3-19 | 61 | 980 | 11523 | 7.32E+00 | 1.08E-23 | 1.08E-23 | 9.95E-24 |
| UP_SEQ_FEATURE | DOMAIN:Ig-like | 31 | 38.27 | 6.03E-25 | TRBV4-2, IGHV3-23, IGLV3-1, IGHV4-39, MYOM2, IGLV2-11, IGLV3-9, FCGR3A, IGLV1-40, IGLV6-57, IGKC, IGLV2-14, IGLV3-21, IGKV3-15, IGLV2-18, SLAMF7, TRAV14DV4, IGKV3-11, HLA-DQA2, HLA-DPA1, HLA-DRB5, TRDV2, FCRL3, IGKV1-5, IGLV4-69, VCAN, HLA-DPB1, IGKV4-1, HLA-DRA, IGKV3-20, IGLV3-19 | 78 | 673 | 20675 | 1.22E+01 | 2.45E-22 | 2.45E-22 | 2.42E-22 |
| INTERPRO | IPR007110:Ig-like_dom | 32 | 39.51 | 1.28E-24 | TRBV4-2, IGHV3-23, IGLV3-1, IGHV4-39, MYOM2, IGLV2-11, IGLV3-9, FCGR3A, IGLV1-40, IGLV6-57, IGKC, IGLV2-14, IGLV3-21, IGKV3-15, IGLV2-18, SLAMF7, TRAV14DV4, IGHA1, IGKV3-11, HLA-DQA2, HLA-DPA1, HLA-DRB5, TRDV2, FCRL3, IGKV1-5, IGLV4-69, VCAN, HLA-DPB1, IGKV4-1, HLA-DRA, IGKV3-20, IGLV3-19 | 78 | 768 | 20808 | 1.11E+01 | 3.07E-22 | 3.07E-22 | 2.89E-22 |
| UP_KW_BIOLOGICAL_PROCESS | KW-1064~Adaptive immunity | 30 | 37.04 | 1.39E-23 | TRBV4-2, IGHV3-23, IGLV3-1, IGHV4-39, IGLV2-11, IGLV3-9, IGLV1-40, IGLV6-57, IGKC, IGLV2-14, IGLV3-21, IGKV3-15, IGLV2-18, SLAMF7, TRAV14DV4, KLRC1, IGHA1, IGKV3-11, HLA-DQA2, HLA-DPA1, HLA-DRB5, TRDV2, IGKV1-5, IGLV4-69, HLA-DPB1, IGKV4-1, HLA-DRA, KLRD1, IGKV3-20, IGLV3-19 | 61 | 521 | 11523 | 1.09E+01 | 3.47E-22 | 1.73E-22 | 1.60E-22 |
| INTERPRO | IPR036179:Ig-like_dom_sf | 32 | 39.51 | 5.57E-23 | TRBV4-2, IGHV3-23, IGLV3-1, IGHV4-39, MYOM2, IGLV2-11, IGLV3-9, FCGR3A, IGLV1-40, IGLV6-57, IGKC, IGLV2-14, IGLV3-21, IGKV3-15, IGLV2-18, SLAMF7, TRAV14DV4, IGHA1, IGKV3-11, HLA-DQA2, HLA-DPA1, HLA-DRB5, TRDV2, FCRL3, IGKV1-5, IGLV4-69, VCAN, HLA-DPB1, IGKV4-1, HLA-DRA, IGKV3-20, IGLV3-19 | 78 | 872 | 20808 | 9.79E+00 | 1.33E-20 | 6.66E-21 | 6.27E-21 |
| GOTERM_MF_DIRECT | GO:0003823~antigen binding | 18 | 22.22 | 1.55E-21 | FCN1, IGKV1-5, IGHV3-23, IGLV3-1, IGHV4-39, JCHAIN, IGLV2-11, IGLV1-40, IGLV6-57, IGKC, IGLV2-14, IGLV3-21, IGKV4-1, IGKV3-15, IGHA1, IGKV3-11, IGKV3-20, IGLV3-19 | 73 | 140 | 19253 | 3.39E+01 | 3.19E-19 | 3.19E-19 | 3.14E-19 |
| UP_KW_PTM | KW-1015~Disulfide bond | 60 | 74.07 | 1.22E-20 | FCN1, TRBV4-2, IGHV3-23, PRF1, IGHV4-39, CTSS, JCHAIN, LGALS3, FCGR3A, GNLY, TRAV14DV4, HLA-DPA1, PTGDR, FCRL3, APLP2, F2R, TNFRSF1B, TGFBR3, ERN1, IGLV4-69, VCAN, TYROBP, CANX, IGKV4-1, IGLV3-19, GRN, IGLV3-1, AOAH, CST7, IGLV2-11, CST3, IGLV3-9, IGLV1-40, IGKC, IGLV6-57, IGLV2-14, IGLV3-21, PSAP, CCL4, IGKV3-15, IGLV2-18, SLAMF7, KLRC1, IGHA1, IGKV3-11, HLA-DQA2, KLRG1, HLA-DRB5, TRDV2, GZMA, IGKV1-5, PPBP, LYZ, GZMK, GOLGB1, HLA-DPB1, HLA-DRA, FAS, KLRD1, IGKV3-20 | 75 | 3956 | 14316 | 2.90E+00 | 1.84E-19 | 1.96E-19 | 1.96E-19 |
| GOTERM_CC_DIRECT | GO:0005576~extracellular region | 43 | 53.09 | 2.08E-20 | FCN1, GRN, IGHV3-23, PLEK, PRF1, IGLV3-1, IGHV4-39, AOAH, CTSS, JCHAIN, IGLV2-11, CST3, LGALS3, IGLV3-9, IGLV1-40, IGLV6-57, IGKC, IGLV2-14, GNLY, IGLV3-21, CCL4, PSAP, IGKV3-15, IGLV2-18, IGHA1, IGKV3-11, ANXA2, IGKV1-5, F2R, PPBP, TNFRSF1B, LYZ, TGFBR3, IGLV4-69, VCAN, IGKV4-1, MNDA, TLN1, S100A9, S100A8, IGKV3-20, VCL, IGLV3-19 | 79 | 2313 | 20795 | 4.89E+00 | 3.69E-18 | 3.69E-18 | 3.15E-18 |
| UP_SEQ_FEATURE | REGION:Complementarity-determining-3 | 17 | 20.99 | 6.78E-20 | IGKV1-5, IGHV3-23, IGLV3-1, IGHV4-39, IGLV2-11, IGLV4-69, IGLV3-9, IGLV1-40, IGLV6-57, IGLV2-14, IGLV3-21, IGKV4-1, IGKV3-15, IGLV2-18, IGKV3-11, IGKV3-20, IGLV3-19 | 78 | 139 | 20675 | 3.24E+01 | 2.75E-17 | 5.59E-18 | 5.53E-18 |
| UP_KW_CELLULAR_COMPONENT | KW-1280~Immunoglobulin | 19 | 23.46 | 7.41E-20 | IGKV1-5, IGHV3-23, IGLV3-1, IGHV4-39, IGLV2-11, IGLV4-69, IGLV3-9, IGLV1-40, IGLV6-57, IGKC, IGLV2-14, IGLV3-21, IGKV4-1, IGKV3-15, IGLV2-18, IGHA1, IGKV3-11, IGKV3-20, IGLV3-19 | 76 | 192 | 18049 | 2.35E+01 | 1.78E-18 | 1.39E-18 | 1.16E-18 |
| UP_SEQ_FEATURE | REGION:Framework-1 | 17 | 20.99 | 8.58E-20 | IGKV1-5, IGHV3-23, IGLV3-1, IGHV4-39, IGLV2-11, IGLV4-69, IGLV3-9, IGLV1-40, IGLV6-57, IGLV2-14, IGLV3-21, IGKV4-1, IGKV3-15, IGLV2-18, IGKV3-11, IGKV3-20, IGLV3-19 | 78 | 141 | 20675 | 3.20E+01 | 3.49E-17 | 5.59E-18 | 5.53E-18 |
| UP_SEQ_FEATURE | REGION:Complementarity-determining-1 | 17 | 20.99 | 9.65E-20 | IGKV1-5, IGHV3-23, IGLV3-1, IGHV4-39, IGLV2-11, IGLV4-69, IGLV3-9, IGLV1-40, IGLV6-57, IGLV2-14, IGLV3-21, IGKV4-1, IGKV3-15, IGLV2-18, IGKV3-11, IGKV3-20, IGLV3-19 | 78 | 142 | 20675 | 3.17E+01 | 3.92E-17 | 5.59E-18 | 5.53E-18 |
| UP_SEQ_FEATURE | REGION:Complementarity-determining-2 | 17 | 20.99 | 9.65E-20 | IGKV1-5, IGHV3-23, IGLV3-1, IGHV4-39, IGLV2-11, IGLV4-69, IGLV3-9, IGLV1-40, IGLV6-57, IGLV2-14, IGLV3-21, IGKV4-1, IGKV3-15, IGLV2-18, IGKV3-11, IGKV3-20, IGLV3-19 | 78 | 142 | 20675 | 3.17E+01 | 3.92E-17 | 5.59E-18 | 5.53E-18 |
| UP_SEQ_FEATURE | REGION:Framework-2 | 17 | 20.99 | 9.65E-20 | IGKV1-5, IGHV3-23, IGLV3-1, IGHV4-39, IGLV2-11, IGLV4-69, IGLV3-9, IGLV1-40, IGLV6-57, IGLV2-14, IGLV3-21, IGKV4-1, IGKV3-15, IGLV2-18, IGKV3-11, IGKV3-20, IGLV3-19 | 78 | 142 | 20675 | 3.17E+01 | 3.92E-17 | 5.59E-18 | 5.53E-18 |
| UP_SEQ_FEATURE | REGION:Framework-3 | 17 | 20.99 | 9.65E-20 | IGKV1-5, IGHV3-23, IGLV3-1, IGHV4-39, IGLV2-11, IGLV4-69, IGLV3-9, IGLV1-40, IGLV6-57, IGLV2-14, IGLV3-21, IGKV4-1, IGKV3-15, IGLV2-18, IGKV3-11, IGKV3-20, IGLV3-19 | 78 | 142 | 20675 | 3.17E+01 | 3.92E-17 | 5.59E-18 | 5.53E-18 |
| INTERPRO | IPR013783:Ig-like_fold | 32 | 39.51 | 9.91E-20 | TRBV4-2, IGHV3-23, IGLV3-1, IGHV4-39, MYOM2, IGLV2-11, IGLV3-9, FCGR3A, IGLV1-40, IGLV6-57, IGKC, IGLV2-14, IGLV3-21, IGKV3-15, IGLV2-18, SLAMF7, TRAV14DV4, IGHA1, IGKV3-11, HLA-DQA2, HLA-DPA1, HLA-DRB5, TRDV2, FCRL3, IGKV1-5, IGLV4-69, VCAN, HLA-DPB1, IGKV4-1, HLA-DRA, IGKV3-20, IGLV3-19 | 78 | 1127 | 20808 | 7.57E+00 | 2.37E-17 | 7.89E-18 | 7.43E-18 |
| UP_KW_CELLULAR_COMPONENT | KW-0964~Secreted | 43 | 53.09 | 1.16E-19 | FCN1, GRN, IGHV3-23, PRF1, IGLV3-1, IGHV4-39, AOAH, CST7, CTSS, JCHAIN, IGLV2-11, CST3, LGALS3, IGLV3-9, FCGR3A, IGLV1-40, IGLV6-57, IGKC, IGLV2-14, GNLY, IGLV3-21, CCL4, PSAP, IGKV3-15, IGLV2-18, IGHA1, IGKV3-11, ANXA2, GZMA, IGKV1-5, PPBP, TNFRSF1B, LYZ, TGFBR3, IGLV4-69, GZMK, VCAN, IGKV4-1, FAS, S100A9, S100A8, IGKV3-20, IGLV3-19 | 76 | 2217 | 18049 | 4.61E+00 | 2.78E-18 | 1.39E-18 | 1.16E-18 |
| INTERPRO | IPR050150:IgV_Light_Chain | 15 | 18.52 | 3.01E-19 | IGKV1-5, IGLV3-1, IGLV2-11, IGLV4-69, IGLV3-9, IGLV1-40, IGLV6-57, IGLV2-14, IGLV3-21, IGKV4-1, IGKV3-15, IGLV2-18, IGKV3-11, IGKV3-20, IGLV3-19 | 78 | 93 | 20808 | 4.30E+01 | 7.19E-17 | 1.80E-17 | 1.69E-17 |
| GOTERM_CC_DIRECT | GO:0019814~immunoglobulin complex | 18 | 22.22 | 5.59E-19 | IGKV1-5, IGHV3-23, IGLV3-1, IGHV4-39, IGLV2-11, IGLV4-69, IGLV3-9, IGLV1-40, IGLV6-57, IGLV2-14, IGLV3-21, IGKV4-1, IGKV3-15, IGLV2-18, TRAV14DV4, IGKV3-11, IGKV3-20, IGLV3-19 | 79 | 194 | 20795 | 2.44E+01 | 9.89E-17 | 4.94E-17 | 4.22E-17 |
| INTERPRO | IPR013106:Ig_V-set | 22 | 27.16 | 3.83E-17 | TRDV2, TRBV4-2, IGKV1-5, IGHV3-23, IGLV3-1, IGHV4-39, IGLV2-11, IGLV4-69, IGLV3-9, VCAN, IGLV1-40, IGLV6-57, IGLV2-14, IGLV3-21, IGKV4-1, IGKV3-15, IGLV2-18, TRAV14DV4, SLAMF7, IGKV3-11, IGKV3-20, IGLV3-19 | 78 | 485 | 20808 | 1.21E+01 | 9.16E-15 | 1.83E-15 | 1.72E-15 |
| UP_KW_DOMAIN | KW-0393~Immunoglobulin domain | 27 | 33.33 | 7.91E-15 | TRBV4-2, IGHV3-23, IGLV3-1, IGHV4-39, MYOM2, IGLV2-11, IGLV3-9, FCGR3A, IGLV1-40, IGLV6-57, IGKC, IGLV2-14, IGLV3-21, IGKV3-15, IGLV2-18, SLAMF7, TRAV14DV4, IGHA1, IGKV3-11, TRDV2, FCRL3, IGKV1-5, IGLV4-69, VCAN, IGKV4-1, IGKV3-20, IGLV3-19 | 74 | 825 | 14625 | 6.47E+00 | 1.18E-13 | 1.19E-13 | 1.19E-13 |
| UP_KW_DOMAIN | KW-0732~Signal | 54 | 66.67 | 8.93E-14 | FCN1, TRBV4-2, IGHV3-23, PRF1, IGHV4-39, CTSS, JCHAIN, FCGR3A, GNLY, TRAV14DV4, HLA-DPA1, FCRL3, APLP2, F2R, TNFRSF1B, TGFBR3, ERN1, IGLV4-69, VCAN, TYROBP, CANX, IGKV4-1, IGLV3-19, GRN, IGLV3-1, AOAH, CST7, IGLV2-11, CST3, IGLV3-9, IGLV1-40, IGLV6-57, IGLV2-14, IGLV3-21, PSAP, CCL4, IGKV3-15, IGLV2-18, SLAMF7, IGKV3-11, HLA-DQA2, HLA-DRB5, TRDV2, HSPA5, GZMA, IGKV1-5, PPBP, LYZ, GZMK, GOLGB1, HLA-DPB1, HLA-DRA, FAS, IGKV3-20 | 74 | 4415 | 14625 | 2.42E+00 | 1.34E-12 | 6.70E-13 | 6.70E-13 |
| SMART | SM00406:IGv | 20 | 24.69 | 1.23E-13 | TRDV2, TRBV4-2, IGKV1-5, IGHV3-23, IGLV3-1, IGHV4-39, IGLV2-11, IGLV4-69, IGLV3-9, IGLV1-40, IGLV6-57, IGLV2-14, IGLV3-21, IGKV4-1, IGKV3-15, IGLV2-18, TRAV14DV4, IGKV3-11, IGKV3-20, IGLV3-19 | 69 | 334 | 10706 | 9.29E+00 | 6.26E-12 | 6.26E-12 | 6.14E-12 |
| GOTERM_CC_DIRECT | GO:0005886~plasma membrane | 53 | 65.43 | 2.49E-13 | FCN1, TRBV4-2, IGHV3-23, PLEK, PRF1, IGHV4-39, LGALS3, FCGR3A, HLA-DPA1, PTGDR, ANXA2, APLP2, F2R, TNFRSF1B, TGFBR3, IGLV4-69, ZEB2, TYROBP, IGKV4-1, TLN1, S100A9, S100A8, VCL, IGLV3-19, GRN, AHNAK, IGLV3-1, IGLV2-11, CST3, IGLV3-9, IGLV1-40, IGKC, IGLV6-57, IGLV2-14, IGLV3-21, PSAP, IGKV3-15, IGLV2-18, SLAMF7, KLRC1, IGHA1, IGKV3-11, HLA-DQA2, KLRG1, HLA-DRB5, HSPA5, IGKV1-5, HLA-DPB1, HLA-DRA, FAS, KLRD1, MYO1F, IGKV3-20 | 79 | 5597 | 20795 | 2.49E+00 | 4.41E-11 | 1.47E-11 | 1.25E-11 |
| INTERPRO | IPR003599:Ig_sub | 19 | 23.46 | 6.43E-13 | FCRL3, IGKV1-5, IGLV3-1, MYOM2, IGLV2-11, IGLV4-69, IGLV3-9, FCGR3A, VCAN, IGLV1-40, IGLV6-57, IGLV2-14, IGLV3-21, IGKV4-1, IGKV3-15, IGLV2-18, IGKV3-11, IGKV3-20, IGLV3-19 | 78 | 531 | 20808 | 9.55E+00 | 1.54E-10 | 2.56E-11 | 2.41E-11 |
| KEGG_PATHWAY | hsa05332:Graft-versus-host disease | 9 | 11.11 | 6.57E-12 | HLA-DRB5, HLA-DPB1, PRF1, HLA-DRA, FAS, KLRD1, KLRC1, HLA-DQA2, HLA-DPA1 | 36 | 45 | 8534 | 4.74E+01 | 6.05E-10 | 6.05E-10 | 4.53E-10 |
| KEGG_PATHWAY | hsa04612:Antigen processing and presentation | 10 | 12.35 | 2.29E-11 | HLA-DRB5, HSPA5, CANX, HLA-DPB1, HLA-DRA, KLRD1, KLRC1, HLA-DQA2, CTSS, HLA-DPA1 | 36 | 81 | 8534 | 2.93E+01 | 2.11E-09 | 1.05E-09 | 7.91E-10 |
| UP_KW_CELLULAR_COMPONENT | KW-1003~Cell membrane | 45 | 55.56 | 2.75E-11 | FCN1, TRBV4-2, IGHV3-23, PRF1, IGLV3-1, IGHV4-39, IGLV2-11, IGLV3-9, FCGR3A, IGLV1-40, IGLV6-57, IGKC, IGLV2-14, IGLV3-21, IGKV3-15, IGLV2-18, TRAV14DV4, KLRC1, IGHA1, IGKV3-11, HLA-DQA2, PTGDR, HLA-DPA1, KLRG1, HLA-DRB5, TRDV2, APLP2, FCRL3, IGKV1-5, F2R, TNFRSF1B, TGFBR3, IGLV4-69, TYROBP, HLA-DPB1, IGKV4-1, FAS, HLA-DRA, KLRD1, TLN1, S100A9, S100A8, IGKV3-20, VCL, IGLV3-19 | 76 | 4134 | 18049 | 2.59E+00 | 6.59E-10 | 2.20E-10 | 1.83E-10 |
| GOTERM_CC_DIRECT | GO:0072562~blood microparticle | 11 | 13.58 | 2.04E-10 | IGKC, IGLV3-21, ZBTB38, IGHV3-23, IGKV1-5, IGKV3-15, IGKV4-1, IGKV3-11, IGHA1, IGKV3-20, JCHAIN | 79 | 148 | 20795 | 1.96E+01 | 3.61E-08 | 9.04E-09 | 7.71E-09 |
| GOTERM_CC_DIRECT | GO:0070062~extracellular exosome | 30 | 37.04 | 7.81E-10 | GRN, AHNAK, IGHV3-23, JCHAIN, CST3, LGALS3, FCGR3A, IGKC, IGLV2-14, IGLV3-21, PSAP, IGHA1, IGKV3-11, HLA-DRB5, HSPA5, ANXA2, APLP2, IGKV1-5, LYZ, TGFBR3, CANX, FAS, HLA-DRA, MNDA, TLN1, S100A9, S100A8, IGKV3-20, VCL, IGLV3-19 | 79 | 2242 | 20795 | 3.52E+00 | 1.38E-07 | 2.76E-08 | 2.36E-08 |
| SMART | SM00409:IG | 19 | 23.46 | 3.12E-09 | FCRL3, IGKV1-5, IGLV3-1, MYOM2, IGLV2-11, IGLV4-69, IGLV3-9, FCGR3A, VCAN, IGLV1-40, IGLV6-57, IGLV2-14, IGLV3-21, IGKV4-1, IGKV3-15, IGLV2-18, IGKV3-11, IGKV3-20, IGLV3-19 | 69 | 531 | 10706 | 5.55E+00 | 1.59E-07 | 7.95E-08 | 7.80E-08 |
| KEGG_PATHWAY | hsa05330:Allograft rejection | 7 | 8.64 | 8.98E-09 | HLA-DRB5, HLA-DPB1, PRF1, HLA-DRA, FAS, HLA-DQA2, HLA-DPA1 | 36 | 39 | 8534 | 4.25E+01 | 8.26E-07 | 2.75E-07 | 2.07E-07 |
| KEGG_PATHWAY | hsa04940:Type I diabetes mellitus | 7 | 8.64 | 1.91E-08 | HLA-DRB5, HLA-DPB1, PRF1, HLA-DRA, FAS, HLA-DQA2, HLA-DPA1 | 36 | 44 | 8534 | 3.77E+01 | 1.76E-06 | 4.40E-07 | 3.30E-07 |
| KEGG_PATHWAY | hsa05320:Autoimmune thyroid disease | 7 | 8.64 | 6.80E-08 | HLA-DRB5, HLA-DPB1, PRF1, HLA-DRA, FAS, HLA-DQA2, HLA-DPA1 | 36 | 54 | 8534 | 3.07E+01 | 6.26E-06 | 1.25E-06 | 9.39E-07 |
| GOTERM_BP_DIRECT | GO:0019886~antigen processing and presentation of exogenous peptide antigen via MHC class II | 6 | 7.41 | 1.42E-07 | HLA-DRB5, HLA-DPB1, HLA-DRA, HLA-DQA2, CTSS, HLA-DPA1 | 79 | 31 | 19478 | 4.77E+01 | 7.43E-05 | 2.48E-05 | 2.42E-05 |
| GOTERM_CC_DIRECT | GO:0098553~lumenal side of endoplasmic reticulum membrane | 6 | 7.41 | 1.94E-07 | HLA-DRB5, CANX, HLA-DPB1, HLA-DRA, HLA-DQA2, HLA-DPA1 | 79 | 35 | 20795 | 4.51E+01 | 3.43E-05 | 4.95E-06 | 4.22E-06 |
| GOTERM_CC_DIRECT | GO:0005615~extracellular space | 24 | 29.63 | 1.96E-07 | FCN1, GRN, ANXA2, GZMA, IGHV3-23, PPBP, LYZ, CST7, CTSS, JCHAIN, TGFBR3, GZMK, CST3, LGALS3, FCGR3A, VCAN, IGKC, GNLY, CCL4, PSAP, IGHA1, S100A9, S100A8, IGKV3-20 | 79 | 1867 | 20795 | 3.38E+00 | 3.47E-05 | 4.95E-06 | 4.22E-06 |
| INTERPRO | IPR003006:Ig/MHC_CS | 7 | 8.64 | 2.10E-07 | HLA-DRB5, IGKC, HLA-DPB1, HLA-DRA, IGHA1, HLA-DQA2, HLA-DPA1 | 78 | 69 | 20808 | 2.71E+01 | 5.01E-05 | 7.16E-06 | 6.74E-06 |
| GOTERM_BP_DIRECT | GO:0002503~peptide antigen assembly with MHC class II protein complex | 5 | 6.17 | 4.17E-07 | HLA-DRB5, HLA-DPB1, HLA-DRA, HLA-DQA2, HLA-DPA1 | 79 | 16 | 19478 | 7.70E+01 | 2.19E-04 | 5.48E-05 | 5.35E-05 |
| INTERPRO | IPR003597:Ig_C1-set | 7 | 8.64 | 4.38E-07 | HLA-DRB5, IGKC, HLA-DPB1, HLA-DRA, IGHA1, HLA-DQA2, HLA-DPA1 | 78 | 78 | 20808 | 2.39E+01 | 1.05E-04 | 1.31E-05 | 1.23E-05 |
| GOTERM_BP_DIRECT | GO:0019882~antigen processing and presentation | 6 | 7.41 | 9.75E-07 | HLA-DRB5, HLA-DPB1, HLA-DRA, HLA-DQA2, CTSS, HLA-DPA1 | 79 | 45 | 19478 | 3.29E+01 | 5.12E-04 | 9.61E-05 | 9.39E-05 |
| GOTERM_BP_DIRECT | GO:0002504~antigen processing and presentation of peptide or polysaccharide antigen via MHC class II | 5 | 6.17 | 1.10E-06 | HLA-DRB5, HLA-DPB1, HLA-DRA, HLA-DQA2, HLA-DPA1 | 79 | 20 | 19478 | 6.16E+01 | 5.76E-04 | 9.61E-05 | 9.39E-05 |
| UP_KW_CELLULAR_COMPONENT | KW-0491~MHC II | 5 | 6.17 | 1.27E-06 | HLA-DRB5, HLA-DPB1, HLA-DRA, HLA-DQA2, HLA-DPA1 | 76 | 20 | 18049 | 5.94E+01 | 3.04E-05 | 7.60E-06 | 6.33E-06 |
| INTERPRO | IPR001003:MHC_II_a_N | 4 | 4.94 | 1.69E-06 | HLA-DPB1, HLA-DRA, HLA-DQA2, HLA-DPA1 | 78 | 7 | 20808 | 1.52E+02 | 4.03E-04 | 4.16E-05 | 3.92E-05 |
| INTERPRO | IPR014745:MHC_II_a/b_N | 5 | 6.17 | 1.74E-06 | HLA-DRB5, HLA-DPB1, HLA-DRA, HLA-DQA2, HLA-DPA1 | 78 | 24 | 20808 | 5.56E+01 | 4.16E-04 | 4.16E-05 | 3.92E-05 |
| GOTERM_CC_DIRECT | GO:0042613~MHC class II protein complex | 5 | 6.17 | 2.57E-06 | HLA-DRB5, HLA-DPB1, HLA-DRA, HLA-DQA2, HLA-DPA1 | 79 | 26 | 20795 | 5.06E+01 | 4.55E-04 | 5.69E-05 | 4.85E-05 |
| GOTERM_MF_DIRECT | GO:0023026~MHC class II protein complex binding | 5 | 6.17 | 2.96E-06 | HLA-DRB5, HLA-DPB1, HLA-DRA, HLA-DQA2, HLA-DPA1 | 73 | 27 | 19253 | 4.88E+01 | 6.09E-04 | 3.04E-04 | 3.00E-04 |
| KEGG_PATHWAY | hsa04145:Phagosome | 8 | 9.88 | 2.96E-06 | FCGR3A, HLA-DRB5, CANX, HLA-DPB1, HLA-DRA, HLA-DQA2, CTSS, HLA-DPA1 | 36 | 159 | 8534 | 1.19E+01 | 2.72E-04 | 4.54E-05 | 3.41E-05 |
| INTERPRO | IPR050160:MHC/Immunoglobulin | 5 | 6.17 | 4.41E-06 | HLA-DRB5, HLA-DPB1, HLA-DRA, HLA-DQA2, HLA-DPA1 | 78 | 30 | 20808 | 4.45E+01 | 1.05E-03 | 9.59E-05 | 9.03E-05 |
| SMART | SM00407:IGc1 | 7 | 8.64 | 7.49E-06 | HLA-DRB5, IGKC, HLA-DPB1, HLA-DRA, IGHA1, HLA-DQA2, HLA-DPA1 | 69 | 75 | 10706 | 1.45E+01 | 3.82E-04 | 1.07E-04 | 1.05E-04 |
| KEGG_PATHWAY | hsa05310:Asthma | 5 | 6.17 | 7.86E-06 | HLA-DRB5, HLA-DPB1, HLA-DRA, HLA-DQA2, HLA-DPA1 | 36 | 32 | 8534 | 3.70E+01 | 7.23E-04 | 9.91E-05 | 7.43E-05 |
| SMART | SM00920:MHC_II_alpha | 4 | 4.94 | 8.42E-06 | HLA-DPB1, HLA-DRA, HLA-DQA2, HLA-DPA1 | 69 | 7 | 10706 | 8.87E+01 | 4.30E-04 | 1.07E-04 | 1.05E-04 |
| KEGG_PATHWAY | hsa05416:Viral myocarditis | 6 | 7.41 | 8.62E-06 | HLA-DRB5, HLA-DPB1, PRF1, HLA-DRA, HLA-DQA2, HLA-DPA1 | 36 | 70 | 8534 | 2.03E+01 | 7.92E-04 | 9.91E-05 | 7.43E-05 |
| UP_KW_LIGAND | KW-0430~Lectin | 7 | 8.64 | 1.09E-05 | FCN1, LGALS3, VCAN, CANX, KLRD1, KLRC1, KLRG1 | 22 | 181 | 6987 | 1.23E+01 | 1.20E-04 | 1.20E-04 | 1.20E-04 |
| GOTERM_MF_DIRECT | GO:0032395~MHC class II receptor activity | 4 | 4.94 | 1.40E-05 | HLA-DPB1, HLA-DRA, HLA-DQA2, HLA-DPA1 | 73 | 13 | 19253 | 8.12E+01 | 2.87E-03 | 9.59E-04 | 9.45E-04 |
| GOTERM_BP_DIRECT | GO:0050778~positive regulation of immune response | 5 | 6.17 | 1.42E-05 | HLA-DRB5, HLA-DPB1, HLA-DRA, HLA-DQA2, HLA-DPA1 | 79 | 37 | 19478 | 3.33E+01 | 7.43E-03 | 1.04E-03 | 1.02E-03 |
| UP_SEQ_FEATURE | DOMAIN:Ig-like C1-type | 5 | 6.17 | 1.47E-05 | HLA-DRB5, HLA-DPB1, HLA-DRA, HLA-DQA2, HLA-DPA1 | 78 | 40 | 20675 | 3.31E+01 | 5.94E-03 | 7.45E-04 | 7.36E-04 |
| KEGG_PATHWAY | hsa05140:Leishmaniasis | 6 | 7.41 | 1.56E-05 | FCGR3A, HLA-DRB5, HLA-DPB1, HLA-DRA, HLA-DQA2, HLA-DPA1 | 36 | 79 | 8534 | 1.80E+01 | 1.44E-03 | 1.60E-04 | 1.20E-04 |
| GOTERM_BP_DIRECT | GO:0050870~positive regulation of T cell activation | 5 | 6.17 | 1.58E-05 | HLA-DRB5, HLA-DPB1, HLA-DRA, HLA-DQA2, HLA-DPA1 | 79 | 38 | 19478 | 3.24E+01 | 8.28E-03 | 1.04E-03 | 1.02E-03 |
| INTERPRO | IPR011162:MHC_I/II-like_Ag-recog | 5 | 6.17 | 2.74E-05 | HLA-DRB5, HLA-DPB1, HLA-DRA, HLA-DQA2, HLA-DPA1 | 78 | 47 | 20808 | 2.84E+01 | 6.53E-03 | 5.46E-04 | 5.14E-04 |
| GOTERM_MF_DIRECT | GO:0042605~peptide antigen binding | 5 | 6.17 | 3.09E-05 | HLA-DRB5, HLA-DPB1, HLA-DRA, HLA-DQA2, HLA-DPA1 | 73 | 48 | 19253 | 2.75E+01 | 6.34E-03 | 1.59E-03 | 1.57E-03 |
| GOTERM_CC_DIRECT | GO:0030658~transport vesicle membrane | 5 | 6.17 | 3.41E-05 | HLA-DRB5, HLA-DPB1, HLA-DRA, HLA-DQA2, HLA-DPA1 | 79 | 49 | 20795 | 2.69E+01 | 6.02E-03 | 6.21E-04 | 5.30E-04 |
| GOTERM_CC_DIRECT | GO:0009986~cell surface | 12 | 14.81 | 3.51E-05 | TGFBR3, LGALS3, TYROBP, HSPA5, ANXA2, FCRL3, F2R, HLA-DPB1, HLA-DRA, FAS, TLN1, HLA-DPA1 | 79 | 662 | 20795 | 4.77E+00 | 6.19E-03 | 6.21E-04 | 5.30E-04 |
| GOTERM_BP_DIRECT | GO:0031640~killing of cells of another organism | 6 | 7.41 | 3.57E-05 | LGALS3, GNLY, GZMA, PRF1, PPBP, LYZ | 79 | 93 | 19478 | 1.59E+01 | 1.86E-02 | 2.08E-03 | 2.03E-03 |
| UP_SEQ_FEATURE | DOMAIN:Immunoglobulin C1-set | 4 | 4.94 | 4.61E-05 | HLA-DRB5, HLA-DPB1, HLA-DRA, HLA-DPA1 | 78 | 19 | 20675 | 5.58E+01 | 1.85E-02 | 2.08E-03 | 2.05E-03 |
| KEGG_PATHWAY | hsa04672:Intestinal immune network for IgA production | 5 | 6.17 | 4.78E-05 | HLA-DRB5, HLA-DPB1, HLA-DRA, HLA-DQA2, HLA-DPA1 | 36 | 50 | 8534 | 2.37E+01 | 4.38E-03 | 4.39E-04 | 3.30E-04 |
| KEGG_PATHWAY | hsa05150:Staphylococcus aureus infection | 6 | 7.41 | 5.40E-05 | FCGR3A, HLA-DRB5, HLA-DPB1, HLA-DRA, HLA-DQA2, HLA-DPA1 | 36 | 102 | 8534 | 1.39E+01 | 4.95E-03 | 4.51E-04 | 3.39E-04 |
| GOTERM_CC_DIRECT | GO:0012507~ER to Golgi transport vesicle membrane | 5 | 6.17 | 6.66E-05 | HLA-DRB5, HLA-DPB1, HLA-DRA, HLA-DQA2, HLA-DPA1 | 79 | 58 | 20795 | 2.27E+01 | 1.17E-02 | 1.07E-03 | 9.15E-04 |
| GOTERM_BP_DIRECT | GO:0006968~cellular defense response | 5 | 6.17 | 8.00E-05 | TYROBP, GNLY, PRF1, MNDA, KLRG1 | 79 | 57 | 19478 | 2.16E+01 | 4.11E-02 | 4.20E-03 | 4.10E-03 |
| GOTERM_CC_DIRECT | GO:0071748~monomeric IgA immunoglobulin complex | 3 | 3.70 | 8.29E-05 | IGHA1, IGKV3-20, JCHAIN | 79 | 4 | 20795 | 1.97E+02 | 1.46E-02 | 1.22E-03 | 1.04E-03 |
| KEGG_PATHWAY | hsa05152:Tuberculosis | 7 | 8.64 | 8.41E-05 | FCGR3A, HLA-DRB5, HLA-DPB1, HLA-DRA, HLA-DQA2, CTSS, HLA-DPA1 | 36 | 182 | 8534 | 9.12E+00 | 7.70E-03 | 6.44E-04 | 4.83E-04 |
| KEGG_PATHWAY | hsa04650:Natural killer cell mediated cytotoxicity | 6 | 7.41 | 1.04E-04 | FCGR3A, TYROBP, PRF1, FAS, KLRD1, KLRC1 | 36 | 117 | 8534 | 1.22E+01 | 9.51E-03 | 7.35E-04 | 5.51E-04 |
| GOTERM_CC_DIRECT | GO:0005765~lysosomal membrane | 9 | 11.11 | 1.29E-04 | GRN, HLA-DRB5, AHNAK, ANXA2, PSAP, HLA-DPB1, HLA-DRA, HLA-DQA2, HLA-DPA1 | 79 | 401 | 20795 | 5.91E+00 | 2.25E-02 | 1.74E-03 | 1.48E-03 |
| UP_SEQ_FEATURE | DOMAIN:MHC class II alpha chain N-terminal | 3 | 3.70 | 1.36E-04 | HLA-DRA, HLA-DQA2, HLA-DPA1 | 78 | 5 | 20675 | 1.59E+02 | 5.37E-02 | 5.52E-03 | 5.45E-03 |
| GOTERM_CC_DIRECT | GO:0071751~secretory IgA immunoglobulin complex | 3 | 3.70 | 1.38E-04 | IGHA1, IGKV3-20, JCHAIN | 79 | 5 | 20795 | 1.58E+02 | 2.41E-02 | 1.74E-03 | 1.48E-03 |
| KEGG_PATHWAY | hsa05321:Inflammatory bowel disease | 5 | 6.17 | 1.43E-04 | HLA-DRB5, HLA-DPB1, HLA-DRA, HLA-DQA2, HLA-DPA1 | 36 | 66 | 8534 | 1.80E+01 | 1.30E-02 | 9.38E-04 | 7.03E-04 |
| GOTERM_CC_DIRECT | GO:0030669~clathrin-coated endocytic vesicle membrane | 5 | 6.17 | 1.47E-04 | HLA-DRB5, HLA-DPB1, HLA-DRA, HLA-DQA2, HLA-DPA1 | 79 | 71 | 20795 | 1.85E+01 | 2.57E-02 | 1.74E-03 | 1.48E-03 |
| GOTERM_MF_DIRECT | GO:0030246~carbohydrate binding | 7 | 8.64 | 1.49E-04 | FCN1, LGALS3, VCAN, CANX, KLRD1, KLRC1, KLRG1 | 73 | 214 | 19253 | 8.63E+00 | 3.03E-02 | 6.14E-03 | 6.05E-03 |
| GOTERM_CC_DIRECT | GO:0030666~endocytic vesicle membrane | 5 | 6.17 | 1.91E-04 | HLA-DRB5, HLA-DPB1, HLA-DRA, HLA-DQA2, HLA-DPA1 | 79 | 76 | 20795 | 1.73E+01 | 3.33E-02 | 2.12E-03 | 1.81E-03 |
| INTERPRO | IPR011001:Saposin-like | 3 | 3.70 | 2.01E-04 | GNLY, PSAP, AOAH | 78 | 6 | 20808 | 1.33E+02 | 4.69E-02 | 3.69E-03 | 3.48E-03 |
| KEGG_PATHWAY | hsa05322:Systemic lupus erythematosus | 6 | 7.41 | 2.50E-04 | FCGR3A, HLA-DRB5, HLA-DPB1, HLA-DRA, HLA-DQA2, HLA-DPA1 | 36 | 141 | 8534 | 1.01E+01 | 2.27E-02 | 1.51E-03 | 1.13E-03 |
| KEGG_PATHWAY | hsa05166:Human T-cell leukemia virus 1 infection | 7 | 8.64 | 2.62E-04 | HLA-DRB5, CANX, HLA-DPB1, HLA-DRA, TLN1, HLA-DQA2, HLA-DPA1 | 36 | 224 | 8534 | 7.41E+00 | 2.38E-02 | 1.51E-03 | 1.13E-03 |
| INTERPRO | IPR008139:SaposinB_dom | 3 | 3.70 | 2.80E-04 | GNLY, PSAP, AOAH | 78 | 7 | 20808 | 1.14E+02 | 6.48E-02 | 4.79E-03 | 4.51E-03 |
| UP_SEQ_FEATURE | DOMAIN:Saposin B-type | 3 | 3.70 | 2.84E-04 | GNLY, PSAP, AOAH | 78 | 7 | 20675 | 1.14E+02 | 1.09E-01 | 1.05E-02 | 1.04E-02 |
| GOTERM_CC_DIRECT | GO:0031902~late endosome membrane | 6 | 7.41 | 3.12E-04 | HLA-DRB5, ANXA2, HLA-DPB1, HLA-DRA, HLA-DQA2, HLA-DPA1 | 79 | 157 | 20795 | 1.01E+01 | 5.37E-02 | 3.25E-03 | 2.77E-03 |
| GOTERM_BP_DIRECT | GO:0006954~inflammatory response | 9 | 11.11 | 3.31E-04 | F2R, CCL4, PPBP, LYZ, TNFRSF1B, S100A9, S100A8, PTGDR, KLRG1 | 79 | 432 | 19478 | 5.14E+00 | 1.60E-01 | 1.58E-02 | 1.55E-02 |
| UP_SEQ_FEATURE | REGION:Connecting peptide | 4 | 4.94 | 3.81E-04 | HLA-DPB1, HLA-DRA, HLA-DQA2, HLA-DPA1 | 78 | 38 | 20675 | 2.79E+01 | 1.44E-01 | 1.29E-02 | 1.27E-02 |
| KEGG_PATHWAY | hsa04514:Cell adhesion molecules | 6 | 7.41 | 4.23E-04 | VCAN, HLA-DRB5, HLA-DPB1, HLA-DRA, HLA-DQA2, HLA-DPA1 | 36 | 158 | 8534 | 9.00E+00 | 3.82E-02 | 2.29E-03 | 1.72E-03 |
| UP_KW_CELLULAR_COMPONENT | KW-0458~Lysosome | 9 | 11.11 | 4.46E-04 | GRN, HLA-DRB5, PSAP, HLA-DPB1, PRF1, HLA-DRA, HLA-DQA2, CTSS, HLA-DPA1 | 76 | 436 | 18049 | 4.90E+00 | 1.06E-02 | 2.14E-03 | 1.78E-03 |
| KEGG_PATHWAY | hsa04658:Th1 and Th2 cell differentiation | 5 | 6.17 | 5.34E-04 | HLA-DRB5, HLA-DPB1, HLA-DRA, HLA-DQA2, HLA-DPA1 | 36 | 93 | 8534 | 1.27E+01 | 4.80E-02 | 2.73E-03 | 2.05E-03 |
| KEGG_PATHWAY | hsa05323:Rheumatoid arthritis | 5 | 6.17 | 5.79E-04 | HLA-DRB5, HLA-DPB1, HLA-DRA, HLA-DQA2, HLA-DPA1 | 36 | 95 | 8534 | 1.25E+01 | 5.19E-02 | 2.80E-03 | 2.10E-03 |
| SMART | SM00741:SapB | 3 | 3.70 | 5.87E-04 | GNLY, PSAP, AOAH | 69 | 6 | 10706 | 7.76E+01 | 2.95E-02 | 5.98E-03 | 5.87E-03 |
| GOTERM_CC_DIRECT | GO:0062023~collagen-containing extracellular matrix | 8 | 9.88 | 6.22E-04 | FCN1, LGALS3, VCAN, ANXA2, PSAP, S100A9, CTSS, S100A8 | 79 | 387 | 20795 | 5.44E+00 | 1.04E-01 | 6.12E-03 | 5.22E-03 |
| KEGG_PATHWAY | hsa05164:Influenza A | 6 | 7.41 | 6.41E-04 | HLA-DRB5, HLA-DPB1, HLA-DRA, FAS, HLA-DQA2, HLA-DPA1 | 36 | 173 | 8534 | 8.22E+00 | 5.73E-02 | 2.95E-03 | 2.21E-03 |
| UP_KW_CELLULAR_COMPONENT | KW-0472~Membrane | 50 | 61.73 | 6.71E-04 | FCN1, TRBV4-2, IGHV3-23, PRF1, IGHV4-39, FCGR3A, TRAV14DV4, HLA-DPA1, PTGDR, FCRL3, APLP2, F2R, TNFRSF1B, TGFBR3, ERN1, IGLV4-69, TYROBP, CANX, IGKV4-1, TLN1, S100A9, S100A8, VCL, IGLV3-19, IGLV3-1, IGLV2-11, IGLV3-9, IGLV1-40, RAB11FIP1, IGKC, IGLV6-57, IGLV2-14, IGLV3-21, IGKV3-15, IGLV2-18, SLAMF7, KLRC1, IGHA1, IGKV3-11, HLA-DQA2, KLRG1, HLA-DRB5, TRDV2, IGKV1-5, GOLGB1, HLA-DPB1, HLA-DRA, FAS, KLRD1, IGKV3-20 | 76 | 8353 | 18049 | 1.42E+00 | 1.60E-02 | 2.68E-03 | 2.24E-03 |
| GOTERM_BP_DIRECT | GO:0140507~granzyme-mediated programmed cell death signaling pathway | 3 | 3.70 | 6.98E-04 | GZMK, GZMA, PRF1 | 79 | 10 | 19478 | 7.40E+01 | 3.07E-01 | 3.05E-02 | 2.98E-02 |
| KEGG_PATHWAY | hsa04640:Hematopoietic cell lineage | 5 | 6.17 | 7.03E-04 | HLA-DRB5, HLA-DPB1, HLA-DRA, HLA-DQA2, HLA-DPA1 | 36 | 100 | 8534 | 1.19E+01 | 6.27E-02 | 3.08E-03 | 2.31E-03 |
| UP_KW_MOLECULAR_FUNCTION | KW-0929~Antimicrobial | 5 | 6.17 | 7.09E-04 | GNLY, PPBP, LYZ, S100A9, S100A8 | 43 | 116 | 11952 | 1.20E+01 | 2.52E-02 | 2.55E-02 | 2.55E-02 |
| GOTERM_CC_DIRECT | GO:0001772~immunological synapse | 4 | 4.94 | 7.78E-04 | LGALS3, GZMA, PRF1, HLA-DRA | 79 | 48 | 20795 | 2.19E+01 | 1.29E-01 | 7.24E-03 | 6.18E-03 |
| KEGG_PATHWAY | hsa05168:Herpes simplex virus 1 infection | 6 | 7.41 | 8.06E-04 | HLA-DRB5, HLA-DPB1, HLA-DRA, FAS, HLA-DQA2, HLA-DPA1 | 36 | 182 | 8534 | 7.82E+00 | 7.15E-02 | 3.37E-03 | 2.53E-03 |
| GOTERM_CC_DIRECT | GO:0032588~trans-Golgi network membrane | 5 | 6.17 | 8.37E-04 | HLA-DRB5, HLA-DPB1, HLA-DRA, HLA-DQA2, HLA-DPA1 | 79 | 112 | 20795 | 1.18E+01 | 1.38E-01 | 7.41E-03 | 6.32E-03 |
| GOTERM_CC_DIRECT | GO:0009897~external side of plasma membrane | 8 | 9.88 | 9.11E-04 | TGFBR3, FCN1, FCGR3A, FCRL3, FAS, SLAMF7, KLRD1, KLRC1 | 79 | 413 | 20795 | 5.10E+00 | 1.49E-01 | 7.68E-03 | 6.55E-03 |
| KEGG_PATHWAY | hsa04659:Th17 cell differentiation | 5 | 6.17 | 9.72E-04 | HLA-DRB5, HLA-DPB1, HLA-DRA, HLA-DQA2, HLA-DPA1 | 36 | 109 | 8534 | 1.09E+01 | 8.56E-02 | 3.89E-03 | 2.92E-03 |
| KEGG_PATHWAY | hsa05145:Toxoplasmosis | 5 | 6.17 | 1.08E-03 | HLA-DRB5, HLA-DPB1, HLA-DRA, HLA-DQA2, HLA-DPA1 | 36 | 112 | 8534 | 1.06E+01 | 9.43E-02 | 4.12E-03 | 3.09E-03 |
| GOTERM_CC_DIRECT | GO:1904724~tertiary granule lumen | 4 | 4.94 | 1.22E-03 | CST3, PPBP, LYZ, CTSS | 79 | 56 | 20795 | 1.88E+01 | 1.94E-01 | 9.81E-03 | 8.37E-03 |
| GOTERM_CC_DIRECT | GO:1904813~ficolin-1-rich granule lumen | 5 | 6.17 | 1.30E-03 | FCN1, CST3, MNDA, CTSS, VCL | 79 | 126 | 20795 | 1.04E+01 | 2.05E-01 | 9.98E-03 | 8.51E-03 |
| KEGG_PATHWAY | hsa05169:Epstein-Barr virus infection | 6 | 7.41 | 1.35E-03 | HLA-DRB5, HLA-DPB1, HLA-DRA, FAS, HLA-DQA2, HLA-DPA1 | 36 | 204 | 8534 | 6.97E+00 | 1.16E-01 | 4.95E-03 | 3.71E-03 |
| UP_SEQ_FEATURE | REGION:Alpha-1 | 3 | 3.70 | 1.59E-03 | HLA-DRA, HLA-DQA2, HLA-DPA1 | 78 | 16 | 20675 | 4.97E+01 | 4.76E-01 | 4.61E-02 | 4.55E-02 |
| UP_SEQ_FEATURE | REGION:Alpha-2 | 3 | 3.70 | 1.59E-03 | HLA-DRA, HLA-DQA2, HLA-DPA1 | 78 | 16 | 20675 | 4.97E+01 | 4.76E-01 | 4.61E-02 | 4.55E-02 |
| UP_KW_BIOLOGICAL_PROCESS | KW-0399~Innate immunity | 9 | 11.11 | 1.67E-03 | FCN1, LGALS3, TRDV2, SLAMF7, KLRD1, KLRC1, S100A9, S100A8, KLRG1 | 61 | 431 | 11523 | 3.94E+00 | 4.08E-02 | 1.39E-02 | 1.28E-02 |
| GOTERM_BP_DIRECT | GO:0030593~neutrophil chemotaxis | 4 | 4.94 | 1.71E-03 | LGALS3, PPBP, S100A9, S100A8 | 79 | 59 | 19478 | 1.67E+01 | 5.93E-01 | 6.90E-02 | 6.74E-02 |
| UP_KW_PTM | KW-0873~Pyrrolidone carboxylic acid | 5 | 6.17 | 1.91E-03 | IGLV2-11, IGLV1-40, IGLV2-14, IGLV2-18, JCHAIN | 75 | 102 | 14316 | 9.36E+00 | 2.82E-02 | 1.53E-02 | 1.53E-02 |
| GOTERM_BP_DIRECT | GO:0061844~antimicrobial humoral immune response mediated by antimicrobial peptide | 5 | 6.17 | 2.12E-03 | LGALS3, GNLY, CCL4, PPBP, S100A9 | 79 | 135 | 19478 | 9.13E+00 | 6.71E-01 | 7.94E-02 | 7.76E-02 |
| GOTERM_BP_DIRECT | GO:0003094~glomerular filtration | 3 | 3.70 | 2.87E-03 | IGHA1, IGKV3-20, JCHAIN | 79 | 20 | 19478 | 3.70E+01 | 7.79E-01 | 1.00E-01 | 9.82E-02 |
| GOTERM_CC_DIRECT | GO:0005770~late endosome | 5 | 6.17 | 3.17E-03 | GRN, F2R, PSAP, CST7, CTSS | 79 | 161 | 20795 | 8.17E+00 | 4.29E-01 | 2.33E-02 | 1.99E-02 |
| INTERPRO | IPR033992:NKR-like_CTLD | 3 | 3.70 | 3.54E-03 | KLRD1, KLRC1, KLRG1 | 78 | 24 | 20808 | 3.33E+01 | 5.71E-01 | 5.64E-02 | 5.31E-02 |
| GOTERM_BP_DIRECT | GO:0002223~stimulatory C-type lectin receptor signaling pathway | 3 | 3.70 | 3.79E-03 | TYROBP, KLRD1, KLRC1 | 79 | 23 | 19478 | 3.22E+01 | 8.64E-01 | 1.24E-01 | 1.22E-01 |
| UP_SEQ_FEATURE | CARBOHYD:N-linked (GlcNAc...) asparagine | 28 | 34.57 | 3.96E-03 | FCN1, GRN, TRBV4-2, PRF1, AOAH, CST7, CTSS, FCGR3A, PSAP, SLAMF7, TRAV14DV4, KLRC1, HLA-DQA2, PTGDR, HLA-DPA1, KLRG1, HLA-DRB5, FCRL3, GZMA, F2R, TNFRSF1B, TGFBR3, ERN1, VCAN, HLA-DPB1, FAS, HLA-DRA, KLRD1 | 78 | 4423 | 20675 | 1.68E+00 | 8.00E-01 | 1.07E-01 | 1.06E-01 |
| UP_SEQ_FEATURE | DOMAIN:C-type lectin | 4 | 4.94 | 4.48E-03 | VCAN, KLRD1, KLRC1, KLRG1 | 78 | 89 | 20675 | 1.19E+01 | 8.39E-01 | 1.14E-01 | 1.12E-01 |
| INTERPRO | IPR001304:C-type_lectin-like | 4 | 4.94 | 4.54E-03 | VCAN, KLRD1, KLRC1, KLRG1 | 78 | 90 | 20808 | 1.19E+01 | 6.63E-01 | 6.79E-02 | 6.39E-02 |
| GOTERM_CC_DIRECT | GO:0035578~azurophil granule lumen | 4 | 4.94 | 4.87E-03 | GRN, ANXA2, MNDA, LYZ | 79 | 91 | 20795 | 1.16E+01 | 5.78E-01 | 3.45E-02 | 2.94E-02 |
| GOTERM_BP_DIRECT | GO:0042742~defense response to bacterium | 5 | 6.17 | 4.94E-03 | GNLY, PPBP, LYZ, S100A9, S100A8 | 79 | 171 | 19478 | 7.21E+00 | 9.26E-01 | 1.53E-01 | 1.49E-01 |
| UP_KW_LIGAND | KW-0106~Calcium | 9 | 11.11 | 5.80E-03 | FCN1, VCAN, TYROBP, ANXA2, CANX, PRF1, AOAH, S100A9, S100A8 | 22 | 990 | 6987 | 2.89E+00 | 6.19E-02 | 3.19E-02 | 3.19E-02 |
| GOTERM_MF_DIRECT | GO:0004888~transmembrane signaling receptor activity | 5 | 6.17 | 5.82E-03 | TGFBR3, FCRL3, FAS, KLRD1, KLRC1 | 73 | 192 | 19253 | 6.87E+00 | 6.99E-01 | 2.00E-01 | 1.97E-01 |
| INTERPRO | IPR016186:C-type_lectin-like/link_sf | 4 | 4.94 | 7.15E-03 | VCAN, KLRD1, KLRC1, KLRG1 | 78 | 106 | 20808 | 1.01E+01 | 8.20E-01 | 1.01E-01 | 9.47E-02 |
| GOTERM_MF_DIRECT | GO:0062082~HLA-E specific inhibitory MHC class Ib receptor activity | 2 | 2.47 | 7.47E-03 | KLRD1, KLRC1 | 73 | 2 | 19253 | 2.64E+02 | 7.86E-01 | 2.20E-01 | 2.17E-01 |
| GOTERM_CC_DIRECT | GO:1990660~calprotectin complex | 2 | 2.47 | 7.49E-03 | S100A9, S100A8 | 79 | 2 | 20795 | 2.63E+02 | 7.36E-01 | 5.10E-02 | 4.35E-02 |
| GOTERM_BP_DIRECT | GO:0070488~neutrophil aggregation | 2 | 2.47 | 7.99E-03 | S100A9, S100A8 | 79 | 2 | 19478 | 2.47E+02 | 9.85E-01 | 2.33E-01 | 2.28E-01 |
| GOTERM_CC_DIRECT | GO:0045121~membrane raft | 5 | 6.17 | 8.19E-03 | AHNAK, ANXA2, FAS, TNFRSF1B, VCL | 79 | 211 | 20795 | 6.24E+00 | 7.67E-01 | 5.37E-02 | 4.58E-02 |
| GOTERM_MF_DIRECT | GO:0002020~protease binding | 4 | 4.94 | 8.54E-03 | CST3, ANXA2, PSAP, CST7 | 73 | 112 | 19253 | 9.42E+00 | 8.29E-01 | 2.20E-01 | 2.17E-01 |
| INTERPRO | IPR016187:CTDL_fold | 4 | 4.94 | 8.73E-03 | VCAN, KLRD1, KLRC1, KLRG1 | 78 | 114 | 20808 | 9.36E+00 | 8.77E-01 | 1.16E-01 | 1.09E-01 |
| GOTERM_BP_DIRECT | GO:0050832~defense response to fungus | 3 | 3.70 | 9.13E-03 | GNLY, S100A9, S100A8 | 79 | 36 | 19478 | 2.05E+01 | 9.92E-01 | 2.52E-01 | 2.47E-01 |
| GOTERM_CC_DIRECT | GO:0034774~secretory granule lumen | 4 | 4.94 | 9.95E-03 | FCN1, S100A9, VCL, S100A8 | 79 | 118 | 20795 | 8.92E+00 | 8.30E-01 | 6.29E-02 | 5.37E-02 |
| GOTERM_BP_DIRECT | GO:0050729~positive regulation of inflammatory response | 4 | 4.94 | 1.03E-02 | MAP3K8, NEAT1, S100A9, S100A8 | 79 | 112 | 19478 | 8.81E+00 | 9.96E-01 | 2.70E-01 | 2.64E-01 |
| UP_KW_MOLECULAR_FUNCTION | KW-0675~Receptor | 14 | 17.28 | 1.09E-02 | TRDV2, TRBV4-2, FCRL3, F2R, TNFRSF1B, TGFBR3, FCGR3A, FAS, TRAV14DV4, SLAMF7, KLRD1, KLRC1, PTGDR, KLRG1 | 43 | 1898 | 11952 | 2.05E+00 | 3.25E-01 | 1.96E-01 | 1.96E-01 |
| GOTERM_CC_DIRECT | GO:0071756~pentameric IgM immunoglobulin complex | 2 | 2.47 | 1.12E-02 | IGKV3-20, JCHAIN | 79 | 3 | 20795 | 1.75E+02 | 8.64E-01 | 6.61E-02 | 5.64E-02 |
| GOTERM_CC_DIRECT | GO:0071752~secretory dimeric IgA immunoglobulin complex | 2 | 2.47 | 1.12E-02 | IGHA1, JCHAIN | 79 | 3 | 20795 | 1.75E+02 | 8.64E-01 | 6.61E-02 | 5.64E-02 |
| GOTERM_BP_DIRECT | GO:0043542~endothelial cell migration | 3 | 3.70 | 1.17E-02 | ZEB2, S100A9, S100A8 | 79 | 41 | 19478 | 1.80E+01 | 9.98E-01 | 2.73E-01 | 2.67E-01 |
| GOTERM_BP_DIRECT | GO:0002577~regulation of antigen processing and presentation | 2 | 2.47 | 1.20E-02 | CST7, CTSS | 79 | 3 | 19478 | 1.64E+02 | 9.98E-01 | 2.73E-01 | 2.67E-01 |
| GOTERM_BP_DIRECT | GO:0002228~natural killer cell mediated immunity | 2 | 2.47 | 1.20E-02 | TYROBP, KLRD1 | 79 | 3 | 19478 | 1.64E+02 | 9.98E-01 | 2.73E-01 | 2.67E-01 |
| GOTERM_BP_DIRECT | GO:0007166~cell surface receptor signaling pathway | 6 | 7.41 | 1.28E-02 | FCGR3A, TRBV4-2, FCRL3, KLRD1, KLRC1, KLRG1 | 79 | 347 | 19478 | 4.26E+00 | 9.99E-01 | 2.79E-01 | 2.73E-01 |
| INTERPRO | IPR008138:SapB_2 | 2 | 2.47 | 1.47E-02 | GNLY, PSAP | 78 | 4 | 20808 | 1.33E+02 | 9.71E-01 | 1.85E-01 | 1.74E-01 |
| GOTERM_MF_DIRECT | GO:0035662~Toll-like receptor 4 binding | 2 | 2.47 | 1.49E-02 | S100A9, S100A8 | 73 | 4 | 19253 | 1.32E+02 | 9.54E-01 | 3.40E-01 | 3.36E-01 |
| GOTERM_BP_DIRECT | GO:1905686~positive regulation of plasma membrane repair | 2 | 2.47 | 1.59E-02 | AHNAK, ANXA2 | 79 | 4 | 19478 | 1.23E+02 | 1.00E+00 | 3.33E-01 | 3.25E-01 |
| UP_KW_BIOLOGICAL_PROCESS | KW-0145~Chemotaxis | 4 | 4.94 | 1.60E-02 | CCL4, PPBP, S100A9, S100A8 | 61 | 102 | 11523 | 7.41E+00 | 3.32E-01 | 1.00E-01 | 9.20E-02 |
| GOTERM_BP_DIRECT | GO:0070527~platelet aggregation | 3 | 3.70 | 1.65E-02 | PLEK, TLN1, VCL | 79 | 49 | 19478 | 1.51E+01 | 1.00E+00 | 3.33E-01 | 3.25E-01 |
| KEGG_PATHWAY | hsa04210:Apoptosis | 4 | 4.94 | 1.79E-02 | ERN1, PRF1, FAS, CTSS | 36 | 136 | 8534 | 6.97E+00 | 8.10E-01 | 6.32E-02 | 4.74E-02 |
| SMART | SM00034:CLECT | 4 | 4.94 | 1.83E-02 | VCAN, KLRD1, KLRC1, KLRG1 | 69 | 88 | 10706 | 7.05E+00 | 6.11E-01 | 1.56E-01 | 1.53E-01 |
| GOTERM_BP_DIRECT | GO:0051603~proteolysis involved in protein catabolic process | 3 | 3.70 | 1.91E-02 | HSPA5, GZMA, CTSS | 79 | 53 | 19478 | 1.40E+01 | 1.00E+00 | 3.72E-01 | 3.63E-01 |
| GOTERM_BP_DIRECT | GO:0002282~microglial cell activation involved in immune response | 2 | 2.47 | 1.99E-02 | GRN, TYROBP | 79 | 5 | 19478 | 9.86E+01 | 1.00E+00 | 3.72E-01 | 3.64E-01 |
| GOTERM_MF_DIRECT | GO:0005509~calcium ion binding | 8 | 9.88 | 2.03E-02 | VCAN, HSPA5, ANXA2, CANX, PRF1, AOAH, S100A9, S100A8 | 73 | 739 | 19253 | 2.86E+00 | 9.85E-01 | 4.10E-01 | 4.04E-01 |
| GOTERM_MF_DIRECT | GO:0042803~protein homodimerization activity | 8 | 9.88 | 2.23E-02 | ERN1, TYROBP, ZBTB38, GZMA, PSAP, PLEK, CST7, JCHAIN | 73 | 753 | 19253 | 2.80E+00 | 9.90E-01 | 4.10E-01 | 4.04E-01 |
| GOTERM_BP_DIRECT | GO:0050853~B cell receptor signaling pathway | 3 | 3.70 | 2.34E-02 | IGKC, MNDA, IGHA1 | 79 | 59 | 19478 | 1.25E+01 | 1.00E+00 | 4.16E-01 | 4.07E-01 |
| GOTERM_BP_DIRECT | GO:0032814~regulation of natural killer cell activation | 2 | 2.47 | 2.38E-02 | KLRD1, KLRC1 | 79 | 6 | 19478 | 8.22E+01 | 1.00E+00 | 4.16E-01 | 4.07E-01 |
| GOTERM_CC_DIRECT | GO:0010008~endosome membrane | 5 | 6.17 | 2.42E-02 | HLA-DRB5, HLA-DPB1, HLA-DRA, HLA-DQA2, HLA-DPA1 | 79 | 292 | 20795 | 4.51E+00 | 9.87E-01 | 1.38E-01 | 1.18E-01 |
| INTERPRO | IPR036723:Alpha-catenin/vinculin-like_sf | 2 | 2.47 | 2.56E-02 | TLN1, VCL | 78 | 7 | 20808 | 7.62E+01 | 9.98E-01 | 2.92E-01 | 2.75E-01 |
| INTERPRO | IPR050919:NKG2/CD94_NK_receptors | 2 | 2.47 | 2.56E-02 | KLRD1, KLRC1 | 78 | 7 | 20808 | 7.62E+01 | 9.98E-01 | 2.92E-01 | 2.75E-01 |
| GOTERM_MF_DIRECT | GO:0023024~MHC class I protein complex binding | 2 | 2.47 | 2.59E-02 | KLRD1, KLRC1 | 73 | 7 | 19253 | 7.54E+01 | 9.95E-01 | 4.10E-01 | 4.04E-01 |
| GOTERM_MF_DIRECT | GO:0050544~arachidonate binding | 2 | 2.47 | 2.59E-02 | S100A9, S100A8 | 73 | 7 | 19253 | 7.54E+01 | 9.95E-01 | 4.10E-01 | 4.04E-01 |
| GOTERM_CC_DIRECT | GO:0071745~IgA immunoglobulin complex | 2 | 2.47 | 2.60E-02 | IGKC, IGHA1 | 79 | 7 | 20795 | 7.52E+01 | 9.91E-01 | 1.44E-01 | 1.23E-01 |
| UP_SEQ_FEATURE | TOPO_DOM:Extracellular | 19 | 23.46 | 2.61E-02 | HLA-DRB5, APLP2, FCRL3, F2R, TNFRSF1B, TGFBR3, FCGR3A, TYROBP, HLA-DPB1, FAS, HLA-DRA, SLAMF7, KLRD1, KLRC1, IGHA1, HLA-DQA2, PTGDR, HLA-DPA1, KLRG1 | 78 | 3012 | 20675 | 1.67E+00 | 1.00E+00 | 6.25E-01 | 6.17E-01 |
| GOTERM_BP_DIRECT | GO:0045087~innate immune response | 7 | 8.64 | 2.67E-02 | LGALS3, TRDV2, KLRC1, S100A9, S100A8, JCHAIN, KLRG1 | 79 | 570 | 19478 | 3.03E+00 | 1.00E+00 | 4.41E-01 | 4.31E-01 |
| GOTERM_BP_DIRECT | GO:0035425~autocrine signaling | 2 | 2.47 | 2.77E-02 | S100A9, S100A8 | 79 | 7 | 19478 | 7.04E+01 | 1.00E+00 | 4.41E-01 | 4.31E-01 |
| GOTERM_BP_DIRECT | GO:0060267~positive regulation of respiratory burst | 2 | 2.47 | 2.77E-02 | IGHA1, JCHAIN | 79 | 7 | 19478 | 7.04E+01 | 1.00E+00 | 4.41E-01 | 4.31E-01 |
| GOTERM_MF_DIRECT | GO:0042802~identical protein binding | 13 | 16.05 | 3.13E-02 | ANXA2, AHNAK, APLP2, PRF1, LYZ, MTSS1, ERN1, CST3, TYROBP, CCL4, PSAP, FAS, SLAMF7 | 73 | 1777 | 19253 | 1.93E+00 | 9.99E-01 | 4.60E-01 | 4.53E-01 |
| GOTERM_BP_DIRECT | GO:0036498~IRE1-mediated unfolded protein response | 2 | 2.47 | 3.16E-02 | ERN1, HSPA5 | 79 | 8 | 19478 | 6.16E+01 | 1.00E+00 | 4.48E-01 | 4.38E-01 |
| GOTERM_BP_DIRECT | GO:1903979~negative regulation of microglial cell activation | 2 | 2.47 | 3.16E-02 | GRN, CST7 | 79 | 8 | 19478 | 6.16E+01 | 1.00E+00 | 4.48E-01 | 4.38E-01 |
| GOTERM_BP_DIRECT | GO:0010466~negative regulation of peptidase activity | 2 | 2.47 | 3.16E-02 | CST3, CST7 | 79 | 8 | 19478 | 6.16E+01 | 1.00E+00 | 4.48E-01 | 4.38E-01 |
| GOTERM_BP_DIRECT | GO:0001915~negative regulation of T cell mediated cytotoxicity | 2 | 2.47 | 3.16E-02 | KLRD1, KLRC1 | 79 | 8 | 19478 | 6.16E+01 | 1.00E+00 | 4.48E-01 | 4.38E-01 |
| GOTERM_CC_DIRECT | GO:0005788~endoplasmic reticulum lumen | 5 | 6.17 | 3.17E-02 | CST3, VCAN, HSPA5, APLP2, CANX | 79 | 318 | 20795 | 4.14E+00 | 9.97E-01 | 1.65E-01 | 1.41E-01 |
| GOTERM_CC_DIRECT | GO:0005764~lysosome | 5 | 6.17 | 3.17E-02 | GRN, PSAP, HLA-DRA, CST7, CTSS | 79 | 318 | 20795 | 4.14E+00 | 9.97E-01 | 1.65E-01 | 1.41E-01 |
| UP_SEQ_FEATURE | REGION:Beta-2 | 2 | 2.47 | 3.30E-02 | HLA-DRB5, HLA-DPB1 | 78 | 9 | 20675 | 5.89E+01 | 1.00E+00 | 6.47E-01 | 6.39E-01 |
| UP_SEQ_FEATURE | SITE:Reactive site | 2 | 2.47 | 3.30E-02 | CST3, CST7 | 78 | 9 | 20675 | 5.89E+01 | 1.00E+00 | 6.47E-01 | 6.39E-01 |
| UP_SEQ_FEATURE | REGION:Beta-1 | 2 | 2.47 | 3.30E-02 | HLA-DRB5, HLA-DPB1 | 78 | 9 | 20675 | 5.89E+01 | 1.00E+00 | 6.47E-01 | 6.39E-01 |
| UP_SEQ_FEATURE | PROPEP:Activation peptide | 3 | 3.70 | 3.35E-02 | GZMK, GZMA, CTSS | 78 | 77 | 20675 | 1.03E+01 | 1.00E+00 | 6.47E-01 | 6.39E-01 |
| UP_SEQ_FEATURE | MOTIF:Secondary area of contact | 2 | 2.47 | 3.66E-02 | CST3, CST7 | 78 | 10 | 20675 | 5.30E+01 | 1.00E+00 | 6.76E-01 | 6.68E-01 |
| GOTERM_MF_DIRECT | GO:0005031~tumor necrosis factor receptor activity | 2 | 2.47 | 3.68E-02 | FAS, TNFRSF1B | 73 | 10 | 19253 | 5.27E+01 | 1.00E+00 | 4.87E-01 | 4.80E-01 |
| GOTERM_BP_DIRECT | GO:0019731~antibacterial humoral response | 3 | 3.70 | 3.73E-02 | IGHA1, IGKV3-20, JCHAIN | 79 | 76 | 19478 | 9.73E+00 | 1.00E+00 | 5.15E-01 | 5.03E-01 |
| GOTERM_MF_DIRECT | GO:0048306~calcium-dependent protein binding | 3 | 3.70 | 3.78E-02 | ANXA2, S100A9, S100A8 | 73 | 82 | 19253 | 9.65E+00 | 1.00E+00 | 4.87E-01 | 4.80E-01 |
| GOTERM_BP_DIRECT | GO:0002544~chronic inflammatory response | 2 | 2.47 | 3.93E-02 | S100A9, S100A8 | 79 | 10 | 19478 | 4.93E+01 | 1.00E+00 | 5.30E-01 | 5.17E-01 |
| GOTERM_MF_DIRECT | GO:0050786~RAGE receptor binding | 2 | 2.47 | 4.04E-02 | S100A9, S100A8 | 73 | 11 | 19253 | 4.80E+01 | 1.00E+00 | 4.89E-01 | 4.82E-01 |
| GOTERM_CC_DIRECT | GO:0044194~cytolytic granule | 2 | 2.47 | 4.05E-02 | GNLY, PRF1 | 79 | 11 | 20795 | 4.79E+01 | 9.99E-01 | 2.05E-01 | 1.75E-01 |
| GOTERM_BP_DIRECT | GO:0070493~thrombin-activated receptor signaling pathway | 2 | 2.47 | 4.32E-02 | F2R, PLEK | 79 | 11 | 19478 | 4.48E+01 | 1.00E+00 | 5.53E-01 | 5.40E-01 |
| GOTERM_BP_DIRECT | GO:0034975~protein folding in endoplasmic reticulum | 2 | 2.47 | 4.32E-02 | HSPA5, CANX | 79 | 11 | 19478 | 4.48E+01 | 1.00E+00 | 5.53E-01 | 5.40E-01 |
| GOTERM_BP_DIRECT | GO:0034121~regulation of toll-like receptor signaling pathway | 2 | 2.47 | 4.70E-02 | S100A9, S100A8 | 79 | 12 | 19478 | 4.11E+01 | 1.00E+00 | 5.88E-01 | 5.74E-01 |
| UP_SEQ_FEATURE | TOPO_DOM:Cytoplasmic | 22 | 27.16 | 4.77E-02 | HLA-DRB5, APLP2, FCRL3, F2R, TNFRSF1B, ERN1, TGFBR3, FCGR3A, TYROBP, GOLGB1, CANX, HLA-DPB1, FAS, HLA-DRA, SLAMF7, KLRD1, KLRC1, IGHA1, HLA-DQA2, PTGDR, HLA-DPA1, KLRG1 | 78 | 3923 | 20675 | 1.49E+00 | 1.00E+00 | 8.41E-01 | 8.31E-01 |
| GOTERM_CC_DIRECT | GO:0071735~IgG immunoglobulin complex | 2 | 2.47 | 4.77E-02 | IGKC, IGHA1 | 79 | 13 | 20795 | 4.05E+01 | 1.00E+00 | 2.34E-01 | 2.00E-01 |
| GOTERM_BP_DIRECT | GO:0032233~positive regulation of actin filament bundle assembly | 2 | 2.47 | 5.08E-02 | PLEK, MTSS1 | 79 | 13 | 19478 | 3.79E+01 | 1.00E+00 | 6.21E-01 | 6.07E-01 |
| GOTERM_MF_DIRECT | GO:0044548~S100 protein binding | 2 | 2.47 | 5.11E-02 | AHNAK, ANXA2 | 73 | 14 | 19253 | 3.77E+01 | 1.00E+00 | 5.85E-01 | 5.77E-01 |
| GOTERM_CC_DIRECT | GO:0043202~lysosomal lumen | 3 | 3.70 | 5.24E-02 | VCAN, PSAP, CTSS | 79 | 98 | 20795 | 8.06E+00 | 1.00E+00 | 2.51E-01 | 2.14E-01 |
| UP_SEQ_FEATURE | DOMAIN:MHC class II beta chain N-terminal | 2 | 2.47 | 5.44E-02 | HLA-DRB5, HLA-DPB1 | 78 | 15 | 20675 | 3.53E+01 | 1.00E+00 | 9.21E-01 | 9.10E-01 |
| GOTERM_BP_DIRECT | GO:0002523~leukocyte migration involved in inflammatory response | 2 | 2.47 | 5.46E-02 | S100A9, S100A8 | 79 | 14 | 19478 | 3.52E+01 | 1.00E+00 | 6.52E-01 | 6.37E-01 |
| GOTERM_MF_DIRECT | GO:0019900~kinase binding | 3 | 3.70 | 5.59E-02 | FCRL3, FAS, MYOM2 | 73 | 102 | 19253 | 7.76E+00 | 1.00E+00 | 6.06E-01 | 5.97E-01 |
| GOTERM_CC_DIRECT | GO:0042383~sarcolemma | 3 | 3.70 | 6.02E-02 | AHNAK, ANXA2, VCL | 79 | 106 | 20795 | 7.45E+00 | 1.00E+00 | 2.80E-01 | 2.39E-01 |
| GOTERM_BP_DIRECT | GO:0030036~actin cytoskeleton organization | 4 | 4.94 | 6.10E-02 | TYROBP, PLEK, TLN1, MTSS1 | 79 | 224 | 19478 | 4.40E+00 | 1.00E+00 | 7.11E-01 | 6.95E-01 |
| UP_SEQ_FEATURE | DOMAIN:TNFR-Cys | 2 | 2.47 | 6.15E-02 | FAS, TNFRSF1B | 78 | 17 | 20675 | 3.12E+01 | 1.00E+00 | 9.99E-01 | 9.86E-01 |
| GOTERM_CC_DIRECT | GO:0098797~plasma membrane protein complex | 2 | 2.47 | 6.19E-02 | AHNAK, ANXA2 | 79 | 17 | 20795 | 3.10E+01 | 1.00E+00 | 2.81E-01 | 2.40E-01 |
| KEGG_PATHWAY | hsa04061:Viral protein interaction with cytokine and cytokine receptor | 3 | 3.70 | 6.30E-02 | CCL4, PPBP, TNFRSF1B | 36 | 100 | 8534 | 7.11E+00 | 9.97E-01 | 2.15E-01 | 1.61E-01 |
| INTERPRO | IPR000010:Cystatin_dom | 2 | 2.47 | 6.46E-02 | CST3, CST7 | 78 | 18 | 20808 | 2.96E+01 | 1.00E+00 | 6.71E-01 | 6.32E-01 |
| INTERPRO | IPR000353:MHC_II_b_N | 2 | 2.47 | 6.46E-02 | HLA-DRB5, HLA-DPB1 | 78 | 18 | 20808 | 2.96E+01 | 1.00E+00 | 6.71E-01 | 6.32E-01 |
| UP_SEQ_FEATURE | REPEAT:TNFR-Cys | 2 | 2.47 | 6.50E-02 | FAS, TNFRSF1B | 78 | 18 | 20675 | 2.95E+01 | 1.00E+00 | 1.00E+00 | 9.90E-01 |
| GOTERM_CC_DIRECT | GO:0043034~costamere | 2 | 2.47 | 6.54E-02 | AHNAK, VCL | 79 | 18 | 20795 | 2.92E+01 | 1.00E+00 | 2.90E-01 | 2.47E-01 |
| GOTERM_BP_DIRECT | GO:0042129~regulation of T cell proliferation | 2 | 2.47 | 6.60E-02 | LGALS3, TNFRSF1B | 79 | 17 | 19478 | 2.90E+01 | 1.00E+00 | 7.37E-01 | 7.20E-01 |
| GOTERM_BP_DIRECT | GO:0014002~astrocyte development | 2 | 2.47 | 6.60E-02 | S100A9, S100A8 | 79 | 17 | 19478 | 2.90E+01 | 1.00E+00 | 7.37E-01 | 7.20E-01 |
| GOTERM_BP_DIRECT | GO:0016064~immunoglobulin mediated immune response | 3 | 3.70 | 6.75E-02 | IGKC, IGHV3-23, IGHV4-39 | 79 | 106 | 19478 | 6.98E+00 | 1.00E+00 | 7.38E-01 | 7.21E-01 |
| UP_KW_BIOLOGICAL_PROCESS | KW-0945~Host-virus interaction | 8 | 9.88 | 6.81E-02 | HSPA5, ANXA2, CANX, HLA-DRA, KLRD1, KLRC1, TLN1, TENT5C | 61 | 695 | 11523 | 2.17E+00 | 8.29E-01 | 3.41E-01 | 3.13E-01 |
| KEGG_PATHWAY | hsa04820:Cytoskeleton in muscle cells | 4 | 4.94 | 6.87E-02 | VCAN, TLN1, MYOM2, VCL | 36 | 232 | 8534 | 4.09E+00 | 9.99E-01 | 2.26E-01 | 1.69E-01 |
| GOTERM_MF_DIRECT | GO:0034987~immunoglobulin receptor binding | 2 | 2.47 | 6.87E-02 | IGHA1, JCHAIN | 73 | 19 | 19253 | 2.78E+01 | 1.00E+00 | 7.08E-01 | 6.98E-01 |
| GOTERM_BP_DIRECT | GO:0030889~negative regulation of B cell proliferation | 2 | 2.47 | 6.97E-02 | TYROBP, MNDA | 79 | 18 | 19478 | 2.74E+01 | 1.00E+00 | 7.47E-01 | 7.30E-01 |
| UP_KW_CELLULAR_COMPONENT | KW-0967~Endosome | 7 | 8.64 | 7.14E-02 | HLA-DRB5, RAB11FIP1, HLA-DPB1, PRF1, HLA-DRA, HLA-DQA2, HLA-DPA1 | 76 | 702 | 18049 | 2.37E+00 | 8.31E-01 | 2.45E-01 | 2.04E-01 |
| INTERPRO | IPR050488:Ig_Fc_receptor | 2 | 2.47 | 7.15E-02 | FCGR3A, FCRL3 | 78 | 20 | 20808 | 2.67E+01 | 1.00E+00 | 6.84E-01 | 6.43E-01 |
| INTERPRO | IPR050380:Immune_Resp_Modulators | 2 | 2.47 | 7.15E-02 | IGKC, IGHA1 | 78 | 20 | 20808 | 2.67E+01 | 1.00E+00 | 6.84E-01 | 6.43E-01 |
| GOTERM_MF_DIRECT | GO:0030414~peptidase inhibitor activity | 2 | 2.47 | 7.22E-02 | CST3, CST7 | 73 | 20 | 19253 | 2.64E+01 | 1.00E+00 | 7.09E-01 | 6.98E-01 |
| GOTERM_BP_DIRECT | GO:0031643~positive regulation of myelination | 2 | 2.47 | 7.34E-02 | CST7, TNFRSF1B | 79 | 19 | 19478 | 2.60E+01 | 1.00E+00 | 7.71E-01 | 7.53E-01 |
| UP_KW_MOLECULAR_FUNCTION | KW-0646~Protease inhibitor | 3 | 3.70 | 7.83E-02 | CST3, APLP2, CST7 | 43 | 132 | 11952 | 6.32E+00 | 9.47E-01 | 7.88E-01 | 7.88E-01 |
| UP_SEQ_FEATURE | CARBOHYD:N-linked (GlcNAc...) (complex) asparagine | 3 | 3.70 | 8.03E-02 | PSAP, IGHA1, JCHAIN | 78 | 126 | 20675 | 6.31E+00 | 1.00E+00 | 1.00E+00 | 9.90E-01 |
| BIOCARTA | h_bbcellPathway:Bystander B Cell Activation | 2 | 2.47 | 8.04E-02 | HLA-DRA, FAS | 16 | 9 | 1622 | 2.25E+01 | 9.86E-01 | 1.00E+00 | 1.00E+00 |
| GOTERM_BP_DIRECT | GO:0045953~negative regulation of natural killer cell mediated cytotoxicity | 2 | 2.47 | 8.09E-02 | KLRD1, KLRC1 | 79 | 21 | 19478 | 2.35E+01 | 1.00E+00 | 8.32E-01 | 8.13E-01 |
| UP_SEQ_FEATURE | REPEAT:TNFR-Cys 3 | 2 | 2.47 | 8.23E-02 | FAS, TNFRSF1B | 78 | 23 | 20675 | 2.30E+01 | 1.00E+00 | 1.00E+00 | 9.90E-01 |
| GOTERM_MF_DIRECT | GO:0051082~unfolded protein binding | 3 | 3.70 | 8.41E-02 | ERN1, HSPA5, CANX | 73 | 129 | 19253 | 6.13E+00 | 1.00E+00 | 7.68E-01 | 7.57E-01 |
| UP_KW_DISEASE | KW-1008~Amyloidosis | 2 | 2.47 | 8.49E-02 | CST3, LYZ | 14 | 33 | 4859 | 2.10E+01 | 8.58E-01 | 1.00E+00 | 1.00E+00 |
| INTERPRO | IPR046350:Cystatin_sf | 2 | 2.47 | 8.52E-02 | CST3, CST7 | 78 | 24 | 20808 | 2.22E+01 | 1.00E+00 | 7.56E-01 | 7.12E-01 |
| KEGG_PATHWAY | hsa04668:TNF signaling pathway | 3 | 3.70 | 8.52E-02 | FAS, MAP3K8, TNFRSF1B | 36 | 119 | 8534 | 5.98E+00 | 1.00E+00 | 2.70E-01 | 2.03E-01 |
| GOTERM_MF_DIRECT | GO:0043236~laminin binding | 2 | 2.47 | 8.60E-02 | LGALS3, CTSS | 73 | 24 | 19253 | 2.20E+01 | 1.00E+00 | 7.68E-01 | 7.57E-01 |
| UP_KW_MOLECULAR_FUNCTION | KW-0789~Thiol protease inhibitor | 2 | 2.47 | 8.76E-02 | CST3, CST7 | 43 | 26 | 11952 | 2.14E+01 | 9.63E-01 | 7.88E-01 | 7.88E-01 |
| GOTERM_BP_DIRECT | GO:0007041~lysosomal transport | 2 | 2.47 | 8.82E-02 | GRN, PSAP | 79 | 23 | 19478 | 2.14E+01 | 1.00E+00 | 8.91E-01 | 8.70E-01 |
| INTERPRO | IPR001751:S100/CaBP7/8-like_CS | 2 | 2.47 | 8.86E-02 | S100A9, S100A8 | 78 | 25 | 20808 | 2.13E+01 | 1.00E+00 | 7.56E-01 | 7.12E-01 |
| INTERPRO | IPR001368:TNFR/NGFR_Cys_rich_reg | 2 | 2.47 | 8.86E-02 | FAS, TNFRSF1B | 78 | 25 | 20808 | 2.13E+01 | 1.00E+00 | 7.56E-01 | 7.12E-01 |
| UP_SEQ_FEATURE | REPEAT:TNFR-Cys 2 | 2 | 2.47 | 8.91E-02 | FAS, TNFRSF1B | 78 | 25 | 20675 | 2.12E+01 | 1.00E+00 | 1.00E+00 | 9.90E-01 |
| UP_SEQ_FEATURE | REPEAT:TNFR-Cys 1 | 2 | 2.47 | 8.91E-02 | FAS, TNFRSF1B | 78 | 25 | 20675 | 2.12E+01 | 1.00E+00 | 1.00E+00 | 9.90E-01 |
| GOTERM_MF_DIRECT | GO:0005539~glycosaminoglycan binding | 2 | 2.47 | 8.95E-02 | TGFBR3, VCAN | 73 | 25 | 19253 | 2.11E+01 | 1.00E+00 | 7.68E-01 | 7.57E-01 |
| UP_KW_BIOLOGICAL_PROCESS | KW-0204~Cytolysis | 2 | 2.47 | 8.98E-02 | GZMA, PRF1 | 61 | 18 | 11523 | 2.10E+01 | 9.05E-01 | 3.74E-01 | 3.44E-01 |
| UP_KW_PTM | KW-0325~Glycoprotein | 32 | 39.51 | 9.07E-02 | FCN1, GRN, TRBV4-2, PRF1, AOAH, CST7, CTSS, JCHAIN, CST3, FCGR3A, PSAP, SLAMF7, TRAV14DV4, KLRC1, IGHA1, HLA-DQA2, PTGDR, HLA-DPA1, KLRG1, HLA-DRB5, APLP2, FCRL3, GZMA, F2R, TNFRSF1B, TGFBR3, ERN1, VCAN, HLA-DPB1, FAS, HLA-DRA, KLRD1 | 75 | 4844 | 14316 | 1.26E+00 | 7.60E-01 | 3.61E-01 | 3.61E-01 |
| GOTERM_BP_DIRECT | GO:0051893~regulation of focal adhesion assembly | 2 | 2.47 | 9.19E-02 | TLN1, VCL | 79 | 24 | 19478 | 2.05E+01 | 1.00E+00 | 8.93E-01 | 8.73E-01 |
| GOTERM_BP_DIRECT | GO:0051493~regulation of cytoskeleton organization | 2 | 2.47 | 9.19E-02 | S100A9, S100A8 | 79 | 24 | 19478 | 2.05E+01 | 1.00E+00 | 8.93E-01 | 8.73E-01 |
| GOTERM_CC_DIRECT | GO:0016020~membrane | 27 | 33.33 | 9.21E-02 | GRN, AHNAK, PLEK, PRF1, LGALS3, RAB11FIP1, PSAP, SLAMF7, KLRC1, HLA-DQA2, PTGDR, HLA-DPA1, KLRG1, HLA-DRB5, HSPA5, ANXA2, APLP2, FCRL3, F2R, TNFRSF1B, VCAN, TYROBP, GOLGB1, CANX, HLA-DPB1, HLA-DRA, KLRD1 | 79 | 5415 | 20795 | 1.31E+00 | 1.00E+00 | 3.93E-01 | 3.35E-01 |
| UP_KW_PTM | KW-0654~Proteoglycan | 3 | 3.70 | 9.29E-02 | TGFBR3, VCAN, APLP2 | 75 | 99 | 14316 | 5.78E+00 | 7.68E-01 | 3.61E-01 | 3.61E-01 |
| GOTERM_CC_DIRECT | GO:0044291~cell-cell contact zone | 2 | 2.47 | 9.31E-02 | AHNAK, VCL | 79 | 26 | 20795 | 2.02E+01 | 1.00E+00 | 3.93E-01 | 3.35E-01 |
| UP_SEQ_FEATURE | CARBOHYD:O-linked (GalNAc...) threonine | 3 | 3.70 | 9.37E-02 | FAS, IGHA1, TNFRSF1B | 78 | 138 | 20675 | 5.76E+00 | 1.00E+00 | 1.00E+00 | 9.90E-01 |
| UP_SEQ_FEATURE | DOMAIN:Chemokine interleukin-8-like | 2 | 2.47 | 9.59E-02 | CCL4, PPBP | 78 | 27 | 20675 | 1.96E+01 | 1.00E+00 | 1.00E+00 | 9.90E-01 |
| SMART | SM00043:CY | 2 | 2.47 | 9.70E-02 | CST3, CST7 | 69 | 16 | 10706 | 1.94E+01 | 9.95E-01 | 6.91E-01 | 6.78E-01 |
| BIOCARTA | h_setPathway:Granzyme A mediated Apoptosis Pathway | 2 | 2.47 | 9.74E-02 | GZMA, PRF1 | 16 | 11 | 1622 | 1.84E+01 | 9.95E-01 | 1.00E+00 | 1.00E+00 |
| INTERPRO | IPR013787:S100_Ca-bd_sub | 2 | 2.47 | 9.87E-02 | S100A9, S100A8 | 78 | 28 | 20808 | 1.91E+01 | 1.00E+00 | 8.13E-01 | 7.65E-01 |
| UP_KW_CELLULAR_COMPONENT | KW-0034~Amyloid | 2 | 2.47 | 9.89E-02 | CST3, LYZ | 76 | 25 | 18049 | 1.90E+01 | 9.18E-01 | 2.96E-01 | 2.47E-01 |
| GOTERM_BP_DIRECT | GO:0051928~positive regulation of calcium ion transport | 2 | 2.47 | 9.91E-02 | F2R, CCL4 | 79 | 26 | 19478 | 1.90E+01 | 1.00E+00 | 9.46E-01 | 9.25E-01 |
