# Supplementary material for: Single-cell RNA sequencing unraveled immune-related expression heterogeneity and lymphoid cell development dysregulation in childhood asthma
Source: Front Immunol. 2026 Jan 2;16:1606650. doi: 10.3389/fimmu.2025.1606650 (PMC12807962; doi:10.3389/fimmu.2025.1606650)
Supplement: Supplementary file 8 [file Table7.docx]

**Supplementary Table 7.** GO results of 7 upregulated genes in CD8 T cells of Asthma 2 paitent

| Category | Term | Count | % | *P-*Value | Genes | List Total | Pop Hits | Pop Total | Fold Enrichment | Bonferroni | Benjamini | FDR |
| --- | --- | --- | --- | --- | --- | --- | --- | --- | --- | --- | --- | --- |
| GOTERM_CC_DIRECT | GO:0005576~extracellular region | 6 | 85.71 | 9.23E-05 | IGKC, CD27, LYZ, S100A9, S100A8, IGLV3-19 | 7 | 2313 | 20795 | 7.71E+00 | 2.40E-03 | 2.40E-03 | 2.03E-03 |
| UP_KW_BIOLOGICAL_PROCESS | KW-0391~Immunity | 5 | 71.43 | 2.42E-04 | TRAV19, IGKC, S100A9, S100A8, IGLV3-19 | 6 | 980 | 11523 | 9.80E+00 | 1.70E-03 | 1.70E-03 | 4.85E-04 |
| UP_KW_MOLECULAR_FUNCTION | KW-0929~Antimicrobial | 3 | 42.86 | 5.53E-04 | LYZ, S100A9, S100A8 | 5 | 116 | 11952 | 6.18E+01 | 3.31E-03 | 3.32E-03 | 3.32E-03 |
| GOTERM_CC_DIRECT | GO:1990660~calprotectin complex | 2 | 28.57 | 5.77E-04 | S100A9, S100A8 | 7 | 2 | 20795 | 2.97E+03 | 1.49E-02 | 7.50E-03 | 6.35E-03 |
| GOTERM_BP_DIRECT | GO:0070488~neutrophil aggregation | 2 | 28.57 | 6.16E-04 | S100A9, S100A8 | 7 | 2 | 19478 | 2.78E+03 | 3.15E-02 | 2.86E-02 | 2.02E-02 |
| GOTERM_BP_DIRECT | GO:0042742~defense response to bacterium | 3 | 42.86 | 1.12E-03 | LYZ, S100A9, S100A8 | 7 | 171 | 19478 | 4.88E+01 | 5.68E-02 | 2.86E-02 | 2.02E-02 |
| GOTERM_MF_DIRECT | GO:0035662~Toll-like receptor 4 binding | 2 | 28.57 | 1.25E-03 | S100A9, S100A8 | 7 | 4 | 19253 | 1.38E+03 | 1.98E-02 | 1.74E-02 | 1.42E-02 |
| GOTERM_CC_DIRECT | GO:0070062~extracellular exosome | 5 | 71.43 | 1.69E-03 | IGKC, LYZ, S100A9, S100A8, IGLV3-19 | 7 | 2242 | 20795 | 6.63E+00 | 4.30E-02 | 1.46E-02 | 1.24E-02 |
| GOTERM_BP_DIRECT | GO:0035425~autocrine signaling | 2 | 28.57 | 2.15E-03 | S100A9, S100A8 | 7 | 7 | 19478 | 7.95E+02 | 1.06E-01 | 3.66E-02 | 2.58E-02 |
| GOTERM_MF_DIRECT | GO:0050544~arachidonate binding | 2 | 28.57 | 2.18E-03 | S100A9, S100A8 | 7 | 7 | 19253 | 7.86E+02 | 3.43E-02 | 1.74E-02 | 1.42E-02 |
| UP_KW_CELLULAR_COMPONENT | KW-0964~Secreted | 5 | 71.43 | 2.77E-03 | IGKC, LYZ, S100A9, S100A8, IGLV3-19 | 7 | 2217 | 18049 | 5.82E+00 | 2.20E-02 | 1.22E-02 | 1.07E-02 |
| UP_KW_CELLULAR_COMPONENT | KW-1003~Cell membrane | 6 | 85.71 | 3.06E-03 | TRAV19, IGKC, CD27, S100A9, S100A8, IGLV3-19 | 7 | 4134 | 18049 | 3.74E+00 | 2.42E-02 | 1.22E-02 | 1.07E-02 |
| GOTERM_BP_DIRECT | GO:0002544~chronic inflammatory response | 2 | 28.57 | 3.08E-03 | S100A9, S100A8 | 7 | 10 | 19478 | 5.57E+02 | 1.48E-01 | 3.66E-02 | 2.58E-02 |
| GOTERM_MF_DIRECT | GO:0050786~RAGE receptor binding | 2 | 28.57 | 3.42E-03 | S100A9, S100A8 | 7 | 11 | 19253 | 5.00E+02 | 5.34E-02 | 1.83E-02 | 1.48E-02 |
| GOTERM_BP_DIRECT | GO:0034121~regulation of toll-like receptor signaling pathway | 2 | 28.57 | 3.69E-03 | S100A9, S100A8 | 7 | 12 | 19478 | 4.64E+02 | 1.75E-01 | 3.66E-02 | 2.58E-02 |
| GOTERM_BP_DIRECT | GO:0002523~leukocyte migration involved in inflammatory response | 2 | 28.57 | 4.31E-03 | S100A9, S100A8 | 7 | 14 | 19478 | 3.98E+02 | 2.01E-01 | 3.66E-02 | 2.58E-02 |
| GOTERM_BP_DIRECT | GO:0014002~astrocyte development | 2 | 28.57 | 5.23E-03 | S100A9, S100A8 | 7 | 17 | 19478 | 3.27E+02 | 2.38E-01 | 3.81E-02 | 2.69E-02 |
| GOTERM_CC_DIRECT | GO:0005886~plasma membrane | 6 | 85.71 | 6.57E-03 | TRAV19, IGKC, CD27, S100A9, S100A8, IGLV3-19 | 7 | 5597 | 20795 | 3.18E+00 | 1.57E-01 | 4.27E-02 | 3.61E-02 |
| GOTERM_BP_DIRECT | GO:0006954~inflammatory response | 3 | 42.86 | 6.94E-03 | LYZ, S100A9, S100A8 | 7 | 432 | 19478 | 1.93E+01 | 3.04E-01 | 4.18E-02 | 2.95E-02 |
| INTERPRO | IPR001751:S100/CaBP7/8-like_CS | 2 | 28.57 | 7.19E-03 | S100A9, S100A8 | 7 | 25 | 20808 | 2.38E+02 | 1.53E-01 | 9.25E-02 | 9.25E-02 |
| GOTERM_BP_DIRECT | GO:0051493~regulation of cytoskeleton organization | 2 | 28.57 | 7.37E-03 | S100A9, S100A8 | 7 | 24 | 19478 | 2.32E+02 | 3.19E-01 | 4.18E-02 | 2.95E-02 |
| INTERPRO | IPR013787:S100_Ca-bd_sub | 2 | 28.57 | 8.05E-03 | S100A9, S100A8 | 7 | 28 | 20808 | 2.12E+02 | 1.70E-01 | 9.25E-02 | 9.25E-02 |
| GOTERM_BP_DIRECT | GO:0002250~adaptive immune response | 3 | 42.86 | 8.93E-03 | TRAV19, IGKC, IGLV3-19 | 7 | 492 | 19478 | 1.70E+01 | 3.73E-01 | 4.33E-02 | 3.06E-02 |
| GOTERM_BP_DIRECT | GO:0006955~immune response | 3 | 42.86 | 1.06E-02 | TRAV19, IGKC, IGLV3-19 | 7 | 537 | 19478 | 1.55E+01 | 4.25E-01 | 4.33E-02 | 3.06E-02 |
| GOTERM_BP_DIRECT | GO:0050832~defense response to fungus | 2 | 28.57 | 1.10E-02 | S100A9, S100A8 | 7 | 36 | 19478 | 1.55E+02 | 4.39E-01 | 4.33E-02 | 3.06E-02 |
| GOTERM_BP_DIRECT | GO:2001244~positive regulation of intrinsic apoptotic signaling pathway | 2 | 28.57 | 1.10E-02 | S100A9, S100A8 | 7 | 36 | 19478 | 1.55E+02 | 4.39E-01 | 4.33E-02 | 3.06E-02 |
| GOTERM_CC_DIRECT | GO:0005615~extracellular space | 4 | 57.14 | 1.17E-02 | IGKC, LYZ, S100A9, S100A8 | 7 | 1867 | 20795 | 6.36E+00 | 2.64E-01 | 6.11E-02 | 5.17E-02 |
| GOTERM_BP_DIRECT | GO:0043542~endothelial cell migration | 2 | 28.57 | 1.26E-02 | S100A9, S100A8 | 7 | 41 | 19478 | 1.36E+02 | 4.82E-01 | 4.58E-02 | 3.23E-02 |
| UP_SEQ_FEATURE | DOMAIN:Ig-like | 3 | 42.86 | 1.45E-02 | TRAV19, IGKC, IGLV3-19 | 7 | 673 | 20675 | 1.32E+01 | 4.27E-01 | 5.53E-01 | 5.53E-01 |
| SMART | SM01394:S_100 | 2 | 28.57 | 1.56E-02 | S100A9, S100A8 | 7 | 28 | 10706 | 1.09E+02 | 1.04E-01 | 1.09E-01 | 1.09E-01 |
| GOTERM_BP_DIRECT | GO:0030593~neutrophil chemotaxis | 2 | 28.57 | 1.80E-02 | S100A9, S100A8 | 7 | 59 | 19478 | 9.43E+01 | 6.12E-01 | 6.13E-02 | 4.33E-02 |
| INTERPRO | IPR007110:Ig-like_dom | 3 | 42.86 | 1.85E-02 | TRAV19, IGKC, IGLV3-19 | 7 | 768 | 20808 | 1.16E+01 | 3.49E-01 | 1.35E-01 | 1.35E-01 |
| UP_KW_BIOLOGICAL_PROCESS | KW-1064~Adaptive immunity | 3 | 42.86 | 1.86E-02 | TRAV19, IGKC, IGLV3-19 | 6 | 521 | 11523 | 1.11E+01 | 1.23E-01 | 5.49E-02 | 1.86E-02 |
| INTERPRO | IPR036179:Ig-like_dom_sf | 3 | 42.86 | 2.35E-02 | TRAV19, IGKC, IGLV3-19 | 7 | 872 | 20808 | 1.02E+01 | 4.21E-01 | 1.35E-01 | 1.35E-01 |
| UP_KW_BIOLOGICAL_PROCESS | KW-0053~Apoptosis | 3 | 42.86 | 2.35E-02 | CD27, S100A9, S100A8 | 6 | 589 | 11523 | 9.78E+00 | 1.54E-01 | 5.49E-02 | 2.35E-02 |
| GOTERM_MF_DIRECT | GO:0048306~calcium-dependent protein binding | 2 | 28.57 | 2.53E-02 | S100A9, S100A8 | 7 | 82 | 19253 | 6.71E+01 | 3.36E-01 | 1.01E-01 | 8.22E-02 |
| GOTERM_BP_DIRECT | GO:0030307~positive regulation of cell growth | 2 | 28.57 | 2.71E-02 | S100A9, S100A8 | 7 | 89 | 19478 | 6.25E+01 | 7.60E-01 | 8.64E-02 | 6.10E-02 |
| UP_KW_PTM | KW-0702~S-nitrosylation | 2 | 28.57 | 2.82E-02 | S100A9, S100A8 | 7 | 68 | 14316 | 6.02E+01 | 1.08E-01 | 1.33E-01 | 1.33E-01 |
| GOTERM_BP_DIRECT | GO:0016064~immunoglobulin mediated immune response | 2 | 28.57 | 3.22E-02 | IGKC, CD27 | 7 | 106 | 19478 | 5.25E+01 | 8.18E-01 | 9.28E-02 | 6.55E-02 |
| KEGG_PATHWAY | hsa04657:IL-17 signaling pathway | 2 | 28.57 | 3.30E-02 | S100A9, S100A8 | 4 | 95 | 8534 | 4.49E+01 | 9.59E-02 | 9.91E-02 | 9.91E-02 |
| GOTERM_CC_DIRECT | GO:0034774~secretory granule lumen | 2 | 28.57 | 3.36E-02 | S100A9, S100A8 | 7 | 118 | 20795 | 5.04E+01 | 5.88E-01 | 1.45E-01 | 1.23E-01 |
| GOTERM_BP_DIRECT | GO:0050729~positive regulation of inflammatory response | 2 | 28.57 | 3.40E-02 | S100A9, S100A8 | 7 | 112 | 19478 | 4.97E+01 | 8.35E-01 | 9.28E-02 | 6.55E-02 |
| GOTERM_BP_DIRECT | GO:0045471~response to ethanol | 2 | 28.57 | 3.46E-02 | CD27, S100A8 | 7 | 114 | 19478 | 4.88E+01 | 8.40E-01 | 9.28E-02 | 6.55E-02 |
| GOTERM_BP_DIRECT | GO:0051092~positive regulation of NF-kappaB transcription factor activity | 2 | 28.57 | 3.64E-02 | S100A9, S100A8 | 7 | 120 | 19478 | 4.64E+01 | 8.55E-01 | 9.28E-02 | 6.55E-02 |
| INTERPRO | IPR013783:Ig-like_fold | 3 | 42.86 | 3.80E-02 | TRAV19, IGKC, IGLV3-19 | 7 | 1127 | 20808 | 7.91E+00 | 5.90E-01 | 1.75E-01 | 1.75E-01 |
| UP_KW_DOMAIN | KW-0393~Immunoglobulin domain | 3 | 42.86 | 4.10E-02 | TRAV19, IGKC, IGLV3-19 | 7 | 825 | 14625 | 7.60E+00 | 1.89E-01 | 2.05E-01 | 2.05E-01 |
| GOTERM_MF_DIRECT | GO:0003823~antigen binding | 2 | 28.57 | 4.28E-02 | IGKC, IGLV3-19 | 7 | 140 | 19253 | 3.93E+01 | 5.04E-01 | 1.37E-01 | 1.11E-01 |
| UP_KW_BIOLOGICAL_PROCESS | KW-0145~Chemotaxis | 2 | 28.57 | 4.35E-02 | S100A9, S100A8 | 6 | 102 | 11523 | 3.77E+01 | 2.67E-01 | 7.61E-02 | 4.35E-02 |
| GOTERM_BP_DIRECT | GO:0006914~autophagy | 2 | 28.57 | 4.89E-02 | S100A9, S100A8 | 7 | 162 | 19478 | 3.44E+01 | 9.26E-01 | 1.19E-01 | 8.38E-02 |
| INTERPRO | IPR018247:EF_Hand_1_Ca_BS | 2 | 28.57 | 5.11E-02 | S100A9, S100A8 | 7 | 181 | 20808 | 3.28E+01 | 7.01E-01 | 1.96E-01 | 1.96E-01 |
| UP_KW_PTM | KW-1015~Disulfide bond | 5 | 71.43 | 5.32E-02 | TRAV19, IGKC, CD27, LYZ, IGLV3-19 | 7 | 3956 | 14316 | 2.58E+00 | 1.96E-01 | 1.33E-01 | 1.33E-01 |
| UP_SEQ_FEATURE | DOMAIN:EF-hand 1 | 2 | 28.57 | 5.33E-02 | S100A9, S100A8 | 7 | 188 | 20675 | 3.14E+01 | 8.75E-01 | 6.83E-01 | 6.83E-01 |
| UP_SEQ_FEATURE | DOMAIN:EF-hand 2 | 2 | 28.57 | 5.39E-02 | S100A9, S100A8 | 7 | 190 | 20675 | 3.11E+01 | 8.78E-01 | 6.83E-01 | 6.83E-01 |
| GOTERM_CC_DIRECT | GO:0019814~immunoglobulin complex | 2 | 28.57 | 5.47E-02 | TRAV19, IGLV3-19 | 7 | 194 | 20795 | 3.06E+01 | 7.68E-01 | 2.03E-01 | 1.72E-01 |
| UP_KW_CELLULAR_COMPONENT | KW-1280~Immunoglobulin | 2 | 28.57 | 6.22E-02 | IGKC, IGLV3-19 | 7 | 192 | 18049 | 2.69E+01 | 4.02E-01 | 1.56E-01 | 1.37E-01 |
| INTERPRO | IPR002048:EF_hand_dom | 2 | 28.57 | 6.53E-02 | S100A9, S100A8 | 7 | 233 | 20808 | 2.55E+01 | 7.89E-01 | 2.15E-01 | 2.15E-01 |
| UP_KW_BIOLOGICAL_PROCESS | KW-0072~Autophagy | 2 | 28.57 | 7.25E-02 | S100A9, S100A8 | 6 | 172 | 11523 | 2.23E+01 | 4.09E-01 | 9.40E-02 | 7.25E-02 |
| INTERPRO | IPR011992:EF-hand-dom_pair | 2 | 28.57 | 7.70E-02 | S100A9, S100A8 | 7 | 276 | 20808 | 2.15E+01 | 8.42E-01 | 2.21E-01 | 2.21E-01 |
| UP_KW_CELLULAR_COMPONENT | KW-0472~Membrane | 6 | 85.71 | 7.82E-02 | TRAV19, IGKC, CD27, S100A9, S100A8, IGLV3-19 | 7 | 8353 | 18049 | 1.85E+00 | 4.79E-01 | 1.56E-01 | 1.37E-01 |
| UP_KW_BIOLOGICAL_PROCESS | KW-0395~Inflammatory response | 2 | 28.57 | 8.06E-02 | S100A9, S100A8 | 6 | 192 | 11523 | 2.00E+01 | 4.45E-01 | 9.40E-02 | 8.06E-02 |
| GOTERM_MF_DIRECT | GO:0008017~microtubule binding | 2 | 28.57 | 8.36E-02 | S100A9, S100A8 | 7 | 278 | 19253 | 1.98E+01 | 7.53E-01 | 2.23E-01 | 1.81E-01 |
