# Supplementary material for: Single-cell RNA sequencing unraveled immune-related expression heterogeneity and lymphoid cell development dysregulation in childhood asthma
Source: Front Immunol. 2026 Jan 2;16:1606650. doi: 10.3389/fimmu.2025.1606650 (PMC12807962; doi:10.3389/fimmu.2025.1606650)
Supplement: Supplementary file 9 [file Table8.docx]

**Supplementary Table 8.** GO results of 14 upregulated genes in CD8 T cells of Asthma 3 paitent

| Category | Term | Count | % | *P-*Value | Genes | List Total | Pop Hits | Pop Total | Fold Enrichment | Bonferroni | Benjamini | FDR |
| --- | --- | --- | --- | --- | --- | --- | --- | --- | --- | --- | --- | --- |
| GOTERM_CC_DIRECT | GO:0005576~extracellular region | 12 | 85.71 | 1.99E-09 | FCN1, CST3, VCAN, IGLV2-14, HBB, S100A12, MNDA, LYZ, IFI30, S100A9, IGKV3-20, S100A8 | 14 | 2313 | 20795 | 7.71E+00 | 1.01E-07 | 1.01E-07 | 8.94E-08 |
| UP_KW_BIOLOGICAL_PROCESS | KW-0391~Immunity | 8 | 57.14 | 9.74E-07 | FCN1, TYROBP, IGLV2-14, S100A12, IFI30, S100A9, IGKV3-20, S100A8 | 10 | 980 | 11523 | 9.41E+00 | 1.07E-05 | 1.07E-05 | 9.74E-06 |
| UP_KW_CELLULAR_COMPONENT | KW-0964~Secreted | 10 | 71.43 | 2.82E-06 | FCN1, CST3, VCAN, IGLV2-14, S100A12, LYZ, IFI30, S100A9, IGKV3-20, S100A8 | 14 | 2217 | 18049 | 5.82E+00 | 3.10E-05 | 3.10E-05 | 2.82E-05 |
| GOTERM_MF_DIRECT | GO:0050786~RAGE receptor binding | 3 | 21.43 | 2.31E-05 | S100A12, S100A9, S100A8 | 14 | 11 | 19253 | 3.75E+02 | 1.01E-03 | 1.02E-03 | 9.92E-04 |
| UP_KW_MOLECULAR_FUNCTION | KW-0929~Antimicrobial | 4 | 28.57 | 4.81E-05 | S100A12, LYZ, S100A9, S100A8 | 9 | 116 | 11952 | 4.58E+01 | 7.70E-04 | 7.70E-04 | 7.70E-04 |
| GOTERM_CC_DIRECT | GO:0034774~secretory granule lumen | 4 | 28.57 | 4.89E-05 | FCN1, S100A12, S100A9, S100A8 | 14 | 118 | 20795 | 5.04E+01 | 2.49E-03 | 7.58E-04 | 6.69E-04 |
| GOTERM_CC_DIRECT | GO:0005615~extracellular space | 8 | 57.14 | 4.91E-05 | FCN1, CST3, VCAN, HBB, LYZ, S100A9, IGKV3-20, S100A8 | 14 | 1867 | 20795 | 6.36E+00 | 2.50E-03 | 7.58E-04 | 6.69E-04 |
| GOTERM_CC_DIRECT | GO:1904813~ficolin-1-rich granule lumen | 4 | 28.57 | 5.94E-05 | FCN1, CST3, HBB, MNDA | 14 | 126 | 20795 | 4.72E+01 | 3.03E-03 | 7.58E-04 | 6.69E-04 |
| INTERPRO | IPR001751:S100/CaBP7/8-like_CS | 3 | 21.43 | 1.07E-04 | S100A12, S100A9, S100A8 | 14 | 25 | 20808 | 1.78E+02 | 5.45E-03 | 3.44E-03 | 3.31E-03 |
| INTERPRO | IPR013787:S100_Ca-bd_sub | 3 | 21.43 | 1.35E-04 | S100A12, S100A9, S100A8 | 14 | 28 | 20808 | 1.59E+02 | 6.86E-03 | 3.44E-03 | 3.31E-03 |
| GOTERM_CC_DIRECT | GO:0070062~extracellular exosome | 8 | 57.14 | 1.60E-04 | CST3, IGLV2-14, HBB, MNDA, LYZ, S100A9, IGKV3-20, S100A8 | 14 | 2242 | 20795 | 5.30E+00 | 8.11E-03 | 1.63E-03 | 1.44E-03 |
| GOTERM_BP_DIRECT | GO:0042742~defense response to bacterium | 4 | 28.57 | 1.78E-04 | S100A12, LYZ, S100A9, S100A8 | 14 | 171 | 19478 | 3.25E+01 | 2.20E-02 | 1.38E-02 | 1.25E-02 |
| SMART | SM01394:S_100 | 3 | 21.43 | 2.35E-04 | S100A12, S100A9, S100A8 | 10 | 28 | 10706 | 1.15E+02 | 3.05E-03 | 3.05E-03 | 3.05E-03 |
| GOTERM_BP_DIRECT | GO:0050832~defense response to fungus | 3 | 21.43 | 2.56E-04 | S100A12, S100A9, S100A8 | 14 | 36 | 19478 | 1.16E+02 | 3.15E-02 | 1.38E-02 | 1.25E-02 |
| UP_KW_LIGAND | KW-0106~Calcium | 6 | 42.86 | 3.00E-04 | FCN1, VCAN, TYROBP, S100A12, S100A9, S100A8 | 7 | 990 | 6987 | 6.05E+00 | 2.69E-03 | 2.70E-03 | 2.70E-03 |
| GOTERM_BP_DIRECT | GO:0043542~endothelial cell migration | 3 | 21.43 | 3.32E-04 | S100A12, S100A9, S100A8 | 14 | 41 | 19478 | 1.02E+02 | 4.07E-02 | 1.38E-02 | 1.25E-02 |
| GOTERM_CC_DIRECT | GO:1904724~tertiary granule lumen | 3 | 21.43 | 5.45E-04 | CST3, HBB, LYZ | 14 | 56 | 20795 | 7.96E+01 | 2.74E-02 | 4.63E-03 | 4.09E-03 |
| GOTERM_BP_DIRECT | GO:0030593~neutrophil chemotaxis | 3 | 21.43 | 6.89E-04 | S100A12, S100A9, S100A8 | 14 | 59 | 19478 | 7.07E+01 | 8.25E-02 | 2.15E-02 | 1.95E-02 |
| UP_KW_PTM | KW-0702~S-nitrosylation | 3 | 21.43 | 1.19E-03 | HBB, S100A9, S100A8 | 12 | 68 | 14316 | 5.26E+01 | 9.48E-03 | 1.07E-02 | 1.07E-02 |
| GOTERM_CC_DIRECT | GO:1990660~calprotectin complex | 2 | 14.29 | 1.25E-03 | S100A9, S100A8 | 14 | 2 | 20795 | 1.49E+03 | 6.18E-02 | 9.11E-03 | 8.04E-03 |
| GOTERM_BP_DIRECT | GO:0070488~neutrophil aggregation | 2 | 14.29 | 1.33E-03 | S100A9, S100A8 | 14 | 2 | 19478 | 1.39E+03 | 1.54E-01 | 3.34E-02 | 3.02E-02 |
| GOTERM_MF_DIRECT | GO:0048306~calcium-dependent protein binding | 3 | 21.43 | 1.36E-03 | S100A12, S100A9, S100A8 | 14 | 82 | 19253 | 5.03E+01 | 5.79E-02 | 2.98E-02 | 2.91E-02 |
| GOTERM_CC_DIRECT | GO:0062023~collagen-containing extracellular matrix | 4 | 28.57 | 1.59E-03 | FCN1, VCAN, S100A9, S100A8 | 14 | 387 | 20795 | 1.54E+01 | 7.80E-02 | 1.01E-02 | 8.96E-03 |
| GOTERM_BP_DIRECT | GO:0050729~positive regulation of inflammatory response | 3 | 21.43 | 2.45E-03 | S100A12, S100A9, S100A8 | 14 | 112 | 19478 | 3.73E+01 | 2.64E-01 | 4.39E-02 | 3.97E-02 |
| GOTERM_BP_DIRECT | GO:0006954~inflammatory response | 4 | 28.57 | 2.63E-03 | S100A12, LYZ, S100A9, S100A8 | 14 | 432 | 19478 | 1.29E+01 | 2.80E-01 | 4.39E-02 | 3.97E-02 |
| GOTERM_MF_DIRECT | GO:0035662~Toll-like receptor 4 binding | 2 | 14.29 | 2.70E-03 | S100A9, S100A8 | 14 | 4 | 19253 | 6.88E+02 | 1.12E-01 | 3.96E-02 | 3.87E-02 |
| GOTERM_BP_DIRECT | GO:0051092~positive regulation of NF-kappaB transcription factor activity | 3 | 21.43 | 2.81E-03 | S100A12, S100A9, S100A8 | 14 | 120 | 19478 | 3.48E+01 | 2.96E-01 | 4.39E-02 | 3.97E-02 |
| UP_KW_BIOLOGICAL_PROCESS | KW-0399~Innate immunity | 4 | 28.57 | 3.69E-03 | FCN1, S100A12, S100A9, S100A8 | 10 | 431 | 11523 | 1.07E+01 | 3.98E-02 | 2.03E-02 | 1.84E-02 |
| GOTERM_MF_DIRECT | GO:0003823~antigen binding | 3 | 21.43 | 3.89E-03 | FCN1, IGLV2-14, IGKV3-20 | 14 | 140 | 19253 | 2.95E+01 | 1.57E-01 | 4.15E-02 | 4.06E-02 |
| GOTERM_BP_DIRECT | GO:0035425~autocrine signaling | 2 | 14.29 | 4.66E-03 | S100A9, S100A8 | 14 | 7 | 19478 | 3.98E+02 | 4.42E-01 | 6.48E-02 | 5.86E-02 |
| GOTERM_MF_DIRECT | GO:0050544~arachidonate binding | 2 | 14.29 | 4.72E-03 | S100A9, S100A8 | 14 | 7 | 19253 | 3.93E+02 | 1.88E-01 | 4.15E-02 | 4.06E-02 |
| GOTERM_BP_DIRECT | GO:0045087~innate immune response | 4 | 28.57 | 5.73E-03 | PPP1R14B, S100A12, S100A9, S100A8 | 14 | 570 | 19478 | 9.76E+00 | 5.12E-01 | 7.16E-02 | 6.47E-02 |
| UP_SEQ_FEATURE | DOMAIN:EF-hand 1 | 3 | 21.43 | 6.01E-03 | S100A12, S100A9, S100A8 | 14 | 188 | 20675 | 2.36E+01 | 3.86E-01 | 2.48E-01 | 2.48E-01 |
| UP_SEQ_FEATURE | DOMAIN:EF-hand 2 | 3 | 21.43 | 6.13E-03 | S100A12, S100A9, S100A8 | 14 | 190 | 20675 | 2.33E+01 | 3.92E-01 | 2.48E-01 | 2.48E-01 |
| GOTERM_BP_DIRECT | GO:0002544~chronic inflammatory response | 2 | 14.29 | 6.66E-03 | S100A9, S100A8 | 14 | 10 | 19478 | 2.78E+02 | 5.66E-01 | 7.56E-02 | 6.84E-02 |
| GOTERM_BP_DIRECT | GO:0034121~regulation of toll-like receptor signaling pathway | 2 | 14.29 | 7.98E-03 | S100A9, S100A8 | 14 | 12 | 19478 | 2.32E+02 | 6.33E-01 | 8.31E-02 | 7.52E-02 |
| INTERPRO | IPR002048:EF_hand_dom | 3 | 21.43 | 8.98E-03 | S100A12, S100A9, S100A8 | 14 | 233 | 20808 | 1.91E+01 | 3.69E-01 | 1.53E-01 | 1.47E-01 |
| UP_KW_BIOLOGICAL_PROCESS | KW-0395~Inflammatory response | 3 | 21.43 | 9.21E-03 | S100A12, S100A9, S100A8 | 10 | 192 | 11523 | 1.80E+01 | 9.67E-02 | 3.38E-02 | 3.07E-02 |
| GOTERM_BP_DIRECT | GO:0002523~leukocyte migration involved in inflammatory response | 2 | 14.29 | 9.31E-03 | S100A9, S100A8 | 14 | 14 | 19478 | 1.99E+02 | 6.89E-01 | 8.95E-02 | 8.09E-02 |
| GOTERM_BP_DIRECT | GO:0014002~astrocyte development | 2 | 14.29 | 1.13E-02 | S100A9, S100A8 | 14 | 17 | 19478 | 1.64E+02 | 7.58E-01 | 9.96E-02 | 9.00E-02 |
| GOTERM_BP_DIRECT | GO:0030889~negative regulation of B cell proliferation | 2 | 14.29 | 1.20E-02 | TYROBP, MNDA | 14 | 18 | 19478 | 1.55E+02 | 7.78E-01 | 9.96E-02 | 9.00E-02 |
| GOTERM_MF_DIRECT | GO:0005509~calcium ion binding | 4 | 28.57 | 1.21E-02 | VCAN, S100A12, S100A9, S100A8 | 14 | 739 | 19253 | 7.44E+00 | 4.14E-01 | 8.85E-02 | 8.65E-02 |
| INTERPRO | IPR011992:EF-hand-dom_pair | 3 | 21.43 | 1.24E-02 | S100A12, S100A9, S100A8 | 14 | 276 | 20808 | 1.62E+01 | 4.71E-01 | 1.58E-01 | 1.52E-01 |
| UP_KW_PTM | KW-1015~Disulfide bond | 8 | 57.14 | 1.35E-02 | FCN1, CST3, VCAN, TYROBP, IGLV2-14, LYZ, IFI30, IGKV3-20 | 12 | 3956 | 14316 | 2.41E+00 | 1.03E-01 | 6.08E-02 | 6.08E-02 |
| GOTERM_BP_DIRECT | GO:0051493~regulation of cytoskeleton organization | 2 | 14.29 | 1.59E-02 | S100A9, S100A8 | 14 | 24 | 19478 | 1.16E+02 | 8.65E-01 | 1.24E-01 | 1.12E-01 |
| UP_KW_CELLULAR_COMPONENT | KW-0034~Amyloid | 2 | 14.29 | 1.79E-02 | CST3, LYZ | 14 | 25 | 18049 | 1.03E+02 | 1.80E-01 | 9.82E-02 | 8.93E-02 |
| UP_KW_DOMAIN | KW-0732~Signal | 9 | 64.29 | 1.89E-02 | FCN1, CST3, VCAN, TYROBP, IGLV2-14, HBB, LYZ, IFI30, IGKV3-20 | 14 | 4415 | 14625 | 2.13E+00 | 1.74E-01 | 1.89E-01 | 1.89E-01 |
| UP_KW_DISEASE | KW-1008~Amyloidosis | 2 | 14.29 | 2.02E-02 | CST3, LYZ | 4 | 33 | 4859 | 7.36E+01 | 1.15E-01 | 1.21E-01 | 1.21E-01 |
| GOTERM_BP_DIRECT | GO:2001244~positive regulation of intrinsic apoptotic signaling pathway | 2 | 14.29 | 2.38E-02 | S100A9, S100A8 | 14 | 36 | 19478 | 7.73E+01 | 9.51E-01 | 1.75E-01 | 1.58E-01 |
| INTERPRO | IPR013106:Ig_V-set | 3 | 21.43 | 3.57E-02 | VCAN, IGLV2-14, IGKV3-20 | 14 | 485 | 20808 | 9.19E+00 | 8.43E-01 | 3.58E-01 | 3.44E-01 |
| GOTERM_CC_DIRECT | GO:0005886~plasma membrane | 8 | 57.14 | 3.59E-02 | FCN1, CST3, TYROBP, IGLV2-14, S100A12, S100A9, IGKV3-20, S100A8 | 14 | 5597 | 20795 | 2.12E+00 | 8.45E-01 | 2.03E-01 | 1.79E-01 |
| GOTERM_BP_DIRECT | GO:0006968~cellular defense response | 2 | 14.29 | 3.74E-02 | TYROBP, MNDA | 14 | 57 | 19478 | 4.88E+01 | 9.91E-01 | 2.60E-01 | 2.35E-01 |
| INTERPRO | IPR003599:Ig_sub | 3 | 21.43 | 4.21E-02 | VCAN, IGLV2-14, IGKV3-20 | 14 | 531 | 20808 | 8.40E+00 | 8.88E-01 | 3.58E-01 | 3.44E-01 |
| GOTERM_BP_DIRECT | GO:0006955~immune response | 3 | 21.43 | 4.84E-02 | CST3, IGLV2-14, IGKV3-20 | 14 | 537 | 19478 | 7.77E+00 | 9.98E-01 | 3.06E-01 | 2.76E-01 |
| GOTERM_BP_DIRECT | GO:0098869~cellular oxidant detoxification | 2 | 14.29 | 4.89E-02 | HBB, S100A9 | 14 | 75 | 19478 | 3.71E+01 | 9.98E-01 | 3.06E-01 | 2.76E-01 |
| GOTERM_CC_DIRECT | GO:0005856~cytoskeleton | 3 | 21.43 | 5.16E-02 | S100A12, S100A9, S100A8 | 14 | 594 | 20795 | 7.50E+00 | 9.33E-01 | 2.53E-01 | 2.23E-01 |
| GOTERM_BP_DIRECT | GO:0002376~immune system process | 2 | 14.29 | 5.40E-02 | TYROBP, IFI30 | 14 | 83 | 19478 | 3.35E+01 | 9.99E-01 | 3.22E-01 | 2.91E-01 |
| UP_KW_CELLULAR_COMPONENT | KW-1003~Cell membrane | 7 | 50.00 | 5.51E-02 | FCN1, TYROBP, IGLV2-14, S100A12, S100A9, IGKV3-20, S100A8 | 14 | 4134 | 18049 | 2.18E+00 | 4.64E-01 | 2.02E-01 | 1.84E-01 |
| GOTERM_CC_DIRECT | GO:0035578~azurophil granule lumen | 2 | 14.29 | 5.54E-02 | MNDA, LYZ | 14 | 91 | 20795 | 3.26E+01 | 9.45E-01 | 2.53E-01 | 2.23E-01 |
| INTERPRO | IPR050150:IgV_Light_Chain | 2 | 14.29 | 5.66E-02 | IGLV2-14, IGKV3-20 | 14 | 93 | 20808 | 3.20E+01 | 9.49E-01 | 4.12E-01 | 3.96E-01 |
| GOTERM_BP_DIRECT | GO:0030307~positive regulation of cell growth | 2 | 14.29 | 5.78E-02 | S100A9, S100A8 | 14 | 89 | 19478 | 3.13E+01 | 9.99E-01 | 3.28E-01 | 2.96E-01 |
| GOTERM_CC_DIRECT | GO:0043202~lysosomal lumen | 2 | 14.29 | 5.96E-02 | VCAN, IFI30 | 14 | 98 | 20795 | 3.03E+01 | 9.56E-01 | 2.53E-01 | 2.23E-01 |
| GOTERM_BP_DIRECT | GO:0031640~killing of cells of another organism | 2 | 14.29 | 6.03E-02 | S100A12, LYZ | 14 | 93 | 19478 | 2.99E+01 | 1.00E+00 | 3.28E-01 | 2.96E-01 |
| UP_SEQ_FEATURE | DOMAIN:Ig-like | 3 | 21.43 | 6.51E-02 | VCAN, IGLV2-14, IGKV3-20 | 14 | 673 | 20675 | 6.58E+00 | 9.96E-01 | 7.71E-01 | 7.71E-01 |
| SMART | SM00409:IG | 3 | 21.43 | 7.01E-02 | VCAN, IGLV2-14, IGKV3-20 | 10 | 531 | 10706 | 6.05E+00 | 6.11E-01 | 4.56E-01 | 4.56E-01 |
| KEGG_PATHWAY | hsa04657:IL-17 signaling pathway | 2 | 14.29 | 7.54E-02 | S100A9, S100A8 | 8 | 95 | 8534 | 2.25E+01 | 5.06E-01 | 3.46E-01 | 3.46E-01 |
| KEGG_PATHWAY | hsa04970:Salivary secretion | 2 | 14.29 | 7.69E-02 | CST3, LYZ | 8 | 97 | 8534 | 2.20E+01 | 5.13E-01 | 3.46E-01 | 3.46E-01 |
| UP_KW_BIOLOGICAL_PROCESS | KW-0145~Chemotaxis | 2 | 14.29 | 7.69E-02 | S100A9, S100A8 | 10 | 102 | 11523 | 2.26E+01 | 5.85E-01 | 2.12E-01 | 1.92E-01 |
| INTERPRO | IPR007110:Ig-like_dom | 3 | 21.43 | 8.11E-02 | VCAN, IGLV2-14, IGKV3-20 | 14 | 768 | 20808 | 5.81E+00 | 9.87E-01 | 5.17E-01 | 4.97E-01 |
| UP_SEQ_FEATURE | REGION:Complementarity-determining-3 | 2 | 14.29 | 8.40E-02 | IGLV2-14, IGKV3-20 | 14 | 139 | 20675 | 2.12E+01 | 9.99E-01 | 7.71E-01 | 7.71E-01 |
| UP_SEQ_FEATURE | REGION:Framework-1 | 2 | 14.29 | 8.51E-02 | IGLV2-14, IGKV3-20 | 14 | 141 | 20675 | 2.09E+01 | 9.99E-01 | 7.71E-01 | 7.71E-01 |
| UP_SEQ_FEATURE | REGION:Complementarity-determining-1 | 2 | 14.29 | 8.57E-02 | IGLV2-14, IGKV3-20 | 14 | 142 | 20675 | 2.08E+01 | 9.99E-01 | 7.71E-01 | 7.71E-01 |
| UP_SEQ_FEATURE | REGION:Framework-2 | 2 | 14.29 | 8.57E-02 | IGLV2-14, IGKV3-20 | 14 | 142 | 20675 | 2.08E+01 | 9.99E-01 | 7.71E-01 | 7.71E-01 |
| UP_SEQ_FEATURE | REGION:Complementarity-determining-2 | 2 | 14.29 | 8.57E-02 | IGLV2-14, IGKV3-20 | 14 | 142 | 20675 | 2.08E+01 | 9.99E-01 | 7.71E-01 | 7.71E-01 |
| UP_SEQ_FEATURE | REGION:Framework-3 | 2 | 14.29 | 8.57E-02 | IGLV2-14, IGKV3-20 | 14 | 142 | 20675 | 2.08E+01 | 9.99E-01 | 7.71E-01 | 7.71E-01 |
| GOTERM_BP_DIRECT | GO:0061844~antimicrobial humoral immune response mediated by antimicrobial peptide | 2 | 14.29 | 8.65E-02 | S100A12, S100A9 | 14 | 135 | 19478 | 2.06E+01 | 1.00E+00 | 4.50E-01 | 4.07E-01 |
| GOTERM_CC_DIRECT | GO:0072562~blood microparticle | 2 | 14.29 | 8.87E-02 | HBB, IGKV3-20 | 14 | 148 | 20795 | 2.01E+01 | 9.91E-01 | 3.48E-01 | 3.07E-01 |
